# Interactions of Monocytes, HIV, and ART Identified by an Innovative scRNAseq Pipeline: Pathways to Reservoirs and HIV-Associated Comorbidities

**DOI:** 10.1128/mBio.01037-20

**Published:** 2020-07-28

**Authors:** Rosiris León-Rivera, Brenda Morsey, Meng Niu, Howard S. Fox, Joan W. Berman

**Affiliations:** a Department of Pathology, Albert Einstein College of Medicine, Bronx, New York, USA; b Department of Neurological Sciences, University of Nebraska Medical Center, Omaha, Nebraska, USA; c Department of Genetics, Cell Biology & Anatomy, University of Nebraska Medical Center, Omaha, Nebraska, USA; Ragon Institute of MGH, MIT, and Harvard; Columbia University/HHMI

**Keywords:** HIV, reservoirs, latency, transcriptome, HIV-associated comorbidities

## Abstract

HIV reservoirs persist despite successful antiretroviral therapy (ART) and are a major obstacle to the eradication and cure of HIV. The mature monocyte subset, CD14^+^CD16^+^, contributes to viral reservoirs and HIV-associated comorbidities. Only a subset of monocytes harbors HIV (HIV^+^), while the rest remain uninfected, exposed cells (HIV^exp^). We developed an innovative single cell RNA sequencing (scRNAseq) pipeline that detects HIV and host transcripts simultaneously, enabling us to examine differences between HIV^+^ and HIV^exp^ mature monocytes. Using this, we characterized uninfected, HIV^+^, and HIV^exp^ primary human mature monocytes with and without ART. We showed that HIV^+^ mature monocytes do not form their own cluster separately from HIV^exp^ but can be distinguished by significant differential gene expression. We found that ART decreased levels of unspliced HIV transcripts potentially by modulating host transcriptional regulators shown to decrease viral infection and replication. We also identified and characterized mature monocyte subpopulations differentially impacted by HIV and ART. We identified genes dysregulated by ART in HIV^exp^ monocytes compared to their uninfected counterpart and, of interest, the junctional protein ALCAM, suggesting that ART impacts monocyte functions. Our data provide a novel method for simultaneous detection of HIV and host transcripts. We identify potential targets, such as those genes whose expression is increased in HIV^+^ mature monocytes compared to HIV^exp^, to block their entry into tissues, preventing establishment/replenishment of HIV reservoirs even with ART, thereby reducing and/or eliminating viral burden and HIV-associated comorbidities. Our data also highlight the heterogeneity of mature monocyte subsets and their potential contributions to HIV pathogenesis in the ART era.

## Abstract (precis)

HIV enters tissues early after infection, leading to establishment and persistence of HIV reservoirs despite antiretroviral therapy (ART). Viral reservoirs are a major obstacle to the eradication and cure of HIV. CD14^+^CD16^+^ (mature) monocytes may contribute to establishment and reseeding of reservoirs. A subset of monocytes, consisting mainly of CD14^+^CD16^+^ cells, harbors HIV (HIV^+^), while the rest remain uninfected, exposed cells (HIV^exp^). It is important to identify cells harboring virus to eliminate reservoirs. Using an innovative single-cell RNA sequencing (scRNAseq) pipeline to detect HIV and host transcripts simultaneously, we characterized HIV^+^ and HIV^exp^ primary human mature monocytes with and without ART. HIV^+^ mature monocytes are not a unique subpopulation but rather can be distinguished from HIV^exp^ by differential gene expression. We characterized mature monocyte subpopulations differently impacted by HIV and ART, highlighting their potential contributions to HIV-associated comorbidities. Our data propose therapeutic targets to block HIV^+^ monocyte entry into tissues, preventing establishment and replenishment of reservoirs even with ART.

## INTRODUCTION

As of 2018, approximately 38 million people worldwide were living with HIV (1) and, of those, about 23 million were on antiretroviral therapy (ART) (2). The life span of people living with HIV (PLWH) has significantly improved because of ART. Despite this, PLWH develop HIV-associated comorbidities, including HIV-associated neurocognitive disorders (HAND), cardiovascular disease, renal disease, metabolic syndrome, and malignancies. These negatively impact quality of life and contribute to increased morbidity (3). Viral reservoirs are established early after infection and persist within the gut, lymphoid tissues, bone marrow, and central nervous system (CNS) (4–11). These can contribute to HIV-associated comorbidities and are a barrier to eradication. ART is not a cure but is rather a lifelong treatment. Even when on ART, 10% to 50% of PLWH with virologic control show a transient increase in HIV RNA of at least 50 copies/ml, termed viral blips (12, 13), and discontinuation of ART leads to plasma viral rebound (14–16). This is because ART does not eliminate cells harboring virus but rather suppresses HIV replication and its ability to infect new cells. Viral reservoirs can produce viral proteins, including Nef and Tat, even with ART (17–21). Persistence of viral reservoirs, production of viral proteins, and viral blips contribute to development of comorbidities in PLWH (22–27). Thus, a key challenge to the development of therapeutic approaches to limit and, ultimately, to eradicate the establishment and reseeding of reservoirs and to cure HIV and HIV-associated comorbidities is that of characterizing the interplay between HIV and its reservoirs.

HIV mainly infects monocytes (28–34), T cells (14, 31, 35–37), dendritic cells (38–42), and macrophages (21, 34, 37, 43–49). Monocytes, specifically, the intermediate, or mature, subset consisting of those that express the lipopolysaccharide (LPS) coreceptor, CD14, and the FCγIII receptor, CD16 (CD14^+^CD16^+^), have been shown to be crucial to HIV pathogenesis and comorbidities. These mature monocytes are increased in peripheral blood of PLWH (50–52), are preferentially infected with HIV (28, 29, 53), and may contribute to establishing, reseeding, and maintaining viral reservoirs (49, 54–58).

Mature monocytes from PLWH are exposed to HIV, viral proteins, and/or inflammatory mediators. However, all are not actually infected with the virus. Some mature monocytes harbor virus (HIV^+^), and others remain uninfected as bystanders, or HIV-exposed cells (HIV^exp^). We previously showed that HIV^+^ mature monocytes, compared to HIV^exp^ mature monocytes, have increased levels of the surface junctional proteins (59) that are important for transmigration across the blood-brain barrier (BBB) into the CNS (60, 61). We demonstrated that this increase gives HIV^+^CD14^+^CD16^+^ monocytes a selective advantage in their ability to transmigrate across the BBB, potentially establishing and replenishing CNS viral reservoirs (59). However, these are only some of the proteins, in addition to CCR2 that, to our knowledge, have been shown to be differentially expressed (DE) between HIV^+^ and HIV^exp^ mature monocytes.

In this study, we performed single-cell RNA sequencing (scRNAseq) to characterize the transcriptomic profile of HIV^+^ and HIV^exp^ mature monocytes with and without ART to identify potential therapeutic targets that may limit entry into and continued replenishment of viral reservoirs by HIV^+^ mature monocytes and, ultimately, to eradicate them. We used primary human monocytes matured, infected with HIV_ADA_, and treated with ART *in vitro* and primary human monocytes matured in culture that remained uninfected. We developed a novel strategy that, to our knowledge, is the first in which HIV and host transcripts are identified concomitantly with and without ART and without use of green fluorescent protein (GFP)-tagged viruses or cell lines. We characterized HIV splicing patterns and distinguished HIV^+^ and HIV^exp^ mature monocytes in the presence and absence of ART. We demonstrate that HIV^+^ mature monocytes, with or without ART, do not form their own cluster distinct from that of their uninfected, exposed counterpart. Importantly, we show that HIV^+^ cells can be distinguished from HIV^exp^ cells on the basis of their differential gene expression. Additionally, HIV-infected mature monocytes with and without ART separated into discrete clusters, consisting of both HIV^+^ and HIV^exp^ cells, with differences in the percentages of HIV^+^ cells within each cluster, highlighting the heterogeneity of mature monocytes and of their ability to be infected. These data suggest that HIV may impact functions of mature monocyte clusters differently.

ART resulted in decreased levels of unspliced HIV transcripts within HIV^+^ mature monocytes, potentially by modulating upstream regulators shown previously to decrease viral infectivity (62–66). We also show varied ART gene dysregulation within specific clusters and expand upon these findings by comparing these genes between HIV^exp^ mature monocytes with and without ART and uninfected monocytes. Another notable finding is that following ART, one cluster may not be present. These data suggest that HIV and ART impact functions of mature monocyte clusters differently.

This report describes and highlights an innovative method to obtain simultaneous single-cell measurements of host and HIV transcriptomes and to characterize HIV-monocyte interactions, responses of HIV-infected mature monocytes to ART, and heterogeneity of mature monocytes. It provides a starting point for development of interventions targeting HIV^+^ mature monocytes, specifically by focusing on the multiple clusters that exist within the mature monocyte population with and without ART.

## RESULTS

### Detection by flow cytometry and scRNAseq of primary human HIV^+^ and HIV^exp^ CD14^+^CD16^+^ monocytes infected with HIV, with and without ART.

HIV infects monocytes, leading to seeding and reseeding of viral reservoirs in many different tissues. We recapitulate the heterogeneous mixture of HIV^+^ and HIV^exp^ cells, as evidenced by flow cytometry and scRNAseq, using a previously described culture method (59, 60, 67, 68). Mature monocytes were isolated, cultured, infected with HIV, and treated with ART as described below in Materials and Methods. For ART, we used a combination of tenofovir and emtricitabine, which is a commonly prescribed ART backbone for treatment of PLWH when used with other antiretrovirals and as preexposure and postexposure prophylaxis.

**FIG 1:**
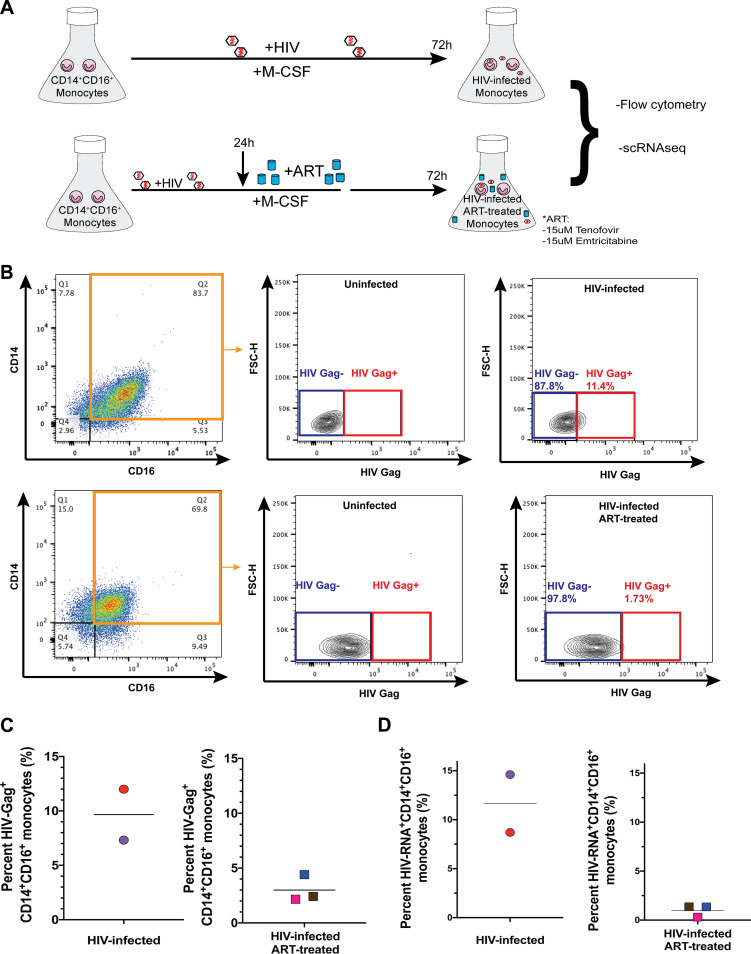
Detection by flow cytometry and scRNAseq of primary human HIV^+^ and HIV^exp^ CD14^+^CD16^+^ monocytes infected with HIV, with and without ART. (A) Experimental design. Monocytes are cultured nonadherently in Teflon-coated flasks with M-CSF to enrich to CD14^+^CD16^+^ (mature) monocytes. Mature monocytes are infected with HIV_ADA._ After 24 h, they are either treated with ART for 48 h or left untreated. HIV-Gag was detected by flow cytometry. Cells were also cryopreserved for scRNAseq. (B) Gating strategy for HIV-infected and HIV-infected ART-treated CD14^+^CD16^+^ monocytes to detect cells harboring productive HIV, HIV-Gag^+^CD14^+^CD16^+^, using their respective uninfected CD14^+^CD16^+^ monocytes to set the threshold of positivity for HIV-Gag^+^CD14^+^CD16^+^ monocytes, and cells not positive for productive infection, HIV-Gag^exp^CD14^+^CD16^+^, but exposed to HIV, viral proteins, and inflammatory mediators. FSC-H, forward scatter height. (C) Percentages of HIV-Gag^+^CD14^+^CD16^+^ monocytes from 5 independent leukopak donors (*n* = 2 for HIV-infected and *n* = 3 for ART-treated HIV-infected monocytes). Horizontal bars indicate the means. (D) Percent HIV^+^ mature monocytes from HIV-infected and HIV-infected ART-treated samples as determined by detection of HIV-transcripts using scRNAseq. Horizontal bars indicate the means.

After maturation and treatments, cells were divided into two portions, one for examining HIV Gag by flow cytometry and the other for scRNAseq analyses (Fig. 1A). Using flow cytometry to detect HIV Gag (Fig. 1B), we found that the mean proportion of HIV-Gag^+^CD14^+^CD16^+^ monocytes in HIV-infected monocytes was 9.66% (7.3% to 12%) (Fig. 1C) and in HIV-infected ART-treated monocytes was 3% (2.16% to 4.41%) (Fig. 1C).

For scRNAseq, cells were kept cryopreserved until use. To ensure that freeze/thawing did not cause cell loss, we stained for live-dead, surface CD14 and CD16, and HIV Gag before freezing (see Fig. S1A in the supplemental material) and after freezing (Fig. S1B). Our freezing and thawing did not cause cell death, as shown previously (69), with 86% to 100% viability postthawing. They also did not cause a loss of HIV Gag^+^ cells, since we detected similar amounts postthawing.

10.1128/mBio.01037-20.1 FIG S1 Detection of viable HIV^+^ and HIV^exp^ mature monocytes before and after freezing and thawing. The figure shows representative flow cytometric HIV-Gag protein staining of HIV-infected ART-treated CD14^+^ CD16^+^ monocytes before freezing and thawing (A) and after freezing and thawing (B) for scRNAseq. Download FIG S1, TIF file, 1.7 MB. Copyright © 2020 León-Rivera et al. 2020 León-Rivera et al. This content is distributed under the terms of the Creative Commons Attribution 4.0 International license.

### Our *in vitro* culture is comprised of viable mature monocytes.

Data from HIV-infected and HIV-infected ART-treated mature monocytes (*n* = 2 and *n* = 3, respectively) were normalized and integrated using the Seurat package (v3.1) in R. We performed joint graph-based clustering on integrated data sets and visualized results with the Uniform Manifold Approximation and Projection (UMAP) dimensionality reduction algorithm to verify that cells had grouped entirely by cell type, and batch effects were removed (see Fig. 5A). We confirmed that our cultured cells were mature monocytes by examining expression of cell-type markers and found a lack of coexpression of *CD14* and *LYZ*, markers coexpressed in less-mature CD14^+^CD16^−^ monocytes, and positive results indicating coexpression of *FCGR3A* and *MS4A7*, mature monocyte markers (data not shown). We did not find expression of *CD3D*, a T-cell marker, or of *CD19* and *CD79A*, B-cell markers. The cells did not coexpress *FCER1A* and *CST3*, conventional dendritic cell markers; *IL3RA*, *GZMB*, *SERPINF1*, and *ITM2C*, plasmacytoid dendritic cell markers; and *MARCO*, *ITGAM*, and *ADGRE1*, macrophage markers (data not shown). Additionally, to ensure that we did not include cells that were stressed/dying after freeze-thawing, we confirmed that no cluster coexpressed the heat shock genes *HSPB1*, *HSPH1*, and *DNAJB6* and DNA damage gene *GADD45B* (data not shown).

### Novel method for detection of HIV-1 transcripts using scRNAseq.

To detect HIV-RNA^+^ and HIV-RNA^exp^ mature monocytes, termed HIV^+^ and HIV^exp^, and to examine differences between them, we performed scRNAseq on monocytes that were infected with HIV (*n* = 2 independent biological samples), termed HIV-infected samples, and on those that were infected with HIV and treated with ART (*n* = 3 independent biological samples), termed HIV-infected ART-treated samples. Among all samples, we captured 28,362 cells after eliminating low-quality cells and multiplets, with means of 12,603 HIV-infected and 10,523 HIV-infected ART-treated monocytes.

**FIG 2:**
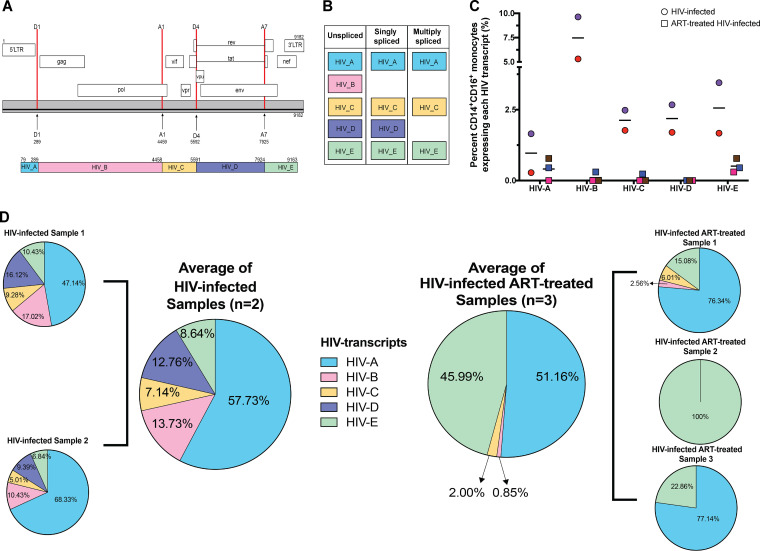
Detection of HIV^+^ mature monocytes from HIV-infected and HIV-infected ART-treated cultures using scRNAseq to show that ART decreases levels of unspliced HIV transcripts. (A) Schematic representation of HIV-1 genome annotations (top) and HIV transcript annotations (bottom) for detection of HIV transcripts. Locations of splice donors (D1 and D4) and acceptors (A1 and A7) for HIV-1 are indicated in the HIV-1 genome structure (top). These locations guided the novel HIV transcript annotation to detect HIV transcripts (HIV_A to HIV_E, bottom). Coordinates of the splice donor, splice acceptors, and HIV transcripts were determined based on the HIV genome. (B) Characterization of HIV transcripts based on known splicing. (C) Percentages of cells expressing each HIV transcript in HIV-infected and HIV-infected ART-treated mature monocytes. Circles are HIV-infected samples, and squares are HIV-infected samples treated with ART. Each circle or square is color coded as one of the different biological samples. Horizontal bars indicate means. (D) Pie chart of percent HIV transcripts captured and sequenced, normalized to the base pair length of each transcript. Data representing mean levels of HIV transcripts by treatment (HIV-infected samples *n* = 2, HIV-infected ART-treated samples *n* = 3) are also shown as pie charts. Each HIV transcript annotation is color coded.

We used several approaches to determine how to optimize detection of HIV transcripts. We first aligned transcripts to a combined genome representing human and HIV genomes, with the HIV genome used as the whole transcript without annotations for different HIV genes. However, this led to few reads mapping to HIV transcripts. We then annotated the HIV genome to detect each of its genes separately, increasing our level of detection of HIV^+^ cells but not to the optimal extent as indicated by our HIV Gag flow cytometry data, similarly to what others have reported (70). In our case, this was because the Cell Ranger pipeline needs to have a reference with only a small number of overlapping gene annotations and disqualifies reads that align to multiple genes. The individual HIV genes have overlapping sequences, and many were disqualified. To overcome this, we created a novel custom reference for the HIV genome (NCBI reference sequence accession no. NC_001802.1) by dividing it into 5 sections, termed HIV-A through HIV-E, creating gene annotations of well-characterized HIV splice sites (Fig. 2A and B).

The intact HIV genome contains long terminal repeat regions (LTR) at the 5′ and 3′ ends. In provirus DNA, these LTR are identical and are subdivided into sequential regions: U3, R, and U5. Transcription normally begins in the 5′ R region and ends after the polyadenylation signal at the 3′ R region, such that an unspliced, full-length genomic HIV RNA transcript contains two LTR that differ from each other (5′ RU5 and U3R 3′). To ensure that our custom reference did not have any overlap in gene annotations of the 5′ and 3′ LTR, we labeled as HIV-A the region of the genome from after the poly-A signal site in the 5′ LTR region to donor site 1 (D1) in the 5′ noncoding region, and those transcripts we captured as HIV-A can be any of the three types of HIV transcripts (unspliced, singly spliced, or multiply spliced). We chose D1 because it is used by all spliced HIV RNAs and all major splice products are produced from an event of direct splicing between D1 and one of the 10 acceptor sites. We annotated as region HIV-B the site from D1 to the first splice acceptor, A1. HIV-B transcripts are found only in either genomic RNA or unspliced mRNA (encoding Gag and Gag-Pro-Pol polyproteins). The region annotated as HIV-C is from A1 to the fourth splice donor, D4, and these transcripts can be either unspliced, singly spliced, or multiply spliced transcripts. The section annotated as HIV-D is from D4 to the seventh splice acceptor, A7, and these transcripts can be from unspliced or partially spliced transcripts but not from multiply spliced transcripts. The section annotated as HIV-E is from A7 to the end of the poly(A) signal sequence addition site in the 3′ LTR and these transcripts can be unspliced, partially spliced, or multiply spliced transcripts. We confirmed that those we identify as HIV transcripts were not due to false alignment by aligning reads of our uninfected cells to the same custom reference. The uninfected samples did not have any transcript that was identified as an HIV transcript, thus confirming that we detected specifically HIV transcripts in our HIV-infected and HIV-infected ART-treated samples. This novel custom reference ensured no overlap in gene annotations and provided splicing information about the HIV transcripts. Additionally, aligning to a combined human and HIV genome enabled characterization of host and viral responses in individual cells.

### Detection of HIV^+^ mature monocytes from HIV-infected and HIV-infected ART-treated cultures using scRNAseq.

After preprocessing the data, filtering out low-quality cells, and confirming the identity of our mature monocytes, we quantified the percentage of HIV^+^ monocytes, defined as any cell expressing any HIV transcript. The mean proportions of HIV^+^ mature monocytes in HIV-infected samples were 11.66% (interquartile range [IQR], 8.7% to 14.61%) (Fig. 1D) and 1.35% (IQR, 0.3% to 1.36%) in HIV-infected ART-treated samples (Fig. 1D).

To assess whether there were differences in the percentages of cells expressing the HIV RNA transcript annotations, we quantified the percentages of mature monocytes, including those from both the HIV-infected and HIV-infected ART-treated samples, expressing each HIV transcript (Fig. 2C). We did not find cells expressing any HIV transcript in uninfected samples. Mean percentages of mature monocytes positive for each HIV transcript were as follows: for HIV-A transcript, 0.9% (0.28% to 1.65%) in HIV-infected samples and 0.41% (0% to 0.78%) in HIV-infected ART-treated samples; for HIV-B transcript, 7.47% (5.31% to 9.63%) in HIV-infected samples and 0.1% (0% to 0.3%) in HIV-infected ART-treated samples; for HIV-C transcript, 2.13% (1.77% to 2.48%) in HIV-infected samples and 0.08% (0% to 0.23%) in HIV-infected ART-treated samples; for HIV-D transcript, 2.18% (1.7% to 2.67%) in HIV-infected samples and 0% in HIV-infected ART-treated samples; and for HIV-E transcript, 2.56% (1.67% to 3.45%) in HIV-infected samples and 0.51% (0.3% to 0.78%) in HIV-infected ART-treated samples. Using our novel method, this was the first time, to our knowledge, that HIV transcripts were detected and characterized by scRNAseq with and without ART in primary cells without the use of GFP-tagged viruses. Our data also show that the HIV-infected ART-treated mature monocytes had decreased percentages of HIV^+^ cells expressing any HIV segment transcripts (HIV-A to HIV-E) compared to those without ART.

### ART decreases unspliced HIV transcripts.

HIV transcript regions used in the gene annotations represented unspliced, singly spliced, and multiply spliced transcripts (Fig. 2B). HIV transcripts have differing lengths, and longer segments may have a higher probability of being captured and of having increased reads. Therefore, we normalized the counts of each HIV transcript to its base pair (bp) length (Fig. 2D). We found that there were various distributions of the percentages of normalized HIV transcripts segments captured in the HIV-infected samples, with a mean of 57.73% being HIV-A, 13.73% HIV-B, 7.14% HIV-C, 12.76% HIV-D, and 8.64% HIV-E. Thus, we likely captured all three of the HIV transcripts in HIV-infected mature monocyte samples. In contrast, the majority of HIV transcripts captured in the HIV-infected ART-treated samples were HIV-A (mean 51.16%) and HIV-E (mean 45.99%), and we captured HIV-B transcripts in only one of the HIV-infected ART-treated samples. These data show that our ART decreased unspliced mRNA similarly to what was reported in peripheral blood mononuclear cells (PBMC) of PLWH on ART (30, 71).

### HIV^+^ and HIV^exp^ mature monocytes have altered expression of genes related to cell movement of HIV^+^ mature monocytes.

**FIG 3:**
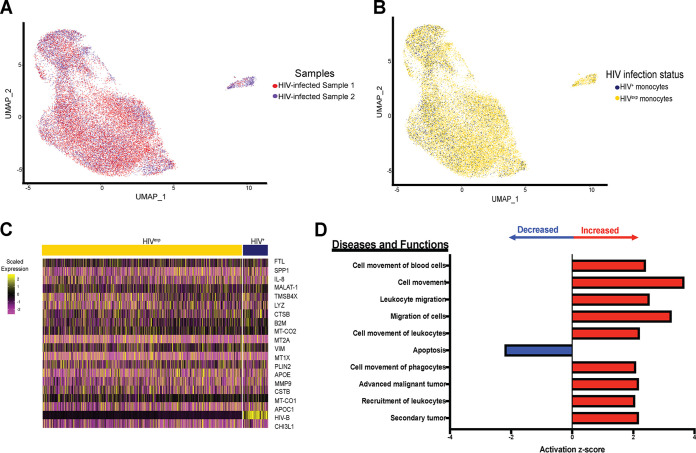
HIV^+^ and HIV^exp^ mature monocytes have altered expression of genes related to cell movement of HIV^+^ mature monocytes. (A and B) UMAP plots of HIV-infected mature monocytes from 2 different donors after integration of data sets and alignment. (A) Data are color coded by biological sample (red, HIV-infected sample 1; blue, HIV-infected sample 2). (B) Data are color coded by HIV infection status (HIV^+^ in blue, HIV^exp^ in yellow). (C) Heat map of top 20 DE genes in HIV^+^ relative to HIV^exp^ mature monocytes. The scaled expression of genes (rows) across HIV infection status (columns) is shown. (D) Top 10 diseases and functions predicted to be increased (red) or decreased (blue) in HIV^+^ relative to HIV^exp^ by IPA of DE genes.

We performed variable gene selection, dimensionality reduction, alignment and integration, and embedding across data collected from the HIV-infected samples (*n* = 2). Our analyses yielded no biological sample-specific features in the resulting clustering and embedding, suggesting that biology, rather than technical batch effects, was the main factor responsible for variations in and subsequent clustering of our data set (Fig. 3A). We found that the HIV^+^ cells did not form their own distinct group separate from the HIV^exp^ cells (Fig. 3B). Importantly, HIV^+^ mature monocytes have differentially expressed (DE) genes compared to those HIV^exp^ (Fig. 3C; see also Table S1 in the supplemental material). Ingenuity pathway analysis (IPA) of DE genes in comparisons between HIV^+^ and HIV^exp^ mature monocytes of the HIV-infected mature monocyte samples showed that HIV^+^ mature monocytes had altered expression of genes related to cell movement and apoptotic function (Fig. 3D).

10.1128/mBio.01037-20.4 TABLE S1 List of DE genes between HIV^+^ and HIV^exp^ mature monocytes from the integrated data set of HIV-infected mature monocytes without ART (HIV^+^ versus HIV^exp^ mature monocytes from HIV-infected mature monocyte cultures). Download Table S1, PDF file, 0.1 MB. Copyright © 2020 León-Rivera et al. 2020 León-Rivera et al. This content is distributed under the terms of the Creative Commons Attribution 4.0 International license.

### Percentages of HIV^+^ mature monocytes are different depending upon the cluster being examined.

**FIG 4:**
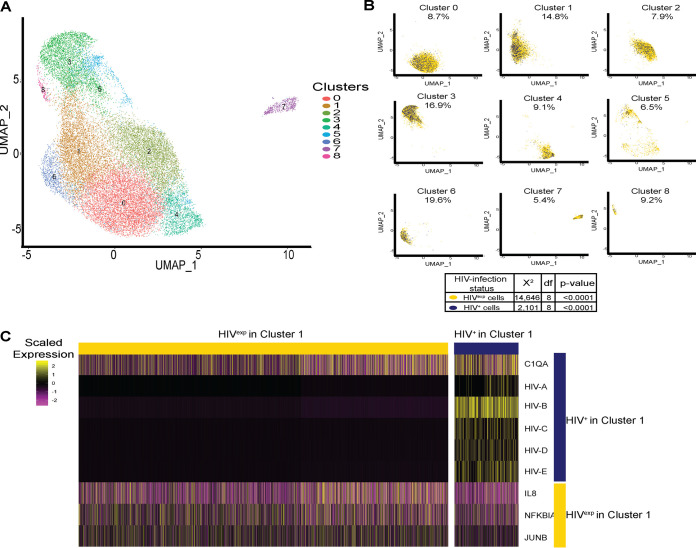
Percentages of HIV^+^ mature monocytes are different depending upon the cluster being examined. (A) UMAP plot of HIV-infected mature monocytes without ART from 2 different donors after integration of data sets and alignment, detecting 9 clusters of mature monocytes. (B) UMAP plot of HIV-infected mature monocytes color coded by HIV infection status (HIV^+^ in blue, HIV^exp^ in yellow) split by monocyte cluster. After alignment and integration, the percentages of HIV^+^ mature monocytes were different in each cluster. (C) Representative heat map of DE genes between HIV^+^ and HIV^exp^ mature monocytes from cluster 1. The scaled expression of genes (rows) across HIV infection status (columns) is shown.

Using data from the HIV-infected monocyte samples, we identified 9 different monocyte clusters (Fig. 4A). As the HIV-infected mature monocyte samples contained both HIV^+^ and HIV^exp^ mature monocytes, we examined the cell content and determined that the percentages of HIV^+^ cells differed per cluster (Fig. 4B). This distribution of HIV^+^ monocytes throughout the clusters is not stochastic (chi-square goodness-of-fit test, *P* value < 0.0001) but instead is a consequence of the fact that the cells clustered based on their biology. These data underscore that HIV^+^ mature monocytes are heterogeneous.

To determine differences between HIV^+^ and HIV^exp^ mature monocytes within a cluster from the HIV-infected samples, we performed DE gene analysis on each cluster. In all clusters, HIV^+^ mature monocytes were the only cells expressing HIV transcripts. We found 130 DE genes between HIV^+^ and HIV^exp^ mature monocytes from each cluster (Fig. 4C; see also Table S2). For example, comparing HIV^+^ and HIV^exp^ monocytes from cluster 1, the HIV^+^ cells had increased levels of complement C1q A chain, *C1qA*, and decreased levels of *IL-8*, *NFKBIA*, and *JUNB* compared to the HIV^exp^ cells from that same cluster (Fig. 4C). Decreased levels of *JUNB* and *NFKBIA* have been shown to play a role in monocyte transition to M2 (72). Increased *C1qA* levels have been shown to regulate markers of M2 macrophage polarization, such as CD163 (73). These results suggest HIV^+^ monocytes from cluster 1 may be acquiring a phenotype similar to that seen with M2 macrophages compared to HIV^exp^ monocytes from the same cluster. Thus, HIV may have been modulating monocytes in different ways depending upon which cluster was infected or may have been causing monocytes to cluster differently due to their infection/exposure differences.

10.1128/mBio.01037-20.5 TABLE S2 List of DE genes between HIV^+^ and HIV^exp^ in every cluster from the integrated data set of HIV-infected mature monocytes without ART (cluster X HIV^+^ versus cluster X HIV^exp^). Download Table S2, PDF file, 0.04 MB. Copyright © 2020 León-Rivera et al. 2020 León-Rivera et al. This content is distributed under the terms of the Creative Commons Attribution 4.0 International license.

### ART downregulates genes in HIV^+^ mature monocytes, decreasing HIV infection by modulating upstream regulators *E2F*, *E2F1*, *IFNA2*, and *let-7*.

**FIG 5:**
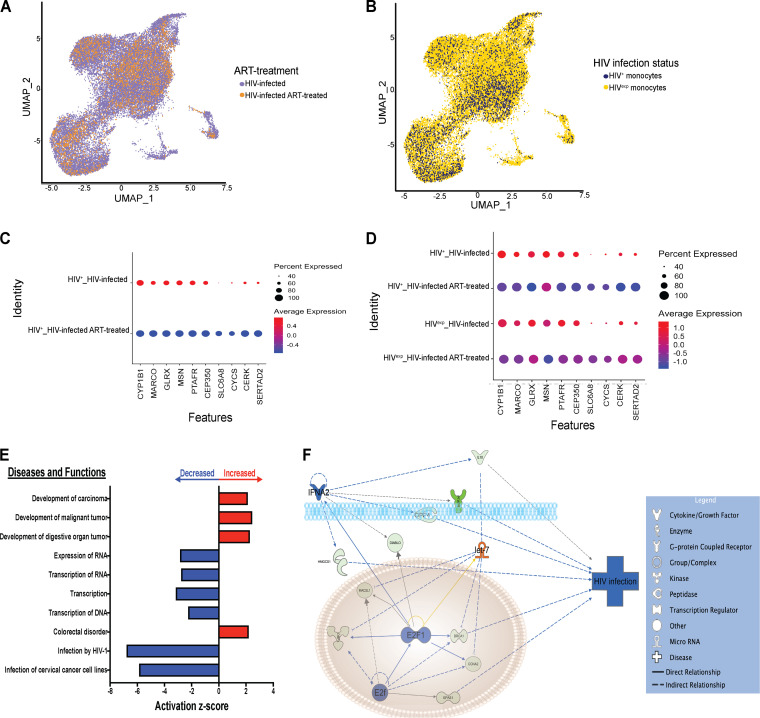
ART downregulates genes in HIV^+^ mature monocytes, decreasing HIV infection by modulating upstream regulators *E2F*, *E2F1*, *IFNA2*, and *let-7*. (A) UMAP plot of HIV-infected mature monocytes with and without ART after integration and alignment of data sets. (A) Data are color coded by treatment status (HIV-infected samples in purple, HIV-infected ART-treated samples in orange). (B) Data are color coded by HIV infection status (HIV^+^ in blue, HIV^exp^ in yellow). (C and D) Differential gene expression testing on integrated analysis showed DE genes in HIV^+^ cells with or without ART (C), and there was a trend indicating that expression levels of these same genes were decreased in HIV^exp^ cells with or without ART. (D) The size of each circle represents the percentage of cells in the cluster where the gene was detected. Color intensity reflects average expression within clusters (based on infection status and ART). (E) IPA diseases and functions of DE genes in HIV^+^ cells whose expression levels were predicted to be increased (red) or decreased (blue) in HIV^+^ cells with ART relative to HIV^+^ without ART (top 10 shown). (F) IPA network and upstream regulators associated with ART decrease of HIV infection. Upstream regulators are indicated in orange when leading to activation of downstream genes or functions or in blue when leading to inhibition. Genes whose expression levels were decreased with ART are indicated in green. Color intensity reflects average scaled expression.

Given that PLWH are most often prescribed ART, we addressed how ART may affect HIV^+^ monocytes. We normalized and performed variable gene selection, dimensionality reduction, alignment and integration, embedding, and joint graph-based clustering across data collected from HIV-infected samples (*n* = 2) and HIV-infected ART-treated samples (*n* = 3) and visualized results with UMAP (Fig. 5A). We show that our alignment strategy grouped cells by cell type and that HIV and monocyte biology, rather than technical batch or ART, underlay the variation and subsequent clustering in our data set. We also found that even after integrating HIV-infected monocytes with and without ART, the HIV^+^ cells did not form a unique cluster separate from the HIV^exp^ cells (Fig. 5B).

Having aligned our data sets of HIV-infected samples with and without ART, we compared how HIV^+^ mature monocytes differed in their responses to ART. Applying DE analysis to comparisons between HIV^+^ monocytes from the HIV-infected monocytes with ART and HIV^+^ monocytes from those without, we found that ART decreased expression of 731 genes in HIV^+^ mature monocytes (Fig. 5C; see also Table S3). Applying the same DE gene testing to comparisons between HIV^exp^ cells with and without ART, we did not find significance. However, examining the top 10 genes whose expression levels were decreased by ART in HIV^+^ monocytes within the HIV^exp^ monocytes, there was a trend toward decreased expression in the ART-treated HIV^exp^ monocytes compared to those without ART (Fig. 5D). The genes with decreases were associated with decreased expression of RNA, transcription, and infection by HIV, showing that ART may modulate gene expression that may reduce viremia, among other functions and pathways (Fig. 5E). Using IPA to examine the genes showing DE mediated by ART in HIV^+^ cells, we found that ART may decrease viral infection/replication by inhibiting *E2F*, *E2F1*, and *IFNA2* and by activating *let-7*, representing upstream regulators (Fig. 5F). ART may change the expression of these genes, leading to decreased viral infection/replication, and may be a cause of decreased levels of unspliced transcripts in HIV-infected ART-treated samples. Our chosen ART may not only function as nucleoside reverse transcriptase inhibitors (NRTIs) but also impact the monocyte transcriptome such that these cells are less likely to become infected.

10.1128/mBio.01037-20.6 TABLE S3 List of DE genes modulated by ART in HIV^+^ mature monocytes from the integrated data set of HIV-infected mature monocytes with and without ART (HIV^+^ with ART versus HIV^+^ without ART). Download Table S3, PDF file, 0.1 MB. Copyright © 2020 León-Rivera et al. 2020 León-Rivera et al. This content is distributed under the terms of the Creative Commons Attribution 4.0 International license.

### ART dysregulates genes within clusters, modulating monocyte functions and potentially affecting HIV-associated comorbidities.

**FIG 6:**
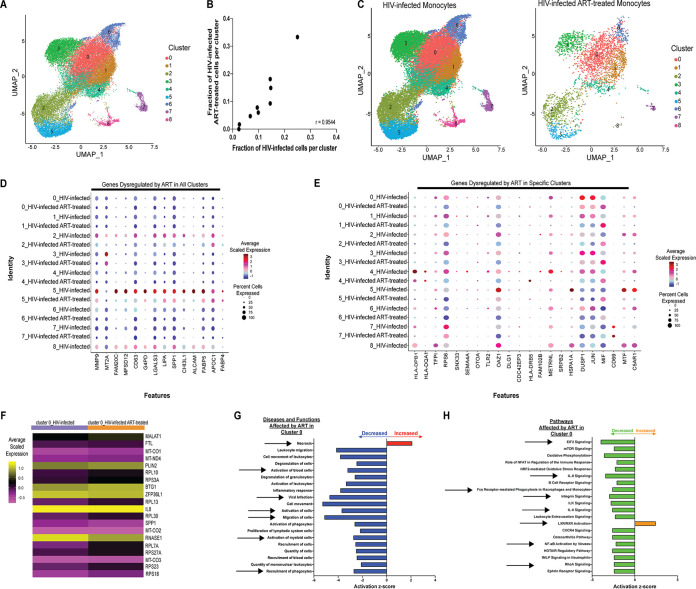
ART dysregulates genes within monocyte clusters, modulating monocyte functions and potentially affecting HIV-associated comorbidities. (A and C) UMAP plot of HIV-infected mature monocytes with and without ART after integration of data sets and alignment. Data are color coded based on monocyte cluster (A) and split by ART status (C). (B) Pearson’s correlation of fraction of mature monocytes (median across donors, *n* = 2 for HIV-infected samples and *n* = 3 for HIV-infected ART-treated samples) in each cluster (*n* = 9). (D and E) Integrated analysis of HIV-infected mature monocytes with and without ART identifies genes dysregulated by ART in all clusters (D) and in a cluster-specific manner (E). The size of the circle represents the percentages of cells in each cluster where the gene is detected. Color intensity reflects the average scaled expression within each cluster with and without ART. (F) Example of responses to ART in monocytes from cluster 0. Columns represent the average scaled expression of cells from that cluster subdivided by ART. (G and H) IPA of DE genes due to ART of monocytes from cluster 0 (top 20 changed shown) diseases and functions (G) and pathways (H) predicted to be increased (red for diseases and functions; orange for pathways) or decreased (blue for diseases and functions; green for pathways).

Our analysis of the HIV-infected and HIV-infected ART-treated samples showed 9 monocyte clusters, similar to the results seen when we aligned and integrated the HIV-infected samples only. We observed a similar proportional representation of 8/9 clusters in the HIV-infected cells with and without ART (*R* = 0.9544, Fig. 6B and C). Having aligned the data sets, showing that there were similar proportional levels of representation of HIV-infected and HIV-infected ART-treated samples in each cluster, we determined how ART may change gene expression in each cluster. Applying DE analysis separately for each monocyte cluster, we found that each exhibited significant gene expression changes due to ART (Table S4; shown in Fig. 6F for cluster 0). We identified 11 genes whose expression levels were decreased by ART in all clusters (*MMP9*, *MT2A*, *FAM20C*, *MFSD12*, *CD63*, *G6PD*, *LGALS3*, *LIPA*, *SPP1*, *CHI3L1*, and *ALCAM*) (Fig. 6D). Additionally, while *FABP4* and *APOC1* were dysregulated in all monocyte clusters, *FABP4* expression was decreased in all clusters except cluster 6, where it was increased, and *APOC1* expression was increased in clusters 0, 1, 3, and 4 and decreased in clusters 2, 5, 6, and 7 (Fig. 6D). We also found variance across clusters in responses to ART (Fig. 6E). These data show the heterogeneity of monocytes and of their responses to ART.

10.1128/mBio.01037-20.7 TABLE S4 List of differentially expressed markers modulated by ART on each mature monocyte cluster from the integrated data set of HIV-infected mature monocytes with and without ART (cluster X with ART versus cluster X without ART). Download Table S4, PDF file, 0.5 MB. Copyright © 2020 León-Rivera et al. 2020 León-Rivera et al. This content is distributed under the terms of the Creative Commons Attribution 4.0 International license.

To examine whether DE genes dysregulated by ART represented differential activity of specific biological diseases, functions, and pathways, we performed IPA. The results showed that ART enriched expression of genes involved in increased necrosis and decreased cell movement (shown in Fig. 6G for cluster 0) in all clusters. ART also appeared to increase apoptosis in clusters 1, 2, 5, 6, and 7 and to decrease viral infectivity in clusters 0, 2, 3, and 6 (Fig. 6G for cluster 0). Additionally, using IPA, we determined whether the genes dysregulated by ART in each cluster were affecting some of the same pathways (Fig. 6H). We found that these DE genes decreased activity in many pathways among multiple clusters (shown in Fig. 6H for cluster 0). These pathways included integrin-linked kinase (ILK) signaling (shared among clusters 0, 2, 5, and 6), interleukin-8 (IL-8) signaling (shared among clusters 0, 3, 5, and 7), integrin signaling (shared among clusters 0, 3, and 5), and eukaryotic initiation factor 2 (eIF2) signaling (shared among cluster 0 and 4); NRF2-mediated oxidative stress response (shared among clusters 0, 3, 5, 6, and 7); oxidative phosphorylation (shared among clusters 0, 2, 5, and 6); and FCγ receptor-mediated phagocytosis in macrophages and monocytes (shared with cluster 0 and 5) (Fig. 6H for cluster 0). Many other pathways were impacted by ART in each cluster independently and not shared among groups. These data show that ART may change monocyte functions and pathways differently depending on the cluster, and that each may contribute to HIV-associated comorbidities in the ART era, with some having a beneficial impact and others being more detrimental for PLWH.

### HIV-infected mature monocytes have a unique cluster that may not be detected with ART.

**FIG 7:**
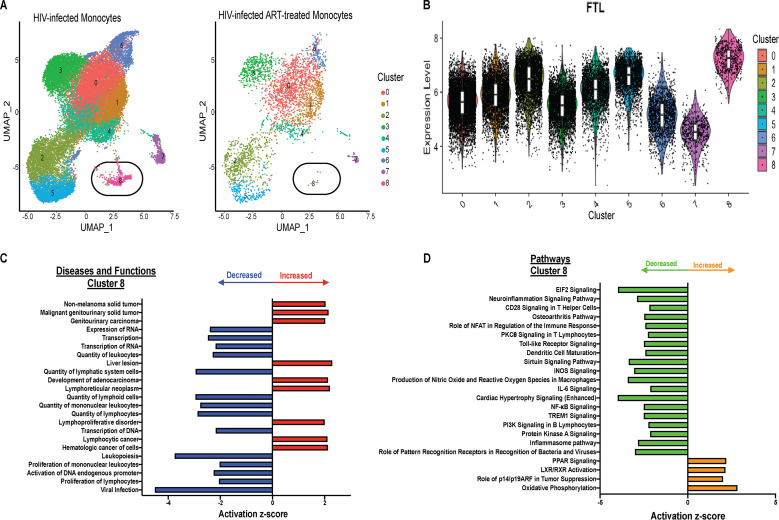
HIV-infected mature monocytes have a unique cluster that is not detected with ART. (A) UMAP plot of HIV-infected mature monocytes with and without ART after integration and alignment of data sets, color coded based on monocyte clusters and split by ART status. (B) Violin plot showing distribution of *FTL* expression in different clusters. White boxes represent interquartile range and median of *FTL* expression. (C and D) IPA of DE genes of cluster 8 compared to all other clusters, showing top diseases and functions (C) and canonical pathways (D) predicted to be increased (red for diseases and functions; orange for pathways) or decreased (blue for diseases and functions; green for pathways). iNOS, inducible nitric oxide synthase; PI3K, phosphatidylinositol 3-kinase; PPAR, peroxisome proliferator-activated receptor.

We identified a unique cluster, cluster 8, in the HIV-infected monocytes with 0.68% of cells. We did not detect this cluster in HIV-infected ART-treated monocytes (0%) (Fig. 7A). This population has the lowest percentage of HIV^+^ cells compared to other clusters (Fig. S3) and the highest expression of the gene encoding ferritin light chain, *FTL* (Fig. 7B). We performed DE gene analysis and found that this cluster had 1,795 DE genes compared to all other clusters (Table S5). Using IPA of these DE genes, we found they modulate diseases and cellular functions (Fig. 7C), including a decrease in the pathways associated with eIF2, neuroinflammation, IL-6, and Toll-like receptor signaling; production of nitric oxide and reactive oxygen species in macrophages; and inflammasome pathways (Fig. 7D). This decrease would likely be beneficial to the host, and their disappearance can have a negative impact, contributing to development of comorbidities in the ART era. This further underscores the heterogeneity of different monocyte subpopulations and their roles in HIV pathogenesis.

10.1128/mBio.01037-20.8 TABLE S5 List of DE genes between cluster 8 and all other monocyte clusters from the integrated data set of HIV-infected mature monocytes with and without ART (cluster 8 versus all other clusters). Download Table S5, PDF file, 0.04 MB. Copyright © 2020 León-Rivera et al. 2020 León-Rivera et al. This content is distributed under the terms of the Creative Commons Attribution 4.0 International license.

### HIV infection and ART may dysregulate genes important for monocyte function.

**FIG 8:**
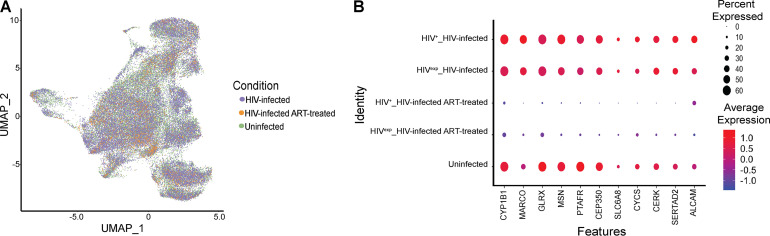
HIV infection and ART may dysregulate genes important for monocyte function. (A) UMAP plot of uninfected mature monocytes and HIV-infected mature monocytes with and without ART after integration and alignment of data sets. Data are color coded by culture condition (HIV-infected samples in purple, HIV-infected ART-treated samples in orange, and uninfected samples in green). (B) Differential gene expression testing on integrated analysis showed DE genes in HIV^+^ cells with or without ART (Fig. 5C and D), and there was a trend indicating that these same genes were dysregulated in HIV^exp^ cells with or without ART and compared with uninfected mature monocytes. The size of each circle represents the percentage of cells in the cluster where the gene is detected. Color intensity reflects average expression within clusters (based on infection status and culture condition).

Having found differences due to ART in HIV-infected mature monocytes, we addressed how ART may be modulating gene expression in the HIV^exp^ monocytes compared to uninfected mature monocytes. We normalized and performed variable gene selection, dimensionality reduction, alignment and integration, embedding, and joint graph-based clustering across data collected from uninfected samples (*n* = 2), HIV-infected samples (*n* = 2), and HIV-infected ART-treated samples (*n* = 3) and visualized results with UMAP (Fig. 8A). We found that even after integration of all data sets, the HIV^+^ cells still did not form a unique cluster separate from the HIV^exp^ cells or from the uninfected monocytes (data not shown). Applying DE analysis between HIV^exp^ monocytes with and without ART and the uninfected monocytes, we did not find any significantly differentially expressed genes. We previously had found trends in differential expression of genes between HIV^exp^ mature monocytes with and without ART (Fig. 5D). Therefore, we examined those genes under all integrated conditions to determine differences between HIV^exp^ mature monocytes with and without ART and uninfected mature monocytes. We found trends toward differences in gene expression in comparisons of HIV^exp^ mature monocytes with ART to the uninfected cells (Fig. 8B), suggesting that such differences may be subtle but distinctive. This is important given that ART may be contributing to the persistence of monocyte dysfunction present in PLWH in the ART era.

To understand potential effects of ART on a specific monocyte function, we examined surface levels of the junctional protein ALCAM, which is essential for monocyte transmigration across the BBB (59). Cultured uninfected and HIV-infected ART-treated mature monocytes were stained for CD14, CD16, and ALCAM; fixed; permeabilized; and stained and analyzed for HIV Gag positivity as described above and were termed HIV-Gag^+^ and HIV-Gag^exp^ to indicate antibody staining. Previously, we showed that HIV-Gag^+^CD14^+^CD16^+^ monocytes have increased ALCAM compared to their HIV-Gag^exp^CD14^+^CD16^+^ counterparts, providing a selective advantage to HIV-Gag^+^CD14^+^CD16^+^ monocytes to transmigrate across the BBB (59). Here, we show that HIV-Gag^+^CD14^+^CD16^+^ ART-treated monocytes have significantly higher surface levels of ALCAM (Fig. S2; *P < *0.05; *n* = 5 to 6) than do HIV-Gag^exp^CD14^+^CD16^+^ ART-treated monocytes. These data support our findings shown in Fig. 8B, in which HIV^+^ mature monocytes treated with ART are shown to have a higher percentage of cells expressing, and higher average expression of, ALCAM than their HIV^exp^ counterparts. These data suggest that even after ART, monocytes harboring virus may still have a selective advantage to transmigrate across the BBB or into other tissues given their increased levels of junctional proteins. While our study was not powered to compare surface ALCAM with uninfected mature monocytes given our sample size, we found a trend indicating that even after ART, ALCAM is still increased on the HIV-Gag^+^CD14^+^CD16^+^ ART-treated monocytes compared to the uninfected mature monocytes. Additionally, our data show a trend toward ART’s decreasing the levels of this junctional protein in HIV-Gag^exp^CD14^+^CD16^+^ ART-treated monocytes to below those of uninfected CD14^+^CD16^+^ monocytes. This is also illustrated in Fig. 8B, where HIV^exp^ mature monocytes treated with ART are shown to have had a smaller percentage, and lower average expression, of ALCAM than uninfected mature monocytes. These data show that ART may impact functions of HIV^exp^ monocytes.

10.1128/mBio.01037-20.2 FIG S2 Surface ALCAM are increased on HIV-Gag^+^CD14^+^CD16^+^ monocytes and appear to be decreased on HIV-Gag^exp^CD14^+^CD16^+^ monocytes, with ART. Surface ALCAM was analyzed by flow cytometry on uninfected CD14^+^CD16^+^ monocytes (*n* = 5) and HIV-Gag^+^ and HIV-Gag^exp^ CD14^+^CD16^+^ monocytes from HIV-infected ART-treated (*n* = 6) cultures from independent leukopak donors (data from each donor are color coded). ΔMFI (change in mean fluorescence intensity) was calculated by subtracting ALCAM MFI values from IgG1 isotype-matched control MFI values (*n* = 5 to 6). Data are represented as means ± standard errors of the means (SEM). Significance was determined by Wilcoxon matched-pair signed rank test. Significance is compared to baseline unless indicated otherwise. *, *P < *0.05. Download FIG S2, TIF file, 0.3 MB. Copyright © 2020 León-Rivera et al. 2020 León-Rivera et al. This content is distributed under the terms of the Creative Commons Attribution 4.0 International license.

10.1128/mBio.01037-20.3 FIG S3 Subpopulations of HIV-infected mature monocytes with and without ART have different percentages of HIV^+^CD14^+^CD16^+^ monocytes. The figure represents a UMAP plot of integrated HIV-infected CD14^+^CD16^+^ mature monocytes with and without ART, color coded by HIV infection status (HIV^+^ in blue, HIV^exp^ in yellow) and shown by monocyte cluster. Download FIG S3, TIF file, 1.2 MB. Copyright © 2020 León-Rivera et al. 2020 León-Rivera et al. This content is distributed under the terms of the Creative Commons Attribution 4.0 International license.

## DISCUSSION

Viral reservoirs are established early after a person is infected with HIV and persist despite ART. These reservoirs are a major obstacle to eradication and cure of HIV. Recent advances in scRNAseq have been used to examine transcriptional complexities associated with viral infection, viral latency, and cellular components associated with HIV-associated comorbidities (22, 70, 74–78). However, understanding host-HIV interactions at the single-cell level has been a distinct hurdle because only a small percentage of cells harbor HIV, and an even smaller number are transcriptionally active with ART, thus hindering the ability to capture and study reservoirs. To overcome this obstacle, researchers have used genetically modified viruses, such as those with GFP. They have also used latency-reversing agents and cell lines transfected with virus, having a larger percentage of transcriptionally active cells than is found physiologically. Some groups examined only the total population of cells and did not evaluate differences between the cells harboring virus and those that remained uninfected in studies of host-virus interactions (22, 70, 74–79). In this report, we describe what is, to our knowledge, the first time HIV has been detected by scRNAseq in primary human cells, even with ART, and without use of modifications described above. This custom HIV-alignment reference divides the HIV genome into 5 nonoverlapping sections and provides information about different HIV splice variants. Thus, we are able to distinguish mature monocytes harboring HIV and to characterize multiple HIV splice variants, even with ART. Here, we provide the first extensive characterization of primary HIV^+^ and HIV^exp^ mature monocytes with and without ART and host-virus interactions and the effects of ART on the mature monocyte transcriptome, all of which are important for development of novel therapeutic targets to eradicate HIV-infected cells and viral reservoirs.

Our initial characterization focused on HIV^+^ and HIV^exp^ mature monocytes without ART, since viral reservoirs are established early after peripheral infection, before individuals initiate ART (6, 37, 47, 80, 81). We found that HIV^+^ and HIV^exp^ mature monocytes do not separate into distinct clusters; instead, what distinguishes them is differential gene expression between the two populations. While challenging, using this differential expression will help develop therapeutic strategies to block establishment and reseeding of viral reservoirs and ultimately eliminate them.

The pathways of DE genes in HIV^+^ monocytes included increased cell movement and decreased apoptosis. These expand extensively upon previous data from our laboratory showing differential expression of junctional proteins on HIV^+^ mature monocytes. We now show that the level of junctional protein ALCAM is still increased in HIV^+^ mature monocytes even in the presence of ART, thus providing them with a selective advantage to transmigrate across the BBB or into other tissues by facilitating their movement (59). Additionally, some studies have shown an antiapoptotic phenotype prevalent in monocytes from PLWH, in nonhuman primate simian immunodeficiency virus (SIV) studies, and in *in vitro* and *ex vivo* studies with HIV-infected cells (82–85). All together, these data suggest potential mechanisms by which HIV modulates mature monocyte functions.

PLWH are now prescribed ART as soon as their HIV is detected. Thus, we examined the effect of a commonly prescribed ART backbone, consisting of tenofovir and emtricitabine, on mature monocytes, characterizing differences between HIV^+^ mature monocytes treated or not treated with ART. ART decreased unspliced HIV transcripts, similarly to what some have found in PBMC and CD4^+^ T cells of PLWH on ART (86–88). Tenofovir and emtricitabine are NRTI whose mechanism of action is to block the reverse transcription of HIV RNA to DNA. This decrease in unspliced HIV transcripts suggests that ART may also have some off-target effects within monocytes. We also found that ART decreased the expression of 731 genes in HIV^+^ monocytes compared to those without ART. Using IPA on these genes, we hypothesized that ART decreased HIV infection by modulating the mature monocyte transcriptome. One mechanism we propose is that ART changes the activity of upstream regulators *E2F*, *E2F1*, *IFNA2*, and *let-7*, possibly resulting in decreased HIV infection (62–66, 89). This suggests a molecular mechanism by which ART may contribute to viral latency by altering transcriptional regulators and by blocking later stages in viral transcription, such as splicing.

Two previous studies suggested that the total monocyte population is comprised of more than 3 subpopulations as assessed by single-cell PCR (90) and scRNAseq (91). We identified 9 monocyte clusters, 8 of which were shared between the HIV-infected monocytes with and without ART. Our findings indicate that mature monocytes may be further subdivided into multiple subpopulations in the context of HIV infection and ART. This led us to hypothesize that ART may impact each mature monocyte subpopulation differently. Comparing the effects of ART within each cluster, we identified DE genes in each and found that some of these were shared by more than one cluster. Expression of these genes is associated with movement (*MMP9*, *SPP1*, *ALCAM*, and others), viral infection (*APOC1*, *LGALS3*, *CD63*, and others), and recruitment of phagocytes (*APOE*, *CHI3L1*, *LGALS3*, and others). Additionally, some genes were differentially expressed in only one monocyte group such as *HLA-DPB1* in cluster 0, *SNX33* in cluster 1, *DLG1* in cluster 2, and *JUN* in cluster 6, among others.

One mature monocyte cluster, cluster 8, was not detected in HIV-infected ART-treated monocytes. This cluster is characterized by significantly increased expression of *FTL*. While there are few studies addressing its role in inflammatory responses, some studies suggested that the effect of increased *FTL* expression levels in murine macrophages may be anti-inflammatory by inhibiting LPS-induced reactive oxygen species (ROS) (92). In bone marrow-derived macrophages, *FTL* expression decreased levels of LPS-induced inflammatory cytokines (93). The increase in expression of *FTL* suggests a potential anti-inflammatory role for this monocyte cluster that is not present after ART. Using IPA on the DE genes in this population, we found that these are involved in decreasing functions that have been previously shown to be dysregulated in HIV-associated comorbidities. These results suggest that loss of this cluster with ART may be detrimental and may contribute to HIV-associated comorbidities, since these monocytes have a phenotype that, if present, may help reduce viremia and control inflammation. Additionally, they underscore that different mature monocyte subpopulations may be beneficial or detrimental in PLWH.

Comparing HIV^exp^ mature monocytes with and without ART to uninfected mature monocytes, we did not find any significantly differentially expressed genes, thus suggesting that the differences between the HIV^exp^ monocytes and their uninfected counterparts may be subtle. We did find interesting trends of decreased expression of genes involved in monocyte responses to pathogens (*MARCO*, *GLRX*, *PTFAR*, and *CERK*) and cytoskeletal reorganization and cell movement (*MSN* and *CEP350*). These findings suggest that ART may impact monocyte functions by dysregulating gene transcripts, sometimes decreasing their levels to below those seen with uninfected cells rather than restoring them to the uninfected levels, as had been suggested previously (94–97). Thus, ART might contribute to the monocyte dysfunction that persists in PLWH in the ART era, and ART-specific changes should be examined in future studies.

To our knowledge, we are the first to detect HIV with our novel alignment strategy without the use of GFP-tagged viruses, cell lines, or chemical modulators to increase the percentages of infected cells. Two limitations common to scRNAseq are that it is unable to detect latently infected monocytes, i.e., cells with integrated viruses that are not being actively transcribed, and that dropout events can occur that result in a reading of zero for expression of a gene that may indeed be expressed (98). Thus, our ability to detect HIV with high sensitivity in the scRNAseq data underscores the success of our method. Another caveat to this study is our use of freeze/thawing for our experiments. Others have used different methods for cryopreservation and compared their scRNAseq results with those from fresh cells. Those studies showed that the best method to preserve cells for scRNAseq is dimethyl sulfoxide (DMSO) cryopreservation (69), which is the method that we used. While we assayed for markers of cell death and stressed cells, we cannot preclude changes in gene expression associated with this methodology. Regardless, all cells were treated in this manner, across all conditions and comparisons. We report the first extensive characterization of primary mature monocytes in the context of HIV infection and ART through the use of a combination of transcriptomic and bioinformatic analyses. Additionally, we characterize many more cells than were characterized in previous studies examining viral reservoirs (22, 74, 75, 78, 79). Our results highlight the heterogeneity of the monocyte viral reservoir by demonstrating that some mature monocytes differ in their HIV infectivity, indicating that different monocyte subsets may have more favorable or less favorable cellular environments to establish HIV infection and that HIV and ART may impact mature monocyte clusters differently. We suggest that these differential responses to HIV and ART can be beneficial or detrimental in HIV pathogenesis.

Characterization of these mature monocyte subsets and their roles in infection and HIV-associated comorbidities is an important subject for future studies. Developing therapeutic strategies to prevent establishment and reseeding of viral reservoirs and to eliminate infection will require focusing on differential expression of genes, as well as on heterogeneity within HIV^+^ mature monocytes. We anticipate that our newly developed HIV-inclusive scRNAseq pipeline will be applied to previously and newly generated scRNAseq data sets to characterize further the heterogeneity of viral reservoirs and HIV-associated comorbidities. Our analysis serves as a valuable tool for understanding virus-host interactions and for characterization of the effects of ART on the transcriptome of HIV-infected mature monocytes, and suggests potential therapeutic targets for blocking formation and reseeding of viral reservoirs, thereby reducing and/or eventually eliminating viral burden, as well as HIV-associated comorbidities.

## MATERIALS AND METHODS

### Human subjects.

Leukopaks from anonymous individuals were obtained from the New York Blood Center. Institutional Review Board (IRB) approval for these studies was obtained from the Einstein Human Research Protection Program (HRPP) at Albert Einstein College of Medicine (IRB no. 1994-003).

### Viral stock production.

174XCEM cells (NIH AIDS Reagent Program; catalog no. 272) were cultured in RPMI 1640 1× (RPMI) (Gibco; catalog no. 11875-093) supplemented with 10% fetal bovine serum (FBS) (Gibco; catalog no. 16000-044) and 1% penicillin-streptomycin (Pen-Strep) (Corning; catalog no. 30-001-CI) at 37°C and 5% CO_2_. CEM cells were infected with 5 ng/ml of viral stocks prepared as described below from HIV_ADA_ (NIH AIDS Reagent Program; catalog no. 416). After 72 h, virus was removed and cells were pelleted by centrifugation and then cultured, splitting at 1:4 every 3 to 4 days. After 10 to 14 days postinoculation, cells were pelleted by centrifugation and supernatants containing virus were collected on a daily basis for a total of 28 days. After 28 days of collection, supernatant levels of HIV p24 were measured using high-sensitivity p24 AlphaLISA (PerkinElmer; catalog no. AL291C), and the supernatants from 3 to 4 collection days with the highest titers were pooled for virus purification. The supernatants collected on the selected days were combined and filtered through 0.45-μM-pore-size filters. The virus was then purified over a 20% sucrose (Sigma; catalog no. S-7903) cushion in 1× Dulbecco’s phosphate-buffered saline (PBS) (Corning; catalog no. 20-031-CV) by centrifugation at 25,000 rpm at 4°C, and the pellet was resuspended in monocyte media (RPMI supplemented with 10% human serum type AB [Corning; catalog no. 35-060-CI], 5% FBS, 1% Pen-Strep, 1% HEPES [Teknova; catalog no. H1030], and 10 ng/ml of recombinant human macrophage colony-stimulating factor [M-CSF] [PeproTech; catalog no. 300-25]). Levels of HIV p24 were then measured using high-sensitivity p24 AlphaLISA, and HIV p24 was diluted in monocyte media as necessary for future analyses of mature monocyte infections.

### Cell isolation and culture.

Isolation and culture of the mature monocytes were performed as described previously (59). Briefly, peripheral blood mononuclear cells (PBMCs) were isolated by Ficoll density gradient centrifugation (Ficoll-Paque Plus) (GE Healthcare; catalog no. 17144002) from leukopaks from anonymous donors obtained from the New York Blood Center. Monocytes were isolated from PBMCs using magnetic bead-positive selection (EasySep human CD14-positive separation kit I) (Stem Cell Technologies; catalog no. 18058). Freshly isolated CD14 monocytes were then cultured for 2 days in Teflon-coated flasks at 2 × 10^6^ cells/ml in monocyte media with M-CSF (10 ng/ml) at 37°C and 5% CO_2_, facilitating maturation and enriching to 60% to 90% CD14^+^ CD16^+^ monocytes.

### HIV infection and ART of mature monocytes.

After the monocytes were cultured for 2 days, they were then divided into groups of cells that were to be left uninfected, infected with HIV, or infected with HIV and subsequently treated with ART. Cells that were to be infected with HIV, with and without subsequent treatment with ART, were resuspended to 10 × 10^6^ cells/ml in fresh monocyte media, inoculated with 1 μg p24/ml of HIV_ADA_ stock, and cultured nonadherently in Teflon-coated flasks for 8 h. After 8 h, virus was removed by centrifugation of the cells, and monocytes were resuspended to 2 × 10^6^ cells/ml in fresh monocyte media and cultured nonadherently in Teflon-coated flasks. Uninfected cells were resuspended to 10 × 10^6^ cells/ml in fresh monocyte media and cultured nonadherently in Teflon-coated flasks for 8 h. The uninfected and the HIV-infected mature monocytes that did not receive subsequent ART were cultured for an additional 64 h. The HIV-infected monocytes in the subset used for ART were cultured for an additional 18 h to facilitate viral replication and infection before the addition of ART. After the 18 h, the drugs used for ART (15 μM emtricitabine [NIH AIDS Reagent Program; catalog no. 10071] and 15 μM tenofovir [NIH AIDS Reagent Program; catalog no. 10199]) were added to the media of these HIV-infected mature monocytes and the cells were then cultured nonadherently in Teflon-coated flasks for an additional 46 h. After a total of 72 h postinfection, uninfected, HIV-infected, and HIV-infected ART-treated cells were divided into two aliquots: one for examining HIV Gag expression by flow cytometry and another to be cryopreserved for single-cell RNAseq (scRNAseq). For scRNAseq cells, 5 × 10^6^ cells/cryotube were frozen in filtered FBS with 10% DMSO (0.2-μm-pore-size filters) until use.

### HIV Gag expression by flow cytometry.

Mature uninfected, HIV-infected, and HIV-infected ART-treated monocytes were stained using antibodies specific for human CD14 conjugated with allophycocyanin (APC) (BD Biosciences; clone M5E2; catalog no. 555399) (4:50 dilution) or CD14 conjugates with fluorescein isothiocyanate (FITC) (BD Biosciences; clone M5E2; catalog no. 555397) (5:50 dilution) and human CD16 conjugated to phycoerythrin/Cy7 (PE-Cy7) (BD Biosciences; clone 3G8; catalog no. 557744) (4:50 dilution). The junctional protein ALCAM was examined as described previously (59), using biotinylated ALCAM (R&D; clone 105902; catalog no. BAM6561) (0.25 μg). Strepavidin-APC (eBioscience; catalog no. 17-4317-82) (0.15 μg) was used as a secondary reagent for biotinylated ALCAM, and corresponding isotype-matched negative-control antibodies were also used. Fluorescence minus one (FMO) controls were included as described previously (59). Calculation of the titers of antibodies was performed to obtain and determine the optimal staining concentrations. Cells (4 × 10^5^ for conditions of permeabilization in 100 μl) were stained in the dark on ice for 30 min, washed once, and fixed with 2% paraformaldehyde (Electron Microscopy Sciences; catalog no. 15714) for 20 min. After fixation, cells were washed and permeabilized with 0.1% Triton X-100 (Sigma; catalog no. T8787)–1× PBS for 5 min and were stained in 100 μl of 1% bovine serum albumin (BSA) (US Biological Life Sciences; catalog no. A1324)–1× PBS with a 1:40 dilution of anti-HIV-1 Gag core antigen antibody conjugated to RD1 (Beckman Coulter; clone KC57-RD1; catalog no. 6604667) (1:40 dilution). At least 10,000 events were acquired with a BD FACSCanto II flow cytometer in the Albert Einstein College of Medicine Flow Cytometry Core Facility. Analysis was performed using FlowJo software (Treestar; v10.6.1). Uninfected CD14^+^ CD16^+^ monocytes from the same donor were used to set the threshold of positivity for HIV-Gag^+^ CD14^+^ CD16^+^ monocytes.

### Preparation of mature monocyte suspensions for scRNAseq.

Frozen mature monocytes were thawed by being placed in a 37°C water bath without agitation until only a small ice crystal was left in the vial. A 1-ml volume of prewarmed DNase-supplemented media (RPMI supplemented with 20% FBS and 1% DNase I [Sigma; catalog no. 10104159001]) was then added using filtered tips. The samples were gently mixed by pipetting and allowed to rest for 5 min at room temperature. After the 5 min, the contents of each vial were transferred to 10 ml of prewarmed DNase-supplemented media and incubated for an additional 5 min at room temperature. The cells were then centrifuged, the supernatant was aspirated, and the mature monocytes were resuspended in 1 ml of RPMI supplemented with 20% FBS and incubated for 15 min at 37°C with 5% CO_2_. After incubation, the cells were counted, centrifuged, and divided into two subsets, one used for staining post-freeze/thawing to verify viability on the day of scRNAseq and the other for scRNAseq.

### Flow cytometry after freeze/thawing to determine efficacy of freeze/thaw protocol and before scRNAseq.

To ascertain that cells were viable for scRNAseq, the cells were thawed as described above. Cells were spun and resuspended at 1 × 10^6^ cells/ml in 1× PBS, and 1 μl/ml of reconstituted fluorescent reactive dye from a LIVE/DEAD fixable blue dead cell stain kit for UV excitation (Thermo Fisher Scientific; catalog no. L23105) was added followed by incubation for 30 min on ice protected from light. After incubating, the cells were washed once and incubated for 45 min on ice and in the dark with a cocktail consisting of Horizon brilliant stain buffer (BD Biosciences; catalog no. 563794) (60 μl) human CD3 conjugated to Brilliant Violet 421 dye (BV421) (BD Biosciences; clone UCHT1; catalog no. 562877) (5 μl), human CD14 coupled to electron coupled dye (ECD) (Beckman Coulter; clone RM052; catalog no. IM2707U) (5 μl), human CD11b coupled to BV605 dye (Biolegend; clone M1/70; catalog no. 101237) (5 μl), human CD16 conjugated to APC-H7 (BD Biosciences; clone 3G8; catalog no. 560195) (5 μl), human CD163 conjugated to BV511 dye (BD Biosciences; clone GHI/61; catalog no. 744921) (5 μl), human CD56 conjugated to Alexa Fluor 700 (AF700) (BD Biosciences; clone B159; catalog no. 557919) (5 μl), and human CD19 conjugated to FITC (BD Biosciences; clone SJ25C1; catalog no. 562947) (5 μl) and their respective isotype and FMO controls. Cells were then washed once and fixed with 2% paraformaldehyde. At least 10,000 events were acquired on an LSRFortessa flow cytometer in the University of Nebraska Medical Center (UNMC) Flow Cytometry core facility machine. Analysis was performed using FlowJo software.

### scRNAseq.

The scRNAseq cDNA libraries were prepared following the user guide manual provided with 10X Genomics Chromium single-cell 3′ reagent kits (v2 for HIV-infected ART-treated mature monocytes and v3 for HIV-infected mature monocytes). The libraries for the three HIV-infected ART-treated independent biological samples were each run individually on four lanes of sequencing on an Illumina NextSeq 550 instrument, yielding a mean of 215,912 reads per cell. For the HIV-infected independent biological samples, they were each divided in half for library preparation. The pooled libraries for one of the two HIV-infected independent biological samples were run on four lanes for sequencing on an Illumina NextSeq 550 instrument, and the pooled libraries for the second HIV-infected independent biological sample were run on two lanes on an Illumina NovaSeq instrument. The two HIV-infected samples combined yielded a mean of 56,416 reads per cell. For the uninfected independent biological samples, they were each divided in half for library preparation. The pooled libraries were run on two lanes for sequencing on an Illumina NovaSeq instrument, yielding a mean of 69,750 reads per cell. Library preparation was performed at the UNMC Genomics Core Facility, and initial bioinformatic analysis was performed by the UNMC Bioinformatics and Systems Biology Core.

First, we aligned the sequenced reads using the CellRanger analysis pipeline (10X Genomics; V3.0.0), which integrates the STAR aligner (99), to a combined human genome (hg19) and the HIV genome as one gene (NCBI reference sequence accession no. NC_001802.1), but this yielded few reads aligning to the HIV genome. We then aligned the sequenced reads to a combined human and HIV genome, with the transcripts for the nine main proteins indicated as nine HIV genes. While this improved our detection of HIV transcripts, it was still not optimal since we detected very few HIV^+^ cells compared to our HIV Gag p24 data. We then aligned the sequenced reads using the CellRanger analysis pipeline with a combined human genome and our novel HIV genome subdivided into 5 transcripts, as described in Results (“Novel method for detection of HIV-1 transcripts using scRNAseq”). Downstream analyses of the data sets were performed using the Seurat (v3.1.4) (100) R package (v3.6.1). Samples were individually filtered to exclude cells expressing fewer than 200 unique genes, cells with genes not expressed in a minimum of 3 cells, and cells with a high (>15%) fraction of mitochondrial molecules, thus leaving 55,339 cells for analysis.

Our samples were individually preprocessed using the sctransform function (v0.2.0) in Seurat, which normalizes and stabilizes the technical noise variance found in raw UMI (unique molecular identified) counts for each cell individually by using a regularized negative binomial regression. We included in the function settings to regress out the effects of the fraction of mitochondrial genes being expressed in each cell to mitigate the influence of this source of variation on the data set and to preserve the option of returning all the genes, not only the highly variable genes. Sctransform normalizes and stabilizes the technical noise variance of UMI counts by using a regularized negative binomial regression (101). After normalization of the samples, they were integrated using the Seurat correction to align the data sets as described previously (100). In brief, we chose to integrate the union of the genes found in all of the data sets. We used the PrepSCTIntegration function in Seurat to ensure that all the necessary Pearson’s residual values had been calculated during the normalization done by the sctransform function. We found anchors in which to integrate the data set by using the FindIntegrationAnchors function, and, last, we integrated the data sets using the IntegrateData function, setting the features to integrate as all the genes shared between the data sets. The IntegrateData function aligned the data set using a variation on canonical correlation analysis, learning a correlation structure of shared genes conserved between the data sets to facilitate identification of individual cells that cannot be described by this shared structure, enabling us to identify the rare populations of cells that might not overlap in the data sets.

Dimensionality reduction was performed using principal-component analysis (PCA) based on the integrated data set. For visualization purposes, we chose to use the Unifold Manifold Approximation and Projection (UMAP) dimensionality reduction algorithm. To choose the dimensionality to include in UMAP, we calculated a threshold where the principal components cumulatively contribute 90% of the standard deviation and the point at which the percent change in variation between consecutive principal components is less than 0.1% and confirmed by using an ElbowPlot. We chose to perform UMAP using the first 20 principal components in analyzing the 2 HIV-infected biological samples and the first 45 principal components in analyzing all 5 HIV-infected samples (2 HIV-infected and 3 HIV-infected ART-treated independent samples) and in analyzing all 7 samples (2 uninfected independent samples, 2 HIV-infected independent samples, and 3 HIV-infected ART-treated independent samples). Clustering was performed using the Seurat default graph-based clustering approach, the Louvain algorithm, and the FindClusters function with a resolution range of 0.5. The resultant clusters were then visualized using UMAP.

### Quantification and statistical analysis.

**(i) Quantification of the number of HIV transcript segments per sample.** To quantify the number of HIV transcripts captured and sequenced in each sample, we obtained the normalized counts of each HIV segment transcript per sample captured, normalized each to its base pair (bp) length, and divided this number by the total number of normalized HIV transcript counts captured for each sample.

**(ii) Statistical analysis for differential gene expression.** Differential gene expression testing was performed on the Pearson’s residual values after regularized negative binomial regression using independent *t* tests per gene for all genes detected in at least 3 cells in at least one of the two groups being compared. *P* values were adjusted using the Benjamini & Hochberg method as described previously (101). To determine the significance thresholds, we calculated the random background distribution of the mean differences of the Pearson’s residual values after regularized negative binomial regression of 1,000 genes and permuted by the group identities. We then choose a significance threshold of anything above the 0.5th and 99.5th percentiles of the background distribution of the mean differences of the Pearson’s residual values after regularized negative binomial regression and if the false-discovery rate (FDR) was below 0.01. The lists of significant genes were then analyzed using Ingenuity pathway analysis (IPA) software (Qiagen). Any pathways or diseases and functions with an activation Z-score of >2 or ≤2 were determined to be significantly activated/increased or inhibited/decreased, respectively.

**(iii) Graphical representation of IPA and statistical analysis.** Statistical analyses were performed using GraphPad Prism (v8.3.0). Details pertaining to the number of replicates, sample size, significance tests, and value and meaning of *n* for each experiment are included in Materials and Methods, Results, or the figure legends. The researcher performing the preparation for scRNAseq, capturing of the samples, sequencing, and alignment of the data sets was blind to the treatments and conditions of the samples during the experiments.

### Data availability.

The data sets generated during and/or analyzed during the current study are available from the lead contact upon reasonable request. Raw and processed scRNAseq data are available from GEO under accession GSE153958.
