# Supplementary figures and images for: Interactions of Monocytes, HIV, and ART Identified by an Innovative scRNAseq Pipeline: Pathways to Reservoirs and HIV-Associated Comorbidities
Source: mBio. 2020 Jul 28;11(4):e01037-20. doi: 10.1128/mBio.01037-20 (PMC7387797; doi:10.1128/mBio.01037-20)

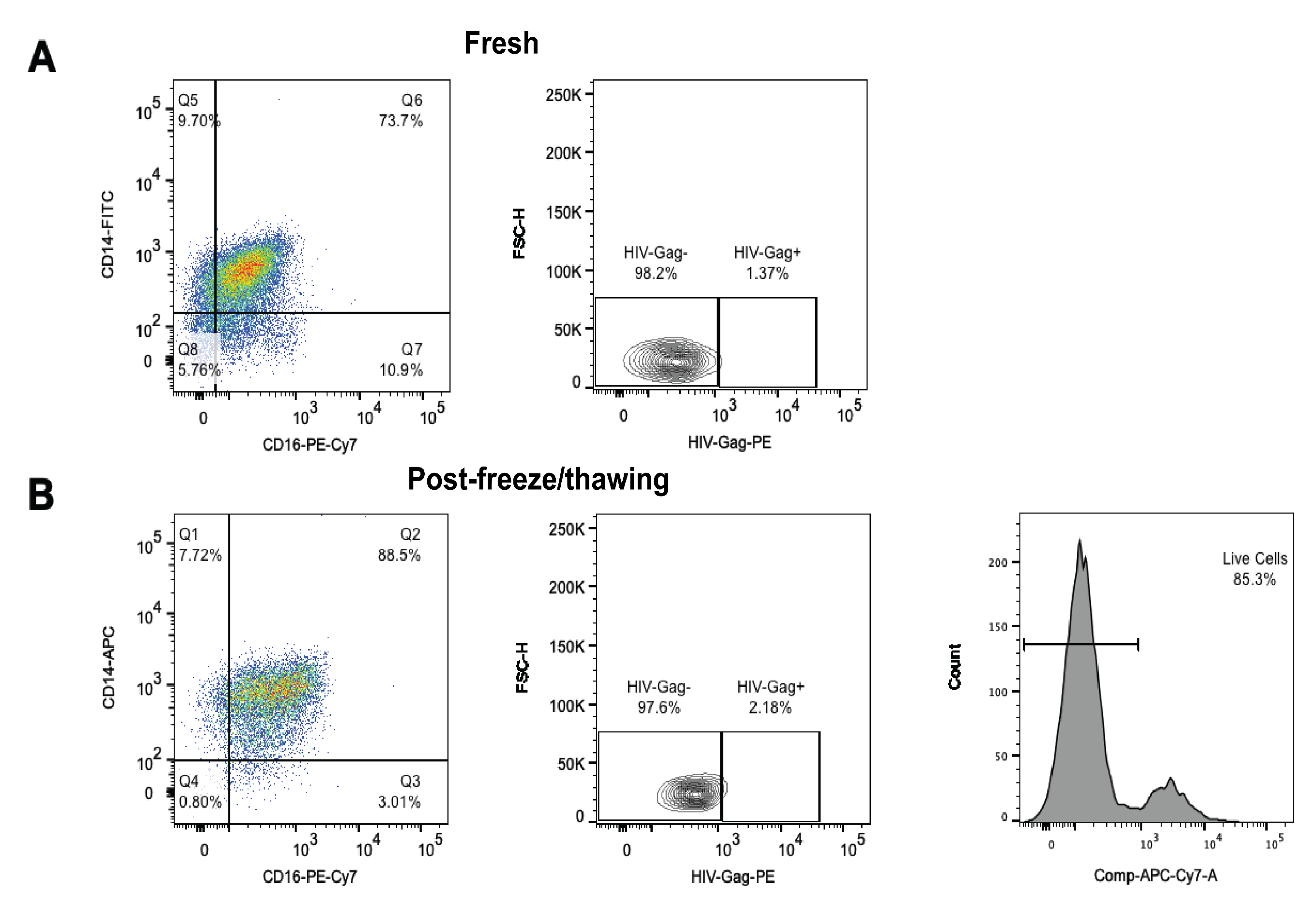

Supplement: FIG S1 [file mBio.01037-20-sf001.tif]

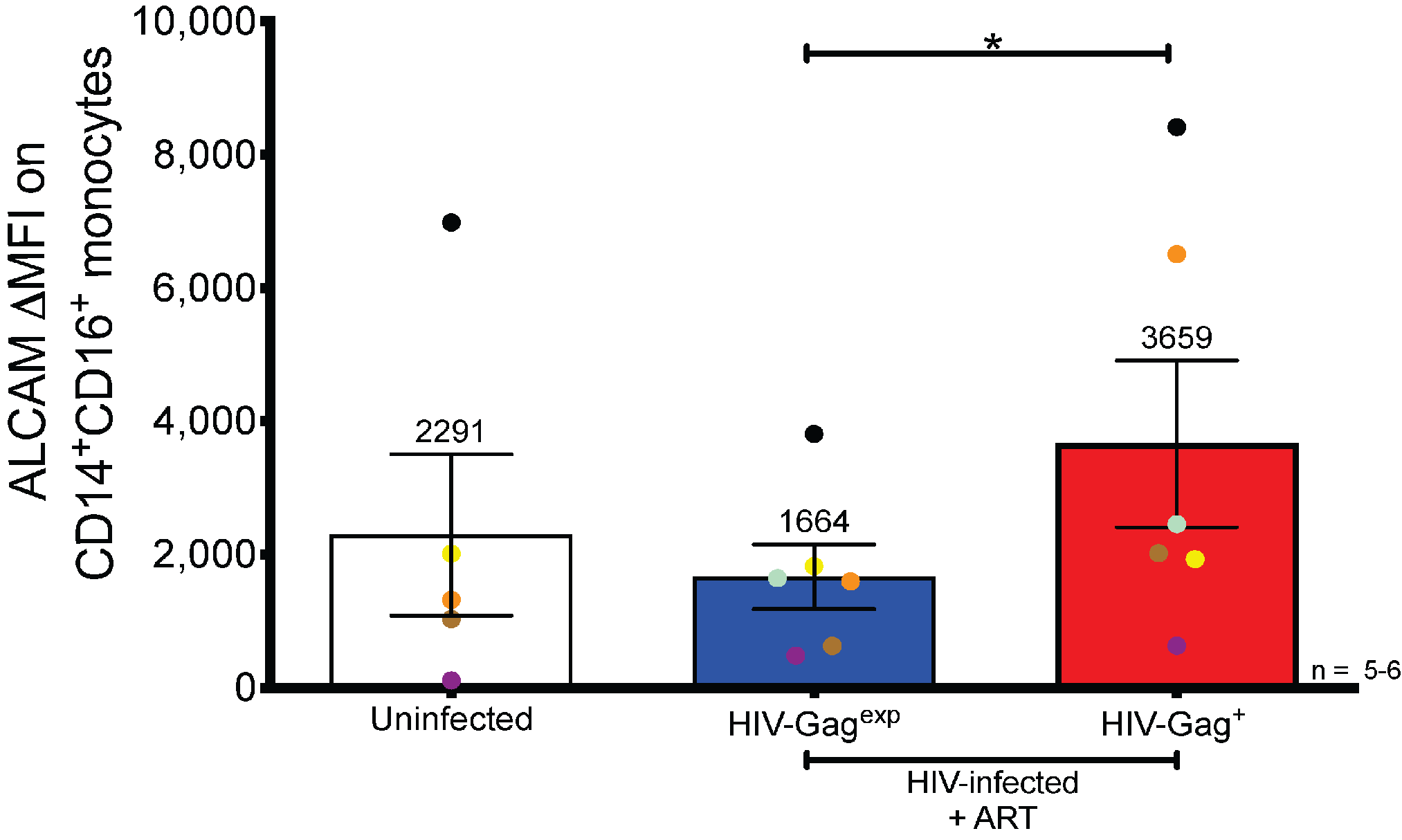

Supplement: FIG S2 [file mBio.01037-20-sf002.tif]

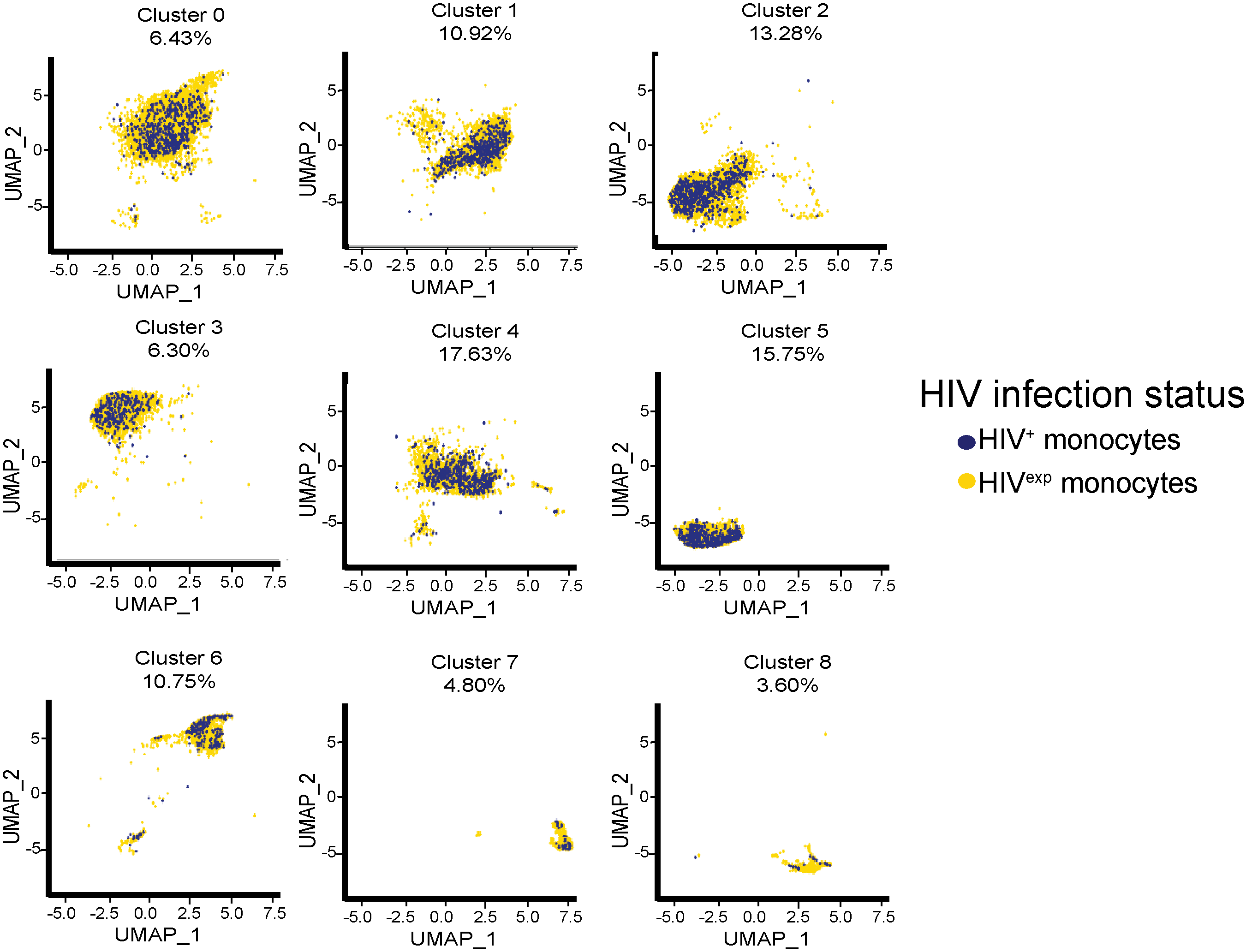

Supplement: FIG S3 [file mBio.01037-20-sf003.tif]
