# Supplementary material for: Interactions of Monocytes, HIV, and ART Identified by an Innovative scRNAseq Pipeline: Pathways to Reservoirs and HIV-Associated Comorbidities
Source: mBio. 2020 Jul 28;11(4):e01037-20. doi: 10.1128/mBio.01037-20 (PMC7387797; doi:10.1128/mBio.01037-20)
Supplement: TABLE S1 [file mBio.01037-20-st001.pdf]

Supplementary Table 1: List of differentially expressed genes between HIV+ and HIV^{exp} mature monocytes from the integrated dataset of HIV-infected mature monocytes without ART-treatment (HIV+ vs HIV^{exp} mature monocytes from HIV-infected mature monocyte cultures).

Gene.ID	Avg. Diff.[1]	Pct.1[2]	Pct.2[3]	P.Value.Adj
FTL	81.3246896	1	1	0.000369336
SPP1	39.2708578	0.997	0.997	1.96331565977059E-21
TMSB4X	12.8485068	1	1	8.816793909191E-24
CTSB	8.67478848	0.994	0.995	3.4405057409309E-30
B2M	7.44177283	1	1	1.6526132342616E-11
MT-CO2	7.20416407	0.987	0.987	2.99743751410066E-22
VIM	7.10976963	1	1	1.71459778438076E-08
APOE	5.7509563	0.848	0.823	4.52015140344873E-24
MMP9	5.27816015	0.936	0.951	3.99286063884207E-14
CSTB	4.8180845	1	1	1.26805115910356E-05
MT-CO1	4.64831762	0.998	0.999	0.006130412
APOC1	4.59031607	0.835	0.788	1.97652482648866E-20
HIV-B	4.05017544	0.584	0	1.09116955338285E-275
CHI3L1	4.04457286	0.435	0.359	1.19060876979202E-21
HLA-B	4.02082395	1	0.999	1.8518182802388E-08
FABP5	3.99094233	0.727	0.695	1.17363259762542E-10
LGALS1	3.92034036	0.999	0.999	8.86451226845659E-35
S100A11	3.70162286	0.999	0.997	3.0966307834642E-08
RPLP1	3.646144	1	1	5.94099293022589E-13
RPS2	3.60425063	1	1	1.78304866256009E-12
CD52	3.312556	0.984	0.983	3.73107257708106E-12
SERF2	3.210584	1	1	6.77465453162876E-18
LGALS3	3.07395253	0.994	0.993	0.000105333
MT-CO3	2.96139786	0.995	0.994	1.22303986402377E-14
LIPA	2.72787398	0.739	0.657	1.35431748969236E-24
RPS18	2.64109258	1	1	1.30320117340566E-09
CD63	2.64089073	0.995	0.997	1.9579969188333E-06
RPS19	2.46779056	1	1	4.48223041138979E-06
MT-ND1	2.38305681	0.974	0.978	7.65533320542562E-10
HCST	2.34547552	0.944	0.929	1.48128316603217E-10
RPS12	2.13044329	0.999	1	7.01611580203258E-07
CST3	2.06659266	0.97	0.936	2.1328755583256E-40
HIV-E	1.95438596	0.205	0	3.98206455482162E-94
RPL12	1.92641452	1	1	6.49961562031577E-18
RPS24	1.91921404	1	1	3.40486584673649E-12
HLA-DRA	1.86561341	0.837	0.8	1.0552657746318E-11
S100A4	1.85587078	0.956	0.928	1.91223620345853E-13
SH3BGRL3	1.79990669	0.988	0.989	6.30613233180293E-07
CD74	1.70951598	0.895	0.806	1.25136200871898E-22
CTSS	1.68194642	0.997	0.994	7.34270077087201E-08
RNASET2	1.67245422	0.987	0.974	4.74259925928252E-16
MMP7	1.54855768	0.151	0.1	1.07743080250329E-08
ITGB2	1.53586528	0.991	0.982	3.7521051901304E-16
CD68	1.53464769	0.998	0.996	0.000280833
RPL13	1.48574188	1	1	0.005643515
GRN	1.45708248	0.955	0.944	2.35947286704185E-05

MT-CYB	1.40209286	0.932	0.943	0.005834318
PLD3	1.38792782	0.956	0.932	0.000428907
RPL7A	1.36201407	1	1	3.69246145607032E-08
MFSD12	1.35688888	0.669	0.578	1.69525888387706E-12
FBP1	1.26399667	0.859	0.833	5.8866532890022E-13
HLA-DRB1	1.26140633	0.706	0.562	3.05380214847396E-21
IL1RN	1.22007542	0.687	0.652	6.26509617302792E-08
ARPC1B	1.19874207	0.829	0.753	5.22298580545937E-15
ACTG1	1.18345352	0.98	0.97	0.000165805
FN1	1.17499386	0.261	0.215	3.00252568584013E-05
CAPG	1.13569679	0.781	0.674	2.18070932460538E-16
RPL3	1.12824942	1	1	0.005297726
HIV-A	1.11859649	0.076	0	1.57722765476768E-32
CTSZ	1.0917669	0.908	0.875	0.000220553
RPS8	1.08061354	1	1	8.83900751189145E-07
GSN	0.94530624	0.827	0.767	2.64237784303551E-16
TGFBI	0.90814081	0.686	0.627	6.60171361736484E-05
ANXA2	0.90171593	0.912	0.902	4.49528798152653E-10
CYP27A1	0.88319988	0.918	0.893	1.75588904598502E-10
C1QA	0.87597445	0.429	0.258	1.26697670219142E-24
CYP1B1	0.87414504	0.745	0.678	3.34734062705505E-09
LAMP1	0.86123133	0.8	0.685	5.33583691501886E-18
HIV-D	0.82070175	0.175	0	3.00610205810959E-82
ATP6AP2	0.81343928	0.871	0.817	5.74511721727176E-18
TXN	0.80952995	0.915	0.891	1.75328330364744E-09
HLA-DPA1	0.79387452	0.626	0.48	1.11110664093426E-17
PRDX1	0.78999431	0.865	0.816	5.09427458692934E-09
AHNAK	0.78879751	0.944	0.907	5.634887145529E-10
RPSA	0.7166846	0.992	0.991	7.84878561420627E-06
COX5B	0.7121352	0.947	0.909	9.8980302790757E-15
CFL1	0.69874741	0.821	0.773	3.97837346470806E-05
TXNRD1	0.69343551	0.466	0.359	3.47170311097598E-10
CALM2	0.69132468	0.828	0.775	0.005032769
HIV-C	0.67684211	0.174	0	3.73016611650886E-83
ADAMDEC1	0.66843416	0.698	0.608	0.000461874
RP11-20G13	0.65717085	0.213	0.133	2.33933269975118E-15
CALM3	0.64179496	0.753	0.672	5.73213608405932E-19
GM2A	0.62587129	0.74	0.628	6.01479380208996E-12
HMG20B	0.62569236	0.638	0.559	6.8363140283054E-11
IL4I1	0.60786315	0.627	0.525	1.97549491517378E-09
STAB1	0.60693051	0.461	0.337	5.00173258734789E-09
TMEM176B	0.6045596	0.675	0.544	6.52601132203446E-05
MARCO	0.5947038	0.527	0.479	0.000221664
ARPC5	0.58965418	0.833	0.756	2.18024534266043E-05
HLA-DPB1	0.58831503	0.554	0.41	2.56341583776632E-13
GUSB	0.58518107	0.691	0.568	2.91195455788321E-16
TIMP2	0.57625501	0.935	0.9	4.49191206898825E-10
CCDC88A	0.57264735	0.805	0.714	1.06609573840228E-17
CD81	0.56775799	0.862	0.811	1.05134433076226E-10
LPL	0.5669732	0.242	0.154	9.9530454488868E-16
BCAP31	0.56155843	0.835	0.801	1.74979145973442E-11
TREM2	0.56012965	0.526	0.397	1.68293854980938E-16

IFI6	0.55845013	0.393	0.28	3.02188878414765E-17
GSTO1	0.55530055	0.97	0.972	9.74782630841028E-08
HLA-DMA	0.53095041	0.714	0.575	2.94913330224203E-14
CD4	0.528139	0.672	0.503	5.72530234401081E-29
C9orf16	0.52110945	0.592	0.456	4.00215463805563E-14
PLTP	0.51709606	0.407	0.265	4.01675271070532E-24
CD36	0.51579873	0.687	0.581	0.000950179
PLEK	0.51403383	0.707	0.639	0.000157042
CD84	0.5126953	0.513	0.376	8.99028005955578E-15
A2M	0.50543785	0.537	0.407	2.83982825668342E-16
DBI	0.50254686	0.836	0.77	1.41176338371876E-11
CXCL16	0.48816396	0.897	0.855	1.73928936001902E-09
COX6C	0.48561937	0.787	0.692	9.72495065677728E-10
FAH	0.48179496	0.564	0.46	8.89707294408294E-12
C1QC	0.47769007	0.342	0.203	1.72717420963551E-11
CTSH	0.47210122	0.849	0.775	5.94550470333261E-16
VMO1	0.47124722	0.366	0.262	3.79148188114381E-07
CYB5A	0.4663307	0.591	0.443	3.01510447149397E-26
IQGAP2	0.46306105	0.699	0.562	3.21147515223582E-14
HEXA	0.45613423	0.836	0.747	7.64010499082084E-09
FAM20C	0.43514595	0.553	0.449	1.35182025050634E-12
TNFSF13B	0.43475058	0.593	0.467	3.51898809271789E-07
NDUFS5	0.4312936	0.902	0.859	1.03704667086294E-07
FDX1	0.43102669	0.46	0.345	3.70996993377064E-17
PLEKHO1	0.42841768	0.658	0.549	1.47041925076302E-06
APMAP	0.42549977	0.698	0.567	1.60536912701806E-10
ATP5G3	0.42477259	0.845	0.792	1.03236997380561E-10
SCARB2	0.42417042	0.719	0.59	2.10264007951796E-14
FERMT3	0.41148006	0.683	0.585	3.06866055042292E-07
EEF1B2	0.40653804	0.982	0.986	1.76888638606499E-06
ALCAM	0.40513084	0.442	0.361	5.6018296802576E-09
RPS6	0.40290292	1	1	0.001070679
CD86	0.40210205	0.702	0.578	1.8276529709926E-12
MGST1	0.40201525	0.797	0.771	5.11620641481427E-06
CECR1	0.40002464	0.689	0.586	2.58656472533261E-08
ANXA11	0.39128512	0.775	0.682	9.19318590328564E-12
KIAA0930	0.37740213	0.719	0.639	0.0012258
CCL22	0.37142003	0.067	0.042	6.00738856724274E-05
PYCARD	0.36049638	0.713	0.58	1.72766643813858E-08
ITGAM	0.35742269	0.759	0.668	2.64320210433332E-17
PTPN6	0.35720978	0.621	0.533	0.000107355
CRABP2	0.35677234	0.144	0.093	3.49727738712561E-06
SLC7A7	0.35529427	0.65	0.52	2.42672227474195E-20
MGLL	0.352509	0.393	0.312	0.001701987
CTSC	0.34787351	0.522	0.401	4.97773251447225E-12
NPL	0.34638556	0.64	0.529	1.46774205023894E-05
CIR1	0.34493622	0.619	0.543	0.002230914
TGFB1	0.3427214	0.727	0.662	0.000235504
TM4SF19.1	0.33818825	0.173	0.112	2.99527822154948E-05
MYL12A	0.33253521	0.896	0.855	0.001028125
C3	0.32639317	0.421	0.361	4.67480115477377E-06
BCL2A1	0.32593234	0.519	0.493	4.42397995400518E-06

PGD	0.3252608	0.728	0.643	1.53092377260591E-10
RP11-290F2	0.32382363	0.636	0.54	0.001336782
PRKAR1A	0.32348421	0.779	0.72	1.12503595772318E-08
ASAP1	0.32246336	0.693	0.622	8.66033700427632E-13
MATK	0.31931245	0.401	0.322	3.68097235672835E-08
CYSTM1	0.31582565	0.543	0.452	2.85116540999432E-09
TMEM91	0.31184538	0.77	0.689	6.29895159059177E-09
PPT1	0.31008864	0.499	0.39	8.15278677143277E-11
SLC25A5	0.30856326	0.56	0.475	4.73365327622889E-09
RHOC	0.30805197	0.348	0.238	4.23841191708565E-11
C1orf54	0.30301028	0.44	0.306	9.39820706564624E-18
MS4A4A	0.30257731	0.496	0.364	1.95518419241863E-14
DNAJC5B	0.29877158	0.298	0.192	1.66171910675391E-12
ATP6V1D	0.29668656	0.429	0.345	7.01751951840412E-06
HLA-DMB	0.29391196	0.385	0.267	9.37206826773246E-09
G6PD	0.29350026	0.336	0.254	9.03343901176162E-06
FKBP1A	0.29285399	0.518	0.419	1.32755999078363E-05
SCPEP1	0.2836126	0.552	0.446	1.91731694676393E-08
MNDA	0.28092691	0.399	0.313	2.36624465395901E-07
C1QB	0.28041554	0.138	0.061	5.13712982459078E-08
CD59	0.27968279	0.373	0.269	1.18937984199839E-13
UCP2	0.27456129	0.361	0.256	5.96701921329582E-06
ITGB1BP1	0.27269883	0.396	0.293	2.6647478128876E-13
MSR1	0.27198005	0.333	0.205	2.9005432498938E-13
EMP1	0.27189113	0.424	0.345	7.64470833732777E-05
DAD1	0.26971548	0.771	0.713	0.000519773
NDUFB2	0.26252858	0.559	0.469	0.004146968
IL2RG	0.2623476	0.519	0.403	2.19199653310935E-08
HAVCR2	0.26208384	0.477	0.353	3.41831167540038E-13
GLA	0.25757282	0.435	0.332	1.20164653136836E-05
HLA-DQB1	0.25564421	0.375	0.263	7.02432747675259E-05
GCLC	0.25418231	0.242	0.158	1.73235905284338E-10
LGALS9	0.25175796	0.531	0.419	5.4831336745031E-11
GPRIN3	0.24992964	0.493	0.374	2.03522280485921E-09
RAB31	0.24843863	0.492	0.379	7.19261554689887E-08
HM13	0.24816702	0.571	0.484	7.42032669374114E-05
C4orf48	0.247584	0.419	0.31	8.66010699808101E-11
SQRDL	0.24552567	0.665	0.579	1.68520909149794E-10
PRKCB	0.24400578	0.467	0.356	1.42225922223654E-10
COX7B	0.24332764	0.782	0.746	0.002425224
TUBA1A	0.24301891	0.384	0.286	0.000100066
PTGR1	0.24258932	0.33	0.233	4.05599246464196E-05
MSN	0.24038141	0.59	0.487	0.002910971
SAMSN1	0.24001303	0.494	0.4	1.43021712132876E-06
RAC2	0.23935577	0.839	0.825	1.06640431946755E-05
YWHAQ	0.23580619	0.565	0.49	8.3593393797352E-10
CHCHD10	0.23433927	0.759	0.698	0.000163045
MYOF	0.23387593	0.435	0.345	1.92383633514496E-07
CYB561A3	0.23309719	0.366	0.252	7.88301130891952E-05
SEMA3C	0.23063014	0.416	0.31	3.46570931974319E-07
OAS1	0.22758188	0.256	0.151	5.10248445099398E-10
NCEH1	0.2269674	0.392	0.29	8.60606046960138E-07

MPG	0.20853987	0.533	0.429	4.59545594385444E-06
NRP2	0.20162686	0.449	0.349	7.51560101670415E-07
IL18	0.19754559	0.459	0.333	6.87425904401937E-12
C20orf24	0.19581051	0.388	0.309	9.00909944694302E-05
GLIPR2	0.19479986	0.445	0.347	9.59957149396457E-05
CHST11	0.19289888	0.596	0.506	0.000115284
TNS3	0.1907448	0.456	0.374	0.000439948
ITGB7	0.1898966	0.3	0.204	2.66081500923694E-08
HLA-DQA1	0.18677858	0.213	0.11	1.75587160750391E-10
IL1R2	0.18573717	0.092	0.044	2.48118799720161E-06
MT-ND6	0.18405953	0.347	0.252	6.87604487102819E-09
SCD	0.18197648	0.414	0.336	0.001128042
SPARC	0.18107421	0.178	0.113	1.6356596761253E-06
PHLDA3	0.17788971	0.248	0.153	1.61677506678712E-10
LDHB	0.17721205	0.403	0.294	1.48094626957708E-09
GALM	0.17487441	0.325	0.224	1.5834978387459E-09
MRAS	0.17250178	0.301	0.201	5.98133659733292E-08
SERPINE1	0.16981977	0.416	0.349	0.000436826
LGMN	0.16973149	0.327	0.225	0.000152451
ATP6V0D2	0.16827657	0.328	0.229	2.32262163557302E-05
GPC4	0.16677018	0.173	0.124	4.40199023286389E-06
CHD4	0.16611606	0.36	0.266	0.000948807
CD300LB	0.16503023	0.295	0.205	0.000348711
ABHD2	0.16427904	0.429	0.343	0.005998062
FRMD4B	0.15935342	0.257	0.164	2.42419247884932E-05
ST14	0.15716748	0.392	0.302	0.005720561
ITGB5	0.15544276	0.233	0.135	4.26489410776887E-08
CD97	0.15405086	0.377	0.293	5.49951043893544E-05
METTL7A	0.15390937	0.251	0.149	8.62725862506868E-09
DCXR	0.1535756	0.365	0.281	0.009918355
AMFR	0.15314844	0.436	0.352	0.00224651
EMILIN1	0.15172727	0.173	0.11	0.000228089
RRBP1	0.14977127	0.263	0.182	0.000945342
LAIR1	0.14812778	0.317	0.215	6.91540475273607E-06
NR1H3	0.14742995	0.221	0.135	4.50538126709038E-08
SLC12A8	0.1464051	0.188	0.114	1.36884113397491E-05
HK3	0.14607211	0.245	0.167	8.41350890892295E-08
MTSS1	0.14589632	0.42	0.327	0.001798289
RGS1	0.14474724	0.468	0.425	0.000395151
MYO1E	0.14266427	0.25	0.181	0.000126896
CAMK1	0.14194564	0.348	0.251	0.000128231
FABP3	0.1412391	0.173	0.088	9.4223716157189E-13
RP11-680A1	0.14006584	0.238	0.154	5.98284968811168E-09
HN1	0.13950041	0.266	0.183	0.002344803
RAMP1	0.1390508	0.163	0.091	0.001151691
SERINC2	0.13202078	0.241	0.152	8.81099777712972E-05
SRD5A3	0.13197354	0.255	0.164	6.09100955445243E-10
EPB41L2	0.12536612	0.197	0.117	7.25440506791195E-07
HMGA1	0.12231661	0.58	0.523	0.006674659
COL6A1	0.12053122	0.081	0.043	0.001940983
UBE2E3	-0.0444246	0.2	0.216	5.93625392577912E-06
CDCP1	-0.0456135	0.077	0.097	9.28887385396896E-05

EFNA5	-0.046297	0.138	0.16	0.001866052
H1F0	-0.0479353	0.353	0.355	1.47908364006907E-07
WDR74	-0.0576325	0.29	0.298	2.68289034905878E-09
AKR1B1	-0.0592565	0.346	0.347	0.001233549
HAUS6	-0.0595456	0.193	0.204	6.41350272081568E-09
NKG7	-0.0615751	0.04	0.04	0.00794804
HIST1H2BK	-0.0624545	0.247	0.25	0.00075872
NUPL1	-0.0648974	0.446	0.441	6.01921819531557E-05
SEMA4D	-0.0654029	0.192	0.223	1.69409000694273E-15
STK17B	-0.0797297	0.512	0.52	0.003277337
MXD1	-0.0816153	0.399	0.423	0.000558631
DENND5A	-0.0823857	0.432	0.445	5.17016305894487E-05
IRS2	-0.0872615	0.374	0.389	0.00135465
MARCKSL1	-0.0880192	0.266	0.294	2.53060225789876E-09
ITPR1	-0.0918514	0.381	0.384	1.5265434331112E-08
KLRB1	-0.0933496	0.009	0.014	3.41849810674847E-05
EVI2A	-0.0946639	0.758	0.786	2.32200038308081E-05
KLF10	-0.1005122	0.585	0.613	2.15622293972048E-10
YPEL3	-0.1022446	0.782	0.815	0.007179463
GNLY	-0.1032693	0.005	0.012	1.76952529214245E-09
H1FX	-0.1034143	0.675	0.681	1.36748555636489E-06
FCAR	-0.107519	0.285	0.32	1.74224788669256E-06
RYBP	-0.108751	0.433	0.45	1.59295556535117E-11
CHML	-0.1113931	0.247	0.272	0.002763882
SLC38A2	-0.1114619	0.697	0.713	0.003891615
RNMT	-0.1123166	0.678	0.687	0.000274047
PIK3R5	-0.115438	0.373	0.403	2.46011329217874E-10
SNORD3B-2	-0.1171475	0.175	0.206	1.77954489859626E-05
TXNIP	-0.1199761	0.982	0.983	1.94970269560917E-20
EPB41L4A-A	-0.1201454	0.57	0.617	0.000456164
MT-ND2	-0.1253205	0.983	0.986	8.72075262227085E-05
HNRNPH1	-0.125656	0.75	0.772	0.007378643
CSRNP1	-0.1275962	0.261	0.311	0.00109291
P4HA1	-0.1321424	0.957	0.956	5.29384962458978E-06
BTG2	-0.1333077	0.63	0.665	0.000352564
VAMP2	-0.1344888	0.521	0.556	1.07007932766391E-08
HIST1H1C	-0.1355367	0.445	0.459	5.25979988033401E-05
SLC3A2	-0.1415489	0.751	0.768	1.42628064940693E-06
RP11-1143G	-0.1476184	0.656	0.675	5.20341212775805E-06
ZNF395	-0.1566768	0.676	0.713	6.22207090227875E-08
SRSF7	-0.1746026	0.684	0.71	2.11960584464041E-05
SNAPC1	-0.1809266	0.36	0.39	6.25409416501065E-12
FUS	-0.2076735	0.767	0.792	5.87313174090968E-09
GPI	-0.2158421	0.829	0.837	2.74853650471564E-08
GPCPD1	-0.2202263	0.639	0.691	1.604063789388E-07
CLK1	-0.2233413	0.581	0.633	0.00084034
BRD2	-0.2260702	0.661	0.705	0.001191429
MAP3K2	-0.2359323	0.791	0.821	5.76618089060295E-05
CREBRF	-0.2369013	0.655	0.697	2.48624415481678E-07
DDIT4	-0.2412791	0.61	0.647	5.20491888232014E-09
ARRDC3	-0.2458836	0.836	0.877	7.61264012948357E-06
CITED2	-0.250387	0.514	0.542	0.009280617

IFNGR2	-0.2677052	0.821	0.864	0.000123245
ALOX5AP	-0.3157613	0.681	0.758	0.000846579
MXI1	-0.3466479	0.8	0.837	5.61572535817819E-14
RPS27	-0.3664257	1	1	6.35942734492334E-11
BZW1	-0.3877095	0.828	0.854	3.96006338534402E-07
FOSL2	-0.3881581	0.719	0.786	2.708985905017E-07
DUSP6	-0.4068133	0.649	0.708	0.000133589
OPN3	-0.4311403	0.433	0.523	8.01546233209178E-28
SLC11A1	-0.5529422	0.824	0.865	3.22429824175406E-05
CTB-61M7.2	-0.5966513	0.486	0.585	3.98254213783839E-23
PNRC1	-0.6615171	0.958	0.967	5.23718215847277E-10
VEGFA	-0.6795621	0.804	0.858	4.1367174186637E-05
HILPDA	-0.6826048	0.755	0.784	1.90572337165755E-06
CXCR4	-0.7062109	0.798	0.851	4.71074006340168E-17
IER2	-0.7291065	0.779	0.838	5.9145836836879E-14
FCGR2B	-0.7641098	0.777	0.845	4.20468796360072E-09
JUNB	-0.8203021	0.968	0.973	1.92624781711041E-11
EGR1	-1.0125078	0.753	0.826	7.58623170916667E-09
SLC2A3	-1.026361	0.933	0.95	0.001325993
NDRG1	-1.0488384	0.952	0.969	5.48838349865104E-16
JUND	-1.3043117	0.966	0.98	5.40404856610453E-22
GLUL	-1.7898066	0.991	0.996	3.85667338477518E-19
JUN	-1.8417747	0.94	0.966	0.007828701
H3F3B	-1.9931806	0.997	0.998	3.03728987727315E-14
DUSP1	-2.3531496	0.942	0.968	1.93129426265017E-10
ZFP36L1	-2.3751693	0.985	0.988	2.10075663970255E-22
FOS	-3.1182203	0.955	0.975	1.39238073348505E-07
NFKBIA	-3.6772163	0.838	0.907	1.32058592142897E-07
BTG1	-3.9523298	0.979	0.986	5.34349163833493E-22
PLIN2	-5.8744952	0.998	0.998	4.89202389614637E-43
MT1X	-6.3815528	0.495	0.635	2.02316151841871E-11
MT2A	-7.1116495	0.709	0.795	8.90930803611087E-07
LYZ	-12.542648	0.999	0.999	3.20003137593781E-08
MALAT1	-14.980666	1	1	0.000187218
IL8	-16.412568	0.887	0.938	4.39617278382751E-19

Footnotes:

1- Value refers to average differential expression within one subset of scaled pearsons residuals

2- Percentage of cells, within the cluster ID for which the gene is a marker, that detect the gene

3- Percentage of all the other cells, excluding the cluster ID for which the gene is a marker, that detect the gene
