# Supplementary material for: Interactions of Monocytes, HIV, and ART Identified by an Innovative scRNAseq Pipeline: Pathways to Reservoirs and HIV-Associated Comorbidities
Source: mBio. 2020 Jul 28;11(4):e01037-20. doi: 10.1128/mBio.01037-20 (PMC7387797; doi:10.1128/mBio.01037-20)
Supplement: TABLE S2 [file mBio.01037-20-st002.pdf]

Supplementary Table 2: List of differentially expressed genes between HIV^{+} and HIV^{exp} in every cluster from the integrated dataset of HIV-infected mature monocytes without ART-treatment (Cluster X HIV^{+} vs Cluster X HIV^{exp})

Gene.ID	Avg. Diff.[1]	Pct.1[2]	Pct.2[3]	P.Value.Adj	Cluster.ID	Comments
TMSB4X	11.1144285	1	0.999	1.15E-14	0	
CTSB	7.049561559	0.988	0.991	8.45E-05	0	
B2M	6.184487622	1	1	2.34E-07	0	
MT-CO2	4.614835097	0.974	0.978	4.22E-12	0	
APOE	3.13683342	0.798	0.745	2.63E-06	0	
HIV-B	2.720394737	0.617	0	1.71E-68	0	Shared with clusters 0, 1, 2, 3, 4, 5, and 6
CST3	2.388406298	0.933	0.856	3.09E-10	0	
CTSS	2.354135649	0.99	0.989	0.003862474	0	
RPS12	2.258222392	1	1	0.004373775	0	
MT-CO3	1.876522474	0.992	0.991	0.000250267	0	
ARL4C	1.796760879	0.724	0.508	4.61E-05	0	
TGFBI	1.701596969	0.735	0.528	5.02E-05	0	
CD74	1.679335767	0.768	0.62	0.003431825	0	
HIV-E	1.417763158	0.215	0	1.42E-21	0	Shared with clusters 0, 1, 2, 3, and 4
STAB1	0.943294705	0.479	0.249	7.56E-05	0	
RPSA	0.875490345	0.993	0.989	0.006221981	0	
CXCL16	0.835601018	0.928	0.834	9.53E-05	0	
HIV-A	0.675986842	0.072	0	2.71E-05	0	Shared with clusters 0, 1, and 3
LAMP1	0.619859519	0.709	0.498	0.007567526	0	
HIV-D	0.550986842	0.169	0	6.12E-17	0	Shared with clusters 0, 1, 2, 3, 4, and 6
CD4	0.539252938	0.551	0.356	0.003137077	0	Shared with clusters 0 and 3
HIV-C	0.529605263	0.186	0	1.47E-19	0	Shared with clusters 0, 1, 2, 3, 4, and 6
DOCK4	0.439336015	0.562	0.398	0.006655337	0	
MT-ND5	0.358303453	0.891	0.917	0.000507561	0	
CYB5A	0.300106961	0.414	0.248	0.000421329	0	
MT-ND4	-0.238983649	0.995	0.996	0.001462902	0	
CD9	-0.54575615	0.969	0.973	0.000682287	0	Shared with clusters 0 and 4
ANXA5	-0.576140485	0.9	0.922	0.00019041	0	
OPN3	-0.661790098	0.526	0.625	2.19E-06	0	
BZW1	-0.681177986	0.836	0.886	0.001793812	0	Shared with clusters 0 and 7
RGCC	-1.519501363	0.724	0.826	2.64E-07	0	
PLIN2	-7.27084755	1	1	1.59E-09	0	
HIV-B	2.10663199	0.628	0	2.08E-78	1	Shared with clusters 0, 1, 2, 3, 4, 5, and 6
C1QA	1.005827456	0.586	0.431	0.003076753	1	
HIV-E	0.77503251	0.218	0	1.45E-26	1	Shared with clusters 0, 1, 2, 3, and 4
HIV-A	0.486345904	0.079	0	1.67E-07	1	Shared with clusters 0, 1, and 3
HIV-D	0.461638492	0.183	0	1.64E-22	1	Shared with clusters 0, 1, 2, 3, 4, and 6
HIV-C	0.317295189	0.168	0	2.86E-20	1	Shared with clusters 0, 1, 2, 3, 4, and 6
JUNB	-1.012443099	0.995	0.992	2.80E-06	1	
NFKBIA	-3.893012207	0.87	0.92	0.001695477	1	
IL8	-12.67445183	0.935	0.962	0.00031458	1	
HIV-B	8.45014245	0.567	0	1.22E-37	2	Shared with clusters 0, 1, 2, 3, 4, 5, and 6
HIV-E	3.96011396	0.202	0	2.79E-11	2	Shared with clusters 0, 1, 2, 3, and 4
HIV-D	1.715099715	0.168	0	8.99E-09	2	Shared with clusters 0, 1, 2, 3, 4, and 6
HIV-C	1.393162393	0.174	0	1.43E-09	2	Shared with clusters 0, 1, 2, 3, 4, and 6
FAM101B	-0.127126622	0.071	0.165	7.17E-05	2	Shared with clusters 2 and 7
HIV-B	4.781395349	0.451	0	3.84E-40	3	Shared with clusters 0, 1, 2, 3, 4, 5, and 6
LGALS1	3.5088376	0.997	0.998	0.000495701	3	
HIV-E	2.724031008	0.175	0	2.58E-14	3	Shared with clusters 0, 1, 2, 3, and 4
RPS8	1.774520141	1	0.999	0.000416713	3	
HIV-A	1.421705426	0.085	0	7.29E-07	3	Shared with clusters 0, 1, and 3
HIV-D	1.029457364	0.158	0	2.46E-14	3	Shared with clusters 0, 1, 2, 3, 4, and 6
HIV-C	0.849612403	0.16	0	2.86E-14	3	Shared with clusters 0, 1, 2, 3, 4, and 6
PLTP	0.832233027	0.668	0.532	9.06E-07	3	
CD4	0.366900494	0.611	0.473	1.39E-05	3	Shared with clusters 0 and 3
FCGR2A	-0.212590065	0.398	0.423	0.008505055	3	
PLD3	-0.852577471	0.991	0.994	2.81E-05	3	

CTSD	-2.585266924	0.998	0.999	7.04E-06	3	
HIV-B	1.5	0.612	0	2.45E-20	4	Shared with clusters 0, 1, 2, 3, 4, 5, and 6
HIV-E	0.49375	0.244	0	2.18E-06	4	Shared with clusters 0, 1, 2, 3, and 4
HIV-D	0.36875	0.175	0	0.001269966	4	Shared with clusters 0, 1, 2, 3, 4, and 6
HIV-C	0.31875	0.175	0	0.001114827	4	Shared with clusters 0, 1, 2, 3, 4, and 6
LGALS2	0.29748193	0.625	0.379	3.88E-05	4	
C16orf98	-0.04336895	0	0.03	2.39E-06	4	
GNB5	-0.057196732	0	0.029	6.39E-08	4	
PM20D2	-0.095014928	0.012	0.055	0.002003789	4	
MME	-0.153680861	0.038	0.089	0.002734286	4	
TUBA4A	-0.284785512	0.188	0.262	0.008212208	4	
CD63	-1.245266342	0.988	1	0.006273682	4	
CD9	-1.583764142	0.988	0.994	0.001909262	4	Shared with clusters 0 and 4
SDC2	-2.111348994	0.981	0.992	4.12E-05	4	
HILPDA	-2.31105044	0.956	0.97	0.002130011	4	
SRGN	-4.019374607	0.994	1	0.002098447	4	
RPL30	-4.592791483	1	1	0.002909382	4	
MT2A	-7.044512099	0.994	0.98	0.003708604	4	
HIV-B	9.952941176	0.694	0	5.40E-07	5	Shared with clusters 0, 1, 2, 3, 4, 5, and 6
FCGR1A	-0.045304778	0	0.033	4.06E-05	5	
TJP2	-0.046952224	0	0.038	2.48E-06	5	
EBLN2	-0.049423394	0	0.036	2.25E-11	5	
THBD	-0.076141099	0.024	0.044	4.30E-05	5	
ARHGAP26	-0.100281035	0.082	0.128	0.003908121	5	
SLC25A37	-0.119604613	0.165	0.157	0.003139514	5	
HIV-B	4.617486339	0.705	0	5.68E-26	6	Shared with clusters 0, 1, 2, 3, 4, 5, and 6
HIV-C	0.699453552	0.208	0	4.64E-05	6	Shared with clusters 0, 1, 2, 3, 4, and 6
HIV-D	0.590163934	0.213	0	3.68E-05	6	Shared with clusters 0, 1, 2, 3, 4, and 6
LTA	-0.046956522	0	0.038	0.008554923	7	
HAPLN3	-0.048695652	0	0.042	0.002535767	7	
ZNF445	-0.052173913	0	0.037	0.004260847	7	
LRIG1	-0.053913043	0	0.045	0.000918545	7	
RP11-284N8.3	-0.055652174	0	0.049	0.00026238	7	
LMOD3	-0.057391304	0	0.04	0.007758803	7	
CD200R1	-0.064347826	0	0.05	0.000138572	7	
EPB41L2	-0.066086957	0	0.038	0.006524215	7	
SIDT1	-0.066086957	0	0.049	0.000326458	7	
UBE2O	-0.066086957	0	0.042	0.002050051	7	
KLHL15	-0.067826087	0	0.04	0.006582756	7	
MPP6	-0.069565217	0	0.042	0.004412425	7	
ATP8A1	-0.073043478	0	0.043	0.003008923	7	
MAD2L1	-0.073043478	0	0.047	0.001691633	7	
AC132872.2	-0.076521739	0	0.045	0.002291322	7	
FAM84B	-0.076521739	0	0.056	3.50E-05	7	
LAG3	-0.07826087	0	0.045	0.00120386	7	
OTUD7B	-0.083478261	0	0.049	0.000871689	7	
METTL2B	-0.085217391	0	0.052	0.007220328	7	
TSPYL4	-0.088695652	0	0.054	0.000410937	7	
HSPA1B	-0.093913043	0	0.052	0.003911148	7	
FEZ1	-0.097391304	0	0.052	0.000108894	7	
HIC1	-0.099130435	0	0.056	3.76E-05	7	
KIAA0922	-0.102608696	0	0.059	1.35E-05	7	
RPS6KA5	-0.106086957	0	0.059	4.07E-05	7	
EOMES	-0.107826087	0	0.056	3.40E-05	7	
POLG2	-0.107826087	0	0.059	2.29E-05	7	
GSAP	-0.113043478	0	0.057	0.000171158	7	
SLFN11	-0.11826087	0	0.064	1.57E-06	7	
TMEM138	-0.123478261	0	0.064	6.27E-08	7	
DDI2	-0.132173913	0	0.068	0.006631303	7	
CD8B	-0.140869565	0	0.07	5.47E-07	7	
VPS13D	-0.142608696	0	0.083	2.64E-08	7	

RP11-356I2.4	-0.146086957	0	0.078	2.78E-07	7	
CEP78	-0.15826087	0	0.08	4.63E-07	7	
FAM101B	-0.166956522	0	0.083	0.000696751	7	Shared with clusters 2 and 7
TET2	-0.170434783	0	0.083	7.43E-05	7	
MTMR4	-0.172173913	0	0.099	2.38E-09	7	
ITGA5	-0.175652174	0	0.085	0.000327337	7	
TNRC6C	-0.253175231	0.03	0.155	0.000845618	7	
GLIPR2	-0.265349144	0.03	0.146	6.06E-07	7	
UBE2E3	-0.269565217	0	0.144	2.34E-15	7	
CTSW	-0.365480896	0.03	0.193	0.006837956	7	
BZW1	-0.520737813	0.909	0.906	0.000361489	7	Shared with clusters 0 and 7
TSHZ3	-0.139240506	0	0.139	8.30E-05	8	

Footnotes:

1- Value refers to average differential expression within one subset of scaled pearsons residuals. Positive values represent increased gene expression in HIV+ cells from that cluster. Negative values represent increased gene expression in HIVexp cells from that cluster.

2- Percentage of cells, within the cluster ID for which the gene is a marker, that detect the gene

3- Percentage of all the other cells, excluding the cluster ID for which the gene is a marker, that detect the gene
