# Supplementary material for: Interactions of Monocytes, HIV, and ART Identified by an Innovative scRNAseq Pipeline: Pathways to Reservoirs and HIV-Associated Comorbidities
Source: mBio. 2020 Jul 28;11(4):e01037-20. doi: 10.1128/mBio.01037-20 (PMC7387797; doi:10.1128/mBio.01037-20)
Supplement: TABLE S3 [file mBio.01037-20-st003.pdf]

Supplementary Table 3: List of differentially expressed genes modulated by ART in HIV$^+$ mature monocytes from the integrated dataset of HIV-infected mature monocytes with and without ART-treatment (HIV$^+$ with ART-treatment vs HIV$^+$ without ART-treatment).

Gene.ID	Avg. Diff.[1]	Pct.1[2]	Pct.2[3]	P.Value.Adj
CYP1B1	-3.701367211	0.138	0.745	4.40E-07
MARCO	-2.331578947	0	0.527	4.68E-10
GLRX	-1.159806413	0.069	0.626	0.000369143
MSN	-1.089376891	0.034	0.589	0.003408079
PTAFR	-0.885517241	0.034	0.532	0.001713199
CEP350	-0.801403509	0	0.51	3.14E-18
SLC6A8	-0.641052632	0	0.187	4.87E-06
CYCS	-0.637894737	0	0.328	3.38E-27
CERK	-0.634736842	0	0.426	1.74E-13
SERTAD2	-0.601403509	0	0.419	2.19E-30
NF1	-0.586315789	0	0.391	1.25E-19
NFIL3	-0.570526316	0	0.389	2.71E-12
FAM118A	-0.555789474	0	0.332	0.000255318
MSR1	-0.527332123	0.138	0.334	0.009137933
TRIP11	-0.523859649	0	0.36	1.06E-18
KPNA4	-0.492631579	0	0.366	6.81E-28
HMGXB4	-0.48245614	0	0.377	1.87E-21
PRPF6	-0.474736842	0	0.36	4.49E-05
USP7	-0.46877193	0	0.358	1.49E-19
SMEK2	-0.458947368	0	0.356	1.09E-23
KIAA1147	-0.458245614	0	0.29	5.38E-09
EPRS	-0.457192982	0	0.341	1.95E-18
SLK	-0.430175439	0	0.334	7.86E-18
ARRDC4	-0.423762855	0.034	0.336	0.009569416
ATXN7	-0.407719298	0	0.293	6.72E-18
PRAM1	-0.402105263	0	0.306	7.65E-17
PIK3R1	-0.402105263	0	0.295	8.78E-15
ITPRIPL2	-0.401403509	0	0.284	9.92E-13
BCAS2	-0.401403509	0	0.329	0.004844279
SETD2	-0.392631579	0	0.299	3.28E-10
TRIM8	-0.384912281	0	0.311	1.83E-16
POGZ	-0.377192982	0	0.285	5.28E-15
KLF3	-0.373684211	0	0.283	6.92E-22
SIK3	-0.366666667	0	0.296	3.41E-12
SLFN5	-0.363508772	0	0.266	5.26E-16
SCG5	-0.360701754	0	0.086	2.19E-08
MOB1B	-0.358947368	0	0.283	3.12E-17
RLIM	-0.352982456	0	0.286	1.38E-21
CSNK1G2	-0.350526316	0	0.283	3.13E-08
LILRB2	-0.348421053	0	0.247	3.09E-09
GSR	-0.347719298	0	0.255	1.81E-09
DAPK1	-0.345263158	0	0.279	5.41E-12
FLNB	-0.343508772	0	0.265	4.48E-08
NIN	-0.339298246	0	0.257	1.19E-18
HDGFRP2	-0.33754386	0	0.279	1.03E-17

LPAR2	-0.33754386	0	0.268	2.40E-05
BAZ1B	-0.337192982	0	0.267	2.85E-17
DNAJB1	-0.333938294	0.034	0.275	3.51E-08
AFMID	-0.332982456	0	0.265	4.40E-23
SPATA13	-0.327368421	0	0.242	3.59E-16
KIAA1598	-0.326315789	0	0.245	1.38E-21
ZNF37A	-0.326315789	0	0.241	5.91E-09
SHPRH	-0.323859649	0	0.242	2.62E-15
LRCH3	-0.320350877	0	0.251	1.37E-15
ABCE1	-0.315789474	0	0.249	7.44E-16
TRAPPC11	-0.315438596	0	0.253	3.90E-17
ZMYM6	-0.315087719	0	0.247	1.51E-14
ODC1	-0.315087719	0	0.229	8.88E-09
CEP152	-0.313333333	0	0.245	1.60E-19
PDCD7	-0.311929825	0	0.249	3.04E-13
DENND1B	-0.307719298	0	0.235	2.58E-13
MTURN	-0.306666667	0	0.242	9.96E-16
APAF1	-0.306315789	0	0.247	1.08E-19
EBP	-0.304561404	0	0.225	4.93E-25
PPP2R5E	-0.303859649	0	0.245	1.19E-18
RASSF5	-0.303157895	0	0.247	5.90E-10
PIK3CA	-0.301403509	0	0.247	1.95E-16
P4HTM	-0.298245614	0	0.253	3.59E-20
STAG1	-0.296140351	0	0.235	1.23E-18
SIPA1L1	-0.292631579	0	0.21	7.79E-11
LRRK2	-0.292631579	0	0.208	0.005878492
EPB41L2	-0.290877193	0	0.197	1.30E-15
PLXNC1	-0.287017544	0	0.218	2.79E-23
CYHR1	-0.287017544	0	0.239	5.62E-12
SAMD9	-0.284561404	0	0.218	1.40E-18
SH3TC1	-0.284561404	0	0.24	1.22E-13
CLPTM1L	-0.283859649	0	0.245	3.23E-18
CLCN7	-0.283157895	0	0.228	1.17E-07
PPID	-0.278947368	0	0.241	2.17E-20
AHCTF1	-0.278947368	0	0.229	7.79E-09
PFKFB4	-0.278947368	0	0.219	9.21E-09
PITPNB	-0.277894737	0	0.228	2.07E-23
RABGAP1	-0.276842105	0	0.234	1.19E-14
ACSL3	-0.276491228	0	0.219	8.70E-16
ATP11A	-0.275087719	0	0.233	5.46E-09
CCND2	-0.270877193	0	0.179	3.56E-13
NSF	-0.270526316	0	0.227	3.66E-23
TGM2	-0.268070175	0	0.144	1.79E-10
FAM76B	-0.267017544	0	0.224	3.20E-26
VOPP1	-0.267017544	0	0.225	2.88E-25
INO80D	-0.265614035	0	0.217	1.55E-14
MCM3AP	-0.264912281	0	0.223	1.60E-14
USF1	-0.264561404	0	0.224	2.78E-11
ING1	-0.263859649	0	0.221	1.98E-19
PRKDC	-0.263508772	0	0.207	2.28E-11
DIAPH1	-0.258596491	0	0.208	6.92E-07
DSTYK	-0.256842105	0	0.211	7.55E-15

ZMYM4	-0.256491228	0	0.209	1.98E-10
CTCF	-0.256491228	0	0.216	9.03E-08
AGPAT3	-0.255789474	0	0.218	1.83E-16
DIDO1	-0.253333333	0	0.213	2.17E-11
QSER1	-0.252982456	0	0.201	0.000208274
FBXW11	-0.251929825	0	0.222	4.55E-22
CLEC12A	-0.251929825	0	0.171	2.42E-08
UBE2S	-0.250877193	0	0.172	0.005695106
PACS1	-0.250526316	0	0.216	1.38E-15
NRIP1	-0.250526316	0	0.209	1.79E-11
MAP3K7	-0.248070175	0	0.207	1.04E-18
GSE1	-0.247017544	0	0.206	1.05E-19
LZTS2	-0.245964912	0	0.213	1.50E-10
MTMR6	-0.245614035	0	0.21	1.05E-19
TP53BP2	-0.245614035	0	0.21	2.52E-14
MAN2A2	-0.245614035	0	0.207	1.75E-12
CTR9	-0.244912281	0	0.208	1.74E-23
TMEM87B	-0.243508772	0	0.206	1.12E-08
HDAC4	-0.241052632	0	0.203	0.002681373
UBE2E3	-0.240701754	0	0.2	6.79E-16
FBXO33	-0.240350877	0	0.202	7.17E-13
KDM1A	-0.239298246	0	0.208	2.49E-16
D2HGDH	-0.236140351	0	0.193	6.57E-30
NPIPB5	-0.234035088	0	0.194	1.34E-08
DOPEY2	-0.232280702	0	0.185	7.68E-14
XPO6	-0.230877193	0	0.2	9.42E-12
PLEKHM1	-0.230526316	0	0.195	1.57E-16
RP11-169K1	-0.229824561	0	0.184	4.36E-10
RUFY2	-0.229473684	0	0.192	3.00E-16
COG4	-0.22877193	0	0.202	1.68E-18
TNFRSF10B	-0.228070175	0	0.196	1.58E-08
ALOX15B	-0.227368421	0	0.173	4.08E-16
MGA	-0.227017544	0	0.188	1.71E-08
BOLA3	-0.225263158	0	0.159	1.25E-19
IFIH1	-0.224912281	0	0.187	4.15E-10
MRPS31	-0.224561404	0	0.187	4.72E-08
ACVR2B	-0.224561404	0	0.186	0.000602084
RNF216	-0.224210526	0	0.191	1.39E-12
WBSCR22	-0.223859649	0	0.199	1.05E-17
ZNF770	-0.223859649	0	0.189	1.06E-08
FMR1	-0.22245614	0	0.198	1.45E-14
ZMAT1	-0.22245614	0	0.166	8.09E-10
WDSUB1	-0.22	0	0.196	1.24E-21
FAM73A	-0.218947368	0	0.188	2.04E-18
ARRDC1	-0.218947368	0	0.193	5.24E-13
IGF1R	-0.218596491	0	0.186	0.000190353
SLC41A2	-0.218245614	0	0.174	1.64E-25
DOCK11	-0.217894737	0	0.187	6.13E-21
FCHSD2	-0.217894737	0	0.187	4.13E-14
LINC-PINT	-0.217192982	0	0.178	4.42E-28
ZNF350	-0.216842105	0	0.195	2.65E-27
DGKH	-0.215438596	0	0.161	3.33E-10

RPP14	-0.214736842	0	0.184	3.43E-12
MADD	-0.213684211	0	0.182	6.07E-12
RPS6KB1	-0.213684211	0	0.185	4.13E-07
CCDC71L	-0.211929825	0	0.18	0.001171381
GOLGA8A	-0.210175439	0	0.167	0.000239349
SMARCA4	-0.208421053	0	0.173	1.75E-16
ERO1LB	-0.207719298	0	0.171	6.93E-18
WDR91	-0.207719298	0	0.18	1.06E-10
SCAPER	-0.207368421	0	0.175	2.41E-07
RMND5A	-0.206666667	0	0.184	1.15E-26
MARK2	-0.205614035	0	0.187	4.11E-09
RPRD1B	-0.205263158	0	0.182	4.82E-19
ATAD2	-0.203508772	0	0.162	1.76E-08
UBE2G2	-0.202807018	0	0.168	9.36E-07
DHX38	-0.20245614	0	0.176	1.45E-12
IGF2BP2	-0.202105263	0	0.172	2.49E-12
DHX32	-0.202105263	0	0.175	9.45E-12
ZNF141	-0.202105263	0	0.17	5.73E-05
ZNF226	-0.201052632	0	0.178	6.91E-21
NCOA2	-0.201052632	0	0.169	1.44E-09
SLC7A8	-0.200701754	0	0.148	4.09E-10
MEF2C	-0.198947368	0	0.155	1.82E-09
GCNT2	-0.198245614	0	0.166	3.94E-17
RBM10	-0.197192982	0	0.172	8.40E-14
RTN4RL2	-0.197192982	0	0.133	4.87E-06
TK1	-0.196842105	0	0.153	1.18E-12
ZNF219	-0.196842105	0	0.176	2.12E-06
CRTC3	-0.196491228	0	0.171	3.57E-12
YARS	-0.196140351	0	0.171	3.12E-21
UBE3C	-0.195789474	0	0.171	6.33E-10
CHSY1	-0.195438596	0	0.164	2.69E-07
NBAS	-0.195438596	0	0.169	6.89E-07
EED	-0.194385965	0	0.173	6.21E-15
DCLRE1B	-0.194035088	0	0.169	8.29E-17
SPECC1	-0.194035088	0	0.158	1.49E-06
NOB1	-0.193684211	0	0.171	1.95E-14
IPO9	-0.193333333	0	0.166	7.02E-09
RFX3	-0.191578947	0	0.158	9.44E-05
STK40	-0.190877193	0	0.164	3.81E-11
ZNF318	-0.189824561	0	0.162	5.91E-10
PHF6	-0.189122807	0	0.168	3.99E-12
PRKRIR	-0.18877193	0	0.162	7.88E-19
ATMIN	-0.18877193	0	0.172	1.82E-11
AC084018.1	-0.188070175	0	0.162	9.14E-16
ORAI2	-0.188070175	0	0.151	8.76E-13
R3HCC1L	-0.188070175	0	0.164	2.68E-12
STRN4	-0.187368421	0	0.167	1.05E-07
PPM1F	-0.186666667	0	0.157	3.14E-06
BMPR2	-0.186315789	0	0.162	2.95E-12
NEDD4L	-0.185964912	0	0.16	1.78E-11
MGAT5	-0.185964912	0	0.158	3.79E-05
TBC1D12	-0.185614035	0	0.161	1.64E-19

PIP4K2B	-0.185614035	0	0.162	0.00112219
SNX29P2	-0.185263158	0	0.152	3.20E-12
FOXJ2	-0.185263158	0	0.165	5.81E-09
GDAP2	-0.185263158	0	0.163	1.53E-07
COG7	-0.184912281	0	0.16	3.37E-12
AQR	-0.184210526	0	0.162	7.07E-19
SNX20	-0.184210526	0	0.161	1.02E-06
C4orf29	-0.183859649	0	0.16	6.14E-18
TTBK2	-0.183508772	0	0.15	1.78E-13
CD99L2	-0.182105263	0	0.157	2.74E-09
TES	-0.181403509	0	0.155	4.38E-13
KIAA1731	-0.180350877	0	0.154	4.68E-06
EIF3J-AS1	-0.179298246	0	0.147	2.12E-13
WHSC1	-0.178947368	0	0.152	2.75E-08
RNASEL	-0.178596491	0	0.158	5.28E-11
TAB3	-0.178245614	0	0.15	2.04E-06
GPR160	-0.178245614	0	0.151	8.26E-05
WDR48	-0.176842105	0	0.159	5.16E-10
HIC2	-0.176140351	0	0.155	8.49E-07
WDTC1	-0.175438596	0	0.154	2.94E-09
SMAD7	-0.175438596	0	0.158	8.55E-05
ARFIP1	-0.175087719	0	0.159	6.67E-09
NAE1	-0.174385965	0	0.157	7.15E-16
USP24	-0.173684211	0	0.156	1.83E-07
ARHGAP19	-0.173684211	0	0.159	8.81E-05
ZNF33A	-0.173684211	0	0.148	0.003063948
CSE1L	-0.17122807	0	0.152	7.09E-14
ENTPD4	-0.170877193	0	0.147	7.26E-12
ZNF263	-0.170526316	0	0.153	2.78E-10
DLG1	-0.170175439	0	0.144	2.05E-12
NUDT5	-0.168421053	0	0.147	1.07E-07
ZNF736	-0.167719298	0	0.145	2.42E-07
THAP6	-0.166666667	0	0.146	1.23E-11
ZMYND15	-0.166315789	0	0.141	7.31E-14
MX2	-0.165964912	0	0.139	2.68E-11
FAM120B	-0.165614035	0	0.149	1.47E-09
PPP1R3B	-0.164912281	0	0.148	1.21E-11
ELF4	-0.163859649	0	0.148	2.99E-09
ZNF493	-0.163859649	0	0.142	4.75E-07
TXLNG	-0.162807018	0	0.143	4.16E-12
LAMC1	-0.162807018	0	0.14	6.28E-12
THAP5	-0.162105263	0	0.151	2.82E-13
HMGXB3	-0.162105263	0	0.144	5.61E-11
MAML1	-0.161403509	0	0.147	3.21E-11
SRFBP1	-0.161403509	0	0.145	2.74E-07
TFIP11	-0.161052632	0	0.149	5.18E-15
TEN1	-0.160701754	0	0.15	1.46E-23
NFATC1	-0.160701754	0	0.146	6.24E-12
ARFGAP1	-0.160350877	0	0.142	7.90E-15
TWISTNB	-0.160350877	0	0.139	3.00E-11
PLA2G4A	-0.160350877	0	0.138	7.68E-10
AGL	-0.160350877	0	0.144	3.34E-09

SLC36A1	-0.16	0	0.135	3.25E-06
FAM8A1	-0.159298246	0	0.144	3.78E-10
LSM14B	-0.159298246	0	0.149	2.23E-06
PLAGL1	-0.158947368	0	0.134	5.12E-16
THUMPD2	-0.158245614	0	0.135	5.56E-11
HMGCS1	-0.157192982	0	0.136	5.12E-12
PLCB3	-0.156140351	0	0.142	4.11E-12
PPP1R3E	-0.155789474	0	0.123	6.14E-12
MPND	-0.155087719	0	0.141	3.14E-11
AP5B1	-0.155087719	0	0.132	2.71E-10
ADAMTS10	-0.154736842	0	0.134	0.000900268
ZCCHC14	-0.153684211	0	0.135	4.18E-05
CAMKK2	-0.152631579	0	0.135	1.27E-08
ZNF100	-0.152280702	0	0.136	5.19E-10
VKORC1L1	-0.151578947	0	0.134	2.53E-14
SYNE1	-0.15122807	0	0.136	8.89E-16
ZNF844	-0.15122807	0	0.134	2.19E-09
SNX33	-0.15122807	0	0.134	4.54E-07
PPP1R12C	-0.150877193	0	0.135	7.71E-10
HERC3	-0.150175439	0	0.13	8.35E-16
PEX26	-0.150175439	0	0.127	1.50E-05
MARVELD1	-0.150175439	0	0.135	0.000356082
ZNF768	-0.149824561	0	0.139	4.81E-07
FKBP5	-0.149122807	0	0.13	0.001047787
REXO4	-0.148070175	0	0.132	1.17E-12
FAM160B1	-0.147719298	0	0.135	1.13E-12
GTPBP2	-0.147368421	0	0.128	5.11E-12
METTL4	-0.146315789	0	0.129	1.01E-20
MUT	-0.145964912	0	0.134	5.10E-12
C10orf118	-0.145263158	0	0.123	5.09E-10
TMEM180	-0.144912281	0	0.129	1.14E-09
EML2	-0.144912281	0	0.128	0.00066856
HINFP	-0.144561404	0	0.13	6.13E-13
P4HA2	-0.144561404	0	0.125	1.31E-09
NMB	-0.143859649	0	0.112	1.03E-13
VAV2	-0.143859649	0	0.127	1.71E-08
E2F3	-0.143859649	0	0.132	4.14E-08
UQCC2	-0.142807018	0	0.112	1.63E-06
PIAS4	-0.14245614	0	0.131	6.26E-08
ZBTB40	-0.141754386	0	0.124	0.004440554
GLB1L	-0.141754386	0	0.12	0.005597023
TMEM120B	-0.141403509	0	0.125	4.15E-10
EFTUD1	-0.141403509	0	0.132	4.09E-07
C9orf69	-0.140701754	0	0.131	1.67E-06
PDPR	-0.140701754	0	0.128	0.001285487
WWC2	-0.140701754	0	0.116	0.005856273
ZNF714	-0.140350877	0	0.121	4.30E-08
BAG3	-0.14	0	0.13	8.15E-12
CCDC57	-0.14	0	0.124	2.25E-08
TXLNA	-0.139649123	0	0.128	7.45E-11
ATAD2B	-0.139298246	0	0.122	9.85E-05
TMEM144	-0.138947368	0	0.119	2.73E-10

TMEM57	-0.138596491	0	0.127	3.57E-09
ZNF333	-0.137894737	0	0.124	0.000574995
ZNF44	-0.13754386	0	0.124	1.01E-10
WDR73	-0.13754386	0	0.118	6.09E-08
DVL1	-0.13754386	0	0.125	2.07E-06
PITRM1	-0.13754386	0	0.125	1.63E-05
EPAS1	-0.136491228	0	0.117	1.67E-09
MLH1	-0.136491228	0	0.127	2.45E-08
TSR1	-0.136491228	0	0.126	4.37E-08
ZNF639	-0.136491228	0	0.124	8.12E-07
MKL2	-0.136491228	0	0.123	0.001454474
IRGQ	-0.136140351	0	0.117	4.48E-05
TAPT1	-0.136140351	0	0.125	0.000228772
TRIM41	-0.135789474	0	0.123	3.12E-09
NSUN5	-0.135438596	0	0.123	8.02E-12
SUPV3L1	-0.135438596	0	0.122	9.00E-08
LEMD3	-0.135438596	0	0.125	0.000200042
FGFR1	-0.135087719	0	0.114	1.58E-09
AP001055.6	-0.135087719	0	0.118	0.001383413
GBP3	-0.134736842	0	0.122	0.00018594
UHRF1BP1	-0.134035088	0	0.122	3.75E-11
OXSM	-0.134035088	0	0.122	1.45E-06
CCDC77	-0.132631579	0	0.121	6.00E-11
SMARCD3	-0.132631579	0	0.113	6.85E-08
PTGES2	-0.132280702	0	0.123	6.40E-08
DKFZP761J1	-0.131929825	0	0.122	1.97E-09
MTAP	-0.131929825	0	0.123	3.95E-07
PPP1R12B	-0.131929825	0	0.118	2.66E-06
ECHDC3	-0.131578947	0	0.126	3.69E-19
LINC01010	-0.131578947	0	0.109	4.75E-16
TMEM168	-0.131578947	0	0.118	2.38E-10
METTL1	-0.13122807	0	0.095	2.71E-11
TXNRD2	-0.13122807	0	0.121	3.54E-07
TAF13	-0.130877193	0	0.121	3.45E-16
PSPC1	-0.130877193	0	0.12	3.02E-12
B3GNT5	-0.130526316	0	0.115	2.60E-17
XPNPEP3	-0.130175439	0	0.117	2.41E-09
NUP188	-0.129473684	0	0.121	2.61E-06
TJAP1	-0.129122807	0	0.116	2.35E-09
PIP5K1C	-0.128421053	0	0.116	5.10E-06
PKNOX1	-0.128070175	0	0.115	2.15E-08
SYNJ2BP	-0.127719298	0	0.119	3.29E-11
ATP8B4	-0.127719298	0	0.098	2.72E-08
YTHDC2	-0.127368421	0	0.112	0.000762686
CCDC159	-0.127017544	0	0.114	1.29E-14
LIN54	-0.127017544	0	0.118	2.16E-06
KLF16	-0.126315789	0	0.118	0.00040078
NDUFAF2	-0.125964912	0	0.115	7.73E-16
PRKCI	-0.125964912	0	0.113	4.87E-09
RAB11FIP4	-0.125964912	0	0.111	8.91E-09
RRP36	-0.125964912	0	0.119	7.14E-06
SIN3B	-0.125964912	0	0.116	0.004073493

ATRN	-0.125614035	0	0.118	1.78E-11
ZNF579	-0.125614035	0	0.12	1.42E-08
RAD51C	-0.125263158	0	0.115	4.03E-09
ERCC6	-0.125263158	0	0.113	1.75E-07
MIR29A	-0.124912281	0	0.112	0.001808048
DTYMK	-0.124561404	0	0.114	1.05E-09
TAF1C	-0.124561404	0	0.111	9.12E-08
CXCL1	-0.124561404	0	0.059	8.62E-07
TNPO2	-0.124210526	0	0.115	2.57E-09
RMDN3	-0.123859649	0	0.113	1.63E-07
STAU2	-0.123157895	0	0.112	5.43E-08
MSI2	-0.122807018	0	0.098	1.23E-08
FAM217B	-0.12245614	0	0.104	0.000504963
LILRA5	-0.12245614	0	0.101	0.00387888
KIAA0020	-0.122105263	0	0.106	8.79E-10
CNOT11	-0.122105263	0	0.114	5.62E-08
MED14	-0.122105263	0	0.115	8.21E-08
RP11-1114A	-0.122105263	0	0.101	7.21E-05
RGP1	-0.121754386	0	0.112	1.81E-06
CLOCK	-0.121403509	0	0.106	2.91E-08
ZFYVE26	-0.121052632	0	0.112	0.002216974
AGPAT9	-0.120701754	0	0.092	6.00E-09
TTLL5	-0.120701754	0	0.11	1.49E-07
ZNF121	-0.120701754	0	0.112	3.58E-05
TFDP2	-0.119649123	0	0.106	1.09E-11
ARID3B	-0.119649123	0	0.106	0.000229547
RPAP2	-0.119649123	0	0.102	0.000389312
PRC1	-0.119298246	0	0.095	0.000115372
KAT2B	-0.118947368	0	0.108	7.61E-09
INTS2	-0.118947368	0	0.108	1.22E-05
FER	-0.118947368	0	0.102	0.009203334
FAM98B	-0.118596491	0	0.105	9.90E-14
SAMD9L	-0.118596491	0	0.106	3.69E-13
TBL2	-0.118596491	0	0.11	1.30E-11
GCN1L1	-0.118596491	0	0.112	0.002487381
HCG11	-0.118245614	0	0.111	1.25E-13
CCDC30	-0.118245614	0	0.096	6.90E-09
SCAI	-0.118245614	0	0.101	8.18E-09
EID3	-0.117894737	0	0.094	3.63E-14
MMS19	-0.117894737	0	0.11	2.48E-05
ZKSCAN8	-0.117192982	0	0.107	4.19E-08
SLC30A6	-0.117192982	0	0.106	5.18E-07
TMEM199	-0.116842105	0	0.107	1.61E-11
XRRA1	-0.116842105	0	0.109	1.85E-11
ZSCAN30	-0.116842105	0	0.101	0.000176314
ZCCHC3	-0.116842105	0	0.105	0.000543525
MIR181A1H(	-0.116491228	0	0.105	6.36E-11
RP11-383J2	-0.116491228	0	0.105	3.08E-07
ADA	-0.116491228	0	0.106	1.19E-06
RP11-611L7	-0.116491228	0	0.108	0.000155005
HIVEP2	-0.115087719	0	0.098	0.00088015
GGH	-0.114736842	0	0.093	6.91E-18

TFB1M	-0.114736842	0	0.105	6.03E-12
SPICE1	-0.114736842	0	0.105	1.62E-05
PPP1R13L	-0.114385965	0	0.103	7.84E-12
SERPINB8	-0.114385965	0	0.105	8.55E-05
ANKRD40	-0.114035088	0	0.108	3.57E-12
TUBD1	-0.114035088	0	0.104	5.14E-09
WDR47	-0.112982456	0	0.104	1.07E-15
RUFY3	-0.112982456	0	0.098	1.79E-11
PREB	-0.112982456	0	0.104	2.65E-10
R3HDM1	-0.112631579	0	0.101	3.24E-08
USP19	-0.112631579	0	0.106	8.91E-05
BARD1	-0.112280702	0	0.099	0.002226082
FAM53B	-0.112280702	0	0.1	0.004257061
MAP4K4	-0.111929825	0	0.098	0.007917857
CARM1	-0.110877193	0	0.105	6.15E-06
MAPK8	-0.110526316	0	0.103	2.98E-12
TRPM4	-0.110175439	0	0.099	1.13E-07
RANGRF	-0.109824561	0	0.104	2.82E-13
MIS18A	-0.109824561	0	0.103	3.26E-09
RP11-1407C	-0.109473684	0	0.098	5.64E-12
HECTD3	-0.109473684	0	0.101	5.04E-08
PDE12	-0.109473684	0	0.1	0.001175228
TADA2B	-0.109122807	0	0.104	5.88E-06
STX2	-0.109122807	0	0.095	5.63E-05
CDIP1	-0.10877193	0	0.103	5.33E-11
SCIMP	-0.108070175	0	0.098	0.00344579
APOC2	-0.108070175	0	0.062	0.004381135
CDC23	-0.107719298	0	0.102	1.10E-11
DBF4	-0.107719298	0	0.096	0.000196659
OSGIN1	-0.107719298	0	0.088	0.000861417
RP13-582O9	-0.107368421	0	0.098	8.78E-10
PARP11	-0.107017544	0	0.096	5.00E-08
ABI2	-0.107017544	0	0.09	1.09E-06
FCHSD1	-0.106666667	0	0.1	9.75E-05
AP4M1	-0.106315789	0	0.096	1.92E-12
ZNF512	-0.106315789	0	0.098	5.70E-08
GMDS-AS1	-0.106315789	0	0.096	8.72E-07
PITPNM1	-0.106315789	0	0.1	1.60E-05
TP53BP1	-0.106315789	0	0.095	0.008280647
MED24	-0.105964912	0	0.099	1.13E-12
ELMOD3	-0.105614035	0	0.094	6.93E-07
ACAT1	-0.105263158	0	0.097	2.32E-20
ZNF529	-0.104912281	0	0.096	2.16E-10
NUDT16	-0.104912281	0	0.1	5.08E-08
TTLL12	-0.104561404	0	0.099	3.13E-05
KNTC1	-0.104561404	0	0.092	0.008230781
AC090186.1	-0.104210526	0	0.096	9.54E-13
CSTF1	-0.104210526	0	0.097	1.09E-07
RPAP3	-0.103859649	0	0.094	0.00133466
C6orf57	-0.103508772	0	0.099	1.93E-08
MB21D2	-0.103508772	0	0.094	3.93E-05
ZNF746	-0.103508772	0	0.095	0.00825738

RP11-804H8	-0.103157895	0	0.093	3.23E-12
HKR1	-0.103157895	0	0.097	1.58E-06
STIM2	-0.103157895	0	0.091	0.003639811
ATXN7L3	-0.102807018	0	0.096	0.000252097
ANKRD50	-0.10245614	0	0.089	3.23E-07
FMNL3	-0.10245614	0	0.092	5.59E-05
ZEB2-AS1	-0.10245614	0	0.09	0.000388541
TELO2	-0.101403509	0	0.095	1.16E-06
CTB-55O6.1	-0.100701754	0	0.089	0.000352346
NLN	-0.100350877	0	0.086	1.41E-06
LPCAT4	-0.1	0	0.095	7.04E-14
HS3ST1	-0.099649123	0	0.086	1.44E-11
HEMK1	-0.099649123	0	0.092	2.17E-05
MTHFD2L	-0.099298246	0	0.091	1.21E-14
RAB40C	-0.099298246	0	0.089	2.97E-08
ABCD3	-0.098947368	0	0.092	7.41E-07
ITFG2	-0.098596491	0	0.089	5.20E-11
TBP	-0.098596491	0	0.091	2.82E-07
TMEM102	-0.098596491	0	0.094	0.000793528
DNAJA3	-0.098596491	0	0.091	0.001689718
RAB38	-0.098245614	0	0.093	3.39E-06
BRCA1	-0.098245614	0	0.081	4.22E-05
CTDP1	-0.097894737	0	0.091	0.000409752
ENTPD1	-0.097192982	0	0.084	7.28E-10
SDR39U1	-0.096842105	0	0.089	9.93E-13
RP11-295P9	-0.096842105	0	0.086	0.005013896
IFRD2	-0.096491228	0	0.091	6.37E-26
SLC38A9	-0.096491228	0	0.089	2.66E-10
SCO1	-0.096140351	0	0.093	8.38E-07
PHLDB3	-0.095789474	0	0.09	7.09E-14
UGGT1	-0.095789474	0	0.091	6.24E-12
NKIRAS1	-0.095789474	0	0.09	1.02E-09
ACAP3	-0.095789474	0	0.086	6.89E-07
ZNF3	-0.095789474	0	0.089	1.27E-06
PTGIR	-0.095438596	0	0.081	2.53E-10
CCDC97	-0.095438596	0	0.089	5.67E-10
ZMPSTE24	-0.095087719	0	0.087	5.45E-10
CTA-292E10	-0.094736842	0	0.08	4.39E-06
CHPF2	-0.094385965	0	0.089	1.92E-07
TULP4	-0.094385965	0	0.086	1.91E-05
GTPBP3	-0.094385965	0	0.088	0.006286921
TTC5	-0.093684211	0	0.089	0.003396233
C22orf46	-0.093333333	0	0.085	9.57E-06
NPIPA1	-0.092982456	0	0.084	8.03E-13
MRPS26	-0.092982456	0	0.085	4.65E-12
TM4SF19	-0.092982456	0	0.067	3.07E-08
TEX10	-0.092982456	0	0.086	1.35E-07
PTPN9	-0.092631579	0	0.088	1.03E-11
APPL2	-0.092631579	0	0.084	0.000125592
NR1D1	-0.091929825	0	0.081	7.79E-10
HAGHL	-0.091578947	0	0.084	2.42E-08
MOB3C	-0.090877193	0	0.082	2.18E-06

NBPF11	-0.090877193	0	0.081	0.000979838
HELB	-0.090877193	0	0.08	0.002880848
CBWD6	-0.090526316	0	0.081	2.83E-12
ZNF814	-0.090526316	0	0.083	1.74E-08
RPPH1	-0.090175439	0	0.063	0.003908061
BTN2A2	-0.090175439	0	0.082	0.004222116
TIMM21	-0.089473684	0	0.086	2.23E-07
GTPBP1	-0.089122807	0	0.082	4.90E-10
FAM50B	-0.089122807	0	0.084	2.96E-08
SH2B1	-0.089122807	0	0.08	7.50E-08
KRBOX4	-0.08877193	0	0.085	0.000112113
SMARCD1	-0.08877193	0	0.084	0.000123191
KIAA1279	-0.088421053	0	0.081	2.32E-08
SLC25A16	-0.088421053	0	0.081	4.23E-05
TOR1B	-0.087017544	0	0.082	0.000336538
ULK2	-0.086666667	0	0.082	1.56E-05
ZNF320	-0.086315789	0	0.079	8.60E-07
EXOSC5	-0.085964912	0	0.084	1.49E-06
AC009948.5	-0.084912281	0	0.076	5.15E-06
FAM220A	-0.084561404	0	0.079	3.81E-07
NBPF14	-0.083859649	0	0.076	9.05E-10
LINC00094	-0.083508772	0	0.079	9.84E-09
CBFA2T3	-0.083508772	0	0.07	4.74E-05
TATDN2	-0.083508772	0	0.079	0.001776324
NOL9	-0.083508772	0	0.076	0.004600646
MLYCD	-0.083157895	0	0.079	0.000105854
RP11-85F14	-0.082807018	0	0.076	9.15E-08
METTL8	-0.082807018	0	0.075	2.09E-05
COG1	-0.08245614	0	0.079	1.66E-06
CYB561D1	-0.08245614	0	0.078	0.000143834
GEMIN2	-0.082105263	0	0.078	1.23E-12
PROSER1	-0.082105263	0	0.079	0.000926486
ZNF429	-0.082105263	0	0.072	0.008898491
VCPKMT	-0.081754386	0	0.076	1.52E-09
MTERF	-0.081754386	0	0.077	2.55E-07
C5orf56	-0.081403509	0	0.078	1.58E-17
DFNB31	-0.081403509	0	0.075	1.14E-14
MTG2	-0.081403509	0	0.077	1.09E-08
G6PC3	-0.081403509	0	0.076	2.27E-08
RP11-1094M	-0.081403509	0	0.076	0.000303811
NUP155	-0.081403509	0	0.077	0.000663776
POLR3K	-0.081052632	0	0.078	0.003094951
ZNF496	-0.080350877	0	0.076	0.0013032
PIGL	-0.08	0	0.074	3.03E-10
TMEM129	-0.08	0	0.076	2.22E-05
HDAC7	-0.079649123	0	0.072	7.70E-07
HELZ2	-0.079649123	0	0.074	0.006213012
GSPT2	-0.079298246	0	0.074	0.001864545
GNL3L	-0.078245614	0	0.071	0.001257434
POLR3F	-0.077894737	0	0.074	0.006036181
SNORD3D	-0.07754386	0	0.069	0.000571252
CPSF4	-0.07754386	0	0.072	0.001833458

PIF1	-0.076842105	0	0.061	6.38E-10
ISOC1	-0.076491228	0	0.074	2.57E-08
CASP7	-0.076491228	0	0.073	3.00E-06
DPAGT1	-0.076491228	0	0.072	0.00161628
MAP7D3	-0.076140351	0	0.067	1.88E-08
FRMD4A	-0.076140351	0	0.067	5.12E-08
MYO19	-0.076140351	0	0.071	7.14E-07
GTF2IRD2B	-0.076140351	0	0.069	1.23E-06
COQ6	-0.075789474	0	0.072	6.24E-06
THEM4	-0.075789474	0	0.068	4.39E-05
LINC00926	-0.075789474	0	0.064	0.000320774
EXOC6	-0.075789474	0	0.071	0.006994777
NCBP1	-0.075438596	0	0.073	0.008886815
TSSC1	-0.075087719	0	0.072	3.27E-11
RINT1	-0.075087719	0	0.072	3.35E-08
MFSD2A	-0.075087719	0	0.064	5.83E-05
MANEA-AS1	-0.074736842	0	0.07	2.28E-05
TCEANC2	-0.074736842	0	0.069	0.00138859
AK9	-0.074035088	0	0.068	6.19E-08
TRIM26	-0.073684211	0	0.072	1.39E-05
RNF125	-0.073684211	0	0.065	0.001107028
NAPB	-0.073333333	0	0.069	0.007720301
ZNF775	-0.072982456	0	0.069	0.000413335
SIRT3	-0.072631579	0	0.069	0.002594729
GTPBP10	-0.072280702	0	0.066	0.000116286
COG8	-0.071578947	0	0.068	4.24E-06
PRKAB1	-0.071578947	0	0.066	0.00030317
HEATR3	-0.071578947	0	0.064	0.003682213
FECH	-0.07122807	0	0.069	1.33E-06
ALS2	-0.07122807	0	0.065	0.000973006
MINPP1	-0.070877193	0	0.067	1.91E-05
KIAA0101	-0.070877193	0	0.019	0.000268998
MIEF2	-0.070526316	0	0.068	8.93E-05
DIABLO	-0.070175439	0	0.065	4.67E-15
ZNF250	-0.070175439	0	0.067	0.000210769
TTL	-0.070175439	0	0.062	0.000456708
SLC46A3	-0.070175439	0	0.061	0.004394626
TPCN2	-0.069824561	0	0.066	0.000155906
LL22NC03-2	-0.069473684	0	0.057	3.28E-08
CLDN15	-0.069473684	0	0.063	0.004716614
IL10	-0.06877193	0	0.061	9.82E-06
ABHD17B	-0.068421053	0	0.062	7.82E-05
TMEM186	-0.068421053	0	0.065	0.000787126
PPCDC	-0.068070175	0	0.066	0.001635842
RIMKLB	-0.066666667	0	0.056	1.52E-08
DPH7	-0.066666667	0	0.062	0.002152956
SPHK2	-0.065964912	0	0.062	1.44E-08
CXorf56	-0.065964912	0	0.064	2.03E-07
RP5-1085F1	-0.065614035	0	0.061	0.001722027
C21orf91	-0.065614035	0	0.057	0.001790348
ATF7IP2	-0.065263158	0	0.058	7.71E-08
TBL3	-0.065263158	0	0.062	3.34E-05

RAB24	-0.064912281	0	0.062	9.26E-09
ZNF600	-0.064561404	0	0.061	9.30E-08
RP4-635E18	-0.064561404	0	0.061	8.87E-07
SDS	-0.064561404	0	0.039	7.03E-05
HOXB2	-0.064210526	0	0.059	3.37E-20
AMN1	-0.064210526	0	0.061	1.39E-06
LPIN1	-0.064210526	0	0.058	4.93E-05
TPD52	-0.064210526	0	0.057	0.000138844
C7orf31	-0.064210526	0	0.061	0.005496808
TMCO6	-0.063859649	0	0.06	1.14E-06
NIPAL3	-0.063859649	0	0.058	3.10E-05
OGFOD1	-0.063859649	0	0.058	0.000124999
C12orf49	-0.063508772	0	0.06	2.85E-09
RP5-899E9.1	-0.063508772	0	0.061	5.64E-09
ELP4	-0.063508772	0	0.06	0.009016975
ODF2	-0.063157895	0	0.059	1.46E-06
RP11-443B7	-0.06245614	0	0.058	3.74E-06
IGSF9	-0.06245614	0	0.059	0.008649509
LACE1	-0.062105263	0	0.059	2.21E-05
JMJD8	-0.061754386	0	0.058	0.000261492
PSME3	-0.061754386	0	0.055	0.000407942
TIMM8A	-0.061403509	0	0.059	8.56E-08
UBOX5	-0.061403509	0	0.058	0.003053975
RP11-215P8	-0.061052632	0	0.055	0.000264167
INO80C	-0.060701754	0	0.058	3.09E-08
DAGLA	-0.060350877	0	0.055	0.000942604
COX10	-0.059649123	0	0.056	5.90E-11
MKS1	-0.059649123	0	0.058	0.001805012
MAD2L1	-0.059298246	0	0.045	6.04E-07
PRR11	-0.058947368	0	0.05	0.000490897
TBC1D32	-0.058947368	0	0.054	0.000887656
CHST13	-0.058947368	0	0.054	0.001208123
RAD52	-0.058596491	0	0.056	0.002855925
ABCC5	-0.058245614	0	0.053	0.000411416
CTC-425F1.4	-0.057894737	0	0.052	3.63E-14
APOL1	-0.05754386	0	0.055	1.85E-13
ZNF574	-0.05754386	0	0.055	0.000300933
ARL 10.00	-0.057192982	0	0.051	1.64E-05
KAT2A	-0.056491228	0	0.052	0.000232396
SYNC	-0.056140351	0	0.052	3.57E-19
RP11-356N1	-0.056140351	0	0.054	3.23E-07
SURF2	-0.055789474	0	0.053	3.15E-05
PIGQ	-0.054736842	0	0.052	0.002025013
FEM1A	-0.054385965	0	0.052	0.000558665
ATP13A2	-0.054385965	0	0.05	0.008339364
FLAD1	-0.054035088	0	0.051	0.000208048
LGALS3BP	-0.054035088	0	0.046	0.000584886
LCAT	-0.054035088	0	0.053	0.008607404
GPSM2	-0.053684211	0	0.047	0.000178549
KIAA0040	-0.053684211	0	0.049	0.002340056
DLEU1	-0.052982456	0	0.049	1.73E-06
AHDC1	-0.052631579	0	0.051	0.002223994

CARD14	-0.052280702	0	0.049	0.000712616
PAXIP1-AS2	-0.052280702	0	0.051	0.003027831
C4orf46	-0.051929825	0	0.051	3.51E-09
RP11-752G1	-0.051929825	0	0.051	0.004223122
ARHGAP11E	-0.051578947	0	0.045	0.002392638
PROC	-0.05122807	0	0.043	6.64E-11
PTPDC1	-0.05122807	0	0.048	0.000267603
ALKBH2	-0.05122807	0	0.05	0.00308037
IQSEC2	-0.050877193	0	0.049	1.67E-05
RP11-708J1	-0.050877193	0	0.046	0.000621458
RP11-294J2	-0.050175439	0	0.046	7.69E-14
EPS8	-0.050175439	0	0.047	1.49E-13
ABCB8	-0.050175439	0	0.048	0.002516002
CD22	-0.049473684	0	0.048	7.88E-06
UCHL1	-0.049473684	0	0.042	0.00075792
TTI2	-0.049473684	0	0.048	0.00273672
AARSD1	-0.049122807	0	0.045	9.77E-09
TUBE1	-0.048070175	0	0.045	2.16E-05
C18orf54	-0.048070175	0	0.044	0.005775376
KLHL18	-0.047719298	0	0.045	0.000670473
ZNF703	-0.047719298	0	0.043	0.000942303
RP11-395A1	-0.047368421	0	0.044	0.00363182
TARBP1	-0.045614035	0	0.042	0.000224021
RP11-81A22	-0.045263158	0	0.044	4.68E-08
CCDC142	-0.045263158	0	0.041	0.000287546
C21orf119	-0.045263158	0	0.043	0.000593963
BCAR3	-0.044561404	0	0.043	0.000365332
TIE1	-0.043859649	0	0.033	4.23E-11
SHOX2	-0.043859649	0	0.036	0.006876383
RP5-1065J2	-0.043508772	0	0.036	0.007773117
TMEM255B	-0.043157895	0	0.042	3.50E-08
TVP23C	-0.043157895	0	0.04	9.44E-06
TMCO4	-0.042807018	0	0.04	0.00990121
LY9	-0.04245614	0	0.037	6.02E-06
MMAA	-0.04245614	0	0.041	0.001725731
GATSL3	-0.042105263	0	0.04	4.81E-07
CTAGE5	-0.041052632	0	0.041	0.004128152
SNRPN	-0.039298246	0	0.037	5.96E-06
AC016831.7	-0.038245614	0	0.036	4.94E-14
RP3-473L9.4	-0.038245614	0	0.036	4.39E-10
SCRG1	-0.038245614	0	0.034	0.003990626
LINC00115	-0.037894737	0	0.032	1.29E-05
NOP16	-0.037894737	0	0.037	1.32E-05
C5orf28	-0.037894737	0	0.037	3.43E-05
ALDH1A2	-0.037894737	0	0.032	9.46E-05
ARHGEF12	-0.037894737	0	0.036	0.009558303
CTC-246B18	-0.03754386	0	0.034	5.25E-11
ZNF584	-0.036842105	0	0.035	2.48E-05
ORC6	-0.036842105	0	0.033	0.006553567
ZFP82	-0.036491228	0	0.035	3.67E-05
PXN-AS1	-0.036491228	0	0.036	0.009307412
HDAC9	-0.035087719	0	0.033	1.58E-06

NIPAL2	-0.035087719	0	0.033	0.002978029
RP11-192H2	-0.033333333	0	0.032	1.37E-08
AP000769.1	-0.032982456	0	0.032	9.38E-05
DPP4	-0.032982456	0	0.025	0.007812396
USP6	-0.032280702	0	0.032	0.003995942
ZNF84	-0.031578947	0	0.031	1.41E-10
EIF5AL1	-0.031578947	0	0.031	1.51E-07
SLC5A6	-0.030526316	0	0.03	0.007696142
NPIPB15	-0.029122807	0	0.028	0.006165393
BMPR1A	-0.027719298	0	0.027	0.001389311

Footnotes:

1- Value refers to average differential expression within one subset of scaled pearsons residuals. Positive values represent increased gene expression in HIV+ cells without ART. Negative values represent increased gene expression in HIV+ cells with ART

2- Percentage of cells, within the cluster ID for which the gene is a marker, that detect the gene

3- Percentage of all the other cells, excluding the cluster ID for which the gene is a marker, that detect the gene
