# Supplementary material for: Interactions of Monocytes, HIV, and ART Identified by an Innovative scRNAseq Pipeline: Pathways to Reservoirs and HIV-Associated Comorbidities
Source: mBio. 2020 Jul 28;11(4):e01037-20. doi: 10.1128/mBio.01037-20 (PMC7387797; doi:10.1128/mBio.01037-20)
Supplement: TABLE S4 [file mBio.01037-20-st004.pdf]

Supplementary Table 4: List of differentially expressed markers modulated by ART-treatment on each mature monocyte cluster from the integrated dataset of HIV-infected mature monocytes with and without ART-treatment (Cluster X with ART-treatment vs Cluster X without ART-treatment).

Gene.ID	Avg. Diff.[1]	Pct.1[2]	Pct.2[3]	P.Value.Adj	Cluster.ID	Comments
APOC1	3.32482547	0.732	0.686	4.04E-56	0	Shared with clusters 0, 1, 2, 3, 4, 5, 6, and 7
APOE	2.87067989	0.746	0.763	1.08E-106	0	Shared with clusters 0, 2, 3, 5, 6, and 7
RGS1	1.88365654	0.692	0.452	1.86E-07	0	Shared with clusters 0 and 5
CD74	0.87698069	0.647	0.684	2.45E-19	0	Shared with clusters 0 and 2
HLA-DQB1	0.73064371	0.575	0.22	0.00033101	0	
HLA-DPB1	0.18276889	0.346	0.284	2.91E-19	0	
HLA-DQA1	0.12072014	0.149	0.062	3.09E-08	0	
HLA-DMA	0.0610958	0.484	0.406	1.42E-13	0	Shared with clusters 0 and 2
CCL5	0.04590349	0.082	0.082	0.00128394	0	Shared with clusters 0, 6, and 7
OAS1	-0.0316326	0.024	0.049	8.58E-13	0	Shared with clusters 0, 2, and 3
EMILIN1	-0.0316534	0.007	0.033	5.84E-33	0	Shared with clusters 0, 1, 4, 5, and 6
SYNGR1	-0.0332177	0.042	0.071	0.00184483	0	Shared with clusters 0, 2, and 7
UCP2	-0.035283	0.123	0.138	1.72E-05	0	Shared with clusters 0 and 3
SPARC	-0.0377788	0.012	0.036	3.69E-15	0	Shared with clusters 0, 2, and 3
MARCH2	-0.0387304	0.04	0.075	1.43E-08	0	Shared with clusters 0, 2, 3, 5, and 7
CST6	-0.0388487	0.019	0.051	8.40E-08	0	
NCS1	-0.0399087	0.005	0.038	0.00070927	0	Shared with clusters 0, 1, 3, 4, 5, and 6
IL7R	-0.0410027	0.005	0.036	1.97E-05	0	Shared with clusters 0, 2, 3, 6, and 7
CD276	-0.0431392	0.015	0.052	0.00041917	0	Shared with clusters 0, 3, 5, and 6
STIP1	-0.0438769	0.024	0.059	5.97E-05	0	Shared with clusters 0, 3, 5, and 6
CCND2	-0.0496209	0.009	0.052	1.05E-10	0	Shared with clusters 0, 3, and 6
HK3	-0.0505779	0.016	0.056	2.08E-10	0	Shared with clusters 0, 1, 3, 5, and 6
PHLDA3	-0.053144	0.026	0.07	1.16E-08	0	Shared with clusters 0, 3, 5, 6, and 7
ITGB5	-0.0540396	0.012	0.055	1.69E-06	0	Shared with clusters 0, 3, and 5, and 6
HLA-DPA1	-0.064121	0.35	0.336	6.95E-29	0	Shared with clusters 0 and 4
MS4A4A	-0.0648877	0.169	0.208	2.26E-06	0	Shared with clusters 0, 2, 5, and 7
MRAS	-0.0669494	0.047	0.098	1.64E-06	0	Shared with clusters 0, 3, and 5
MRC1L1	-0.0685575	0.013	0.069	0.00266066	0	Shared with clusters 0, 4, and 7
ITGB7	-0.070273	0.009	0.067	4.49E-21	0	Shared with clusters 0, 1, 2, and 3
ST20	-0.0723459	0.035	0.094	0.00424306	0	Shared with clusters 0, 5, and 7
CHI3L1	-0.073406	0.113	0.162	0	0	Shared with clusters 0, 1, 2, 3, 4, 5, 6, and 7
RP11-290F2	-0.0749416	0.011	0.076	0.00960833	0	
TREM2	-0.0773688	0.137	0.17	1.55E-09	0	Shared with clusters 0, 2, 3, 6, and 7
CD59	-0.0776658	0.109	0.161	1.34E-09	0	Shared with clusters 0, 1, 2, 3, 5, 6, and 7
GALM	-0.0779079	0.054	0.11	0.00115775	0	
CD300LB	-0.0793341	0.046	0.107	1.09E-05	0	Shared with clusters 0,1, 2, 3, and 5
CYB5A	-0.0803823	0.226	0.263	7.33E-18	0	Shared with clusters 0, 2, and 5
G6PD	-0.0810255	0.056	0.115	5.82E-24	0	Shared with clusters 0, 1, 2, 3, 4, 5, 6, and 7
C9orf16	-0.0820934	0.186	0.235	4.05E-10	0	Shared with clusters 0, 2, and 6
VENTX	-0.0871432	0.006	0.08	3.99E-08	0	Shared with clusters 0, 2, and 6
FDX1	-0.0876959	0.139	0.195	2.22E-27	0	Shared with clusters 0, 2, 3, 5, and 6
C20orf24	-0.0903596	0.15	0.212	0.00862634	0	Shared with clusters 0 and 5
MNDA	-0.0943255	0.089	0.148	8.90E-12	0	Shared with clusters 0, 2, 6, and 7
FABP4	-0.0992475	0.175	0.141	3.12E-51	0	Shared with clusters 0, 1, 2, 3, 4, 5, 6, and 7
EMP1	-0.1029044	0.184	0.234	0.00093277	0	Shared with clusters 0, 1, 3, 5, and 6
NDUFB2	-0.1034625	0.269	0.326	0.00950326	0	Shared with clusters 0, 2, 5, and 6
PPT1	-0.1149513	0.127	0.19	4.57E-18	0	Shared with clusters 0, 1, 2, 3, 6, and 7
GYPC	-0.1157934	0.234	0.288	0.00429851	0	
GLA	-0.1160113	0.118	0.194	0.00011809	0	Shared with clusters 0, 2, 5, and 7
TUBA1A	-0.117205	0.08	0.158	1.62E-08	0	Shared with clusters 0, 5, and 6
IGSF6	-0.1182201	0.092	0.154	0.00138816	0	Shared with clusters 0, 2, 5, and 6
MSMO1	-0.119793	0.008	0.101	3.64E-06	0	Shared with clusters 0, 5, 6, and 7
PPA1	-0.1210953	0.049	0.139	3.37E-05	0	
PTGR1	-0.1286491	0.023	0.122	9.84E-16	0	Shared with clusters 0, 1, 3, 5, and 6
LDHB	-0.130516	0.089	0.18	4.42E-13	0	Shared with clusters 0 and 6
IL1RAP	-0.1387723	0.017	0.127	0.00018431	0	Shared with clusters 0 and 6
CLCN6	-0.1430233	0.012	0.13	0.00046665	0	Shared with clusters 0, 2, and 7
FKBP1A	-0.1433886	0.186	0.253	4.05E-10	0	Shared with clusters 0, 1, 2, 3, 5, and 6

MT1E	-0.1465915	0.082	0.124	3.61E-224	0	Shared with clusters 0, 1, 2, 3, 4, 6,and 7
EVI2A	-0.1622841	0.869	0.885	0.00073391	0	Shared with clusters 0 and 6
MATK	-0.1630857	0.067	0.176	1.87E-32	0	Shared with clusters 0, 3, 4, 5, 6, and 7
CDCP1	-0.1750922	0.052	0.153	0.00303389	0	
ITGB1BP1	-0.1784169	0.06	0.192	1.10E-06	0	Shared with clusters 0, 3, 5, and 6
TAF10	-0.1792423	0.039	0.181	9.94E-08	0	Shared with clusters 0, 1, 2, and 6
C1QC	-0.1888295	0.036	0.14	1.30E-08	0	Shared with clusters 0, 3, and 5
PLBD1	-0.1927944	0.056	0.188	8.44E-21	0	Shared with clusters 0, 1, 2, and 6
CD93	-0.2142499	0.014	0.16	4.62E-08	0	Shared with clusters 0, 2, 5, and 6
LIPA	-0.2197898	0.406	0.492	1.55E-217	0	Shared with clusters 0, 1, 2, 3, 4, 5, 6, and 7
PYCARD	-0.2220204	0.35	0.412	4.46E-14	0	Shared with clusters 0 and 2
HSPA8	-0.2235075	0.161	0.29	2.63E-10	0	Shared with clusters 0, 3, and 5
PCOLCE2	-0.2248895	0.055	0.17	1.68E-05	0	
CYSTM1	-0.2257502	0.194	0.323	3.43E-06	0	Shared with clusters 0, 2, 3, 5, 6, and 7
QSER1	-0.2334518	0.023	0.197	0.00601998	0	
C1QA	-0.2336053	0.049	0.129	6.05E-57	0	Shared with clusters 0, 2, 3, 4, and 7
CD84	-0.2390544	0.078	0.197	3.17E-37	0	Shared with clusters 0, 1, 3, 5, 6, and 7
SIPA1L2	-0.2455164	0.021	0.205	0.00107058	0	
SECISBP2	-0.2588613	0.04	0.219	0.00532521	0	Shared with clusters 0 and 2
PRKCB	-0.2595782	0.06	0.217	0.00017027	0	Shared with clusters 0 and 5
RPN2	-0.2617015	0.035	0.218	0.0090983	0	Shared with clusters 0, 1, 2, 3, and 5
MT1G	-0.266037	0.093	0.193	0	0	Shared with clusters 0, 1, 3, 4, 5, 6,and 7
ATP2A3	-0.2696181	0.021	0.215	4.04E-05	0	
C3	-0.2697355	0.072	0.226	4.58E-06	0	Shared with clusters 0, 3, 4, 5, 6, and 7
TSPYL2	-0.2789369	0.099	0.261	0.00108875	0	
ABHD2	-0.2797513	0.037	0.234	0.00400873	0	Shared with clusters 0, 3, 5, and 7
TXNRD1	-0.2799135	0.061	0.204	1.77E-40	0	Shared with clusters 0, 1, 3, 4, 5, 6, and 7
MYOF	-0.286644	0.028	0.232	8.15E-07	0	Shared with clusters 0, 3, 5, and 7
ALCAM	-0.2929559	0.049	0.229	1.52E-31	0	Shared with clusters 0, 1, 2, 3, 4 ,5, 6, and 7
CD48	-0.2958314	0.2	0.305	2.42E-10	0	Shared with clusters 0 and 2
A2M	-0.3025311	0.053	0.199	2.11E-33	0	Shared with clusters 0, 2, 3, and 7
SCPEP1	-0.3193925	0.065	0.248	2.37E-19	0	Shared with clusters 0, 1, 2, and 7
SLC25A5	-0.3230539	0.118	0.296	1.67E-22	0	Shared with clusters 0, 2, and 6
MAFK	-0.324561	0.07	0.293	0.00656007	0	
FABP5	-0.3245929	0.524	0.609	1.04E-172	0	Shared with clusters 0, 1, 2, 3, 4, 5, 6, and 7
DENND1B	-0.3270318	0.056	0.265	0.00113373	0	
C19orf10	-0.3306036	0.322	0.463	0.00100741	0	Shared with clusters 0, 2, and 5
SCD	-0.3351293	0.069	0.265	0.00030096	0	Shared with clusters 0, 2, 4, and 5
MMP14	-0.3421525	0.042	0.27	0.00220484	0	Shared with clusters 0 and 2
POGZ	-0.3518527	0.05	0.291	0.00922245	0	Shared with clusters 0 and 2
NFXL1	-0.3621732	0.014	0.189	3.56E-07	0	Shared with clusters 0, 2, 5, 6, and 7
HLA-DRB1	-0.3655254	0.462	0.425	4.76E-22	0	Shared with clusters 0, 1, and 4
THBD	-0.3682044	0.028	0.194	3.32E-06	0	Shared with clusters 0, 2, and 5
TNFSF13B	-0.3764557	0.25	0.303	0.00978367	0	Shared with clusters 0, 2, and 7
FBXO11	-0.3810594	0.072	0.329	0.0078562	0	
FAM20C	-0.4123685	0.073	0.289	3.71E-27	0	Shared with clusters 0, 1, 2, 3, 4, 5, 6, and 7
HMG20B	-0.4133224	0.183	0.398	3.17E-31	0	Shared with clusters 0, 1, 3, 4, 5, and 7
EWSR1	-0.4241284	0.082	0.346	0.00261274	0	
SLC7A7	-0.4307483	0.113	0.34	9.13E-12	0	Shared with clusters 0, 2, and 7
COX6C	-0.4361562	0.39	0.556	0.00656547	0	Shared with clusters 0, 1, 2, 5, and 6
GUSB	-0.4483409	0.117	0.369	1.34E-49	0	Shared with clusters 0, 1, 2, 3, 5, 6, and 7
MT1H	-0.4483551	0.059	0.15	5.51E-107	0	Shared with clusters 0, 1, 3, 4, 5, and 7
SLC16A10	-0.4484395	0.042	0.254	0.00454642	0	Shared with clusters 0, 2, and 7
MAP3K7CL	-0.4536038	0.183	0.316	3.27E-05	0	
KCNQ1OT1	-0.463592	0.078	0.274	0.00349009	0	
PTPN6	-0.4806498	0.121	0.384	6.58E-07	0	Shared with clusters 0, 3, 5, 6, and 7
BCL2A1	-0.4933592	0.216	0.4	2.26E-12	0	Shared with clusters 0, 2, 3, 5, 6, and 7
HMGA1	-0.499594	0.088	0.368	8.13E-08	0	Shared with clusters 0 and 6
S100A9	-0.5153945	0.146	0.32	1.37E-17	0	Shared with clusters 0, 1, 2, 3, 5, 6, and 7
MT1F	-0.5168601	0.146	0.365	0.00073208	0	
CD4	-0.5174421	0.147	0.365	0.0069541	0	Shared with clusters 0 and 7
DFNA5	-0.5280434	0.219	0.466	3.68E-05	0	Shared with clusters 0, 2, and 7
ZNF217	-0.5552844	0.06	0.378	1.99E-05	0	Shared with clusters 0 and 5
CALM3	-0.5560422	0.296	0.526	4.10E-07	0	Shared with clusters 0, 2, 3, 6, and 7

ATXN2L	-0.5644266	0.065	0.414	1.23E-06	0	Shared with clusters 0 and 2
PSMA7	-0.5724348	0.431	0.612	7.97E-10	0	Shared with clusters 0, 3, 5, and 6
EXOC3	-0.5993772	0.07	0.405	0.00060661	0	Shared with clusters 0, 2, 5, and 7
COX5B	-0.6027441	0.724	0.856	4.21E-13	0	Shared with clusters 0, 2, 5, and 6
KIAA1551	-0.6069529	0.071	0.392	5.38E-06	0	Shared with clusters 0, 2, 5, and 7
SLC36A4	-0.607705	0.153	0.452	0.00099731	0	Shared with clusters 0, 2, and 5
FERMT3	-0.6114529	0.137	0.45	7.66E-07	0	Shared with clusters 0, 1, 3, 5, 6, and 7
REV3L	-0.6128965	0.099	0.419	1.97E-07	0	Shared with clusters 0, 2, and 5
SP3	-0.6193936	0.105	0.452	0.00420087	0	Shared with clusters 0, 2, and 5
TFPI	-0.6194837	0.246	0.49	1.50E-08	0	
RGS10	-0.6255951	0.32	0.574	5.02E-07	0	Shared with clusters 0 and 2
PCNX	-0.6264683	0.064	0.428	7.51E-05	0	Shared with clusters 0, 2, and 5
LAMP1	-0.6330348	0.272	0.521	2.83E-18	0	Shared with clusters 0, 2, 3, 6, and 7
GM2A	-0.634578	0.159	0.465	7.27E-24	0	Shared with clusters 0, 2, 3, 5, 6, and 7
PTAFR	-0.6364457	0.074	0.394	0.00021414	0	Shared with clusters 0 and 7
MFSD12	-0.638967	0.104	0.429	2.41E-168	0	Shared with clusters 0, 1, 2, 3,4, 5, 6, and 7
CHML	-0.6447921	0.055	0.343	6.89E-05	0	Shared with clusters 0, 2, 5, and 7
HLA-DRA	-0.6454081	0.702	0.778	2.74E-13	0	Shared with clusters 0 and 4
CAPG	-0.6625319	0.249	0.497	1.69E-35	0	Shared with clusters 0, 1, 2, 3, 5, 6, and 7
SESTD1	-0.6648797	0.135	0.472	0.00066092	0	Shared with clusters 0, 2, and 5
MLLT6	-0.6674896	0.079	0.447	2.29E-06	0	Shared with clusters 0 and 2
GNA12	-0.6749458	0.052	0.447	0.00848978	0	Shared with clusters 0 and 7
LPCAT1	-0.6756925	0.137	0.477	0.00157332	0	Shared with clusters 0 and 2
RLF	-0.6839521	0.095	0.469	0.00584681	0	Shared with clusters 0, 2, and 5
IQGAP2	-0.6944404	0.067	0.365	0.00093204	0	Shared with clusters 0, 1, and 2
DBI	-0.714969	0.35	0.654	5.27E-10	0	Shared with clusters 0, 2, 5, and 6
CTSL	-0.7163612	0.305	0.491	2.77E-09	0	Shared with clusters 0, 1, 2, 3, 5, 6, and 7
ANXA11	-0.7266332	0.169	0.538	6.35E-07	0	Shared with clusters 0, 2, 3, and 5
PIK3R5	-0.7369284	0.11	0.492	0.00046537	0	Shared with clusters 0 and 2
GK	-0.7394278	0.123	0.472	0.0016319	0	
FAM133B	-0.7672184	0.237	0.559	0.00402251	0	
SERPINB2	-0.7723592	0.063	0.242	1.54E-21	0	Shared with clusters 0, 1, 2, and 5
ITPR1	-0.7748476	0.117	0.456	0.00175649	0	Shared with clusters 0 and 2
CFL1	-0.7775953	0.448	0.689	6.10E-11	0	Shared with clusters 0, 1, 2, 3, 5, 6, and 7
ITGAM	-0.7780683	0.197	0.52	1.34E-07	0	Shared with clusters 0, 2, and 7
PLEK	-0.7792622	0.234	0.548	1.17E-05	0	Shared with clusters 0, 5, 6, and 7
SLC16A6	-0.7835381	0.102	0.364	0.00036596	0	Shared with clusters 0 and 7
PCF11	-0.7916663	0.135	0.51	0.00468503	0	Shared with clusters 0 and 2
ATP5G3	-0.7962093	0.352	0.684	1.18E-06	0	Shared with clusters 0, 5, and 6
NUPL1	-0.7967526	0.134	0.519	8.33E-06	0	Shared with clusters 0 and 2
TGFB1	-0.809601	0.156	0.547	3.75E-09	0	Shared with clusters 0, 1, 2, 3, 5, and 6
CLEC5A	-0.8103956	0.034	0.363	1.39E-08	0	Shared with clusters 0 and 2
TMEM170B	-0.8169694	0.053	0.476	1.16E-05	0	Shared with clusters 0, 2, and 5
CTSH	-0.8254172	0.364	0.653	8.09E-14	0	Shared with clusters 0, 2, and 7
SPEN	-0.8254626	0.064	0.498	1.06E-05	0	Shared with clusters 0 and 2
AKAP10	-0.8453507	0.087	0.52	4.32E-05	0	Shared with clusters 0, 2, and 5
TMEM176B	-0.861797	0.197	0.426	9.63E-05	0	Shared with clusters 0, 2, and 7
CLK1	-0.8759263	0.476	0.722	0.00668066	0	Shared with clusters 0 and 2
KIAA0930	-0.8764846	0.141	0.506	0.00124664	0	Shared with clusters 0, 2, 3, 5, and 7
RYBP	-0.8896025	0.114	0.531	5.61E-06	0	Shared with clusters 0, 2, 3, and 5
KMT2C	-0.930751	0.095	0.552	1.54E-07	0	Shared with clusters 0 and 2
TRIM38	-0.9550018	0.089	0.528	6.02E-05	0	Shared with clusters 0, 2, and 5
IL1RN	-0.9603878	0.298	0.565	2.98E-25	0	Shared with clusters 0, 1, 3, 4, 5 6, and 7
PRDX1	-0.9745387	0.434	0.725	2.01E-15	0	Shared with clusters 0, 1, 2, 3, 5, 6, and 7
MPHOSPH8	-1.0055262	0.227	0.543	0.00140029	0	Shared with clusters 0 and 5
SLC43A2	-1.0112372	0.153	0.54	1.22E-07	0	Shared with clusters 0 and 2
ETS2	-1.0239979	0.174	0.559	6.18E-05	0	Shared with clusters 0, 2, and 5
ALDH2	-1.0247969	0.117	0.547	0.00700572	0	Shared with clusters 0, 5, and 7
VNN 1.00	-1.0260464	0.016	0.393	1.56E-10	0	Shared with clusters 0, 2, and 5
SMCHD1	-1.0392446	0.119	0.588	3.70E-05	0	Shared with clusters 0, 2, and 5
AHR	-1.0404734	0.138	0.562	0.00476262	0	Shared with clusters 0 and 7
YME1L1	-1.0484219	0.17	0.615	2.22E-06	0	Shared with clusters 0 and 2
ARPC1B	-1.0509456	0.308	0.623	6.06E-78	0	Shared with clusters 0, 1, 2, 3, 5, 6, and 7
ZCCHC6	-1.0843756	0.178	0.606	6.20E-05	0	Shared with clusters 0, 5, and 7

JARID2	-1.0897437	0.224	0.613	0.0019091	0	Shared with clusters 0 and 2
OPN3	-1.1161159	0.315	0.631	5.55E-08	0	
PTPN12	-1.1275597	0.176	0.626	2.44E-06	0	Shared with clusters 0, 2, and 7
HIPK2	-1.1824257	0.103	0.607	1.60E-07	0	Shared with clusters 0, 2, and 7
MIDN	-1.18612	0.207	0.572	0.00964961	0	Shared with clusters 0 and 6
ACP5	-1.1861813	0.526	0.764	0.00022083	0	Shared with clusters 0, 2, 5, and 7
ARID5B	-1.1870002	0.192	0.616	0.00044833	0	
MYL12A	-1.1870215	0.402	0.797	6.61E-05	0	Shared with clusters 0, 5, and 6
PHIP	-1.1955811	0.185	0.642	0.00027224	0	Shared with clusters 0, 2, and 5
ATP6AP2	-1.2080504	0.314	0.73	4.82E-13	0	Shared with clusters 0, 1, 2, 3, 5, and 7
GSN	-1.209302	0.136	0.658	3.47E-49	0	Shared with clusters 0, 1, 2, 3, 5, 6, and 7
SPAG9	-1.2502215	0.2	0.671	0.0001068	0	Shared with clusters 0 and 2
FNDC3A	-1.2668593	0.176	0.611	6.49E-05	0	Shared with clusters 0, 2, 5, and 7
TXN	-1.2713853	0.577	0.847	1.06E-09	0	Shared with clusters 0, 2, 3, 5, and 6
EGLN3	-1.2791543	0.294	0.665	0.00608293	0	
NBPF10	-1.2811496	0.408	0.753	7.66E-08	0	Shared with clusters 0, 2, and 5
SFPQ	-1.2970953	0.184	0.676	4.27E-06	0	Shared with clusters 0 and 2
IRF2BP2	-1.3061476	0.213	0.639	0.00123349	0	
SERINC1	-1.3190671	0.188	0.717	6.20E-06	0	
MARCO	-1.3338085	0.105	0.458	0.00010705	0	Shared with clusters 0,1, 2, and 7
ST3GAL1	-1.3362948	0.193	0.657	0.00242902	0	Shared with clusters 0, 2, and 5
FAM107B	-1.3406466	0.204	0.674	0.00048267	0	
TNS1	-1.4064581	0.216	0.718	0.00832499	0	Shared with clusters 0, 2, and 7
FBP1	-1.4090913	0.274	0.748	6.56E-82	0	Shared with clusters 0, 1, 2, 3, 5, 6, and 7
FCN1	-1.4688855	0.441	0.629	0.00019687	0	Shared with clusters 0, 4, and 7
RPS27	-1.4731355	1	1	0.00061563	0	Shared with clusters 0, 2, and 7
FAM13A	-1.4818787	0.149	0.681	1.61E-07	0	Shared with clusters 0, 2, 5, and 7
TUBA1B	-1.4907898	0.314	0.788	7.55E-12	0	Shared with clusters 0, 1, 3, 5, 6, and 7
MT-ND4L	-1.5042748	0.155	0.567	0.00837317	0	
ANXA2	-1.5279814	0.478	0.868	7.11E-13	0	Shared with clusters 0, 1, 2, 3, 5, 6, and 7
CD81	-1.5297785	0.084	0.71	6.69E-28	0	Shared with clusters 0, 1, 2, 5, and 6
ZNF207	-1.5601737	0.271	0.784	5.75E-07	0	Shared with clusters 0 and 2
KMT2E	-1.6743389	0.298	0.822	0.00015362	0	Shared with clusters 0 and 2
ZNF395	-1.7146936	0.173	0.742	5.67E-09	0	Shared with clusters 0 and 2
BACH1	-1.7504489	0.174	0.766	0.00146598	0	Shared with clusters 0 and 2
CYP1B1	-1.7771321	0.153	0.579	0.00176818	0	Shared with clusters 0, 1, 2, 3, 5, and 7
COTL1	-1.820855	0.411	0.846	0.00023823	0	Shared with clusters 0, 2, and 6
PRRC2C	-1.8539822	0.226	0.809	0.00010932	0	Shared with clusters 0 and 2
BRD2	-1.8601221	0.266	0.792	5.60E-08	0	Shared with clusters 0, 2, and 5
FUS	-1.8793263	0.39	0.846	1.81E-09	0	Shared with clusters 0, 2, and 5
TIMP2	-2.0271692	0.231	0.852	0.00042424	0	Shared with clusters 0, 2, 5, and 7
ABCA1	-2.0973867	0.377	0.836	1.41E-08	0	Shared with clusters 0, 2, 3, and 7
THBS1	-2.1021546	0.121	0.414	3.47E-33	0	Shared with clusters 0, 2, 5, and 7
GSTO1	-2.1763662	0.673	0.96	0.00010608	0	Shared with clusters 0, 3, 5, 6, and 7
S100A4	-2.1983203	0.688	0.887	4.79E-18	0	Shared with clusters 0, 2, and 6
RNASET2	-2.2000572	0.776	0.961	0.0008757	0	Shared with clusters 0 and 2
CTSZ	-2.2254118	0.313	0.828	0.00031541	0	Shared with clusters 0, 1, 2, 3, 6, and 7
CXCR4	-2.339307	0.675	0.921	0.00593717	0	Shared with clusters 0, 2, 3, and 5
H1FX	-2.4302158	0.26	0.698	4.85E-06	0	Shared with clusters 0 and 2
MXI1	-2.4763235	0.308	0.887	0.00046386	0	Shared with clusters 0, 2, 3, and 5
RGCC	-2.4918597	0.719	0.872	5.64E-08	0	Shared with clusters 0, 1, 5, 6, and 7
MAP3K2	-2.5195317	0.294	0.879	0.00022377	0	Shared with clusters 0, 2, and 5
CTB-61M7.2	-2.5212604	0.297	0.807	4.50E-17	0	Shared with clusters 0, 2, 5, and 7
NAMPT	-2.5817614	0.482	0.895	4.90E-05	0	Shared with clusters 0, 2, and 5
MT1X	-2.6493547	0.454	0.644	1.46E-287	0	Shared with clusters 0, 1, 3, 4, 5, 6,and 7
BZW1	-2.6922959	0.401	0.884	1.46E-06	0	Shared with clusters 0 and 2
ZNF292	-2.7074171	0.198	0.891	2.34E-11	0	Shared with clusters 0, 2, 5, and 6
FCGR2A	-2.7704841	0.371	0.895	0.00101787	0	Shared with clusters 0, 5, and 7
PHLDA1	-2.9732171	0.507	0.861	8.72E-06	0	Shared with clusters 0, 2, and 7
FCGR3A	-3.0141401	0.269	0.85	1.85E-16	0	Shared with clusters 0, 2, 5, and 7
DMXL2	-3.0256896	0.332	0.922	0.00457068	0	Shared with clusters 0, 2, 5, and 7
PLD3	-3.0392641	0.658	0.91	0.00088418	0	Shared with clusters 0 and 7
ZEB2	-3.058229	0.498	0.944	2.27E-05	0	Shared with clusters 0, 2, and 7
P4HA1	-3.6152589	0.606	0.975	2.17E-05	0	Shared with clusters 0 and 2

SDC2	-3.991403	0.582	0.938	1.14E-23	0	Shared with clusters 0 and 7
MT2A	-4.0874901	0.741	0.826	3.07E-210	0	Shared with clusters 0, 2, 1, 3, 4, 5, 6, and 7
CD63	-4.2485946	0.947	0.998	6.74E-10	0	Shared with clusters 0, 1, 2, 3, 4, 5, 6, and 7
ACTB	-4.6316009	0.831	0.987	1.22E-06	0	Shared with clusters 0, 1, 3, 5, 6, and 7
TMSB4X	-4.6799394	0.992	0.999	4.70E-22	0	Shared with clusters 0 and 2
VEGFA	-4.6882057	0.283	0.944	0.00159734	0	Shared with clusters 0, 2, and 7
ACTG1	-4.7137456	0.56	0.96	2.46E-09	0	Shared with clusters 0 and 6
LGALS3	-4.7492969	0.853	0.991	8.13E-47	0	Shared with clusters 0, 1, 2, 3, 4, 5, 6, and 7
BNIP3L	-4.7719408	0.673	0.989	0.00222217	0	Shared with clusters 0 and 2
IER3	-4.7811147	0.488	0.895	0.00469871	0	Shared with clusters 0 ,2, and 5
HK2	-4.8151694	0.459	0.969	4.94E-05	0	Shared with clusters 0, 2, 3, and 7
EEF1B2	-4.8220891	0.364	0.981	7.81E-14	0	Shared with clusters 0, 5, and 6
FCGR2B	-4.9534619	0.605	0.943	1.44E-07	0	Shared with clusters 0, 5, and 7
RPL21	-4.9804884	0.997	1	3.13E-06	0	Shared with clusters 0, 2, and 5
SLC11A1	-5.0966895	0.407	0.95	1.74E-08	0	Shared with clusters 0, 2, and 7
RPSA	-5.1488245	0.651	0.989	4.13E-11	0	Shared with clusters 0, 1, 5, and 6
MT-ND5	-6.2270346	0.445	0.925	2.89E-16	0	
RPL34	-6.3506766	1	1	2.05E-08	0	Shared with clusters 0 and 2
MMP9	-6.6043479	0.598	0.96	1.09E-28	0	Shared with clusters 0, 1, 2, 3, 4, 5, 6, and 7
RPS15A	-6.7553208	0.998	1	5.01E-16	0	Shared with clusters 0 and 6
RPL39	-7.1063961	1	1	0.00027885	0	Shared with clusters 0 and 6
RPS5	-7.1432808	0.95	1	9.57E-10	0	Shared with clusters 0, 1, and 5
RPL41	-7.3245427	1	1	1.73E-09	0	Shared with clusters 0, 2, and 7
RPL12	-7.4540074	0.993	1	8.91E-07	0	
NDRG1	-8.0005287	0.593	0.994	8.39E-06	0	Shared with clusters 0 and 2
RPS19	-8.310952	1	1	2.20E-08	0	Shared with clusters 0 and 6
RPL35A	-8.5664765	0.998	1	0.00099001	0	
HLA-A	-8.8809407	0.974	0.999	0.00614409	0	
MT-ND1	-10.335099	0.604	0.973	0.00025624	0	Shared with clusters 0, 2, and 5
RPS2	-10.502278	0.993	1	1.92E-25	0	Shared with clusters 0 and 4
RPL3	-10.730888	0.991	1	1.45E-16	0	Shared with clusters 0, 1, and 4
RPS6	-10.757907	0.997	1	4.69E-17	0	
TXNIP	-11.269519	0.851	0.994	0.00159643	0	Shared with clusters 0, 2, 3, and 5
GLUL	-11.787927	0.928	1	1.31E-07	0	
CXCL5	-11.952718	0.345	0.763	1.24E-51	0	Shared with clusters 0, 2, 3, 5, and 7
VCAN	-12.098949	0.454	0.984	1.03E-38	0	Shared with clusters 0, 2, 5, 6, and 7
RPS3	-12.168567	0.994	1	1.27E-16	0	Shared with clusters 0, 5, 6, and 7
RPLP1	-12.455107	0.993	1	1.20E-22	0	Shared with clusters 0, 2, and 6
RPL32	-12.699758	0.997	1	4.13E-14	0	Shared with clusters 0, 1, and 4
RPS12	-13.379566	0.968	1	6.50E-37	0	Shared with clusters 0, 4, 5, and 6
RPS24	-14.010744	0.976	1	2.35E-39	0	Shared with clusters 0, 1, 2, and 6
CSTB	-14.34119	1	1	0.00587614	0	Shared with clusters 0, 1, 5, 6, and 7
RPS18	-14.871369	1	1	2.11E-36	0	Shared with clusters 0, 1, 4, and 6
RPS23	-15.203975	0.992	1	1.25E-24	0	Shared with clusters 0, 2, and 6
MT-CO3	-15.484437	0.819	0.994	0.00075097	0	Shared with clusters 0, 5, and 6
RPS27A	-15.872758	0.997	1	2.93E-11	0	Shared with clusters 0, 6, and 7
RPL7A	-16.649593	0.962	1	1.32E-22	0	Shared with clusters 0, 1, 4, 5, 6, and 7
RNASE1	-18.560458	0.321	0.967	4.27E-11	0	Shared with clusters 0, 4, and 7
MT-CO2	-18.859359	0.757	0.983	1.45E-07	0	Shared with clusters 0, 2, and 6
SPP1	-18.860227	0.957	0.999	5.49E-119	0	Shared with clusters 0, 1, 2, 3, 4, 5, 6, and 7
RPL30	-19.69651	0.994	1	2.64E-13	0	Shared with clusters 0, 6, and 7
IL8	-20.403241	1	0.998	6.20E-15	0	Shared with clusters 0, 2, 4, 5, and 6
RPL13	-20.976252	1	1	2.22E-26	0	Shared with clusters 0, 1, 4, 5, and 6
ZFP36L1	-21.189016	0.94	1	3.27E-19	0	Shared with clusters 0, 2, 5, 6, and 7
BTG1	-23.214122	0.999	1	0.00588268	0	Shared with clusters 0, 2, 6, and 7
RPS3A	-23.813308	1	1	1.23E-09	0	Shared with clusters 0, 1, and 6
RPL10	-28.694749	1	1	7.06E-09	0	Shared with clusters 0, 4, and 7
PLIN2	-36.631612	0.963	1	9.84E-08	0	Shared with clusters 0, 2, and 7
MT-ND4	-37.464076	0.9	0.997	9.11E-12	0	Shared with clusters 0, 2, 3, 5, and 7
MT-CO1	-38.467622	0.966	0.998	0.00172118	0	Shared with clusters 0, 1, 2, 3, and 5
FTL	-170.33427	1	1	7.13E-05	0	Shared with clusters 0 and 7
MALAT1	-447.99787	1	1	2.56E-07	0	Shared with clusters 0 and 2
APOC1	1.89059233	0.701	0.744	4.05E-05	1	Shared with clusters 0, 1, 2, 3, 4, 5, 6, and 7
RAMP1	-0.0270858	0.008	0.023	4.67E-09	1	Shared with clusters 1, 2, and 6

FLT1	-0.0340347	0.004	0.034	9.22E-14	1	Shared with clusters 1, 3, 4, 5, and 6
NCS1	-0.0411738	0.004	0.043	0.00132032	1	Shared with clusters 0, 1, 3, 4, 5, and 6
TGM2	-0.0417026	0.004	0.041	0.0001132	1	Shared with clusters 1, 3, 4, 5, and 6
EMILIN1	-0.0478791	0.006	0.05	2.92E-10	1	Shared with clusters 0, 1, 4, 5, and 6
S100A8	-0.0485212	0.042	0.078	3.60E-06	1	Shared with clusters 1, 2, and 6
GCLC	-0.0551758	0.026	0.071	1.17E-07	1	Shared with clusters 1, 2, 3, 4, 5, 6, and 7
HK3	-0.059026	0.016	0.073	3.08E-05	1	Shared with clusters 0, 1, 3, 5, and 6
EMP1	-0.0594672	0.216	0.248	6.41E-05	1	Shared with clusters 0, 1, 3, 5, and 6
C1QB	-0.0650124	0.014	0.044	0.00681974	1	Shared with clusters 1, 2, 5, and 6
G6PD	-0.0726984	0.067	0.129	1.56E-13	1	Shared with clusters 0, 1, 2, 3, 4, 5, 6, and 7
CD59	-0.0889498	0.137	0.204	0.00276958	1	Shared with clusters 0, 1, 2, 3, 5, 6, and 7
CHI3L1	-0.0914064	0.095	0.148	7.51E-214	1	Shared with clusters 0, 1, 2, 3, 4, 5, 6, and 7
ITGB7	-0.0960435	0.018	0.098	2.24E-06	1	Shared with clusters 0, 1, 2, and 3
CD300LB	-0.096201	0.032	0.112	0.00234662	1	Shared with clusters 0,1, 2, 3, and 5
PPT1	-0.112821	0.123	0.209	2.07E-05	1	Shared with clusters 0, 1, 2, 3, 6, and 7
MT1E	-0.1141339	0.113	0.129	4.71E-149	1	Shared with clusters 0, 1, 2, 3, 4, 6,and 7
SNX33	-0.1762132	0.008	0.157	1.47E-05	1	
FKBP1A	-0.1780443	0.149	0.263	8.64E-06	1	Shared with clusters 0, 1, 2, 3, 5, and 6
SERPINB2	-0.1816382	0.018	0.099	3.45E-05	1	Shared with clusters 0, 1, 2, and 5
PTGR1	-0.1911361	0.016	0.177	0.00672455	1	Shared with clusters 0, 1, 3, 5, and 6
FABP4	-0.1976732	0.141	0.155	9.44E-11	1	Shared with clusters 0, 1, 2, 3, 4, 5, 6, and 7
RPN2	-0.1999241	0.032	0.196	0.00017548	1	Shared with clusters 0, 1, 2, 3, and 5
TXNRD1	-0.2211929	0.04	0.187	2.70E-33	1	Shared with clusters 0, 1, 3, 4, 5, 6, and 7
PLBD1	-0.2233173	0.067	0.236	2.50E-06	1	Shared with clusters 0, 1, 2, and 6
TAF10	-0.2275387	0.04	0.23	0.00361351	1	Shared with clusters 0, 1, 2, and 6
SEMA4A	-0.2428863	0.02	0.202	0.00876924	1	
ALCAM	-0.2567606	0.053	0.236	1.19E-15	1	Shared with clusters 0, 1, 2, 3, 4 ,5, 6, and 7
PDIA6	-0.2625787	0.198	0.364	6.86E-06	1	Shared with clusters 1, 2, and 6
FABP5	-0.2777559	0.537	0.56	3.76E-84	1	Shared with clusters 0, 1, 2, 3, 4, 5, 6, and 7
MT1G	-0.2999728	0.089	0.205	6.24E-171	1	Shared with clusters 0, 1, 3, 4, 5, 6,and 7
SCPEP1	-0.3273555	0.091	0.305	3.11E-08	1	Shared with clusters 0, 1, 2, and 7
LIPA	-0.3380954	0.376	0.448	8.50E-94	1	Shared with clusters 0, 1, 2, 3, 4, 5, 6, and 7
CD84	-0.3449786	0.048	0.252	1.71E-05	1	Shared with clusters 0, 1, 3, 5, 6, and 7
CALM2	-0.348745	0.582	0.658	0.00089926	1	Shared with clusters 1, 2, and 6
SNRNP48	-0.3619543	0.044	0.311	0.00518088	1	Shared with clusters 1 and 6
HIST1H1E	-0.3843784	0.004	0.257	1.15E-05	1	Shared with clusters 1, 2, 5, and 7
FAM20C	-0.4006923	0.095	0.323	1.29E-16	1	Shared with clusters 0, 1, 2, 3, 4, 5, 6, and 7
COX6C	-0.4128284	0.418	0.58	0.00022926	1	Shared with clusters 0, 1, 2, 5, and 6
S100A9	-0.4241986	0.168	0.35	3.86E-11	1	Shared with clusters 0, 1, 2, 3, 5, 6, and 7
HMG20B	-0.424924	0.234	0.452	4.06E-06	1	Shared with clusters 0, 1, 3, 4, 5, and 7
GUSB	-0.4844126	0.143	0.42	5.55E-16	1	Shared with clusters 0, 1, 2, 3, 5, 6, and 7
PLEKHO1	-0.520913	0.115	0.405	3.77E-07	1	Shared with clusters 1, 2, 5, 6, and 7
DAD1	-0.5637314	0.331	0.599	0.00020952	1	Shared with clusters 1, 2, and 5
FERMT3	-0.5883467	0.145	0.488	6.58E-06	1	Shared with clusters 0, 1, 3, 5, 6, and 7
CFL1	-0.6123958	0.43	0.638	1.39E-12	1	Shared with clusters 0, 1, 2, 3, 5, 6, and 7
CTSL	-0.6487049	0.259	0.475	1.09E-10	1	Shared with clusters 0, 1, 2, 3, 5, 6, and 7
MT1H	-0.6741877	0.065	0.221	1.91E-30	1	Shared with clusters 0, 1, 3, 4, 5, and 7
HLA-DRB1	-0.6947337	0.451	0.488	1.67E-11	1	Shared with clusters 0, 1, and 4
MFSD12	-0.7004685	0.119	0.461	2.39E-49	1	Shared with clusters 0, 1, 2, 3,4, 5, 6, and 7
MGAT1	-0.7683445	0.863	0.919	0.00763655	1	Shared with clusters 1 and 5
CAPG	-0.7872442	0.301	0.592	1.78E-11	1	Shared with clusters 0, 1, 2, 3, 5, 6, and 7
TGFB1	-0.7994803	0.2	0.586	0.00018394	1	Shared with clusters 0, 1, 2, 3, 5, and 6
IQGAP2	-0.7998243	0.115	0.458	0.00867603	1	Shared with clusters 0, 1, and 2
TMEM59	-0.9207186	0.56	0.811	0.00074734	1	Shared with clusters 1 and 2
PRDX1	-1.0187566	0.471	0.737	4.86E-10	1	Shared with clusters 0, 1, 2, 3, 5, 6, and 7
ARPC1B	-1.0740162	0.257	0.625	9.24E-25	1	Shared with clusters 0, 1, 2, 3, 5, 6, and 7
IL1RN	-1.1133637	0.333	0.62	0.00174718	1	Shared with clusters 0, 1, 3, 4, 5 6, and 7
ATP6AP2	-1.1432955	0.347	0.728	9.92E-05	1	Shared with clusters 0, 1, 2, 3, 5, and 7
TUBA1B	-1.1681292	0.352	0.731	6.41E-11	1	Shared with clusters 0, 1, 3, 5, 6, and 7
GSN	-1.1819571	0.166	0.663	7.12E-19	1	Shared with clusters 0, 1, 2, 3, 5, 6, and 7
CYP1B1	-1.1922573	0.143	0.525	4.56E-16	1	Shared with clusters 0, 1, 2, 3, 5, and 7
RGCC	-1.2962967	0.598	0.723	2.67E-11	1	Shared with clusters 0, 1, 5, 6, and 7
FBP1	-1.2977907	0.275	0.718	1.69E-34	1	Shared with clusters 0, 1, 2, 3, 5, 6, and 7
SOCS3	-1.3312549	0.23	0.604	0.00071396	1	Shared with clusters 1, 2, and 5

LIMS1	-1.352952	0.343	0.75	0.0004252	1	Shared with clusters 1, 5, and 7
ANXA2	-1.3662164	0.513	0.84	3.68E-07	1	Shared with clusters 0, 1, 2, 3, 5, 6, and 7
CD81	-1.6038962	0.111	0.723	8.66E-10	1	Shared with clusters 0, 1, 2, 5, and 6
MT1X	-1.6996977	0.402	0.488	9.10E-236	1	Shared with clusters 0, 1, 3, 4, 5, 6,and 7
FLNA	-1.7490596	0.337	0.791	1.45E-07	1	Shared with clusters 1, 3, 5, and 6
CTSZ	-1.9685816	0.24	0.791	0.00433635	1	Shared with clusters 0, 1, 2, 3, 6, and 7
ANXA5	-1.9989728	0.408	0.877	0.00235762	1	Shared with clusters 1, 5, 6, and 7
MT2A	-2.2123231	0.699	0.72	1.99E-160	1	Shared with clusters 0, 2, 1, 3, 4, 5, 6, and 7
CD9	-2.2581959	0.79	0.957	7.08E-09	1	Shared with clusters 1, 2, 5, and 7
MARCO	-2.5095706	0.133	0.618	0.00124919	1	Shared with clusters 0,1, 2, and 7
SERF2	-2.9821355	0.996	0.999	0.0014424	1	Shared with clusters 1, 2, and 6
TMBIM6	-2.9828539	0.545	0.96	1.33E-05	1	Shared with clusters 1 and 7
CD63	-3.1882639	0.919	0.992	2.98E-14	1	Shared with clusters 0, 1, 2, 3, 4, 5, 6, and 7
ACTB	-3.7222212	0.76	0.98	6.59E-11	1	Shared with clusters 0, 1, 3, 5, 6, and 7
FN1	-4.2569348	0.139	0.334	2.88E-11	1	Shared with clusters 1, 3, and 7
S100A11	-4.9212271	0.99	0.996	3.73E-08	1	Shared with clusters 1, 5, 6, and 7
EGR1	-5.3108307	0.382	0.894	9.88E-05	1	Shared with clusters 1, 2, 5, and 6
MMP9	-5.3250848	0.57	0.92	1.32E-20	1	Shared with clusters 0, 1, 2, 3, 4, 5, 6, and 7
LGALS3	-5.3777094	0.889	0.99	1.28E-18	1	Shared with clusters 0, 1, 2, 3, 4, 5, 6, and 7
HLA-B	-7.2450956	0.988	0.997	4.91E-06	1	Shared with clusters 1 and 2
RPSA	-8.6846631	0.701	0.997	3.11E-05	1	Shared with clusters 0, 1, 5, and 6
RPS5	-10.833262	0.962	1	0.0099189	1	Shared with clusters 0, 1, and 5
S100A6	-10.99954	1	1	1.58E-06	1	Shared with clusters 1, 2, 5, and 6
CSTB	-12.991086	1	1	9.71E-10	1	Shared with clusters 0, 1, 5, 6, and 7
B2M	-13.807166	1	1	1.43E-08	1	Shared with clusters 1 and 2
EREG	-15.456567	0.564	0.934	0.00016034	1	Shared with clusters 1, 2, 5, 6, and 7
FOS	-15.701411	0.966	0.995	0.00804806	1	Shared with clusters 1, 5, and 6
SPP1	-16.303196	0.937	0.996	4.83E-77	1	Shared with clusters 0, 1, 2, 3, 4, 5, 6, and 7
RPL3	-17.008675	0.984	1	0.00488935	1	Shared with clusters 0, 1, and 4
RPL32	-19.793583	1	1	1.27E-05	1	Shared with clusters 0, 1, and 4
RPS24	-22.272288	0.978	1	0.00059108	1	Shared with clusters 0, 1, 2, and 6
MT-CO1	-23.638944	0.905	0.996	1.20E-06	1	Shared with clusters 0, 1, 2, 3, and 5
RPL7A	-24.642619	0.97	1	1.18E-05	1	Shared with clusters 0, 1, 4, 5, 6, and 7
RPS18	-26.595361	1	1	0.00068768	1	Shared with clusters 0, 1, 4, and 6
RPS3A	-31.699084	1	1	0.00139598	1	Shared with clusters 0, 1, and 6
RPL13	-32.560711	1	1	1.36E-06	1	Shared with clusters 0, 1, 4, 5, and 6
TPT1	-55.824408	0.998	1	0.00055788	1	Shared with clusters 1, 6, and 7
RPL17	2.30932662	0.899	0.681	0.00669952	2	Shared with clusters 2 and 6
IL8	1.87914425	0.926	0.814	4.06E-16	2	Shared with clusters 0, 2, 4, 5, and 6
OTOA	0.09203699	0.201	0.128	0.00052662	2	
CLK1	0.0264865	0.473	0.447	5.00E-18	2	Shared with clusters 0 and 2
EFNA5	-0.0273071	0.05	0.07	1.81E-10	2	
IRAK2	-0.030319	0.024	0.052	5.43E-05	2	
CLIP2	-0.0316509	0.015	0.046	0.00669255	2	
PDE2A	-0.0330971	0.045	0.073	2.52E-05	2	
GREM1	-0.0342986	0.031	0.036	0.00022855	2	Shared with clusters 2 and 5
HIVEP2	-0.0362572	0.021	0.056	1.63E-07	2	
VENTX	-0.0395218	0.002	0.038	1.79E-18	2	Shared with clusters 0, 2, and 6
RP11-138A9	-0.0426862	0.132	0.166	0.00301552	2	
CCDC84	-0.0454084	0.021	0.066	0.00460057	2	
PLK2	-0.0467404	0.014	0.053	0.00097045	2	
LTB	-0.0482205	0.01	0.058	5.98E-16	2	Shared with clusters 2, 6, and 7
PHF2	-0.0486473	0.015	0.062	0.00622677	2	
CSF1	-0.0506235	0.003	0.043	3.01E-14	2	Shared with clusters 2 and 5
FAM115C	-0.0521051	0.055	0.098	0.00435722	2	Shared with clusters 2 and 7
DLEU7	-0.052226	0.017	0.063	0.00784848	2	
HIST1H2BK	-0.055635	0.173	0.194	0.00020216	2	
CLCN6	-0.059976	0.039	0.099	0.00042546	2	Shared with clusters 0, 2, and 7
DGKD	-0.0609247	0.026	0.081	0.00462621	2	Shared with clusters 2 and 7
OLFML2B	-0.0615173	0.043	0.093	9.96E-13	2	
METTL2B	-0.0619264	0.041	0.097	3.02E-05	2	
CD93	-0.0661398	0.007	0.065	1.19E-38	2	Shared with clusters 0, 2, 5, and 6
LRRK2	-0.0676729	0.033	0.093	6.16E-17	2	
AC079767.4	-0.0716913	0.358	0.415	3.30E-11	2	Shared with clusters 2 and 5

MS4A6A	-0.0736738	0.082	0.128	0.00234652	2	
NFXL1	-0.0752652	0.021	0.085	7.34E-35	2	Shared with clusters 0, 2, 5, 6, and 7
GOLGA8A	-0.0770159	0.038	0.107	1.25E-06	2	Shared with clusters 2, 6, and 7
IL7R	-0.0772156	0.021	0.07	5.85E-14	2	Shared with clusters 0, 2, 3, 6, and 7
DGKE	-0.0797587	0.039	0.111	0.00055344	2	Shared with clusters 2 and 5
ANGPTL4	-0.0805076	0.041	0.088	0.00974918	2	Shared with clusters 2 and 5
CFP	-0.0831538	0.017	0.089	9.92E-05	2	
NR4A3	-0.0837899	0.034	0.086	0.00048547	2	Shared with clusters 2, 5, 6, and 7
MS4A14	-0.089754	0.021	0.104	3.99E-05	2	
SMOX	-0.0919424	0.111	0.189	0.00022702	2	
HIP1	-0.0962236	0.029	0.111	2.18E-05	2	Shared with clusters 2 and 6
TM4SF19.1	-0.0981401	0.062	0.117	1.88E-26	2	Shared with clusters 2, 4, and 5
PREPL	-0.0998472	0.082	0.17	0.00501684	2	
SEMA4D	-0.1038512	0.036	0.128	9.69E-13	2	Shared with clusters 2 and 5
TNIK	-0.1092757	0.031	0.127	0.00030854	2	
TNFRSF10B	-0.1112873	0.039	0.14	0.00215238	2	
DNAJB2	-0.1119277	0.204	0.29	0.00020698	2	
TANC2	-0.1124888	0.036	0.133	5.06E-06	2	Shared with clusters 2 and 5
TPRA1	-0.1139598	0.286	0.347	0.00212733	2	
SLA	-0.1179567	0.026	0.122	2.61E-09	2	Shared with clusters 2 and 5
IL3RA	-0.1212486	0.051	0.128	5.83E-05	2	
ANKZF1	-0.1231732	0.063	0.167	2.63E-06	2	
HBEGF	-0.1267246	0.019	0.117	0.00213264	2	Shared with clusters 2 and 5
SH3D21	-0.1275104	0.099	0.202	0.00305063	2	
SLC25A37	-0.12886	0.096	0.188	1.05E-28	2	Shared with clusters 2 and 5
LUC7L	-0.1321424	0.094	0.211	5.36E-05	2	
CYCS	-0.1337028	0.16	0.233	2.80E-06	2	Shared with clusters 2 and 5
DGKA	-0.1353717	0.055	0.177	0.00039788	2	
CD101	-0.136285	0.017	0.135	1.33E-08	2	Shared with clusters 2 and 5
FAM193B	-0.1363381	0.051	0.174	0.00332939	2	Shared with clusters 2 and 5
ULK1	-0.1384013	0.036	0.162	0.00322914	2	Shared with clusters 2 and 5
LINC00926	-0.139002	0.033	0.145	1.47E-06	2	
TLR2	-0.1404821	0.029	0.151	2.44E-22	2	
LACC1	-0.141318	0.031	0.147	0.00567891	2	Shared with clusters 2 and 7
THBD	-0.1417964	0.01	0.105	3.26E-21	2	Shared with clusters 0, 2, and 5
SECISBP2	-0.1440527	0.053	0.182	0.00018606	2	Shared with clusters 0 and 2
SIK1	-0.1487731	0.091	0.201	0.00574512	2	Shared with clusters 2 and 6
REV3L	-0.150271	0.101	0.207	2.81E-20	2	Shared with clusters 0, 2, and 5
ENTPD1	-0.1516192	0.051	0.181	0.00055552	2	
HAUS6	-0.1551979	0.053	0.188	1.41E-07	2	Shared with clusters 2, 5, and 6
CCNT2	-0.1556165	0.082	0.215	0.00522599	2	
IFRD1	-0.1570957	0.304	0.361	0.00011639	2	Shared with clusters 2 and 6
MSANTD3	-0.1577409	0.069	0.197	0.00205272	2	Shared with clusters 2 and 5
HIST1H1D	-0.1582967	0.021	0.134	3.05E-19	2	Shared with clusters 2 and 3
POM121C	-0.1589682	0.06	0.2	0.00099668	2	
GAB2	-0.1602486	0.07	0.211	0.00210232	2	Shared with clusters 2 and 5
ZBTB43	-0.1618974	0.17	0.281	7.40E-10	2	
BANP	-0.1629154	0.158	0.272	0.00035014	2	
RALGDS	-0.1639224	0.05	0.193	5.13E-07	2	Shared with clusters 2 and 5
CASC7	-0.1642201	0.111	0.241	0.00183644	2	
TAF1	-0.1657858	0.055	0.195	0.00033069	2	Shared with clusters 2 and 5
CSRNP1	-0.1676153	0.091	0.207	0.00127939	2	
PFKFB3	-0.169699	0.172	0.257	0.0043988	2	
RANBP2	-0.1709058	0.06	0.198	1.72E-09	2	
CYTL1	-0.1712102	0.045	0.153	5.92E-05	2	
USP37	-0.1713584	0.053	0.203	0.00229907	2	
PTGS2	-0.1739611	0.027	0.11	0.00092368	2	Shared with clusters 2 and 5
KIAA1147	-0.1748582	0.038	0.185	4.55E-09	2	Shared with clusters 2 and 5
C9orf72	-0.1766662	0.213	0.327	1.41E-07	2	Shared with clusters 2 and 5
SRSF1	-0.1770562	0.166	0.306	0.00041059	2	
ODC1	-0.1802745	0.053	0.202	0.00037199	2	
TOB1	-0.1829619	0.232	0.338	0.00634227	2	
SNAPC1	-0.1838737	0.137	0.261	0.00088658	2	Shared with clusters 2 and 5
VMO1	-0.193078	0.153	0.203	6.36E-08	2	Shared with clusters 2, 4, and 7

MMP14	-0.1936406	0.072	0.232	0.00579223	2	Shared with clusters 0 and 2
ZNF326	-0.194172	0.087	0.245	0.00951052	2	
HIST1H1E	-0.1954591	0.026	0.181	3.04E-16	2	Shared with clusters 1, 2, 5, and 7
SERPINB2	-0.1959986	0.017	0.079	1.47E-09	2	Shared with clusters 0, 1, 2, and 5
LPCAT1	-0.1969855	0.113	0.258	5.96E-13	2	Shared with clusters 0 and 2
NAP1L4	-0.1983433	0.108	0.275	0.00246841	2	Shared with clusters 2 and 7
SAFB2	-0.2009925	0.175	0.332	6.48E-07	2	
SLC16A10	-0.2019994	0.072	0.161	7.57E-11	2	Shared with clusters 0, 2, and 7
PTPLAD2	-0.2056097	0.238	0.374	6.28E-05	2	
POGZ	-0.2062401	0.084	0.249	0.00513726	2	Shared with clusters 0 and 2
EDEM1	-0.2088605	0.07	0.249	0.00082898	2	
FCAR	-0.2094259	0.046	0.206	1.50E-18	2	Shared with clusters 2, 5, and 6
TREM1	-0.2139289	0.096	0.209	0.00072128	2	
PDPK1	-0.2144345	0.122	0.289	0.00485258	2	Shared with clusters 2, 5, 6, and 7
NLRP1	-0.2161049	0.067	0.241	5.58E-08	2	
CREBBP	-0.2166798	0.079	0.256	0.00189499	2	
ETS2	-0.21897	0.082	0.215	1.02E-14	2	Shared with clusters 0, 2, and 5
STARD10	-0.2225487	0.086	0.263	0.00162826	2	Shared with clusters 2, 5, and 7
KDM4B	-0.2230801	0.098	0.286	1.98E-05	2	
SFXN1	-0.2254913	0.072	0.255	0.00162228	2	
PTP4A1	-0.2267066	0.232	0.363	2.94E-05	2	
PLK3	-0.2272543	0.197	0.329	1.58E-05	2	
AVL9	-0.2278345	0.11	0.294	0.00191277	2	
MAP2K3	-0.2307704	0.163	0.314	0.00024556	2	Shared with clusters 2 and 5
CHML	-0.2328565	0.036	0.207	9.68E-11	2	Shared with clusters 0, 2, 5, and 7
TMEM167B	-0.2337933	0.218	0.377	0.00829476	2	
PTK2B	-0.2338095	0.17	0.324	1.04E-07	2	Shared with clusters 2 and 5
P2RX7	-0.234372	0.051	0.246	0.00259453	2	Shared with clusters 2, 5, 6, and 7
SLC36A4	-0.2364218	0.18	0.33	3.43E-07	2	Shared with clusters 0, 2, and 5
LPAR2	-0.2369232	0.051	0.253	0.0059142	2	Shared with clusters 2, 6, and 7
VPS8	-0.2385108	0.149	0.33	0.00479735	2	
WDR45B	-0.2413597	0.204	0.363	0.00023432	2	
GGA2	-0.2420188	0.118	0.296	1.95E-05	2	Shared with clusters 2 and 5
VAMP2	-0.2431472	0.28	0.421	0.0020169	2	Shared with clusters 2 and 5
SESTD1	-0.2461806	0.113	0.295	8.73E-09	2	Shared with clusters 0, 2, and 5
MLLT6	-0.2479135	0.117	0.294	2.52E-15	2	Shared with clusters 0 and 2
H1F0	-0.2485577	0.098	0.231	1.08E-13	2	Shared with clusters 2, 5, and 7
FUT11	-0.2493922	0.065	0.257	2.72E-05	2	Shared with clusters 2 and 5
CSF2RA	-0.2500503	0.214	0.345	7.81E-06	2	
ITPR1	-0.2519529	0.093	0.261	4.17E-19	2	Shared with clusters 0 and 2
ZNF160	-0.2525986	0.134	0.33	2.73E-09	2	Shared with clusters 2, 5, and 7
ZNF281	-0.2551077	0.184	0.35	0.00363664	2	
MED13L	-0.2563063	0.084	0.289	0.00200627	2	
DOCK4	-0.2568201	0.089	0.278	6.42E-12	2	Shared with clusters 2 and 5
DDI2	-0.2571001	0.136	0.316	0.00149405	2	Shared with clusters 2 and 6
FNBP4	-0.2572579	0.168	0.35	0.00821621	2	
ZBTB25	-0.2595127	0.153	0.332	2.10E-06	2	Shared with clusters 2, 5, and 6
MXD1	-0.2604614	0.148	0.314	5.65E-09	2	Shared with clusters 2 and 5
CMIP	-0.2607386	0.099	0.291	0.00012554	2	
THBS1	-0.2615214	0.051	0.126	9.26E-75	2	Shared with clusters 0, 2, 5, and 7
SLC8A1	-0.2616165	0.045	0.248	0.00445035	2	
ATXN2L	-0.2632993	0.122	0.326	1.31E-05	2	Shared with clusters 0 and 2
G3BP1	-0.2636118	0.094	0.287	1.28E-05	2	
RLF	-0.2651355	0.093	0.295	2.36E-11	2	Shared with clusters 0, 2, and 5
LY6E	-0.2658547	0.22	0.363	1.74E-05	2	
GAL	-0.2678864	0.038	0.083	0.00042589	2	
SRSF3	-0.2710674	0.403	0.527	0.00011121	2	
TRIM25	-0.2726761	0.099	0.295	4.62E-12	2	Shared with clusters 2 and 5
KIAA1551	-0.2729194	0.079	0.281	2.69E-10	2	Shared with clusters 0, 2, 5, and 7
IVNS1ABP	-0.2792519	0.158	0.343	7.68E-10	2	Shared with clusters 2, 5, and 7
ZNF516	-0.2793456	0.091	0.308	2.46E-05	2	Shared with clusters 2, 5, and 7
YEATS2	-0.2802072	0.079	0.301	0.0002315	2	
FAIM	-0.2802603	0.103	0.295	1.75E-06	2	Shared with clusters 2 and 5
NUP62	-0.2818384	0.178	0.358	3.44E-06	2	

HIST1H2AC	-0.282594	0.11	0.282	0.00953576	2	Shared with clusters 2 and 5
IRF1	-0.2838787	0.249	0.382	0.00155508	2	Shared with clusters 2 and 6
PHF20L1	-0.2839178	0.127	0.327	0.00038168	2	
DENND5A	-0.2906919	0.123	0.33	3.79E-07	2	
NPHP3	-0.2913715	0.139	0.345	0.00060536	2	Shared with clusters 2, 5, and 7
KDM7A	-0.2916583	0.113	0.325	5.68E-08	2	Shared with clusters 2 and 5
DFNA5	-0.2926213	0.412	0.538	0.00937748	2	Shared with clusters 0, 2, and 7
RICTOR	-0.2926739	0.262	0.429	3.11E-05	2	
TMEM170B	-0.2935461	0.06	0.264	3.41E-15	2	Shared with clusters 0, 2, and 5
NGLY1	-0.2937052	0.137	0.346	0.00689644	2	
CHMP1B	-0.2949846	0.358	0.444	0.00013076	2	
HIST1H1C	-0.2959247	0.105	0.263	2.18E-11	2	Shared with clusters 2 and 5
SH3BP2	-0.2966301	0.117	0.304	7.16E-13	2	Shared with clusters 2 and 7
PIK3R5	-0.2978397	0.094	0.313	0.00174161	2	Shared with clusters 0 and 2
SLC2A1	-0.2979841	0.226	0.342	8.32E-08	2	
NUPL1	-0.2994776	0.125	0.327	2.87E-07	2	Shared with clusters 0 and 2
EXOC3	-0.3011479	0.072	0.302	1.14E-08	2	Shared with clusters 0, 2, 5, and 7
RYBP	-0.3023332	0.113	0.323	2.92E-16	2	Shared with clusters 0, 2, 3, and 5
MDM4	-0.3026883	0.293	0.458	1.07E-06	2	
NF1	-0.3029244	0.086	0.311	1.82E-05	2	Shared with clusters 2, 5, and 7
SBF2	-0.3060127	0.278	0.443	7.28E-05	2	
ST8SIA4	-0.3061632	0.079	0.295	2.15E-08	2	Shared with clusters 2 and 5
ZBTB4	-0.3076176	0.082	0.323	0.00063602	2	Shared with clusters 2 and 5
PHC3	-0.3083325	0.118	0.345	5.70E-05	2	Shared with clusters 2 and 5
DENND3	-0.3097338	0.099	0.311	1.66E-13	2	Shared with clusters 2, 5, and 6
CERK	-0.3116407	0.101	0.325	1.11E-08	2	
ELL2	-0.3134282	0.173	0.35	9.21E-12	2	
MYO1G	-0.315358	0.072	0.274	3.67E-11	2	Shared with clusters 2 and 5
NUMA1	-0.3162388	0.127	0.367	5.17E-05	2	
VNN 1.00	-0.3172023	0.015	0.199	4.18E-11	2	Shared with clusters 0, 2, and 5
LIMD1	-0.3178317	0.106	0.33	6.62E-05	2	Shared with clusters 2 and 7
COLEC12	-0.3207213	0.058	0.25	2.82E-07	2	
U2AF1	-0.3208589	0.273	0.477	0.00149866	2	
ST3GAL1	-0.3222736	0.142	0.323	9.92E-31	2	Shared with clusters 0, 2, and 5
CLEC12A	-0.3231353	0.113	0.323	2.03E-10	2	
SGK3	-0.3288302	0.16	0.37	5.09E-06	2	Shared with clusters 2 and 5
SP3	-0.3300059	0.161	0.389	0.00093591	2	Shared with clusters 0, 2, and 5
PKN2	-0.3319998	0.165	0.392	1.35E-05	2	Shared with clusters 2 and 5
TUBA4A	-0.3336118	0.196	0.391	1.00E-07	2	Shared with clusters 2 and 5
PDK1	-0.3361438	0.142	0.337	2.84E-22	2	Shared with clusters 2 and 5
PPP1R10	-0.3396179	0.127	0.373	4.02E-09	2	Shared with clusters 2 and 6
RBM5	-0.341465	0.18	0.406	1.49E-05	2	
CLEC5A	-0.3459188	0.024	0.2	1.10E-23	2	Shared with clusters 0 and 2
PCNX	-0.3473134	0.082	0.334	1.55E-06	2	Shared with clusters 0, 2, and 5
HNRNPU-AS	-0.3508405	0.106	0.323	2.05E-11	2	Shared with clusters 2 and 5
HELZ	-0.3540454	0.13	0.38	0.00078518	2	Shared with clusters 2 and 5
ACTN1	-0.3544899	0.144	0.38	0.00940905	2	Shared with clusters 2 and 5
RBM33	-0.3552373	0.096	0.363	9.15E-07	2	
HNRNPH1	-0.3568298	0.413	0.604	0.0054916	2	
PER1	-0.3580346	0.165	0.365	1.49E-07	2	Shared with clusters 2, 5, and 6
KLHL24	-0.3609433	0.196	0.404	6.40E-08	2	Shared with clusters 2 and 5
CCNG2	-0.3615971	0.197	0.419	1.78E-07	2	Shared with clusters 2 and 5
ZMYM2	-0.3693146	0.302	0.494	0.0001227	2	Shared with clusters 2 and 7
AHRR	-0.3696295	0.069	0.33	0.00577027	2	Shared with clusters 2, 5, and 7
KLF10	-0.369706	0.264	0.434	7.44E-07	2	
ASH1L	-0.3818825	0.137	0.398	1.40E-06	2	Shared with clusters 2, 5, and 7
NBPF10	-0.3824498	0.377	0.528	2.21E-12	2	Shared with clusters 0, 2, and 5
PCF11	-0.3830936	0.16	0.397	0.00059366	2	Shared with clusters 0 and 2
AKAP10	-0.3880912	0.151	0.407	3.49E-09	2	Shared with clusters 0, 2, and 5
CDH23	-0.3890236	0.168	0.387	4.16E-07	2	Shared with clusters 2, 6, and 7
ENO2	-0.3918902	0.233	0.445	9.43E-06	2	
LGALS8	-0.3923658	0.132	0.405	0.00400542	2	Shared with clusters 2 and 7
ATP13A3	-0.3948041	0.146	0.364	2.85E-14	2	Shared with clusters 2 and 7
MTURN	-0.3986328	0.051	0.365	4.56E-05	2	

TET2	-0.3990691	0.094	0.367	9.32E-12	2	Shared with clusters 2, 5, 6, and 7
MGLL	-0.4014138	0.153	0.287	8.69E-06	2	Shared with clusters 2, 3, 4, 5, 6, and 7
BBX	-0.4061749	0.117	0.384	0.00410747	2	Shared with clusters 2 and 5
FOSL2	-0.4081965	0.331	0.47	1.12E-30	2	Shared with clusters 2, 5, and 7
P2RX4	-0.4129289	0.295	0.532	0.00273795	2	
UQCRQ	-0.4142083	0.453	0.629	4.03E-07	2	
CELF2	-0.4303831	0.19	0.426	0.00208991	2	Shared with clusters 2 and 5
RCSD1	-0.4333462	0.261	0.519	0.00030714	2	
KDM2A	-0.433524	0.117	0.424	0.00740984	2	
KMT2C	-0.4367548	0.137	0.418	7.11E-10	2	Shared with clusters 0 and 2
HNRNPA3	-0.4398727	0.153	0.448	0.00043321	2	
ZNF267	-0.4401447	0.214	0.471	0.00018864	2	
FNDC3B	-0.4436335	0.134	0.396	0.00280806	2	Shared with clusters 2 and 7
CD55	-0.4495459	0.134	0.395	0.00016546	2	Shared with clusters 2 and 5
ARL8B	-0.4551567	0.244	0.505	0.00017924	2	
SLC43A2	-0.4552843	0.149	0.347	1.84E-10	2	Shared with clusters 0 and 2
CTB-61M7.2	-0.4607264	0.144	0.338	5.17E-33	2	Shared with clusters 0, 2, 5, and 7
SPEN	-0.4607589	0.081	0.398	3.59E-08	2	Shared with clusters 0 and 2
SLC43A3	-0.4678881	0.285	0.495	4.94E-10	2	Shared with clusters 2 and 7
TMEM30A	-0.4744189	0.31	0.518	5.49E-05	2	
ALOX5	-0.4773801	0.048	0.361	4.41E-08	2	Shared with clusters 2 and 5
CEP170	-0.4795604	0.16	0.461	0.00021237	2	Shared with clusters 2 and 7
INSIG1	-0.480749	0.37	0.498	0.0075534	2	Shared with clusters 2 and 5
MARCH2	-0.4828753	0.084	0.399	1.97E-13	2	Shared with clusters 0, 2, 3, 5, and 7
YME1L1	-0.4837494	0.196	0.479	0.00015765	2	Shared with clusters 0 and 2
ZNF331	-0.4846192	0.153	0.366	1.36E-11	2	Shared with clusters 2, 4, and 5
PRKAG2	-0.4914005	0.358	0.55	2.95E-07	2	
TDP2	-0.49443	0.352	0.559	2.08E-10	2	Shared with clusters 2 and 7
NKTR	-0.5051169	0.204	0.471	2.70E-13	2	Shared with clusters 2 and 5
ADAM28	-0.507354	0.165	0.402	3.82E-06	2	Shared with clusters 2, 5, and 7
LPP	-0.5091983	0.129	0.424	4.32E-14	2	Shared with clusters 2 and 7
FAM63B	-0.5118489	0.237	0.505	0.0006595	2	Shared with clusters 2 and 6
PINK1	-0.5210571	0.115	0.469	1.73E-06	2	Shared with clusters 2 and 7
SYNGR1	-0.5329879	0.163	0.395	3.55E-13	2	Shared with clusters 0, 2, and 7
HNRNPD	-0.5340766	0.196	0.519	0.00274024	2	
SF1	-0.5357933	0.237	0.53	8.07E-07	2	Shared with clusters 2 and 5
ADM	-0.5384659	0.611	0.655	0.00265782	2	Shared with clusters 2 and 4
PTBP3	-0.5390117	0.134	0.467	0.00123841	2	
PHIP	-0.5397892	0.214	0.498	1.59E-10	2	Shared with clusters 0, 2, and 5
STX7	-0.5452165	0.307	0.581	0.00022989	2	Shared with clusters 2, 5, and 6
RAMP1	-0.5473462	0.148	0.321	7.80E-10	2	Shared with clusters 1, 2, and 6
MACF1	-0.5488072	0.127	0.447	5.21E-07	2	
MDM2	-0.5489759	0.232	0.443	2.00E-13	2	Shared with clusters 2, 3, and 5
GPC4	-0.5500794	0.142	0.34	5.47E-08	2	Shared with clusters 2, 5, and 6
TRA2B	-0.5526664	0.4	0.595	5.62E-06	2	Shared with clusters 2 and 5
TUBGCP2	-0.5549958	0.312	0.565	1.86E-05	2	
SRSF11	-0.5560845	0.345	0.585	1.53E-07	2	
AKAP13	-0.5574638	0.187	0.514	0.00311043	2	
RNMT	-0.5580837	0.24	0.54	6.65E-06	2	
ZNF638	-0.5591967	0.199	0.519	1.26E-10	2	Shared with clusters 2 and 5
FNIP2	-0.5593315	0.3	0.509	0.00413657	2	Shared with clusters 2, 5, and 7
ABCC3	-0.5627429	0.244	0.487	1.82E-05	2	Shared with clusters 2, 6, and 7
FMNL1	-0.5630378	0.23	0.51	0.00175336	2	
LUC7L3	-0.5708734	0.187	0.505	3.56E-07	2	Shared with clusters 2 and 5
FNDC3A	-0.578804	0.149	0.429	5.16E-11	2	Shared with clusters 0, 2, 5, and 7
SOCS3	-0.5794879	0.244	0.402	1.26E-05	2	Shared with clusters 1, 2, and 5
ABHD5	-0.5798889	0.283	0.529	0.00033039	2	Shared with clusters 2, 5, and 6
SFPQ	-0.5899234	0.163	0.47	0.00011096	2	Shared with clusters 0 and 2
BRD2	-0.5941071	0.286	0.543	5.91E-18	2	Shared with clusters 0, 2, and 5
NUMB	-0.5959529	0.293	0.587	0.00013293	2	Shared with clusters 2 and 5
IRAK3	-0.5959825	0.129	0.475	8.99E-20	2	Shared with clusters 2 and 5
CDKN1A	-0.5970798	0.319	0.461	0.00510639	2	Shared with clusters 2 and 7
GCLC	-0.5977604	0.16	0.377	2.48E-08	2	Shared with clusters 1, 2, 3, 4, 5, 6, and 7
GPCPD1	-0.5986168	0.273	0.534	6.33E-08	2	Shared with clusters 2 and 5

GPATCH2L	-0.5993886	0.172	0.504	0.00099829	2	
SLC38A2	-0.6080142	0.321	0.573	0.00015155	2	
CD300LB	-0.608582	0.132	0.463	6.24E-10	2	Shared with clusters 0,1, 2, 3, and 5
HLA-DMB	-0.6090145	0.252	0.484	9.71E-06	2	Shared with clusters 2 and 6
GOLGA4	-0.6093213	0.111	0.468	2.73E-13	2	
JARID2	-0.6154134	0.262	0.531	0.0018297	2	Shared with clusters 0 and 2
TRIM38	-0.618073	0.142	0.506	9.78E-06	2	Shared with clusters 0, 2, and 5
CXCR4	-0.6192148	0.566	0.686	1.78E-15	2	Shared with clusters 0, 2, 3, and 5
FUS	-0.6199996	0.422	0.633	1.12E-18	2	Shared with clusters 0, 2, and 5
ATF4	-0.6217014	0.497	0.668	0.00338539	2	
SMCHD1	-0.6250565	0.151	0.532	0.00297691	2	Shared with clusters 0, 2, and 5
NAMPT	-0.6252089	0.264	0.471	4.15E-21	2	Shared with clusters 0, 2, and 5
HP1BP3	-0.6256778	0.232	0.565	0.00028385	2	
PTPN12	-0.6275316	0.218	0.537	9.75E-05	2	Shared with clusters 0, 2, and 7
GPAA1	-0.6283756	0.209	0.559	1.86E-08	2	Shared with clusters 2 and 7
PIK3CB	-0.644755	0.244	0.546	4.88E-06	2	
C4orf48	-0.6468607	0.377	0.615	2.12E-06	2	Shared with clusters 2 and 7
NR4A2	-0.6502597	0.268	0.411	0.00445364	2	
S100A8	-0.6528778	0.154	0.312	3.58E-09	2	Shared with clusters 1, 2, and 6
DYNLT1	-0.6545003	0.314	0.636	0.00116482	2	
AMICA1	-0.6582162	0.235	0.521	9.54E-06	2	Shared with clusters 2 and 5
SPAG9	-0.6607062	0.233	0.549	8.69E-12	2	Shared with clusters 0 and 2
C1QB	-0.6702297	0.093	0.185	2.92E-10	2	Shared with clusters 1, 2, 5, and 6
CREBRF	-0.6717276	0.172	0.515	2.11E-14	2	Shared with clusters 2 and 5
TNS1	-0.6755008	0.252	0.563	7.52E-07	2	Shared with clusters 0, 2, and 7
HIPK2	-0.6922061	0.142	0.518	5.26E-05	2	Shared with clusters 0, 2, and 7
RIOK3	-0.6964658	0.214	0.573	1.91E-07	2	
ADAM8	-0.6992741	0.295	0.559	6.29E-05	2	
NDUFAB1	-0.7074002	0.163	0.605	2.41E-08	2	Shared with clusters 2 and 5
ATRX	-0.7132691	0.168	0.546	0.00742924	2	
CD59	-0.7240735	0.345	0.567	3.03E-06	2	Shared with clusters 0, 1, 2, 3, 5, 6, and 7
C1orf162	-0.7259833	0.357	0.622	2.34E-05	2	
MGEA5	-0.7296705	0.321	0.6	2.01E-19	2	Shared with clusters 2 and 5
LST1	-0.7296948	0.254	0.536	1.91E-06	2	
OAS1	-0.7314957	0.213	0.457	4.97E-15	2	Shared with clusters 0, 2, and 3
SPARC	-0.7321045	0.199	0.385	6.53E-16	2	Shared with clusters 0, 2, and 3
CD109	-0.7324023	0.249	0.538	5.08E-13	2	Shared with clusters 2 and 7
IER5	-0.7325977	0.123	0.485	6.22E-19	2	Shared with clusters 2 and 5
NDUFB2	-0.7355312	0.346	0.636	0.0009374	2	Shared with clusters 0, 2, 5, and 6
IGSF6	-0.7374678	0.178	0.468	2.20E-05	2	Shared with clusters 0, 2, 5, and 6
ITGB7	-0.7384504	0.158	0.527	2.57E-10	2	Shared with clusters 0, 1, 2, and 3
TAF10	-0.7603737	0.122	0.572	5.16E-09	2	Shared with clusters 0, 1, 2, and 6
SOD1	-0.7665365	0.336	0.661	6.93E-10	2	Shared with clusters 2 and 5
C1orf54	-0.7669661	0.357	0.604	1.25E-08	2	Shared with clusters 2 and 7
ZNF207	-0.7773691	0.357	0.654	2.60E-10	2	Shared with clusters 0 and 2
PHF3	-0.7821598	0.151	0.576	0.00317105	2	Shared with clusters 2 and 5
GLIPR2	-0.7962495	0.388	0.66	0.0033799	2	
BTG2	-0.8120983	0.216	0.488	3.38E-13	2	Shared with clusters 2, 5, and 6
GLTSCR2	-0.8177412	0.581	0.762	3.78E-07	2	
G6PD	-0.8330648	0.202	0.458	0.00067852	2	Shared with clusters 0, 1, 2, 3, 4, 5, 6, and 7
SORL1	-0.8364136	0.12	0.451	5.78E-35	2	Shared with clusters 2 and 5
GLA	-0.8375066	0.271	0.586	3.43E-05	2	Shared with clusters 0, 2, 5, and 7
AMFR	-0.8441937	0.235	0.562	9.41E-05	2	
PDE4DIP	-0.8480267	0.314	0.626	0.0007722	2	
KMT2E	-0.8608259	0.31	0.703	1.06E-05	2	Shared with clusters 0 and 2
ZNF395	-0.8849238	0.214	0.61	2.54E-06	2	Shared with clusters 0 and 2
MS4A4A	-0.8918111	0.398	0.659	4.57E-10	2	Shared with clusters 0, 2, 5, and 7
TUBA1C	-0.9105782	0.31	0.653	9.16E-06	2	Shared with clusters 2 and 5
ARRDC3	-0.9153785	0.617	0.752	3.33E-12	2	Shared with clusters 2, 5, and 6
FAM13A	-0.9344519	0.257	0.637	0.00042778	2	Shared with clusters 0, 2, 5, and 7
SLC3A2	-0.9365519	0.309	0.666	0.00035239	2	
SCD	-0.947406	0.189	0.5	0.00375155	2	Shared with clusters 0, 2, 4, and 5
BCAT1	-0.9494964	0.221	0.59	4.22E-08	2	Shared with clusters 2 and 7
MGAT4B	-0.954918	0.13	0.614	1.88E-07	2	Shared with clusters 2, 5, and 7

N4BP2L2	-0.9670917	0.521	0.798	8.97E-06	2	
BACH1	-0.9727001	0.19	0.621	0.00313668	2	Shared with clusters 0 and 2
CHD2	-0.9779004	0.259	0.645	6.31E-08	2	Shared with clusters 2, 5, and 6
ABCA1	-0.9812903	0.31	0.642	7.05E-05	2	Shared with clusters 0, 2, 3, and 8
CYSTM1	-1.007404	0.364	0.65	7.34E-08	2	Shared with clusters 0, 2, 3, 5, 6, and 7
TNFAIP3	-1.0084774	0.17	0.492	0.00694065	2	
CTSC	-1.0166556	0.221	0.59	1.09E-10	2	
WSB1	-1.0280966	0.557	0.784	1.00E-12	2	Shared with clusters 2 and 5
MARCO	-1.0294329	0.075	0.298	1.32E-08	2	Shared with clusters 0,1, 2, and 7
FKBP1A	-1.0545998	0.395	0.689	3.71E-11	2	Shared with clusters 0, 1, 2, 3, 5, and 6
PRRC2C	-1.0831175	0.262	0.72	5.86E-09	2	Shared with clusters 0 and 2
MAP3K2	-1.0874709	0.257	0.673	2.20E-14	2	Shared with clusters 0, 2, and 5
HILPDA	-1.0910209	0.491	0.641	5.78E-05	2	
CCNL1	-1.1144033	0.477	0.736	1.50E-11	2	Shared with clusters 2, 5, and 6
PYCARD	-1.114702	0.331	0.721	2.12E-23	2	Shared with clusters 0 and 2
RGS10	-1.1171743	0.326	0.715	3.69E-06	2	Shared with clusters 0 and 2
YBX3	-1.1471941	0.264	0.709	6.75E-08	2	
MXI1	-1.1479607	0.288	0.717	1.92E-09	2	Shared with clusters 0, 2, 3, and 5
CXCL5	-1.1649585	0.216	0.249	4.87E-68	2	Shared with clusters 0, 2, 3, 5, and 7
ANKRD12	-1.1706749	0.206	0.706	7.39E-05	2	Shared with clusters 2 and 5
MNDA	-1.2199736	0.355	0.664	3.32E-20	2	Shared with clusters 0, 2, 6, and 7
FDX1	-1.2206877	0.489	0.69	1.76E-08	2	Shared with clusters 0, 2, 3, 5, and 6
ZNF292	-1.2298217	0.178	0.676	1.05E-23	2	Shared with clusters 0, 2, 5, and 6
ALCAM	-1.2407155	0.196	0.61	0.00133846	2	Shared with clusters 0, 1, 2, 3, 4 ,5, 6, and 7
H1FX	-1.2461495	0.211	0.567	6.58E-09	2	Shared with clusters 0 and 2
CD48	-1.2492866	0.297	0.725	4.05E-18	2	Shared with clusters 0 and 2
RPN2	-1.2517981	0.153	0.651	6.10E-19	2	Shared with clusters 0, 1, 2, 3, and 5
TREM2	-1.2701388	0.59	0.789	0.00278627	2	Shared with clusters 0, 2, 3, 6, and 7
PHLDA1	-1.2810063	0.53	0.675	1.07E-05	2	Shared with clusters 0, 2, and 7
TNFSF13B	-1.2816037	0.364	0.688	4.43E-11	2	Shared with clusters 0, 2, and 7
IER5L	-1.3019159	0.13	0.667	0.0001173	2	Shared with clusters 2 and 7
BZW1	-1.3151711	0.401	0.751	9.23E-06	2	Shared with clusters 0 and 2
TDRD3	-1.3170991	0.053	0.526	1.35E-05	2	
PDIA6	-1.323671	0.264	0.679	1.63E-20	2	Shared with clusters 1, 2, and 6
TGFBI	-1.3290873	0.228	0.502	7.56E-07	2	Shared with clusters 2, 4, 5, and 7
SCARB2	-1.3474266	0.333	0.748	0.001051	2	Shared with clusters 2, 3, and 7
DMXL2	-1.3694288	0.324	0.705	6.74E-19	2	Shared with clusters 0, 2, 5, and 7
SLC7A7	-1.369857	0.341	0.769	7.60E-05	2	Shared with clusters 0, 2, and 7
CYB5A	-1.3709558	0.496	0.769	4.21E-18	2	Shared with clusters 0, 2, and 5
CDA	-1.3814481	0.364	0.707	8.56E-16	2	Shared with clusters 2 and 7
HLA-DMA	-1.3915839	0.528	0.765	1.26E-25	2	Shared with clusters 0 and 2
C19orf10	-1.391744	0.503	0.803	2.08E-15	2	Shared with clusters 0, 2, and 5
NDUFB5	-1.4076636	0.362	0.841	0.0003936	2	Shared with clusters 2, 5, and 6
LPL	-1.4252784	0.314	0.514	3.50E-16	2	Shared with clusters 2, 4, and 5
PLBD1	-1.4733097	0.201	0.729	6.96E-38	2	Shared with clusters 0, 1, 2, and 6
IER3	-1.4989436	0.168	0.417	1.65E-12	2	Shared with clusters 0 ,2, and 5
DAD1	-1.5131379	0.453	0.843	0.00112304	2	Shared with clusters 1, 2, and 5
FCGR3A	-1.5144464	0.283	0.572	9.09E-15	2	Shared with clusters 0, 2, 5, and 7
DDX17	-1.5382427	0.298	0.792	1.00E-10	2	Shared with clusters 2, 5, and 7
CECR1	-1.5555014	0.3	0.748	0.00015635	2	Shared with clusters 2 and 7
CTSA	-1.5692976	0.544	0.824	6.11E-05	2	Shared with clusters 2 and 7
MTRNR2L8	-1.58859	0.118	0.717	0.00553374	2	
PPT1	-1.5964289	0.429	0.789	1.66E-41	2	Shared with clusters 0, 1, 2, 3, 6, and 7
IFI6	-1.6008989	0.588	0.716	8.81E-19	2	Shared with clusters 2. 3, 5, 6, and 7
C9orf16	-1.6020445	0.556	0.777	1.11E-12	2	Shared with clusters 0, 2, and 6
BCL2A1	-1.6166942	0.415	0.686	0.0035575	2	Shared with clusters 0, 2, 3, 5, 6, and 7
EPB41L3	-1.6172835	0.211	0.64	2.67E-16	2	Shared with clusters 2 and 5
SLC25A5	-1.651759	0.297	0.729	6.67E-20	2	Shared with clusters 0, 2, and 6
PLEKHO1	-1.6775573	0.286	0.751	1.30E-05	2	Shared with clusters 1, 2, 5, 6, and 7
IER2	-1.6855089	0.395	0.627	7.73E-08	2	Shared with clusters 2, 5, and 6
C1QA	-1.6873432	0.141	0.403	3.12E-09	2	Shared with clusters 0, 2, 3, 4, and 7
TMEM91	-1.7010539	0.527	0.845	5.09E-06	2	Shared with clusters 2, 5, and 7
PNISR	-1.7170418	0.611	0.896	2.11E-10	2	Shared with clusters 2 and 5
FAM20C	-1.7199782	0.268	0.728	1.12E-13	2	Shared with clusters 0, 1, 2, 3, 4, 5, 6, and 7

VEGFA	-1.7389479	0.199	0.657	2.32E-18	2	Shared with clusters 0, 2, and 7
SLC11A1	-1.7478234	0.247	0.57	1.15E-30	2	Shared with clusters 0, 2, and 7
CD86	-1.7563381	0.578	0.843	1.89E-12	2	Shared with clusters 2, 3, and 7
IQGAP2	-1.772836	0.208	0.799	2.78E-15	2	Shared with clusters 0, 1, and 2
COX6C	-1.7959594	0.624	0.884	2.68E-14	2	Shared with clusters 0, 1, 2, 5, and 6
TGFB1	-1.8167414	0.307	0.775	0.0008393	2	Shared with clusters 0, 1, 2, 3, 5, and 6
ANXA11	-1.8355715	0.443	0.873	0.00109572	2	Shared with clusters 0, 2, 3, and 5
SCPEP1	-1.8628891	0.278	0.785	4.06E-32	2	Shared with clusters 0, 1, 2, and 7
BNIP3L	-1.9030246	0.54	0.902	1.64E-13	2	Shared with clusters 0 and 2
HEXB	-1.9105787	0.491	0.863	0.00017333	2	Shared with clusters 2 and 7
ITGAM	-1.9646299	0.431	0.86	2.39E-10	2	Shared with clusters 0, 2, and 7
IL4I1	-1.971749	0.283	0.622	1.26E-08	2	Shared with clusters 2, 3, and 7
ZEB2	-1.9886154	0.563	0.873	0.0080235	2	Shared with clusters 0, 2, and 7
RP11-20G13	-2.0488624	0.329	0.495	1.33E-33	2	Shared with clusters 2 and 5
TMEM176B	-2.1025632	0.259	0.654	2.05E-20	2	Shared with clusters 0, 2, and 7
EGR1	-2.1153778	0.278	0.573	4.53E-07	2	Shared with clusters 1, 2, 5, and 6
KIAA0930	-2.1327288	0.37	0.837	2.93E-06	2	Shared with clusters 0, 2, 3, 5, and 7
HEXA	-2.1930326	0.542	0.909	0.00593867	2	Shared with clusters 2 and 7
CALM3	-2.2107583	0.666	0.884	1.65E-09	2	Shared with clusters 0, 2, 3, 6, and 7
ARPC5	-2.2168839	0.645	0.9	0.00061445	2	Shared with clusters 2, 5, and 6
P4HA1	-2.2699238	0.616	0.933	0.00066947	2	Shared with clusters 0 and 2
LGALS2	-2.2791673	0.515	0.521	3.02E-15	2	Shared with clusters 2, 6, and 7
FOSB	-2.2826781	0.516	0.658	0.00122951	2	Shared with clusters 2, 5, and 6
HK2	-2.2998537	0.503	0.817	3.01E-13	2	Shared with clusters 0, 2, 3, and 7
EIF4B	-2.3830491	0.362	0.87	2.83E-05	2	Shared with clusters 2 and 5
CXCL3	-2.3947654	0.492	0.55	0.00894863	2	Shared with clusters 2, 4, and 6
CALM2	-2.4040949	0.751	0.895	9.41E-20	2	Shared with clusters 1, 2, and 6
DBI	-2.4474105	0.576	0.919	1.64E-16	2	Shared with clusters 0, 2, 5, and 6
CTSH	-2.466246	0.576	0.904	5.09E-14	2	Shared with clusters 0, 2, and 7
S100A9	-2.5217266	0.307	0.633	4.80E-17	2	Shared with clusters 0, 1, 2, 3, 5, 6, and 7
A2M	-2.5243442	0.395	0.809	2.14E-31	2	Shared with clusters 0, 2, 3, and 7
GM2A	-2.5790401	0.491	0.84	1.33E-06	2	Shared with clusters 0, 2, 3, 5, 6, and 7
CD74	-2.5807539	0.717	0.919	3.27E-15	2	Shared with clusters 0 and 2
COX5B	-2.6225832	0.887	0.977	6.77E-14	2	Shared with clusters 0, 2, 5, and 6
TMEM59	-2.6278537	0.659	0.94	1.02E-13	2	Shared with clusters 1 and 2
VCAN	-2.6362236	0.214	0.52	8.46E-96	2	Shared with clusters 0, 2, 5, 6, and 7
MT1E	-2.6876692	0.274	0.383	8.84E-12	2	Shared with clusters 0, 1, 2, 3, 4, 6,and 7
LRRFIP1	-2.7101698	0.542	0.928	0.00052532	2	
CFL1	-2.7229236	0.686	0.915	2.38E-05	2	Shared with clusters 0, 1, 2, 3, 5, 6, and 7
LRPAP1	-2.7681438	0.599	0.91	8.47E-11	2	Shared with clusters 2 and 7
CTSL	-2.9114098	0.561	0.731	0.00087335	2	Shared with clusters 0, 1, 2, 3, 5, 6, and 7
BCAP31	-2.9245598	0.664	0.951	1.96E-05	2	Shared with clusters 2, 3, 6, and 7
TXN	-2.9748383	0.696	0.952	0.00081107	2	Shared with clusters 0, 2, 3, 5, and 6
AIF1	-3.0059425	0.53	0.878	0.00333591	2	Shared with clusters 2 and 7
PRDX1	-3.1038164	0.657	0.934	5.32E-09	2	Shared with clusters 0, 1, 2, 3, 5, 6, and 7
CALR	-3.1754871	0.501	0.92	3.83E-12	2	Shared with clusters 2 and 7
MTRNR2L12	-3.1874022	0.218	0.872	1.81E-19	2	Shared with clusters 2, 5, and 6
CAPG	-3.212942	0.532	0.855	2.68E-13	2	Shared with clusters 0, 1, 2, 3, 5, 6, and 7
GUSB	-3.3157666	0.487	0.899	1.27E-46	2	Shared with clusters 0, 1, 2, 3, 5, 6, and 7
TALDO1	-3.4119606	0.597	0.945	1.15E-05	2	Shared with clusters 2, 5, 6, and 7
COTL1	-3.4234891	0.556	0.944	5.35E-13	2	Shared with clusters 0, 2, and 6
LAMP1	-3.5358851	0.696	0.939	6.19E-15	2	Shared with clusters 0, 2, 3, 6, and 7
NDRG1	-3.6341556	0.513	0.901	4.11E-10	2	Shared with clusters 0 and 2
ACP5	-3.6809108	0.796	0.93	2.82E-08	2	Shared with clusters 0, 2, 5, and 7
MFSD12	-3.9891086	0.384	0.755	0.00018243	2	Shared with clusters 0, 1, 2, 3,4, 5, 6, and 7
GSN	-4.0189669	0.467	0.909	2.77E-09	2	Shared with clusters 0, 1, 2, 3, 5, 6, and 7
ATP6AP2	-4.2833104	0.624	0.93	2.51E-23	2	Shared with clusters 0, 1, 2, 3, 5, and 7
ANXA2	-4.3558347	0.722	0.977	1.80E-07	2	Shared with clusters 0, 1, 2, 3, 5, 6, and 7
TIMP2	-4.3602221	0.41	0.97	4.90E-29	2	Shared with clusters 0, 2, 5, and 7
SLC2A3	-4.3796463	0.492	0.855	1.83E-07	2	
SOD2	-4.5260275	0.768	0.94	1.41E-05	2	
APOC1	-4.6478797	0.967	0.963	3.59E-66	2	Shared with clusters 0, 1, 2, 3, 4, 5, 6, and 7
NCF2	-4.6812917	0.585	0.98	9.00E-06	2	Shared with clusters 2 and 7
H3F3B	-4.8066094	0.955	0.994	0.00011616	2	Shared with clusters 2, 5, and 6

CD9	-5.0880922	0.78	0.965	0.00325383	2	Shared with clusters 1, 2, 5, and 7
CD81	-5.0902668	0.262	0.958	1.37E-61	2	Shared with clusters 0, 1, 2, 5, and 6
CYP1B1	-5.1563368	0.503	0.881	1.70E-19	2	Shared with clusters 0, 1, 2, 3, 5, and 7
CYP27A1	-5.1638578	0.69	0.97	8.47E-13	2	Shared with clusters 2, 5, and 6
FABP4	-5.257828	0.499	0.368	1.16E-08	2	Shared with clusters 0, 1, 2, 3, 4, 5, 6, and 7
BTG1	-5.9091836	0.976	0.987	4.85E-24	2	Shared with clusters 0, 2, 6, and 7
ARPC1B	-6.0842468	0.561	0.945	2.86E-48	2	Shared with clusters 0, 1, 2, 3, 5, 6, and 7
CTSZ	-6.1619232	0.597	0.971	1.41E-12	2	Shared with clusters 0, 1, 2, 3, 6, and 7
TXNIP	-6.4070217	0.741	0.975	4.30E-07	2	Shared with clusters 0, 2, 3, and 5
RNASET2	-6.8141544	0.945	0.995	0.00010135	2	Shared with clusters 0 and 2
EREG	-6.8764449	0.492	0.656	9.60E-07	2	Shared with clusters 1, 2, 5, 6, and 7
FBP1	-6.9555288	0.578	0.976	2.68E-36	2	Shared with clusters 0, 1, 2, 3, 5, 6, and 7
GRN	-7.0395066	0.868	0.989	0.00229883	2	Shared with clusters 2 and 7
MMP7	-7.7723538	0.226	0.264	1.72E-22	2	Shared with clusters 2, 5, 6, and 7
RPS27	-7.7846283	0.997	1	0.00144207	2	Shared with clusters 0, 2, and 7
RPL21	-8.2715721	0.979	1	3.17E-05	2	Shared with clusters 0, 2, and 5
ZFP36L1	-8.3923791	0.882	0.969	4.89E-29	2	Shared with clusters 0, 2, 5, 6, and 7
RPL34	-8.5714512	1	1	0.00301626	2	Shared with clusters 0 and 2
S100A4	-9.0573728	0.852	0.958	7.21E-23	2	Shared with clusters 0, 2, and 6
TIMP1	-9.1206306	0.851	0.96	5.81E-07	2	Shared with clusters 2, 3, 5, 6, and 7
MT-ND5	-9.3631083	0.69	0.966	0.00051223	2	
OAZ1	-9.5245034	0.978	1	5.40E-21	2	
HCST	-10.214565	0.943	0.989	6.40E-12	2	Shared with clusters 2, 6, and 7
FABP5	-10.685599	0.887	0.879	1.26E-15	2	Shared with clusters 0, 1, 2, 3, 4, 5, 6, and 7
APOE	-10.688432	0.954	0.958	3.20E-75	2	Shared with clusters 0, 2, 3, 5, 6, and 7
CD52	-11.642421	0.988	0.999	2.66E-08	2	Shared with clusters 2, 6, and 7
LIPA	-12.149588	0.889	0.961	2.12E-99	2	Shared with clusters 0, 1, 2, 3, 4, 5, 6, and 7
SERF2	-13.361952	1	1	5.95E-15	2	Shared with clusters 1, 2, and 6
RPL41	-15.119763	1	1	1.83E-07	2	Shared with clusters 0, 2, and 7
MT2A	-15.804991	0.64	0.715	2.00E-06	2	Shared with clusters 0, 2, 1, 3, 4, 5, 6, and 7
PLIN2	-16.164705	0.804	0.994	1.36E-08	2	Shared with clusters 0, 2, and 7
CHI3L1	-17.693782	0.491	0.751	2.08E-104	2	Shared with clusters 0, 1, 2, 3, 4, 5, 6, and 7
RPS23	-17.781775	0.969	1	0.00027199	2	Shared with clusters 0, 2, and 6
CD63	-19.174405	0.986	0.999	4.90E-26	2	Shared with clusters 0, 1, 2, 3, 4, 5, 6, and 7
S100A6	-19.730004	0.997	1	9.53E-08	2	Shared with clusters 1, 2, 5, and 6
TMSB10	-20.111862	1	1	9.84E-14	2	Shared with clusters 2 and 6
RPS24	-20.824575	0.978	1	8.79E-20	2	Shared with clusters 0, 1, 2, and 6
MT-ND1	-23.446296	0.779	0.994	4.21E-08	2	Shared with clusters 0, 2, and 5
HLA-B	-23.550777	0.991	1	0.00087378	2	Shared with clusters 1 and 2
LGALS3	-23.993837	0.937	0.998	2.31E-25	2	Shared with clusters 0, 1, 2, 3, 4, 5, 6, and 7
RPLP1	-26.411736	0.997	1	1.06E-05	2	Shared with clusters 0, 2, and 6
MMP9	-26.653584	0.83	0.971	5.12E-29	2	Shared with clusters 0, 1, 2, 3, 4, 5, 6, and 7
NEAT1	-38.934862	1	0.998	9.06E-24	2	Shared with clusters 2 and 6
TMSB4X	-45.56403	1	1	8.05E-10	2	Shared with clusters 0 and 2
EEF1A1	-53.157311	0.998	1	1.59E-06	2	Shared with clusters 2 and 5
B2M	-53.420655	1	1	0.00012928	2	Shared with clusters 1 and 2
MT-CO2	-60.741053	0.916	0.998	4.14E-09	2	Shared with clusters 0, 2, and 6
MT-ND4	-61.920772	0.952	1	0.00025785	2	Shared with clusters 0, 2, 3, 5, and 7
MT-CO1	-72.206462	0.985	1	0.00025102	2	Shared with clusters 0, 1, 2, 3, and 5
SPP1	-178.71498	0.991	1	2.24E-78	2	Shared with clusters 0, 1, 2, 3, 4, 5, 6, and 7
MALAT1	-245.56224	1	1	7.23E-21	2	Shared with clusters 0 and 2
APOC1	1.46003904	0.723	0.684	1.27E-16	3	Shared with clusters 0, 1, 2, 3, 4, 5, 6, and 7
APOE	0.8828714	0.737	0.747	1.50E-25	3	Shared with clusters 0, 2, 3, 5, 6, and 7
NCS1	-0.0289014	0.005	0.031	4.33E-06	3	Shared with clusters 0, 1, 3, 4, 5, and 6
TGM2	-0.0299249	0.007	0.033	9.96E-09	3	Shared with clusters 1, 3, 4, 5, and 6
SPARC	-0.0334868	0.012	0.037	5.58E-14	3	Shared with clusters 0, 2, and 3
IL7R	-0.0339934	0.002	0.028	8.36E-07	3	Shared with clusters 0, 2, 3, 6, and 7
ITGB5	-0.0351624	0.012	0.041	3.75E-07	3	Shared with clusters 0, 3, and 5, and 6
SLAMF7	-0.0358947	0	0.033	2.54E-12	3	Shared with clusters 3 and 6
CD276	-0.0402945	0.005	0.045	2.05E-05	3	Shared with clusters 0, 3, 5, and 6
GCLC	-0.0423666	0.024	0.06	4.96E-19	3	Shared with clusters 1, 2, 3, 4, 5, 6, and 7
CCND2	-0.047925	0.012	0.053	5.32E-05	3	Shared with clusters 0, 3, and 6
STIP1	-0.0484567	0.012	0.053	0.00116428	3	Shared with clusters 0, 3, 5, and 6
HK3	-0.0508497	0.01	0.057	1.34E-14	3	Shared with clusters 0, 1, 3, 5, and 6

MARCH2	-0.0561674	0.012	0.061	3.28E-09	3	Shared with clusters 0, 2, 3, 5, and 7
UCP2	-0.0564243	0.09	0.137	0.00013592	3	Shared with clusters 0 and 3
IFI6	-0.0585915	0.083	0.109	2.81E-16	3	Shared with clusters 2. 3, 5, 6, and 7
PHLDA3	-0.0603013	0.017	0.069	0.00030224	3	Shared with clusters 0, 3, 5, 6, and 7
G6PD	-0.0658403	0.063	0.114	2.09E-27	3	Shared with clusters 0, 1, 2, 3, 4, 5, 6, and 7
OAS1	-0.0699386	0.032	0.085	0.0008196	3	Shared with clusters 0, 2, and 3
FLT1	-0.0746741	0.002	0.065	0.00167625	3	Shared with clusters 1, 3, 4, 5, and 6
MRAS	-0.0796962	0.034	0.099	0.00081099	3	Shared with clusters 0, 3, and 5
CD300LB	-0.0875524	0.034	0.102	0.00230138	3	Shared with clusters 0,1, 2, 3, and 5
CD59	-0.0954443	0.085	0.158	8.15E-07	3	Shared with clusters 0, 1, 2, 3, 5, 6, and 7
ITGB7	-0.0955989	0.007	0.085	7.16E-15	3	Shared with clusters 0, 1, 2, and 3
DLG1	-0.096251	0	0.083	1.56E-05	3	
EMP1	-0.1000058	0.17	0.23	0.00058929	3	Shared with clusters 0, 1, 3, 5, and 6
FDX1	-0.1113231	0.156	0.225	8.62E-13	3	Shared with clusters 0, 2, 3, 5, and 6
TREM2	-0.1121253	0.097	0.174	1.15E-08	3	Shared with clusters 0, 2, 3, 6, and 7
CHI3L1	-0.1146638	0.109	0.176	5.55E-220	3	Shared with clusters 0, 1, 2, 3, 4, 5, 6, and 7
C1QC	-0.1231961	0.022	0.101	3.68E-16	3	Shared with clusters 0, 3, and 5
FKBP1A	-0.124292	0.175	0.257	4.07E-09	3	Shared with clusters 0, 1, 2, 3, 5, and 6
FABP4	-0.1379771	0.163	0.137	5.25E-44	3	Shared with clusters 0, 1, 2, 3, 4, 5, 6, and 7
ITGB1BP1	-0.1473724	0.085	0.2	4.08E-06	3	Shared with clusters 0, 3, 5, and 6
PTGR1	-0.1583734	0.022	0.154	5.20E-06	3	Shared with clusters 0, 1, 3, 5, and 6
HAVCR2	-0.1619561	0.083	0.206	0.00143654	3	Shared with clusters 3 and 7
NCEH1	-0.1665861	0.051	0.172	3.87E-06	3	Shared with clusters 3, 5, and 7
SIRPB1	-0.1698466	0.019	0.153	0.00310641	3	
MATK	-0.1966417	0.078	0.22	3.64E-11	3	Shared with clusters 0, 3, 4, 5, 6, and 7
PPT1	-0.2042023	0.112	0.231	0.00122513	3	Shared with clusters 0, 1, 2, 3, 6, and 7
ATP1B3	-0.206114	0.134	0.267	0.00103415	3	Shared with clusters 3, 5, and 7
ATP6V1D	-0.2068761	0.097	0.25	0.00394864	3	Shared with clusters 3, 5, 6, and 7
MGLL	-0.2169797	0.095	0.218	1.40E-07	3	Shared with clusters 2, 3, 4, 5, 6, and 7
CD84	-0.2208924	0.054	0.192	1.27E-20	3	Shared with clusters 0, 1, 3, 5, 6, and 7
CDC42EP3	-0.2296964	0.034	0.199	7.13E-05	3	
CYSTM1	-0.2336413	0.17	0.327	0.00162794	3	Shared with clusters 0, 2, 3, 5, 6, and 7
ALCAM	-0.2342165	0.029	0.194	4.45E-43	3	Shared with clusters 0, 1, 2, 3, 4 ,5, 6, and 7
RPN2	-0.2388821	0.039	0.207	8.07E-05	3	Shared with clusters 0, 1, 2, 3, and 5
TXNRD1	-0.2453688	0.046	0.182	9.67E-35	3	Shared with clusters 0, 1, 3, 4, 5, 6, and 7
C3	-0.2566771	0.073	0.254	1.45E-05	3	Shared with clusters 0, 3, 4, 5, 6, and 7
C1QA	-0.2635041	0.027	0.129	6.63E-33	3	Shared with clusters 0, 2, 3, 4, and 7
HSPA8	-0.2656784	0.173	0.32	4.77E-05	3	Shared with clusters 0, 3, and 5
ABHD2	-0.2674924	0.032	0.228	1.84E-06	3	Shared with clusters 0, 3, 5, and 7
MYOF	-0.2762117	0.046	0.245	1.21E-05	3	Shared with clusters 0, 3, 5, and 7
CD86	-0.3091752	0.29	0.422	1.06E-06	3	Shared with clusters 2, 3, and 7
HIST1H1D	-0.3191695	0.005	0.187	2.99E-06	3	Shared with clusters 2 and 3
FAM20C	-0.3225115	0.078	0.27	1.64E-30	3	Shared with clusters 0, 1, 2, 3, 4, 5, 6, and 7
FABP5	-0.3898676	0.474	0.535	9.51E-133	3	Shared with clusters 0, 1, 2, 3, 4, 5, 6, and 7
A2M	-0.3923492	0.066	0.248	5.33E-14	3	Shared with clusters 0, 2, 3, and 7
HMG20B	-0.4094429	0.2	0.412	6.41E-19	3	Shared with clusters 0, 1, 3, 4, 5, and 7
STAB1	-0.4131181	0.032	0.215	0.00054414	3	Shared with clusters 3 and 7
BCL2A1	-0.422342	0.217	0.392	2.37E-07	3	Shared with clusters 0, 2, 3, 5, 6, and 7
LIPA	-0.4447146	0.409	0.495	1.99E-117	3	Shared with clusters 0, 1, 2, 3, 4, 5, 6, and 7
PTPN6	-0.4734149	0.092	0.384	1.53E-06	3	Shared with clusters 0, 3, 5, 6, and 7
CD36	-0.5061571	0.268	0.438	0.00011767	3	Shared with clusters 3 and 7
CALM3	-0.5205862	0.326	0.56	8.58E-07	3	Shared with clusters 0, 2, 3, 6, and 7
SCARB2	-0.5210293	0.141	0.439	0.00015203	3	Shared with clusters 2, 3, and 7
TPM4	-0.5219324	0.148	0.419	0.00391581	3	Shared with clusters 3, 5, and 7
PSMA7	-0.5494501	0.431	0.626	0.00158457	3	Shared with clusters 0, 3, 5, and 6
MFSD12	-0.55581	0.112	0.426	2.16E-94	3	Shared with clusters 0, 1, 2, 3,4, 5, 6, and 7
GUSB	-0.5643585	0.119	0.428	4.91E-15	3	Shared with clusters 0, 1, 2, 3, 5, 6, and 7
S100A9	-0.5663504	0.127	0.338	7.22E-06	3	Shared with clusters 0, 1, 2, 3, 5, 6, and 7
FERMT3	-0.6114308	0.139	0.479	1.49E-05	3	Shared with clusters 0, 1, 3, 5, 6, and 7
NFIL3	-0.6353593	0.036	0.442	0.00142658	3	Shared with clusters 3 and 5
CTSL	-0.6652227	0.268	0.475	2.62E-14	3	Shared with clusters 0, 1, 2, 3, 5, 6, and 7
IL4I1	-0.7162969	0.2	0.396	2.37E-05	3	Shared with clusters 2, 3, and 7
ANXA11	-0.7165673	0.195	0.555	0.00019367	3	Shared with clusters 0, 2, 3, and 5
CFL1	-0.730026	0.406	0.672	4.89E-12	3	Shared with clusters 0, 1, 2, 3, 5, 6, and 7

LAMP1	-0.7414908	0.221	0.529	1.89E-14	3	Shared with clusters 0, 2, 3, 6, and 7
GM2A	-0.7544022	0.112	0.486	4.88E-13	3	Shared with clusters 0, 2, 3, 5, 6, and 7
ASAP1	-0.7776546	0.105	0.495	0.00232855	3	Shared with clusters 3 and 5
KIAA0930	-0.8134795	0.119	0.5	3.14E-07	3	Shared with clusters 0, 2, 3, 5, and 7
STAT3	-0.8335413	0.131	0.559	0.0014371	3	
TGFB1	-0.8349316	0.161	0.576	0.0049544	3	Shared with clusters 0, 1, 2, 3, 5, and 6
CAPG	-0.8421027	0.294	0.594	6.87E-13	3	Shared with clusters 0, 1, 2, 3, 5, 6, and 7
FOXN3	-0.8546208	0.056	0.528	4.30E-05	3	Shared with clusters 3 and 5
RYBP	-0.8793185	0.08	0.532	1.21E-06	3	Shared with clusters 0, 2, 3, and 5
IL1RN	-0.8884977	0.304	0.577	2.30E-22	3	Shared with clusters 0, 1, 3, 4, 5 6, and 7
CCDC88A	-0.9801057	0.17	0.591	0.00294324	3	Shared with clusters 3, 5, and 7
BCAP31	-1.0383627	0.345	0.709	0.00181258	3	Shared with clusters 2, 3, 6, and 7
ARPC1B	-1.0481501	0.307	0.635	5.12E-39	3	Shared with clusters 0, 1, 2, 3, 5, 6, and 7
ICAM3	-1.0872775	0.114	0.503	6.65E-05	3	Shared with clusters 3 and 5
TYMP	-1.1413793	0.438	0.708	0.00981173	3	Shared with clusters 3, 5, and 7
PRDX1	-1.1507106	0.462	0.747	2.23E-05	3	Shared with clusters 0, 1, 2, 3, 5, 6, and 7
FUCA1	-1.2468095	0.122	0.48	0.00075218	3	Shared with clusters 3, 4, 5, and 7
FN1	-1.3020635	0.068	0.178	7.07E-09	3	Shared with clusters 1, 3, and 7
ATP6AP2	-1.3386245	0.355	0.745	0.00081653	3	Shared with clusters 0, 1, 2, 3, 5, and 7
MDM2	-1.3494683	0.219	0.64	0.00160275	3	Shared with clusters 2, 3, and 5
GSN	-1.4072149	0.141	0.687	3.39E-20	3	Shared with clusters 0, 1, 2, 3, 5, 6, and 7
TXN	-1.4691503	0.586	0.85	0.00541935	3	Shared with clusters 0, 2, 3, 5, and 6
TUBA1B	-1.5358724	0.35	0.794	6.99E-06	3	Shared with clusters 0, 1, 3, 5, 6, and 7
ANXA2	-1.5833083	0.491	0.864	4.97E-09	3	Shared with clusters 0, 1, 2, 3, 5, 6, and 7
CYP1B1	-1.6811035	0.141	0.57	3.30E-05	3	Shared with clusters 0, 1, 2, 3, 5, and 7
FLNA	-1.7523831	0.277	0.766	2.69E-09	3	Shared with clusters 1, 3, 5, and 6
FBP1	-1.8128449	0.258	0.777	4.93E-17	3	Shared with clusters 0, 1, 2, 3, 5, 6, and 7
GSTO1	-2.1351099	0.689	0.958	0.00654865	3	Shared with clusters 0, 3, 5, 6, and 7
CTSZ	-2.196564	0.246	0.812	0.0069576	3	Shared with clusters 0, 1, 2, 3, 6, and 7
MXI1	-2.6738068	0.331	0.906	3.25E-05	3	Shared with clusters 0, 2, 3, and 5
CXCR4	-2.8658016	0.691	0.935	3.19E-05	3	Shared with clusters 0, 2, 3, and 5
MT1E	-2.9050008	0.513	0.659	1.46E-29	3	Shared with clusters 0, 1, 2, 3, 4, 6,and 7
CD63	-4.3867339	0.942	0.996	8.62E-13	3	Shared with clusters 0, 1, 2, 3, 4, 5, 6, and 7
HK2	-4.6196455	0.479	0.96	0.00348779	3	Shared with clusters 0, 2, 3, and 7
ACTB	-4.9224381	0.854	0.986	1.33E-06	3	Shared with clusters 0, 1, 3, 5, 6, and 7
CXCL5	-5.2222367	0.255	0.593	0.00318856	3	Shared with clusters 0, 2, 3, 5, and 7
MMP9	-5.8652187	0.572	0.93	6.08E-28	3	Shared with clusters 0, 1, 2, 3, 4, 5, 6, and 7
LGALS3	-6.1006941	0.878	0.99	4.86E-11	3	Shared with clusters 0, 1, 2, 3, 4, 5, 6, and 7
PSAP	-7.0042548	0.861	0.993	0.00032482	3	Shared with clusters 3 and 7
NPC2	-7.302363	0.993	0.999	1.45E-05	3	Shared with clusters 3 and 5
TKT	-7.7912498	0.659	0.997	0.00651692	3	Shared with clusters 3 and 5
MT-ND2	-11.759042	0.628	0.987	7.19E-05	3	
MT1G	-12.120172	0.523	0.765	9.24E-105	3	Shared with clusters 0, 1, 3, 4, 5, 6,and 7
TXNIP	-12.600763	0.847	0.998	0.00047178	3	Shared with clusters 0, 2, 3, and 5
MT1H	-14.002093	0.214	0.414	2.62E-68	3	Shared with clusters 0, 1, 3, 4, 5, and 7
TIMP1	-14.422883	0.764	0.991	3.38E-08	3	Shared with clusters 2, 3, 5, 6, and 7
SPP1	-18.034837	0.973	0.998	1.99E-115	3	Shared with clusters 0, 1, 2, 3, 4, 5, 6, and 7
MT1X	-43.341144	0.993	0.999	0	3	Shared with clusters 0, 1, 3, 4, 5, 6,and 7
MT-ND4	-43.521681	0.876	0.998	4.28E-09	3	Shared with clusters 0, 2, 3, 5, and 7
MT2A	-43.53026	0.998	1	0	3	Shared with clusters 0, 2, 1, 3, 4, 5, 6, and 7
MT-CO1	-44.472576	0.954	1	1.06E-06	3	Shared with clusters 0, 1, 2, 3, and 5
LYZ	-78.411162	0.988	1	2.87E-07	3	Shared with clusters 3 and 7
HLA-DRB5	0.95124617	0.426	0.054	0.00091577	4	
APOC1	0.70692666	0.722	0.707	0.00193737	4	Shared with clusters 0, 1, 2, 3, 4, 5, 6, and 7
CYP27B1	-0.0280221	0.005	0.028	1.28E-06	4	Shared with clusters 4 and 5
TM4SF19.1	-0.0282489	0.019	0.045	3.02E-23	4	Shared with clusters 2, 4, and 5
SLC28A3	-0.0295666	0	0.027	3.84E-12	4	Shared with clusters 4 and 5
PAQR7	-0.0303767	0	0.03	1.30E-05	4	
KBTBD6	-0.0336168	0	0.033	4.73E-06	4	
LPL	-0.0345025	0.005	0.035	1.24E-98	4	Shared with clusters 2, 4, and 5
LINC00900	-0.0380721	0	0.037	0.00072838	4	
ADAL	-0.0506278	0	0.045	0.00053718	4	
NCS1	-0.0567787	0.005	0.058	0.00035011	4	Shared with clusters 0, 1, 3, 4, 5, and 6
IQSEC2	-0.0587282	0	0.057	1.05E-06	4	

PSME3	-0.0587282	0	0.054	0.00150181	4	
FLT1	-0.0611584	0	0.055	1.48E-44	4	Shared with clusters 1, 3, 4, 5, and 6
WDR3	-0.0631835	0	0.062	6.89E-05	4	
FSTL3	-0.0676387	0	0.064	1.54E-05	4	
TGM2	-0.0692588	0	0.065	2.46E-30	4	Shared with clusters 1, 3, 4, 5, and 6
GCLC	-0.0781635	0.062	0.132	0.00533504	4	Shared with clusters 1, 2, 3, 4, 5, 6, and 7
EMILIN1	-0.0878898	0	0.079	1.16E-55	4	Shared with clusters 0, 1, 4, 5, and 6
RTN4RL2	-0.0919401	0	0.085	1.08E-07	4	Shared with clusters 4 and 5
RBM19	-0.1032807	0	0.098	5.47E-05	4	
PAFAH2	-0.1069259	0	0.096	1.53E-05	4	
CHI3L1	-0.1161057	0.096	0.152	6.74E-110	4	Shared with clusters 0, 1, 2, 3, 4, 5, 6, and 7
GBP1	-0.117127	0.005	0.109	0.00335713	4	
SZT2	-0.1206966	0	0.109	1.16E-06	4	
SDCCAG3	-0.1211017	0	0.115	0.00011742	4	
PLEKHH3	-0.1251519	0	0.113	0.00485016	4	
GDF15	-0.1405427	0	0.075	0.00014102	4	
TNKS	-0.1969164	0.005	0.182	0.00049219	4	
G6PD	-0.2124797	0.067	0.225	2.24E-05	4	Shared with clusters 0, 1, 2, 3, 4, 5, 6, and 7
ADAMTSL4	-0.2181287	0.014	0.202	0.00647638	4	
C3	-0.2427808	0.057	0.239	3.36E-09	4	Shared with clusters 0, 3, 4, 5, 6, and 7
MRC1L1	-0.2528851	0.01	0.21	2.68E-06	4	Shared with clusters 0, 4, and 7
MATK	-0.2680375	0.12	0.321	0.0001035	4	Shared with clusters 0, 3, 4, 5, 6, and 7
MGLL	-0.2769752	0.11	0.262	7.38E-07	4	Shared with clusters 2, 3, 4, 5, 6, and 7
MT1E	-0.2806959	0.124	0.15	4.17E-40	4	Shared with clusters 0, 1, 2, 3, 4, 6,and 7
SCD	-0.2999645	0.062	0.276	0.00440384	4	Shared with clusters 0, 2, 4, and 5
FAM102B	-0.3122722	0	0.254	1.54E-32	4	
ALCAM	-0.3298335	0.081	0.299	3.96E-14	4	Shared with clusters 0, 1, 2, 3, 4 ,5, 6, and 7
FAM111A	-0.3463696	0.005	0.28	2.67E-06	4	
FABP4	-0.3606307	0.148	0.132	2.17E-26	4	Shared with clusters 0, 1, 2, 3, 4, 5, 6, and 7
MT1G	-0.3822054	0.11	0.177	1.19E-67	4	Shared with clusters 0, 1, 3, 4, 5, 6,and 7
TXNRD1	-0.4461931	0.148	0.37	5.55E-07	4	Shared with clusters 0, 1, 3, 4, 5, 6, and 7
MT1H	-0.5097932	0.024	0.144	2.45E-31	4	Shared with clusters 0, 1, 3, 4, 5, and 7
HMG20B	-0.5301994	0.254	0.527	0.00031949	4	Shared with clusters 0, 1, 3, 4, 5, and 7
FABP5	-0.5762653	0.483	0.565	1.10E-32	4	Shared with clusters 0, 1, 2, 3, 4, 5, 6, and 7
FAM20C	-0.5888423	0.11	0.4	0.00018386	4	Shared with clusters 0, 1, 2, 3, 4, 5, 6, and 7
CARD16	-0.749111	0.679	0.756	0.00796788	4	Shared with clusters 4 and 7
ZCCHC2	-0.8116743	0.191	0.583	0.00616908	4	Shared with clusters 4 and 7
VSIG4	-0.8246467	0.053	0.417	0.00129904	4	
IL1RN	-0.932402	0.306	0.57	4.03E-09	4	Shared with clusters 0, 1, 3, 4, 5 6, and 7
MFSD12	-0.9837119	0.124	0.567	6.07E-30	4	Shared with clusters 0, 1, 2, 3,4, 5, 6, and 7
SAMSN1	-1.0868782	0.263	0.678	0.00011654	4	
RHOB	-1.4791181	0.22	0.619	9.83E-06	4	Shared with clusters 4 and 5
LIPA	-1.571405	0.512	0.672	8.92E-13	4	Shared with clusters 0, 1, 2, 3, 4, 5, 6, and 7
ZNF331	-1.7604904	0.167	0.676	8.64E-07	4	Shared with clusters 2, 4, and 5
CPVL	-1.9687145	0.249	0.796	0.00964116	4	Shared with clusters 4 and 5
C1QA	-1.9843165	0.124	0.478	5.57E-09	4	Shared with clusters 0, 2, 3, 4, and 7
DUSP2	-2.1031625	0.359	0.693	0.00201232	4	Shared with clusters 4, 5, and 6
ADM	-2.1747022	0.608	0.792	0.00061201	4	Shared with clusters 2 and 4
VMO1	-2.2178962	0.397	0.583	1.52E-17	4	Shared with clusters 2, 4, and 7
MT1X	-2.6933671	0.335	0.379	1.11E-61	4	Shared with clusters 0, 1, 3, 4, 5, 6,and 7
METRNL	-2.7332298	0.124	0.855	1.04E-06	4	
MT2A	-2.8338285	0.684	0.587	3.02E-48	4	Shared with clusters 0, 2, 1, 3, 4, 5, 6, and 7
HLA-DPA1	-2.8345939	0.617	0.836	1.34E-07	4	Shared with clusters 0 and 4
FUCA1	-3.4567721	0.187	0.763	1.28E-08	4	Shared with clusters 3, 4, 5, and 7
FCN1	-4.3128497	0.593	0.91	0.00492028	4	Shared with clusters 0, 4, and 7
HLA-DRB1	-4.4830152	0.775	0.872	7.20E-14	4	Shared with clusters 0, 1, and 4
CD63	-4.5323155	0.923	0.996	0.00457808	4	Shared with clusters 0, 1, 2, 3, 4, 5, 6, and 7
TGFBI	-4.7692613	0.541	0.91	2.69E-05	4	Shared with clusters 2, 4, 5, and 7
LGALS3	-6.2886181	0.766	0.985	0.00272821	4	Shared with clusters 0, 1, 2, 3, 4, 5, 6, and 7
HLA-DRA	-6.4182194	0.895	0.951	9.17E-06	4	Shared with clusters 0 and 4
IL8	-6.8507096	1	0.984	0.00460152	4	Shared with clusters 0, 2, 4, 5, and 6
MMP9	-7.2944744	0.512	0.868	0.00923179	4	Shared with clusters 0, 1, 2, 3, 4, 5, 6, and 7
CXCL3	-7.7826251	0.794	0.888	0.00027893	4	Shared with clusters 2, 4, and 6
UBC	-12.033789	0.914	1	0.00027699	4	

RNASE1	-13.19619	0.344	0.78	0.00010274	4	Shared with clusters 0, 4, and 7
RPL3	-19.910895	0.976	1	0.00018199	4	Shared with clusters 0, 1, and 4
RPL32	-20.645478	1	1	5.18E-05	4	Shared with clusters 0, 1, and 4
SPP1	-25.78748	0.88	0.989	3.68E-18	4	Shared with clusters 0, 1, 2, 3, 4, 5, 6, and 7
RPS2	-27.00935	1	1	1.67E-05	4	Shared with clusters 0 and 4
RPL7A	-27.19482	0.981	1	8.64E-05	4	Shared with clusters 0, 1, 4, 5, 6, and 7
RPS12	-27.439965	1	1	0.00615106	4	Shared with clusters 0, 4, 5, and 6
RPS18	-31.486104	1	1	2.39E-06	4	Shared with clusters 0, 1, 4, and 6
RPL13	-34.706206	1	1	0.00271678	4	Shared with clusters 0, 1, 4, 5, and 6
RPL10	-42.869943	1	1	0.00739197	4	Shared with clusters 0, 4, and 7
VIM	-117.30433	0.995	1	0.0004504	4	Shared with clusters 4 and 7
IFI30	1.09482582	0.603	0.261	4.36E-10	5	Shared with clusters 5 and 6
RGS1	0.2935161	0.603	0.591	2.99E-08	5	Shared with clusters 0 and 5
RP11-1114A	-0.0273166	0.005	0.029	7.49E-07	5	Shared with clusters 5 and 6
SIRPB2	-0.0276074	0	0.027	1.89E-32	5	
DKFZP667F	-0.0284879	0.014	0.042	0.00025306	5	
CD93	-0.0309697	0.009	0.036	3.29E-19	5	Shared with clusters 0, 2, 5, and 6
CLEC10A	-0.0318461	0.009	0.032	7.57E-07	5	
HSPA6	-0.0322885	0.023	0.053	0.00026023	5	Shared with clusters 5 and 7
FPR1	-0.0324318	0.014	0.038	3.13E-18	5	Shared with clusters 5 and 6
TMEM71	-0.0324359	0.028	0.057	1.44E-08	5	
RP5-894A10	-0.0334516	0.005	0.037	6.91E-09	5	
BCL2L11	-0.0337464	0.014	0.043	3.75E-07	5	
ARHGAP6	-0.0347703	0.019	0.05	1.35E-05	5	
TRAF3IP3	-0.0357941	0.023	0.056	0.00152131	5	
DGKE	-0.0360849	0.019	0.053	2.44E-08	5	Shared with clusters 2 and 5
ADAMTS10	-0.0360849	0.019	0.052	1.98E-05	5	
FAM26F	-0.0391524	0.014	0.054	1.24E-07	5	
HBEGF	-0.0391565	0.033	0.06	1.46E-05	5	Shared with clusters 2 and 5
ERO1LB	-0.0400329	0.033	0.069	0.00880777	5	
RUNX2	-0.0401804	0.037	0.071	0.00140112	5	
NFXL1	-0.0413435	0.019	0.055	6.09E-14	5	Shared with clusters 0, 2, 5, 6, and 7
THBD	-0.0420725	0.014	0.043	4.47E-30	5	Shared with clusters 0, 2, and 5
HERC5	-0.0423632	0.009	0.045	0.00016437	5	
XYLT1	-0.0428014	0.009	0.05	1.70E-07	5	
RP11-640M	-0.0441243	0.037	0.078	3.73E-05	5	
CSF2RB	-0.044415	0.033	0.074	0.00063044	5	
RP11-796E2	-0.0461802	0.07	0.101	0.00108609	5	
SIPA1L1	-0.047331	0.014	0.059	3.89E-05	5	
DSC2	-0.0474784	0.019	0.063	0.00047241	5	
PDGFC	-0.0474825	0.033	0.076	0.00589635	5	
SEMA4D	-0.0511316	0.023	0.069	2.47E-07	5	Shared with clusters 2 and 5
CASP1	-0.0512954	0.084	0.113	1.56E-07	5	
OLFML2B	-0.0518647	0.028	0.072	2.86E-07	5	
RASGRP4	-0.0525895	0.014	0.064	0.00631498	5	
MPEG1	-0.0531793	0.033	0.078	1.82E-05	5	
REV3L	-0.0552352	0.07	0.107	6.63E-15	5	Shared with clusters 0, 2, and 5
ULK1	-0.0562468	0.033	0.086	3.61E-05	5	Shared with clusters 2 and 5
VNN 1.00	-0.0565294	0	0.041	1.47E-46	5	Shared with clusters 0, 2, and 5
CBX7	-0.0565376	0.023	0.081	1.69E-11	5	Shared with clusters 5 and 6
EPB41L4A-A	-0.056558	0.098	0.125	4.88E-09	5	
SP4	-0.0574058	0	0.056	0.00402829	5	
FAM193B	-0.0581471	0.037	0.093	8.07E-05	5	Shared with clusters 2 and 5
GPR155	-0.0590194	0.023	0.077	1.39E-07	5	
CD101	-0.0595968	0	0.056	1.54E-101	5	Shared with clusters 2 and 5
RP11-67L2.2	-0.0595968	0	0.058	8.67E-06	5	
ZBTB20	-0.0606289	0.033	0.088	0.00192179	5	Shared with clusters 5, 6, and 7
RNF144B	-0.0616609	0.065	0.11	0.0006813	5	
ASGR1	-0.0634056	0.037	0.094	8.83E-06	5	
TANC2	-0.063553	0.037	0.099	0.00010434	5	Shared with clusters 2 and 5
RCOR3	-0.0653223	0.093	0.155	0.00117254	5	
PSIP1	-0.0657441	0.042	0.09	1.61E-13	5	
SYTL3	-0.066469	0.023	0.077	2.81E-09	5	
HIST1H2AC	-0.0666205	0.037	0.083	5.31E-14	5	Shared with clusters 2 and 5

C9orf72	-0.0676567	0.089	0.132	7.63E-06	5	Shared with clusters 2 and 5
MIR142	-0.068271	0.168	0.207	0.00735303	5	
RP11-932O9	-0.0693849	0.005	0.073	8.30E-08	5	
ST8SIA4	-0.0708552	0.037	0.098	1.75E-27	5	Shared with clusters 2 and 5
ACVR2B	-0.0720183	0.019	0.087	2.53E-05	5	Shared with clusters 5 and 6
SLC35E2B	-0.0723296	0.079	0.149	0.00108119	5	
CLEC4E	-0.0743568	0.019	0.073	4.85E-06	5	
PTGER4	-0.0756796	0.051	0.115	0.00284978	5	
HEXIM1	-0.076298	0.145	0.215	0.0075122	5	Shared with clusters 5 and 6
GAB2	-0.0766994	0.037	0.11	0.00034042	5	Shared with clusters 2 and 5
SNAPC1	-0.0780263	0.065	0.121	0.00051723	5	Shared with clusters 2 and 5
PLSCR1	-0.0789027	0.084	0.137	5.16E-05	5	
TRIM22	-0.0802215	0.079	0.163	0.00158665	5	
VNN 2.00	-0.0807785	0.005	0.065	1.86E-28	5	Shared with clusters 5 and 7
CCNG2	-0.0809587	0.117	0.168	8.41E-15	5	Shared with clusters 2 and 5
STARD10	-0.081229	0.047	0.118	7.27E-07	5	Shared with clusters 2, 5, and 7
FCAR	-0.0815115	0.014	0.085	4.19E-35	5	Shared with clusters 2, 5, and 6
PTGS2	-0.0826787	0.009	0.053	1.81E-09	5	Shared with clusters 2 and 5
THBS1	-0.0839975	0.023	0.046	7.05E-110	5	Shared with clusters 0, 2, 5, and 7
CR1	-0.0848739	0.023	0.098	0.00019858	5	
ZNF43	-0.0855988	0.005	0.086	0.0028495	5	
IL7R	-0.0860452	0.033	0.081	4.46E-07	5	Shared with clusters 0, 2, 3, 6, and 7
DENND3	-0.0860493	0.047	0.114	1.07E-29	5	Shared with clusters 2, 5, and 6
TAGLN	-0.0863278	0	0.082	7.17E-07	5	
PHF1	-0.0870854	0.093	0.164	1.65E-06	5	
ABHD5	-0.0874049	0.164	0.192	5.30E-14	5	Shared with clusters 2, 5, and 6
PDK4	-0.0919017	0.079	0.125	0.00018681	5	
ZNF141	-0.092172	0.005	0.091	0.00504301	5	Shared with clusters 5 and 6
SERPINB2	-0.0934866	0.005	0.044	9.31E-32	5	Shared with clusters 0, 1, 2, and 5
INPP5D	-0.0936381	0.023	0.111	0.00312883	5	
ADAM28	-0.0940845	0.051	0.111	8.02E-30	5	Shared with clusters 2, 5, and 7
IRS2	-0.0948217	0.065	0.145	1.18E-08	5	
EZH1	-0.0954074	0.079	0.158	2.63E-05	5	
NFKBIL1	-0.0970169	0.084	0.172	0.00837499	5	
HIST1H1D	-0.0981595	0	0.076	5.14E-73	5	Shared with clusters 5 and 7
MTMR4	-0.0983233	0.061	0.145	0.00039812	5	Shared with clusters 5 and 7
CBX4	-0.0987533	0.033	0.124	0.00149892	5	
RAB27A	-0.0991916	0.033	0.122	3.51E-07	5	
TMEM170B	-0.1003547	0.014	0.107	4.41E-23	5	Shared with clusters 0, 2, and 5
HDAC4	-0.1007929	0.014	0.111	0.00014979	5	
PTK2B	-0.1011164	0.117	0.188	8.79E-08	5	Shared with clusters 2 and 5
MARCKSL1	-0.1024024	0.023	0.109	0.00130805	5	
ITPR2	-0.1028406	0.023	0.114	0.00704945	5	
RBM12B	-0.1038686	0.042	0.137	0.00979136	5	Shared with clusters 5 and 6
SLA	-0.1063422	0.009	0.092	9.55E-11	5	Shared with clusters 2 and 5
ZNF766	-0.1078043	0.014	0.111	0.00014829	5	
NLRP1	-0.1083858	0.005	0.103	7.50E-28	5	
SHOX2	-0.1104294	0	0.095	1.63E-06	5	
KIAA1147	-0.110581	0.019	0.114	3.68E-13	5	Shared with clusters 2 and 5
ZNF652	-0.1108799	0.042	0.142	0.00587687	5	Shared with clusters 5 and 6
VAMP2	-0.1145699	0.145	0.238	0.00018361	5	Shared with clusters 2 and 5
LEPR	-0.1159911	0.037	0.138	0.00045512	5	
TAF1	-0.1183336	0.051	0.157	0.00354796	5	Shared with clusters 2 and 5
TLR6	-0.1243212	0.051	0.159	0.00090216	5	Shared with clusters 5 and 7
TBL1X	-0.1247512	0.023	0.131	1.67E-09	5	
DAPP1	-0.1250502	0.047	0.15	0.00055614	5	
ZBTB25	-0.1262378	0.103	0.202	5.30E-08	5	Shared with clusters 2, 5, and 6
SLC25A37	-0.1266638	0.065	0.156	1.18E-10	5	Shared with clusters 2 and 5
RALGDS	-0.1272371	0.033	0.142	0.00171538	5	Shared with clusters 2 and 5
ETS2	-0.127831	0.056	0.142	8.86E-05	5	Shared with clusters 0, 2, and 5
SESTD1	-0.1278433	0.107	0.2	8.86E-08	5	Shared with clusters 0, 2, and 5
HIST1H1C	-0.1284125	0.047	0.142	1.75E-16	5	Shared with clusters 2 and 5
ARHGAP26	-0.1301654	0.056	0.165	0.00038042	5	
CDKN1C	-0.1310336	0.028	0.113	0.00088852	5	

NR1D2	-0.1327865	0.028	0.14	0.00014092	5	Shared with clusters 5 and 7
RP11-334C1	-0.1348301	0.023	0.135	3.76E-11	5	
CSF3R	-0.1377911	0.14	0.234	0.00871408	5	Shared with clusters 5 and 7
NFIL3	-0.1380573	0.065	0.174	1.69E-06	5	Shared with clusters 3 and 5
AC079767.4	-0.1381023	0.187	0.284	0.00047087	5	Shared with clusters 2 and 5
PREX1	-0.1383563	0.084	0.192	4.05E-07	5	
FUT11	-0.1393678	0.056	0.164	1.58E-07	5	Shared with clusters 2 and 5
CCL3	-0.1394661	0.626	0.609	6.29E-11	5	Shared with clusters 5 and 6
MAP3K1	-0.1396627	0.065	0.179	1.58E-05	5	
RSBN1	-0.1406988	0.107	0.221	0.00522444	5	
HAUS6	-0.1412558	0.019	0.146	0.00554655	5	Shared with clusters 2, 5, and 6
IRAK3	-0.142292	0.065	0.166	2.84E-28	5	Shared with clusters 2 and 5
PER1	-0.1458059	0.084	0.184	4.88E-08	5	Shared with clusters 2, 5, and 6
ELF2	-0.147702	0.079	0.209	0.00289064	5	
CEP95	-0.1478331	0.033	0.163	0.00231545	5	
ZNF331	-0.1481321	0.051	0.149	1.35E-21	5	Shared with clusters 2, 4, and 5
AMICA1	-0.1484147	0.023	0.132	5.13E-05	5	Shared with clusters 2 and 5
POU2F2	-0.1487136	0.042	0.148	0.00458975	5	
YIPF4	-0.1489143	0.21	0.307	6.99E-07	5	
DUSP2	-0.1497784	0.112	0.172	4.52E-13	5	Shared with clusters 4, 5, and 6
BRWD3	-0.1526534	0.033	0.167	0.00200863	5	
NPHP3	-0.1535462	0.089	0.204	6.39E-06	5	Shared with clusters 2, 5, and 7
CLMN	-0.1576294	0.065	0.169	1.81E-14	5	Shared with clusters 5 and 7
AKNA	-0.1598246	0.075	0.213	3.42E-05	5	
SOCS3	-0.1607051	0.061	0.171	1.32E-15	5	Shared with clusters 1, 2, and 5
DAGLA	-0.1609754	0.019	0.167	0.00087887	5	
HIST1H1E	-0.1615569	0.009	0.145	3.68E-21	5	Shared with clusters 1, 2, 5, and 7
EXOC3	-0.1615733	0.065	0.198	2.81E-11	5	Shared with clusters 0, 2, 5, and 7
RHOB	-0.1630763	0.173	0.236	2.71E-06	5	Shared with clusters 4 and 5
P2RX7	-0.1637562	0.037	0.173	1.63E-05	5	Shared with clusters 2, 5, 6, and 7
NBPF10	-0.1641289	0.243	0.335	2.61E-06	5	Shared with clusters 0, 2, and 5
CLEC7A	-0.1648005	0.112	0.16	2.62E-10	5	Shared with clusters 5 and 7
CHML	-0.1696004	0.042	0.177	0.00018663	5	Shared with clusters 0, 2, 5, and 7
YPEL3	-0.1697724	0.112	0.206	7.90E-25	5	
TRIM25	-0.1712099	0.051	0.19	9.40E-08	5	Shared with clusters 2 and 5
SP3	-0.1715293	0.131	0.26	0.00488122	5	Shared with clusters 0, 2, and 5
HIST2H2AC	-0.1738351	0.037	0.173	0.00035116	5	Shared with clusters 5 and 7
MXD1	-0.1767674	0.075	0.199	4.79E-08	5	Shared with clusters 2 and 5
KDM7A	-0.1795564	0.117	0.241	0.00020294	5	Shared with clusters 2 and 5
F11R	-0.1810062	0.084	0.233	1.43E-05	5	Shared with clusters 5 and 7
TTL	-0.1823044	0.028	0.188	5.08E-06	5	
ZNF516	-0.1828942	0.047	0.199	0.00299117	5	Shared with clusters 2, 5, and 7
IER2	-0.1841351	0.192	0.241	5.18E-33	5	Shared with clusters 2, 5, and 6
FAM118A	-0.187264	0.005	0.162	4.68E-12	5	Shared with clusters 5 and 6
SERPINA1	-0.1883042	0.065	0.202	0.005	5	
PKN2	-0.1902086	0.075	0.227	7.78E-05	5	Shared with clusters 2 and 5
PCYOX1	-0.1920884	0.019	0.188	0.00203584	5	
KIAA1551	-0.1922441	0.051	0.203	6.68E-09	5	Shared with clusters 0, 2, 5, and 7
SBF2	-0.1928829	0.224	0.333	0.00547334	5	
SORL1	-0.1937061	0.056	0.16	2.98E-67	5	Shared with clusters 2 and 5
CD55	-0.1941443	0.051	0.195	7.62E-09	5	Shared with clusters 2 and 5
ZCCHC11	-0.195459	0.056	0.22	4.41E-05	5	
TRPS1	-0.1958931	0.042	0.197	0.00104737	5	
KLHL24	-0.1985469	0.121	0.256	1.92E-06	5	Shared with clusters 2 and 5
HELZ	-0.19928	0.131	0.277	0.00060998	5	Shared with clusters 2 and 5
CP	-0.2002629	0	0.108	3.11E-17	5	
PCNX	-0.201295	0.033	0.197	1.85E-12	5	Shared with clusters 0, 2, and 5
CTB-61M7.2	-0.2027571	0.037	0.154	1.22E-30	5	Shared with clusters 0, 2, 5, and 7
ZNF217	-0.2087528	0.061	0.227	0.00347045	5	Shared with clusters 0 and 5
RLF	-0.2131308	0.047	0.222	3.76E-05	5	Shared with clusters 0, 2, and 5
GGA2	-0.213868	0.07	0.243	0.00036571	5	Shared with clusters 2 and 5
ANAPC7	-0.2142898	0.014	0.206	1.44E-07	5	
STK17B	-0.2157765	0.103	0.241	5.76E-07	5	
SLC36A4	-0.2168044	0.121	0.278	0.00118955	5	Shared with clusters 0, 2, and 5

SGK3	-0.218418	0.136	0.293	0.00664706	5	Shared with clusters 2 and 5
SNHG8	-0.2185818	0.182	0.314	5.09E-09	5	
CREBRF	-0.2194255	0.093	0.22	4.91E-31	5	Shared with clusters 2 and 5
MYO1G	-0.2198555	0.056	0.202	2.45E-05	5	Shared with clusters 2 and 5
PHF21A	-0.2220548	0.093	0.245	9.13E-09	5	
TSPYL1	-0.2227674	0.033	0.225	8.24E-10	5	
GCN1L1	-0.2232015	0.019	0.227	0.00840656	5	
HNRNPU-AS	-0.2232138	0.061	0.219	8.32E-10	5	Shared with clusters 2 and 5
KMT2A	-0.2265802	0.084	0.246	9.01E-15	5	
HMGB2	-0.2273747	0.243	0.354	0.0028714	5	
ZNF160	-0.230217	0.033	0.223	6.22E-06	5	Shared with clusters 2, 5, and 7
ST3GAL1	-0.2303726	0.065	0.208	2.75E-14	5	Shared with clusters 0, 2, and 5
AHRR	-0.233866	0.023	0.211	0.00013504	5	Shared with clusters 2, 5, and 7
DOCK4	-0.2353363	0.051	0.23	0.00678966	5	Shared with clusters 2 and 5
ZBTB4	-0.2392802	0.056	0.253	0.00095713	5	Shared with clusters 2 and 5
NCF1	-0.2398781	0.084	0.196	3.30E-17	5	
TIPARP	-0.2414835	0.098	0.247	0.00839903	5	
AKAP10	-0.2433756	0.075	0.262	2.37E-07	5	Shared with clusters 0, 2, and 5
RBM33	-0.2433797	0.079	0.284	2.65E-05	5	
UBA5	-0.244678	0.033	0.245	0.00964665	5	
PHC3	-0.2477537	0.061	0.255	0.00027854	5	Shared with clusters 2 and 5
BTG2	-0.24811	0.229	0.303	5.90E-20	5	Shared with clusters 2, 5, and 6
PGPEP1	-0.2515624	0.093	0.286	5.84E-05	5	Shared with clusters 5 and 7
FOXN3	-0.2524388	0.093	0.279	0.00035904	5	Shared with clusters 3 and 5
H1F0	-0.2528852	0.103	0.245	8.73E-06	5	Shared with clusters 2, 5, and 7
CELF2	-0.2612195	0.145	0.305	7.24E-08	5	Shared with clusters 2 and 5
SLC52A2	-0.2677681	0.07	0.297	0.00364581	5	Shared with clusters 5 and 6
IVNS1ABP	-0.2717529	0.192	0.364	0.00316007	5	Shared with clusters 2, 5, and 7
NF1	-0.2733174	0.065	0.279	0.00304462	5	Shared with clusters 2, 5, and 7
NAMPT	-0.2804967	0.131	0.26	8.06E-18	5	Shared with clusters 0, 2, and 5
PHF3	-0.2851573	0.093	0.291	4.67E-15	5	Shared with clusters 2 and 5
BBX	-0.285317	0.14	0.325	4.21E-05	5	Shared with clusters 2 and 5
RYBP	-0.2858945	0.117	0.301	1.86E-05	5	Shared with clusters 0, 2, 3, and 5
PDK1	-0.2880815	0.098	0.281	1.45E-06	5	Shared with clusters 2 and 5
ICAM3	-0.2923038	0.056	0.226	5.89E-22	5	Shared with clusters 3 and 5
ZNF148	-0.2924554	0.075	0.294	1.81E-05	5	Shared with clusters 5 and 6
ASH1L	-0.2945113	0.103	0.318	0.00013211	5	Shared with clusters 2, 5, and 7
FOXO3	-0.2948225	0.173	0.351	2.86E-05	5	
TM4SF19	-0.2997821	0.121	0.313	0.0014181	5	
ICAM5	-0.3032673	0.07	0.288	4.79E-05	5	Shared with clusters 5 and 7
FNDC3A	-0.3053069	0.065	0.248	1.54E-16	5	Shared with clusters 0, 2, 5, and 7
FOSL2	-0.3145871	0.224	0.333	1.53E-08	5	Shared with clusters 2, 5, and 7
EGR1	-0.3174703	0.136	0.179	2.18E-40	5	Shared with clusters 1, 2, 5, and 6
HIST1H4C	-0.3301621	0.121	0.272	2.38E-08	5	
NPEPPS	-0.3301662	0.145	0.374	0.00357879	5	
TMEM176A	-0.3305635	0.019	0.227	1.23E-12	5	
UNC50	-0.3338152	0.15	0.404	0.00160943	5	
SPHK1	-0.3389181	0.103	0.357	2.53E-05	5	
ALOX5	-0.3397659	0.019	0.261	7.35E-13	5	Shared with clusters 2 and 5
NKTR	-0.3424443	0.178	0.367	2.36E-09	5	Shared with clusters 2 and 5
GPCPD1	-0.3610008	0.187	0.389	8.92E-06	5	Shared with clusters 2 and 5
ZNF638	-0.3714974	0.136	0.369	4.56E-08	5	Shared with clusters 2 and 5
KLF2	-0.3745567	0.098	0.286	1.29E-20	5	Shared with clusters 5 and 6
TAF1D	-0.3807162	0.192	0.417	1.65E-07	5	
ANGPTL4	-0.3838902	0.042	0.199	0.00085354	5	Shared with clusters 2 and 5
SQLE	-0.3850697	0.079	0.33	7.37E-05	5	
ATM	-0.3865563	0.178	0.4	1.06E-09	5	
IGBP1	-0.3869822	0.136	0.368	0.00030252	5	
MDM2	-0.3912292	0.15	0.344	1.39E-07	5	Shared with clusters 2, 3, and 5
FAIM	-0.3968973	0.079	0.361	1.83E-05	5	Shared with clusters 2 and 5
IL8	-0.4009804	0.86	0.61	2.61E-05	5	Shared with clusters 0, 2, 4, 5, and 6
ZCCHC6	-0.4034869	0.131	0.368	4.37E-05	5	Shared with clusters 0, 5, and 7
IER5	-0.4077052	0.07	0.314	3.13E-19	5	Shared with clusters 2 and 5
MPHOSPH8	-0.4080287	0.164	0.411	1.60E-06	5	Shared with clusters 0 and 5

TET2	-0.4081393	0.061	0.344	0.00078126	5	Shared with clusters 2, 5, 6, and 7
EPB41L3	-0.4163261	0.075	0.266	1.17E-37	5	Shared with clusters 2 and 5
SMCHD1	-0.4173499	0.075	0.38	0.00014176	5	Shared with clusters 0, 2, and 5
HSPH1	-0.4204338	0.126	0.375	5.29E-10	5	
GPR183	-0.4210072	0.093	0.247	9.34E-05	5	
CPVL	-0.4277237	0.089	0.325	7.00E-11	5	Shared with clusters 4 and 5
MGEA5	-0.4297878	0.154	0.382	1.84E-12	5	Shared with clusters 2 and 5
STX7	-0.4328962	0.266	0.5	0.00376172	5	Shared with clusters 2, 5, and 6
AKAP9	-0.4344566	0.14	0.397	9.19E-07	5	
LMO4	-0.4385397	0.112	0.349	0.00018079	5	
BCAR1	-0.438982	0.126	0.387	1.26E-09	5	
ARL4C	-0.4417424	0.056	0.23	8.44E-20	5	
CXCR4	-0.4439293	0.332	0.482	4.12E-08	5	Shared with clusters 0, 2, 3, and 5
LUC7L3	-0.4480412	0.136	0.404	4.03E-10	5	Shared with clusters 2 and 5
BNIP3	-0.4500889	0.393	0.554	0.00046573	5	
DPYSL3	-0.4513789	0.065	0.326	0.00012833	5	
PHIP	-0.4524233	0.14	0.412	0.00415387	5	Shared with clusters 0, 2, and 5
C1orf63	-0.4524847	0.318	0.506	1.86E-08	5	
SF1	-0.4525953	0.22	0.489	0.0009591	5	Shared with clusters 2 and 5
ATXN1	-0.4597132	0.089	0.372	0.0014625	5	
MIS18BP1	-0.4601473	0.079	0.351	2.13E-08	5	
PNP	-0.4622032	0.112	0.418	9.98E-07	5	
SLC7A8	-0.4630796	0.121	0.379	2.24E-05	5	
CNIH1	-0.4641813	0.304	0.587	0.00211801	5	
SLC28A3	-0.4718479	0.121	0.373	1.68E-11	5	Shared with clusters 4 and 5
ARRDC3	-0.4775119	0.341	0.475	9.09E-09	5	Shared with clusters 2, 5, and 6
PCM1	-0.4777003	0.159	0.445	0.00937041	5	
TRA2B	-0.4779502	0.374	0.559	0.00338222	5	Shared with clusters 2 and 5
ALOX5AP	-0.4788471	0.084	0.26	1.16E-13	5	
CLEC2B	-0.4878243	0.234	0.398	1.49E-08	5	Shared with clusters 5 and 7
TNIP2	-0.4931729	0.131	0.445	1.40E-06	5	
FOS	-0.4935702	0.846	0.791	1.81E-05	5	Shared with clusters 1, 5, and 6
TRIM38	-0.4950691	0.107	0.439	4.25E-06	5	Shared with clusters 0, 2, and 5
MSANTD3	-0.4971086	0.098	0.439	0.0003148	5	Shared with clusters 2 and 5
VIMP	-0.499357	0.271	0.528	0.00176827	5	
NQO1	-0.5038538	0.173	0.452	0.00502208	5	
DHRS11	-0.5041487	0.178	0.45	0.00663535	5	Shared with clusters 5 and 7
SLC1A4	-0.5180404	0.229	0.484	0.00210764	5	
RTN4RL2	-0.5185769	0.089	0.333	0.00185276	5	Shared with clusters 4 and 5
IFNGR1	-0.5236184	0.276	0.507	4.92E-05	5	
BRD2	-0.5246177	0.248	0.503	3.00E-05	5	Shared with clusters 0, 2, and 5
HMGN2	-0.5369245	0.565	0.694	0.00370457	5	
SLCO4A1	-0.5386323	0.201	0.458	3.49E-06	5	
DOPEY2	-0.5424001	0.107	0.5	1.46E-05	5	Shared with clusters 5 and 7
PITPNA	-0.5439891	0.075	0.463	9.59E-05	5	
EVI2B	-0.5458116	0.21	0.418	1.08E-05	5	
HOMER3	-0.5517336	0.079	0.46	9.11E-05	5	
FAM13A	-0.5587859	0.187	0.426	1.35E-06	5	Shared with clusters 0, 2, 5, and 7
FUS	-0.5671939	0.374	0.593	0.00022143	5	Shared with clusters 0, 2, and 5
IDI1	-0.5673741	0.112	0.463	0.00143288	5	
FCGR2A	-0.5718955	0.098	0.355	2.01E-25	5	Shared with clusters 0, 5, and 7
CCNL1	-0.572436	0.36	0.552	1.72E-09	5	Shared with clusters 2, 5, and 6
IER3	-0.5806597	0.079	0.238	2.71E-20	5	Shared with clusters 0 ,2, and 5
LXN	-0.5819661	0.07	0.405	9.65E-13	5	Shared with clusters 5 and 7
CAPN2	-0.5847633	0.136	0.507	0.00447133	5	
ALDH2	-0.5850418	0.098	0.395	1.26E-06	5	Shared with clusters 0, 5, and 7
EIF4A3	-0.585349	0.14	0.526	0.00355862	5	
HAMP	-0.5940026	0.196	0.33	0.00481931	5	
DGKH	-0.5955466	0.056	0.452	9.33E-11	5	
EBP	-0.5967548	0.164	0.469	4.77E-05	5	
ZMIZ1-AS1	-0.597537	0.294	0.588	0.00020614	5	
CHD2	-0.6053716	0.159	0.492	2.63E-06	5	Shared with clusters 2, 5, and 6
MCL1	-0.6096923	0.299	0.504	3.22E-05	5	Shared with clusters 5 and 6
PALLD	-0.6184811	0.047	0.333	6.27E-17	5	

BIN1	-0.623625	0.145	0.481	0.00104132	5	
ZNF292	-0.6372054	0.136	0.473	1.41E-19	5	Shared with clusters 0, 2, 5, and 6
CYP27B1	-0.6390238	0.229	0.474	6.41E-06	5	Shared with clusters 4 and 5
TUBB6	-0.6391303	0.21	0.526	1.15E-06	5	
MXI1	-0.6439547	0.234	0.521	2.06E-10	5	Shared with clusters 0, 2, 3, and 5
MAP4	-0.6576581	0.131	0.557	0.00225819	5	
ITGB5	-0.6669998	0.117	0.459	1.72E-05	5	Shared with clusters 0, 3, and 5, and 6
ABCA1	-0.6693833	0.224	0.476	0.00154652	5	Shared with clusters 0, 2, 3, and 9
MT1H	-0.6815754	0.033	0.072	2.56E-13	5	Shared with clusters 0, 1, 3, 4, 5, and 7
TMEM70	-0.6825706	0.355	0.658	5.75E-16	5	
DDX60L	-0.7089453	0.206	0.534	0.00017363	5	
MAP3K2	-0.7150761	0.182	0.538	7.40E-07	5	Shared with clusters 0, 2, and 5
EPB41L2	-0.7203101	0.121	0.454	0.00179797	5	Shared with clusters 5 and 6
ACP2	-0.7542285	0.196	0.576	4.44E-07	5	
MARCH2	-0.7549575	0.21	0.596	0.00132843	5	Shared with clusters 0, 2, 3, 5, and 7
NDUFS8	-0.7611703	0.36	0.685	0.00133953	5	
NR1H3	-0.7634556	0.252	0.549	2.18E-05	5	Shared with clusters 5 and 7
NCS1	-0.7737597	0.079	0.568	8.42E-17	5	Shared with clusters 0, 1, 3, 4, 5, and 6
LHFPL2	-0.7904322	0.136	0.528	0.00053586	5	
FLT1	-0.7952526	0.131	0.555	7.00E-07	5	Shared with clusters 1, 3, 4, 5, and 6
MTHFS	-0.7954655	0.304	0.678	0.00272432	5	Shared with clusters 5, 6, and 7
LONP1	-0.7968621	0.15	0.617	0.00231896	5	
ASPHD1	-0.7973699	0.341	0.676	0.0094302	5	
CEBPD	-0.8039472	0.285	0.511	1.17E-08	5	Shared with clusters 5 and 7
SLC1A3	-0.8072809	0.276	0.613	0.0002037	5	
C1QB	-0.8187563	0.07	0.199	3.99E-06	5	Shared with clusters 1, 2, 5, and 6
FRMD4B	-0.8236544	0.318	0.605	1.28E-12	5	
FCGR2B	-0.8293102	0.159	0.381	9.26E-19	5	Shared with clusters 0, 5, and 7
DOT1L	-0.8294454	0.173	0.617	5.76E-09	5	
TGM2	-0.8339709	0.154	0.498	2.69E-12	5	Shared with clusters 1, 3, 4, 5, and 6
ACAT2	-0.8452128	0.131	0.471	2.95E-08	5	Shared with clusters 5 and 6
NR4A3	-0.8506106	0.121	0.434	9.42E-17	5	Shared with clusters 2, 5, 6, and 7
NDUFS6	-0.8641256	0.332	0.663	0.00036427	5	Shared with clusters 5 and 6
ZFP36L2	-0.8670948	0.388	0.554	1.93E-11	5	Shared with clusters 5 and 7
TGFBI	-0.8740284	0.224	0.405	7.41E-13	5	Shared with clusters 2, 4, 5, and 7
KCNN4	-0.8937684	0.318	0.671	0.00086023	5	Shared with clusters 5 and 7
LAT	-0.8988918	0.322	0.667	8.90E-05	5	
MAP2K3	-0.9145036	0.299	0.643	0.00099561	5	Shared with clusters 2 and 5
WSB1	-0.9218058	0.509	0.745	0.00301164	5	Shared with clusters 2 and 5
CYCS	-0.9492084	0.355	0.633	2.61E-06	5	Shared with clusters 2 and 5
CSF1	-0.9499291	0.089	0.323	2.53E-16	5	Shared with clusters 2 and 5
GSR	-0.9515509	0.145	0.623	1.54E-05	5	Shared with clusters 5 and 6
ANKRD12	-0.9600326	0.178	0.626	0.00023274	5	Shared with clusters 2 and 5
HK3	-0.9620639	0.407	0.745	0.00066287	5	Shared with clusters 0, 1, 3, 5, and 6
CHD4	-0.9628134	0.192	0.611	0.00047514	5	
C1orf122	-0.9631165	0.224	0.607	6.19E-05	5	
JAKMIP2	-0.9633622	0.084	0.534	4.06E-06	5	
DMXL2	-0.9800798	0.257	0.614	3.30E-09	5	Shared with clusters 0, 2, 5, and 7
PNISR	-0.9828073	0.439	0.742	4.01E-07	5	Shared with clusters 2 and 5
SEMA3C	-0.9847404	0.229	0.622	0.00566057	5	Shared with clusters 5 and 7
CYTIP	-0.9888604	0.262	0.54	7.47E-06	5	
MSMO1	-0.9889546	0.159	0.551	1.55E-09	5	Shared with clusters 0, 5, 6, and 7
DCXR	-0.9908098	0.416	0.718	6.09E-08	5	
MGAT4B	-1.001503	0.136	0.67	0.00384807	5	Shared with clusters 2, 5, and 7
EZR	-1.0019494	0.159	0.622	7.88E-08	5	
ATP6V0D2	-1.0059179	0.215	0.641	6.44E-10	5	
NRIP3	-1.0065158	0.276	0.687	4.43E-05	5	Shared with clusters 5 and 7
SOD1	-1.0099314	0.379	0.724	0.00561588	5	Shared with clusters 2 and 5
LILRB4	-1.0161688	0.537	0.767	1.07E-06	5	
DNAJA1	-1.0179053	0.262	0.681	5.04E-09	5	
GPI	-1.054068	0.435	0.764	0.00021424	5	
MT-ND6	-1.0672676	0.206	0.689	1.55E-05	5	
ST20	-1.07355	0.224	0.639	6.78E-17	5	Shared with clusters 0, 5, and 7
EIF4B	-1.0773383	0.192	0.605	2.22E-14	5	Shared with clusters 2 and 5

MT1G	-1.0933597	0.056	0.122	1.68E-10	5	Shared with clusters 0, 1, 3, 4, 5, 6,and 7
CD276	-1.0973691	0.257	0.721	9.83E-12	5	Shared with clusters 0, 3, 5, and 6
MAFF	-1.1000025	0.257	0.67	0.00084806	5	
BHLHE40	-1.1063176	0.355	0.689	0.0054632	5	
CALU	-1.1198858	0.322	0.697	3.31E-08	5	
HSPE1	-1.1314677	0.388	0.716	8.64E-08	5	
MRAS	-1.1409364	0.355	0.719	1.62E-08	5	Shared with clusters 0, 3, and 5
MS4A4A	-1.1507736	0.402	0.685	5.70E-05	5	Shared with clusters 0, 2, 5, and 7
PHLDA3	-1.153579	0.192	0.627	2.27E-15	5	Shared with clusters 0, 3, 5, 6, and 7
GREM1	-1.160873	0.098	0.278	1.55E-29	5	Shared with clusters 2 and 5
ASPH	-1.1645548	0.238	0.7	0.00012452	5	Shared with clusters 5 and 7
VCAN	-1.1750227	0.075	0.301	5.77E-85	5	Shared with clusters 0, 2, 5, 6, and 7
RRBP1	-1.1823822	0.257	0.699	2.24E-08	5	
NABP1	-1.1882551	0.304	0.656	0.0009869	5	
RBPJ	-1.2002465	0.364	0.72	7.07E-10	5	
NRP2	-1.200951	0.29	0.744	1.68E-08	5	Shared with clusters 5 and 7
PNRC1	-1.2013605	0.472	0.723	0.00096133	5	
C20orf24	-1.2031338	0.505	0.783	0.00082343	5	Shared with clusters 0 and 5
FOSB	-1.2275836	0.322	0.514	0.00229381	5	Shared with clusters 2, 5, and 6
CD300LB	-1.2469305	0.21	0.697	1.57E-13	5	Shared with clusters 0,1, 2, 3, and 5
FCGR3A	-1.2571814	0.238	0.554	8.19E-08	5	Shared with clusters 0, 2, 5, and 7
CTNNA1	-1.2701598	0.229	0.785	2.66E-06	5	Shared with clusters 5 and 7
EMILIN1	-1.2765651	0.168	0.557	6.75E-19	5	Shared with clusters 0, 1, 4, 5, and 6
SERPINE1	-1.2907476	0.21	0.586	9.86E-06	5	
SCG5	-1.2924513	0.257	0.405	1.17E-17	5	
SOAT1	-1.3030871	0.416	0.781	4.20E-05	5	
CYB5A	-1.3050693	0.5	0.775	1.28E-05	5	Shared with clusters 0, 2, and 5
DDX17	-1.3180846	0.266	0.73	0.00327373	5	Shared with clusters 2, 5, and 7
FAM129B	-1.3254114	0.266	0.755	2.31E-07	5	Shared with clusters 5 and 7
RAB31	-1.3355026	0.35	0.779	1.18E-05	5	
NDUFAB1	-1.3467691	0.407	0.832	0.004016	5	Shared with clusters 2 and 5
MPC2	-1.3594404	0.28	0.731	0.0001438	5	
STIP1	-1.3710919	0.22	0.667	8.29E-22	5	Shared with clusters 0, 3, 5, and 6
RHEB	-1.3714687	0.617	0.881	1.70E-07	5	
FKBP2	-1.387789	0.593	0.872	0.00038619	5	
ACTN1	-1.3992931	0.262	0.752	2.56E-07	5	Shared with clusters 0 and 6
MYO1E	-1.4033763	0.248	0.758	1.47E-24	5	
IGSF6	-1.410875	0.322	0.599	1.24E-07	5	Shared with clusters 0, 2, 5, and 6
GPC4	-1.4224856	0.421	0.642	4.36E-13	5	Shared with clusters 2, 5, and 6
CD59	-1.4293782	0.5	0.781	1.29E-09	5	Shared with clusters 0, 1, 2, 3, 5, 6, and 7
GCLC	-1.5103574	0.364	0.663	1.10E-18	5	Shared with clusters 1, 2, 3, 4, 5, 6, and 7
PEA15	-1.5108161	0.43	0.809	0.00171787	5	Shared with clusters 2, 5, 6, and 7
FUCA1	-1.5233071	0.224	0.619	0.00348409	5	Shared with clusters 3, 4, 5, and 7
RPN2	-1.5252074	0.248	0.802	0.00032247	5	Shared with clusters 0, 1, 2, 3, and 5
ATP1B3	-1.5289875	0.491	0.868	0.00931745	5	Shared with clusters 3, 5, and 7
TUBB4B	-1.5296182	0.29	0.657	2.00E-11	5	
WDR1	-1.540766	0.421	0.843	6.54E-10	5	
UPP1	-1.543342	0.285	0.741	0.00062597	5	
GLA	-1.5629264	0.332	0.745	1.18E-13	5	Shared with clusters 0, 2, 5, and 7
IFI6	-1.5666656	0.696	0.83	0.00065881	5	Shared with clusters 2. 3, 5, 6, and 7
HSD3B7	-1.5716088	0.444	0.845	5.81E-11	5	Shared with clusters 5 and 7
RNASE6	-1.5856848	0.257	0.755	2.23E-05	5	Shared with clusters 5 and 7
C1QC	-1.6314145	0.159	0.42	0.00013871	5	Shared with clusters 0, 3, and 5
TIMP3	-1.6352683	0.336	0.34	1.47E-13	5	Shared with clusters 5 and 7
TNS3	-1.6536773	0.402	0.819	0.00223238	5	
ACOT13	-1.6611269	0.411	0.837	0.00016878	5	
NCEH1	-1.6779714	0.444	0.796	9.79E-07	5	Shared with clusters 3, 5, and 7
SH3BP5	-1.69559	0.388	0.788	2.29E-11	5	
TNFSF14	-1.6964869	0.402	0.75	5.59E-10	5	Shared with clusters 5 and 7
TUBA4A	-1.700697	0.36	0.71	7.48E-10	5	Shared with clusters 2 and 5
HSPA1A	-1.7111732	0.327	0.738	5.07E-25	5	
RHOC	-1.7910752	0.673	0.857	7.81E-16	5	
ABHD2	-1.7918042	0.29	0.799	5.43E-14	5	Shared with clusters 0, 3, 5, and 7
OSBPL8	-1.8037752	0.285	0.777	0.00079972	5	

FNIP2	-1.8099142	0.551	0.831	4.10E-06	5	Shared with clusters 2, 5, and 7
NUMB	-1.8124698	0.416	0.797	0.00275351	5	Shared with clusters 2 and 5
PRKCB	-1.8181625	0.421	0.844	7.80E-08	5	Shared with clusters 0 and 5
TUBA1A	-1.8231016	0.313	0.706	8.76E-16	5	Shared with clusters 0, 5, and 6
NDUFB2	-1.8517901	0.579	0.9	2.55E-06	5	Shared with clusters 0, 2, 5, and 6
SCD	-1.8844635	0.393	0.765	3.11E-11	5	Shared with clusters 0, 2, 4, and 5
C19orf10	-1.9037531	0.621	0.886	0.00015678	5	Shared with clusters 0, 2, and 5
ITGB1BP1	-1.9062636	0.463	0.838	4.51E-13	5	Shared with clusters 0, 3, 5, and 6
FDX1	-1.9255981	0.607	0.833	4.24E-08	5	Shared with clusters 0, 2, 3, 5, and 6
IRAK1	-1.9359678	0.252	0.834	2.54E-10	5	
CCNI	-1.9795842	0.421	0.841	3.91E-10	5	
YWHAQ	-1.992403	0.593	0.914	4.70E-07	5	
CD83	-1.9955974	0.556	0.855	9.70E-16	5	
PTGR1	-2.021239	0.178	0.73	5.24E-24	5	Shared with clusters 0, 1, 3, 5, and 6
ACADVL	-2.0245644	0.458	0.918	0.00061627	5	
FKBP1A	-2.0326693	0.724	0.939	0.00452846	5	Shared with clusters 0, 1, 2, 3, 5, and 6
TUBA1C	-2.0470238	0.364	0.833	0.00286953	5	Shared with clusters 2 and 5
TMEM91	-2.0620828	0.607	0.868	2.53E-05	5	Shared with clusters 2, 5, and 7
CRABP2	-2.0994127	0.472	0.546	2.37E-24	5	
SEC61B	-2.151646	0.575	0.931	1.84E-10	5	
SLC7A11	-2.1559585	0.262	0.711	1.78E-06	5	Shared with clusters 5 and 7
PIK3AP1	-2.1602832	0.481	0.881	6.14E-16	5	Shared with clusters 5 and 7
TCEB1	-2.1762718	0.589	0.898	1.97E-16	5	
TKT	-2.2348653	0.346	0.845	1.01E-09	5	Shared with clusters 3 and 5
PGD	-2.2356107	0.617	0.931	0.005596	5	Shared with clusters 5 and 7
MGAT1	-2.2398208	0.748	0.955	0.00053095	5	Shared with clusters 1 and 5
MMP19	-2.2682636	0.5	0.833	1.13E-16	5	
CYSTM1	-2.2934956	0.701	0.919	4.92E-12	5	Shared with clusters 0, 2, 3, 5, 6, and 7
HM13	-2.2964116	0.64	0.919	6.54E-05	5	Shared with clusters 5 and 7
ATP6V1A	-2.3453439	0.467	0.918	2.59E-15	5	Shared with clusters 5 and 7
MYOF	-2.4049694	0.173	0.882	1.52E-28	5	Shared with clusters 0, 3, 5, and 7
ACTR2	-2.425676	0.444	0.923	0.00837386	5	
MSN	-2.4358449	0.341	0.91	6.89E-18	5	
EMP3	-2.4914938	0.668	0.901	0.00073065	5	
ATP6V1D	-2.5184254	0.584	0.878	4.66E-11	5	Shared with clusters 3, 5, 6, and 7
INSIG1	-2.5265548	0.397	0.803	6.17E-16	5	Shared with clusters 2 and 5
EREG	-2.5711501	0.248	0.362	4.75E-19	5	Shared with clusters 1, 2, 5, 6, and 7
CAP1	-2.5786734	0.551	0.938	1.52E-08	5	
FAH	-2.5912095	0.593	0.836	9.62E-09	5	Shared with clusters 5, 6, and 7
EMP1	-2.6074439	0.706	0.932	2.47E-25	5	Shared with clusters 0, 1, 3, 5, and 6
ITGAX	-2.6209179	0.528	0.936	0.00299802	5	Shared with clusters 5 and 7
RP11-20G13	-2.6214175	0.336	0.546	1.10E-24	5	Shared with clusters 2 and 5
ATF3	-2.6396668	0.598	0.84	3.88E-20	5	Shared with clusters 5 and 7
G6PD	-2.7009428	0.664	0.908	6.17E-20	5	Shared with clusters 0, 1, 2, 3, 4, 5, 6, and 7
YWHAB	-2.710391	0.463	0.924	1.17E-11	5	Shared with clusters 5 and 6
ATP1A1	-2.7487939	0.477	0.929	1.64E-11	5	
HSPA8	-2.7793623	0.472	0.857	1.81E-15	5	Shared with clusters 0, 3, and 5
ASAP1	-2.9276786	0.332	0.939	2.52E-13	5	Shared with clusters 3 and 5
COX6C	-2.9382203	0.855	0.968	2.14E-05	5	Shared with clusters 0, 1, 2, 5, and 6
S100A9	-2.9823446	0.463	0.764	0.00061811	5	Shared with clusters 0, 1, 2, 3, 5, 6, and 7
C3	-2.9949339	0.762	0.922	5.43E-09	5	Shared with clusters 0, 3, 4, 5, 6, and 7
MTRNR2L12	-3.0032845	0.21	0.856	2.55E-15	5	Shared with clusters 2, 5, and 6
DYNLL1	-3.0280497	0.701	0.915	5.79E-13	5	Shared with clusters 5 and 6
HMOX1	-3.0466389	0.509	0.831	1.94E-14	5	Shared with clusters 5 and 7
TM4SF19.1	-3.0579873	0.533	0.787	1.53E-39	5	Shared with clusters 2, 4, and 5
PLEKHO1	-3.1523954	0.421	0.883	1.82E-13	5	Shared with clusters 1, 2, 5, 6, and 7
DAD1	-3.1943245	0.626	0.954	1.05E-15	5	Shared with clusters 1, 2, and 5
APOC1	-3.2165751	0.986	0.962	4.01E-22	5	Shared with clusters 0, 1, 2, 3, 4, 5, 6, and 7
CXCL5	-3.2565711	0.065	0.215	0.00273563	5	Shared with clusters 0, 2, 3, 5, and 7
MATK	-3.3174048	0.575	0.923	9.54E-29	5	Shared with clusters 0, 3, 4, 5, 6, and 7
GUSB	-3.3897221	0.65	0.936	6.92E-07	5	Shared with clusters 0, 1, 2, 3, 5, 6, and 7
ATP5G3	-3.4762792	0.86	0.975	0.00122855	5	Shared with clusters 0, 5, and 6
RPSA	-3.5559478	0.738	0.97	2.28E-10	5	Shared with clusters 0, 1, 5, and 6
MT1X	-3.6037948	0.229	0.28	3.46E-24	5	Shared with clusters 0, 1, 3, 4, 5, 6,and 7

MGLL	-3.6330936	0.701	0.913	5.07E-19	5	Shared with clusters 2, 3, 4, 5, 6, and 7
ANXA11	-3.6442127	0.668	0.98	5.20E-11	5	Shared with clusters 0, 2, 3, and 5
TPM4	-3.6603651	0.575	0.878	3.11E-14	5	Shared with clusters 3, 5, and 7
H3F3B	-3.6684987	0.963	0.996	2.18E-07	5	Shared with clusters 2, 5, and 6
NDUFS5	-3.7084866	0.864	0.986	6.38E-18	5	Shared with clusters 2, 5, and 6
BCL2A1	-3.7230786	0.72	0.937	1.54E-22	5	Shared with clusters 0, 2, 3, 5, 6, and 7
KIAA0930	-3.7706103	0.491	0.955	3.52E-16	5	Shared with clusters 0, 2, 3, 5, and 7
PTPN6	-3.7886917	0.654	0.965	9.38E-19	5	Shared with clusters 0, 3, 5, 6, and 7
LPL	-3.7904199	0.612	0.77	3.45E-33	5	Shared with clusters 2, 4, and 5
CCDC88A	-3.9151957	0.505	0.969	3.99E-14	5	Shared with clusters 3, 5, and 7
EEF1B2	-3.9202413	0.584	0.974	7.85E-07	5	Shared with clusters 0, 5, and 6
COX5B	-3.9317044	0.953	0.989	9.00E-14	5	Shared with clusters 0, 2, 5, and 6
PPP1R14B	-3.9501053	0.537	0.95	6.55E-14	5	Shared with clusters 5, 6, and 7
RPS5	-4.0017242	0.85	0.986	3.86E-05	5	Shared with clusters 0, 1, and 5
ARPC5	-4.0266941	0.762	0.98	7.00E-10	5	Shared with clusters 2, 5, and 6
TXNIP	-4.0284961	0.607	0.899	1.14E-06	5	Shared with clusters 0, 2, 3, and 5
DBI	-4.0318543	0.827	0.974	7.15E-11	5	Shared with clusters 0, 2, 5, and 6
LMNA	-4.1069319	0.556	0.971	4.84E-25	5	Shared with clusters 5 and 7
C15orf48	-4.1452816	0.724	0.948	6.32E-19	5	Shared with clusters 5 and 7
TGFB1	-4.1850238	0.551	0.965	3.72E-26	5	Shared with clusters 0, 1, 2, 3, 5, and 6
GM2A	-4.2447599	0.729	0.939	3.41E-10	5	Shared with clusters 0, 2, 3, 5, 6, and 7
CD84	-4.2503215	0.5	0.953	1.52E-37	5	Shared with clusters 0, 1, 3, 5, 6, and 7
FAM20C	-4.3049588	0.528	0.969	1.35E-39	5	Shared with clusters 0, 1, 2, 3, 4, 5, 6, and 7
FERMT3	-4.3437098	0.612	0.971	6.66E-24	5	Shared with clusters 0, 1, 3, 5, 6, and 7
ALCAM	-4.420057	0.575	0.941	1.28E-39	5	Shared with clusters 0, 1, 2, 3, 4 ,5, 6, and 7
ATP6V0B	-4.4818859	0.86	0.975	2.55E-09	5	Shared with clusters 5 and 7
MYL12A	-4.4906911	0.724	0.986	6.47E-13	5	Shared with clusters 0, 5, and 6
CD81	-4.5786939	0.285	0.96	3.59E-09	5	Shared with clusters 0, 1, 2, 5, and 6
PSMA7	-4.6916297	0.771	0.977	2.06E-16	5	Shared with clusters 0, 3, 5, and 6
TIMP2	-4.7168617	0.505	0.994	0.00014744	5	Shared with clusters 0, 2, 5, and 7
TYMP	-4.7931025	0.664	0.966	1.86E-19	5	Shared with clusters 3, 5, and 7
LSP1	-4.8582404	0.883	0.987	0.00028171	5	Shared with clusters 5 and 7
ATP6AP2	-4.875642	0.766	0.986	0.0035284	5	Shared with clusters 0, 1, 2, 3, 5, and 7
PLEK	-5.1013662	0.668	0.93	3.77E-18	5	Shared with clusters 0, 5, 6, and 7
HMG20B	-5.2297951	0.687	0.943	8.95E-31	5	Shared with clusters 0, 1, 3, 4, 5, and 7
ACP5	-5.3055075	0.748	0.948	2.95E-11	5	Shared with clusters 0, 2, 5, and 7
LIMS1	-5.3452456	0.696	0.981	5.46E-13	5	Shared with clusters 1, 5, and 7
ANXA1	-5.3846233	0.743	0.971	0.00196203	5	
NPC2	-5.4535495	1	1	3.00E-05	5	Shared with clusters 3 and 5
CYP1B1	-5.6263157	0.486	0.851	2.38E-10	5	Shared with clusters 0, 1, 2, 3, 5, and 7
ZFP36L1	-5.6656196	0.794	0.933	1.72E-17	5	Shared with clusters 0, 2, 5, 6, and 7
TXNRD1	-5.9758369	0.514	0.943	1.15E-62	5	Shared with clusters 0, 1, 3, 4, 5, 6, and 7
H2AFY	-6.1355714	0.939	0.996	9.27E-05	5	Shared with clusters 5 and 7
HSP90AA1	-6.4009641	0.715	0.986	4.83E-23	5	
TALDO1	-6.4193567	0.79	0.988	7.39E-11	5	Shared with clusters 2, 5, 6, and 7
CYP27A1	-6.4761482	0.748	0.982	2.49E-08	5	Shared with clusters 2, 5, and 6
MMP7	-6.6417104	0.299	0.296	3.09E-09	5	Shared with clusters 2, 5, 6, and 7
RPL21	-6.6826566	0.935	0.999	0.0015115	5	Shared with clusters 0, 2, and 5
LAPTM5	-6.7137902	0.949	0.999	2.61E-05	5	
PLA2G7	-6.8158035	0.813	0.98	0.00379064	5	Shared with clusters 5 and 7
CFL1	-6.9302219	0.902	0.993	3.74E-16	5	Shared with clusters 0, 1, 2, 3, 5, 6, and 7
TIMP1	-7.0483467	0.888	0.956	1.13E-06	5	Shared with clusters 2, 3, 5, 6, and 7
PRDX1	-7.2849566	0.822	0.989	3.85E-17	5	Shared with clusters 0, 1, 2, 3, 5, 6, and 7
ANXA5	-7.3769279	0.734	0.989	2.50E-22	5	Shared with clusters 1, 5, 6, and 7
GSTO1	-7.5113034	0.841	0.996	5.45E-42	5	Shared with clusters 0, 3, 5, 6, and 7
CAPG	-7.5373668	0.832	0.975	4.79E-19	5	Shared with clusters 0, 1, 2, 3, 5, 6, and 7
CD9	-7.5577375	0.855	0.993	1.21E-07	5	Shared with clusters 1, 2, 5, and 7
TXN	-7.7843014	0.874	0.996	1.39E-22	5	Shared with clusters 0, 2, 3, 5, and 6
CTSL	-8.1292726	0.818	0.931	4.41E-22	5	Shared with clusters 0, 1, 2, 3, 5, 6, and 7
TUBA1B	-8.1739948	0.813	0.973	1.35E-17	5	Shared with clusters 0, 1, 3, 5, 6, and 7
MT2A	-8.4492944	0.477	0.522	0.00088972	5	Shared with clusters 0, 2, 1, 3, 4, 5, 6, and 7
RPS3	-9.0543055	0.935	1	2.18E-06	5	Shared with clusters 0, 5, 6, and 7
ARPC1B	-9.3643959	0.893	0.996	2.45E-18	5	Shared with clusters 0, 1, 2, 3, 5, 6, and 7
ANXA2	-9.5065036	0.888	0.997	1.42E-16	5	Shared with clusters 0, 1, 2, 3, 5, 6, and 7

APOE	-9.8228231	0.972	0.952	1.16E-17	5	Shared with clusters 0, 2, 3, 5, 6, and 7
GSN	-10.105744	0.794	0.993	3.78E-43	5	Shared with clusters 0, 1, 2, 3, 5, 6, and 7
FLNA	-10.521251	0.701	0.994	5.48E-37	5	Shared with clusters 1, 3, 5, and 6
FABP4	-10.546913	0.481	0.434	6.04E-17	5	Shared with clusters 0, 1, 2, 3, 4, 5, 6, and 7
FBP1	-10.813416	0.706	0.99	1.52E-24	5	Shared with clusters 0, 1, 2, 3, 5, 6, and 7
MYL6	-10.970759	0.986	0.999	3.61E-10	5	Shared with clusters 5 and 6
MT-ND5	-11.638336	0.869	0.998	7.72E-07	5	
IL1RN	-12.113923	0.879	0.947	1.54E-60	5	Shared with clusters 0, 1, 3, 4, 5 6, and 7
RPS12	-12.441652	0.935	0.998	5.83E-06	5	Shared with clusters 0, 4, 5, and 6
LIPA	-12.696655	0.967	0.991	3.93E-60	5	Shared with clusters 0, 1, 2, 3, 4, 5, 6, and 7
RPL7A	-12.757239	0.925	1	4.34E-06	5	Shared with clusters 0, 1, 4, 5, 6, and 7
RGCC	-14.263693	0.958	0.995	1.46E-51	5	Shared with clusters 0, 1, 5, 6, and 7
RPL13	-15.165558	1	1	0.00460607	5	Shared with clusters 0, 1, 4, 5, and 6
MFSD12	-15.581041	0.776	0.986	4.42E-108	5	Shared with clusters 0, 1, 2, 3,4, 5, 6, and 7
CD63	-26.78132	0.995	1	4.02E-11	5	Shared with clusters 0, 1, 2, 3, 4, 5, 6, and 7
S100A6	-28.096325	1	1	3.71E-19	5	Shared with clusters 1, 2, 5, and 6
CHI3L1	-29.390914	0.72	0.851	2.41E-76	5	Shared with clusters 0, 1, 2, 3, 4, 5, 6, and 7
MT-ND1	-29.796625	0.879	1	8.08E-06	5	Shared with clusters 0, 2, and 5
ACTB	-30.796158	0.972	1	1.03E-18	5	Shared with clusters 0, 1, 3, 5, 6, and 7
EEF1A1	-31.241828	0.991	1	0.00115816	5	Shared with clusters 2 and 5
S100A11	-33.948209	1	1	7.63E-17	5	Shared with clusters 1, 5, 6, and 7
LGALS3	-34.960475	0.995	1	4.45E-16	5	Shared with clusters 0, 1, 2, 3, 4, 5, 6, and 7
MT-ND3	-35.1198	0.897	0.998	1.83E-07	5	Shared with clusters 5 and 6
MMP9	-36.493124	0.953	0.996	7.23E-28	5	Shared with clusters 0, 1, 2, 3, 4, 5, 6, and 7
FABP5	-41.286451	0.991	0.993	1.38E-207	5	Shared with clusters 0, 1, 2, 3, 4, 5, 6, and 7
MT-CO3	-53.465692	0.991	1	0.00165245	5	Shared with clusters 0, 5, and 6
MT-ND4	-63.921499	1	1	2.80E-24	5	Shared with clusters 0, 2, 3, 5, and 7
MT-CO1	-71.740521	0.995	1	1.02E-15	5	Shared with clusters 0, 1, 2, 3, and 5
CSTB	-71.791976	1	1	2.32E-38	5	Shared with clusters 0, 1, 5, 6, and 7
SPP1	-247.77286	1	0.998	1.64E-167	5	Shared with clusters 0, 1, 2, 3, 4, 5, 6, and 7
RPL17	1.551053	0.964	0.646	1.52E-10	6	Shared with clusters 2 and 6
RPL39	0.73739909	1	0.998	0.00252148	6	Shared with clusters 0 and 2
FABP4	0.0539663	0.171	0.136	1.48E-17	6	Shared with clusters 0, 1, 2, 3, 4, 5, 6, and 7
NCS1	-0.0276413	0	0.016	6.43E-25	6	Shared with clusters 0, 1, 3, 4, 5, and 6
GPC4	-0.0285539	0.021	0.031	1.67E-13	6	Shared with clusters 2, 5, and 6
LTB	-0.0307125	0	0.015	6.44E-68	6	Shared with clusters 2, 6, and 7
SLC52A2	-0.031941	0	0.022	0.00066881	6	Shared with clusters 5 and 6
SDF2L1	-0.0331695	0	0.021	1.12E-07	6	
ZNF84	-0.0337838	0	0.02	0.00236769	6	
MTHFS	-0.033854	0.014	0.034	0.00309506	6	Shared with clusters 5, 6, and 7
NR4A3	-0.034398	0	0.019	1.33E-38	6	Shared with clusters 2, 5, 6, and 7
S100A8	-0.0346086	0.029	0.049	1.08E-15	6	Shared with clusters 1, 2, and 6
MMP7	-0.035451	0.029	0.044	1.19E-23	6	Shared with clusters 2, 5, 6, and 7
FDX1	-0.0371183	0.093	0.097	1.19E-08	6	Shared with clusters 0, 2, 3, 5, and 6
AP000769.1	-0.0386978	0	0.02	0.00143828	6	
ACAT2	-0.0413829	0.007	0.029	1.98E-07	6	Shared with clusters 5 and 6
FLT1	-0.0416111	0.007	0.035	5.00E-05	6	Shared with clusters 1, 3, 4, 5, and 6
HK3	-0.04298	0.036	0.061	0.00209004	6	Shared with clusters 0, 1, 3, 5, and 6
NDUFS6	-0.0471393	0.007	0.035	2.27E-05	6	Shared with clusters 5 and 6
SLAMF7	-0.0479115	0	0.028	2.02E-11	6	Shared with clusters 3 and 6
CCDC142	-0.0497543	0	0.03	0.00217712	6	
NKIRAS1	-0.0503686	0	0.032	0.00336658	6	
TGM2	-0.0509828	0	0.026	1.47E-27	6	Shared with clusters 1, 3, 4, 5, and 6
CCL5	-0.0516673	0.021	0.024	6.04E-10	6	Shared with clusters 0, 6, and 7
CAB39L	-0.0522113	0	0.031	2.02E-05	6	
RAMP1	-0.0524395	0.007	0.029	3.37E-18	6	Shared with clusters 1, 2, and 6
IL7R	-0.0528256	0	0.015	5.06E-61	6	Shared with clusters 0, 2, 3, 6, and 7
LRRC37A3	-0.0528256	0	0.033	0.00410069	6	
CD276	-0.053668	0.007	0.039	1.62E-05	6	Shared with clusters 0, 3, 5, and 6
LDHB	-0.0553528	0.021	0.048	0.00033298	6	Shared with clusters 0 and 6
PHLDA3	-0.0557389	0.007	0.041	2.35E-07	6	Shared with clusters 0, 3, 5, 6, and 7
FAM101B	-0.056125	0.007	0.034	4.98E-12	6	Shared with clusters 6 and 7
RP11-206L1	-0.0565111	0	0.031	0.00116044	6	
TMCO6	-0.0583538	0	0.034	0.00153531	6	

GCLC	-0.060337	0.029	0.048	2.45E-10	6	Shared with clusters 1, 2, 3, 4, 5, 6, and 7
ZNF440	-0.0614251	0	0.036	0.00760355	6	
GTPBP1	-0.0620393	0	0.037	0.00088047	6	
PSME2	-0.0626536	0	0.036	2.46E-17	6	
C9orf41	-0.0638821	0	0.039	0.00692891	6	
MLLT11	-0.0638821	0	0.036	0.00140475	6	
AARSD1	-0.0663391	0	0.039	3.60E-06	6	
PRICKLE3	-0.0663391	0	0.038	0.00037086	6	
CDH23	-0.0669533	0	0.036	3.04E-08	6	Shared with clusters 2, 6, and 7
RP11-468E2	-0.0675676	0	0.037	0.00019894	6	
MOB3C	-0.0687961	0	0.041	0.00016368	6	
EVI2A	-0.0688487	0.879	0.922	0.00831017	6	Shared with clusters 0 and 6
RP4-798A10	-0.0700246	0	0.042	0.00025166	6	
AMT	-0.0712531	0	0.042	0.00029885	6	
RP11-680A1	-0.0718673	0	0.038	5.07E-07	6	
AC016586.1	-0.0724816	0	0.041	0.00849155	6	
HIST1H2BN	-0.0730958	0	0.033	5.35E-06	6	
STIP1	-0.0755528	0	0.048	1.16E-57	6	Shared with clusters 0, 3, 5, and 6
PCSK5	-0.0755528	0	0.048	0.00085707	6	
CCND2	-0.0804668	0	0.044	3.26E-13	6	Shared with clusters 0, 3, and 6
ZNF347	-0.0804668	0	0.048	0.00031045	6	
MSMO1	-0.0835381	0	0.043	8.52E-35	6	Shared with clusters 0, 5, 6, and 7
AGAP5	-0.0835381	0	0.047	0.00019187	6	
ZNF182	-0.0835381	0	0.052	0.0082766	6	
ZNF738	-0.0847666	0	0.05	1.00E-05	6	
CD59	-0.0850474	0.057	0.085	0.00016565	6	Shared with clusters 0, 1, 2, 3, 5, 6, and 7
EPB41L2	-0.0859951	0	0.052	8.74E-08	6	Shared with clusters 5 and 6
MAML3	-0.0859951	0	0.05	2.24E-06	6	
SH3BP5-AS	-0.0878378	0	0.052	1.05E-05	6	
PEA15	-0.0895051	0.043	0.079	0.00098175	6	Shared with clusters 2, 5, 6, and 7
VENTX	-0.0896806	0	0.047	0.00108083	6	Shared with clusters 0, 2, and 6
CD180	-0.0923657	0.007	0.061	0.00418887	6	
ITGB1BP1	-0.0934363	0.014	0.063	2.57E-10	6	Shared with clusters 0, 3, 5, and 6
C1QB	-0.0952088	0	0.035	2.19E-30	6	Shared with clusters 1, 2, 5, and 6
KIAA1683	-0.0958231	0	0.052	6.98E-06	6	
ARHGEF10	-0.0976658	0	0.053	1.23E-05	6	
KPNA5	-0.0976658	0	0.055	0.00037598	6	
IL1RAP	-0.0995086	0	0.053	2.86E-05	6	Shared with clusters 0 and 6
MCCC1	-0.1007371	0	0.058	1.46E-05	6	
MFSD8	-0.1021938	0.007	0.063	0.0031536	6	
TUBGCP6	-0.1025799	0	0.058	9.02E-06	6	
CRTC1	-0.1038084	0	0.061	3.63E-06	6	
HERC3	-0.1044226	0	0.063	3.37E-08	6	
PTGR1	-0.1068621	0.036	0.087	0.00019415	6	Shared with clusters 0, 1, 3, 5, and 6
EMILIN1	-0.1068796	0	0.051	1.93E-31	6	Shared with clusters 0, 1, 4, 5, and 6
METTL4	-0.1074939	0	0.066	0.00020587	6	
SLC25A28	-0.1074939	0	0.066	0.00244466	6	
TAF10	-0.1075641	0.007	0.072	3.25E-07	6	Shared with clusters 0, 1, 2, and 6
C22orf46	-0.1081081	0	0.057	0.00030287	6	
MIR29A	-0.1081081	0	0.066	0.00033234	6	
NOD2	-0.1081081	0	0.066	7.09E-12	6	
TTN-AS1	-0.1087224	0	0.061	3.50E-07	6	
FAM8A1	-0.1105651	0	0.068	0.00110352	6	
RDH13	-0.1124079	0	0.063	8.48E-06	6	
TUBD1	-0.1130221	0	0.064	9.97E-11	6	
HELB	-0.1148649	0	0.064	1.43E-11	6	
ODC1	-0.1148649	0	0.06	7.21E-05	6	
RP11-1407O	-0.122464	0.007	0.075	0.00794182	6	
RP11-611L7	-0.1228501	0	0.067	2.09E-07	6	
FAM120B	-0.1246929	0	0.071	5.15E-09	6	
NAA15	-0.1253071	0	0.075	0.00141136	6	
RP11-426C2	-0.1259214	0	0.073	2.70E-08	6	
ZNF407	-0.1271499	0	0.067	0.00130377	6	
CST7	-0.1316778	0.007	0.064	0.00068446	6	Shared with clusters 6 and 7

TUBA1A	-0.1321867	0.05	0.098	6.69E-06	6	Shared with clusters 0, 5, and 6
SCAF1	-0.1369779	0	0.075	3.50E-05	6	
GSR	-0.1375921	0	0.072	6.37E-10	6	Shared with clusters 5 and 6
MFN1	-0.1375921	0	0.079	0.00011018	6	
G6PD	-0.1403299	0.05	0.139	1.47E-18	6	Shared with clusters 0, 1, 2, 3, 4, 5, 6, and 7
CNPPD1	-0.1408915	0.007	0.078	0.00099921	6	
RAB11FIP2	-0.1449631	0	0.08	3.76E-07	6	
EFEMP2	-0.1474201	0	0.08	1.28E-12	6	
PEX1	-0.1498771	0	0.082	3.48E-05	6	Shared with clusters 2, 5, and 6
MZF1	-0.1611618	0.007	0.093	0.00025305	6	
MNDA	-0.1651808	0.086	0.195	4.13E-05	6	Shared with clusters 0, 2, 6, and 7
ATAD2	-0.1670762	0	0.087	6.05E-08	6	
HIP1	-0.1738329	0	0.093	2.51E-07	6	Shared with clusters 2 and 6
TNFRSF10D	-0.1771323	0.007	0.106	3.41E-05	6	
ATP6V1D	-0.1806423	0.05	0.166	1.83E-07	6	Shared with clusters 3, 5, 6, and 7
FGD6	-0.1816602	0.007	0.105	0.00048265	6	
ZC3H18	-0.1842752	0	0.097	2.66E-08	6	
KLHL8	-0.1869603	0.007	0.102	0.00558961	6	
IGSF6	-0.1945419	0.043	0.131	8.26E-07	6	Shared with clusters 0, 2, 5, and 6
ZNF506	-0.198245	0.007	0.117	0.00484453	6	
RP11-1114A	-0.1984029	0	0.101	2.11E-21	6	Shared with clusters 5 and 6
EMP1	-0.1984205	0.121	0.213	9.72E-05	6	Shared with clusters 0, 1, 3, 5, and 6
MT1E	-0.2014918	0.079	0.135	4.47E-11	6	Shared with clusters 0, 1, 2, 3, 4, 6,and 7
HIST2H2BE	-0.2046157	0.021	0.103	0.00010211	6	
CNOT3	-0.2047736	0.007	0.114	1.35E-05	6	
IL4R	-0.2050018	0.007	0.117	7.67E-06	6	
MATK	-0.2125132	0.021	0.137	2.88E-16	6	Shared with clusters 0, 3, 4, 5, 6, and 7
FPR1	-0.2143735	0	0.092	0.00184985	6	Shared with clusters 5 and 6
TRAK1	-0.2143735	0	0.115	3.96E-07	6	Shared with clusters 6 and 7
SS18L1	-0.2149877	0	0.113	8.29E-21	6	
SLC20A1	-0.2162864	0.014	0.112	0.00111152	6	
CCL3	-0.2224816	0.593	0.516	6.41E-08	6	Shared with clusters 5 and 6
FAM73A	-0.2234293	0.007	0.126	0.00068698	6	
ZNF141	-0.2272727	0	0.122	2.16E-13	6	Shared with clusters 5 and 6
ACVR2B	-0.2287294	0.007	0.123	0.00037933	6	Shared with clusters 5 and 6
N4BP2	-0.2351	0.007	0.123	0.00182318	6	
FAM193A	-0.2369428	0.007	0.127	0.00031651	6	
TNF	-0.2492278	0.007	0.079	1.01E-06	6	
FHAD1	-0.2518428	0	0.12	6.59E-14	6	
TMSB10	-0.2609337	1	1	0.00045104	6	Shared with clusters 2 and 6
SIK1	-0.2639698	0.014	0.118	0.00066297	6	Shared with clusters 2 and 6
AC132872.2	-0.2678133	0	0.111	2.14E-06	6	
CD93	-0.2686557	0.007	0.13	0.00033775	6	Shared with clusters 0, 2, 5, and 6
MYO15B	-0.2949105	0.014	0.149	0.00025388	6	Shared with clusters 6 and 7
GOLGA8A	-0.2988241	0.014	0.144	0.00453869	6	Shared with clusters 2, 6, and 7
PLBD1	-0.3055809	0.014	0.18	0.00105618	6	Shared with clusters 0, 1, 2, and 6
ZNF652	-0.3063531	0.007	0.158	0.00323484	6	Shared with clusters 5 and 6
SNRNP48	-0.3253949	0.014	0.165	0.00745444	6	Shared with clusters 1 and 6
ZBTB20	-0.3399789	0.021	0.174	0.005422	6	Shared with clusters 5, 6, and 7
BTAF1	-0.3465075	0.014	0.186	0.00036791	6	
MGLL	-0.3671464	0.05	0.238	3.04E-12	6	Shared with clusters 2, 3, 4, 5, 6, and 7
CDC16	-0.3676202	0.021	0.186	0.00760692	6	
RBM12B	-0.374377	0.014	0.195	5.49E-05	6	Shared with clusters 5 and 6
CYSTM1	-0.3814321	0.043	0.328	1.62E-14	6	Shared with clusters 0, 2, 3, 5, 6, and 7
NDUFB2	-0.392822	0.129	0.475	2.25E-05	6	Shared with clusters 0, 2, 5, and 6
NFXL1	-0.3958055	0.007	0.15	9.12E-07	6	Shared with clusters 0, 2, 5, 6, and 7
HAUS6	-0.4077747	0.021	0.173	0.00189864	6	Shared with clusters 2, 5, and 6
C3	-0.4098456	0.029	0.272	7.48E-12	6	Shared with clusters 0, 3, 4, 5, 6, and 7
CBX7	-0.4196034	0.007	0.204	9.08E-10	6	Shared with clusters 5 and 6
APOC1	-0.4220428	0.821	0.972	5.45E-21	6	Shared with clusters 0, 1, 2, 3, 4, 5, 6, and 7
HMGA1	-0.4309582	0.036	0.357	0.00186474	6	Shared with clusters 0 and 6
SLC25A5	-0.4357318	0.007	0.348	1.01E-19	6	Shared with clusters 0, 2, and 6
BCL2A1	-0.4473675	0.057	0.416	3.93E-18	6	Shared with clusters 0, 2, 3, 5, 6, and 7
INTS6	-0.4480168	0.007	0.197	8.36E-13	6	

MT1G	-0.4558617	0.086	0.212	2.87E-06	6	Shared with clusters 0, 1, 3, 4, 5, 6,and 7
RPL13A	-0.45788	0.993	0.994	3.20E-11	6	Shared with clusters 6 and 7
C9orf16	-0.4638645	0.079	0.486	7.39E-15	6	Shared with clusters 0, 2, and 6
IFI6	-0.4690418	0.029	0.314	1.19E-13	6	Shared with clusters 2. 3, 5, 6, and 7
FKBP1A	-0.4750965	0.05	0.434	1.98E-07	6	Shared with clusters 0, 1, 2, 3, 5, and 6
LPAR2	-0.5015269	0.021	0.236	0.00671353	6	Shared with clusters 2, 6, and 7
PFN1	-0.5137417	0.671	0.947	0.00010464	6	
COX6C	-0.5149351	0.321	0.793	6.94E-10	6	Shared with clusters 0, 1, 2, 5, and 6
ALCAM	-0.5173921	0.064	0.359	1.01E-08	6	Shared with clusters 0, 1, 2, 3, 4 ,5, 6, and 7
IFI30	-0.5302913	0.164	0.277	3.05E-12	6	Shared with clusters 5 and 6
HEXIM1	-0.5320639	0.043	0.203	0.00177103	6	Shared with clusters 5 and 6
PPT1	-0.5527027	0.086	0.424	8.33E-05	6	Shared with clusters 0, 1, 2, 3, 6, and 7
PDIA6	-0.5561425	0.121	0.43	0.00994618	6	Shared with clusters 1, 2, and 6
HSPA8	-0.5650755	0.064	0.458	1.81E-10	6	
NDUFS5	-0.5749561	0.529	0.915	1.58E-08	6	Shared with clusters 2, 5, and 6
TREM2	-0.5913829	0.107	0.532	1.95E-10	6	Shared with clusters 0, 2, 3, 6, and 7
FAH	-0.6008775	0.029	0.468	1.87E-07	6	Shared with clusters 5, 6, and 7
FCAR	-0.6081783	0.021	0.248	8.35E-12	6	Shared with clusters 2, 5, and 6
DDI2	-0.6135311	0.064	0.281	0.002235	6	Shared with clusters 2 and 6
HLA-DMB	-0.6250439	0.064	0.276	0.00193607	6	Shared with clusters 2 and 6
COX5B	-0.6336609	0.714	0.955	6.44E-09	6	Shared with clusters 0, 2, 5, and 6
P2RX7	-0.6341348	0.007	0.265	3.04E-20	6	Shared with clusters 2, 5, 6, and 7
CALCOCO1	-0.6517024	0.043	0.292	0.00077967	6	
LGALS2	-0.6632152	0.193	0.637	1.22E-07	6	Shared with clusters 2, 6, and 7
IFRD1	-0.6773078	0.107	0.324	0.00050045	6	Shared with clusters 2 and 6
CALM3	-0.6779221	0.236	0.797	1.31E-11	6	Shared with clusters 0, 2, 3, 6, and 7
S100A9	-0.710881	0.071	0.552	2.25E-23	6	Shared with clusters 0, 1, 2, 3, 5, 6, and 7
DYNLL1	-0.7440505	0.336	0.846	0.00010152	6	Shared with clusters 5 and 6
TXNRD1	-0.7628115	0.021	0.496	6.24E-26	6	Shared with clusters 0, 1, 3, 4, 5, 6, and 7
COX7B	-0.773324	0.157	0.808	0.00033067	6	Shared with clusters 0, 1, 2, 5, and 6
DBI	-0.7771323	0.229	0.857	4.91E-16	6	Shared with clusters 0, 2, 5, and 6
ZNF148	-0.7785539	0.021	0.342	2.36E-06	6	Shared with clusters 5 and 6
IRF1	-0.8031766	0.057	0.35	0.00148096	6	Shared with clusters 2 and 6
PTPN6	-0.8202176	0.071	0.62	1.10E-05	6	Shared with clusters 0, 3, 5, 6, and 7
7SK.2	-0.8563005	0.057	0.295	0.00145316	6	
ATP5G3	-0.8719024	0.207	0.872	7.25E-11	6	Shared with clusters 0, 5, and 6
CD84	-0.8855388	0.093	0.549	1.26E-05	6	Shared with clusters 0, 1, 3, 5, 6, and 7
CXCL2	-0.8987188	0.871	0.891	0.00464651	6	
DENND3	-0.9100562	0.064	0.387	0.0025611	6	Shared with clusters 2, 5, and 6
FAM20C	-0.9204984	0.064	0.569	3.08E-06	6	Shared with clusters 0, 1, 2, 3, 4, 5, 6, and 7
FABP5	-0.9519656	0.721	0.939	4.25E-53	6	Shared with clusters 0, 1, 2, 3, 4, 5, 6, and 7
YWHAB	-0.9535978	0.071	0.632	0.00765175	6	Shared with clusters 5 and 6
FAM118A	-0.9675149	0.021	0.353	1.89E-05	6	Shared with clusters 5 and 6
ZBTB25	-0.9705511	0.086	0.381	2.39E-07	6	Shared with clusters 2, 5, and 6
CALM2	-0.9811688	0.564	0.914	9.31E-05	6	Shared with clusters 1, 2, and 6
PPP1R10	-1.0107231	0.036	0.383	7.25E-07	6	Shared with clusters 2 and 6
RAC2	-1.0108986	0.129	0.886	6.95E-07	6	
PSMA7	-1.0160583	0.129	0.811	1.73E-11	6	Shared with clusters 0, 3, 5, and 6
IL1RN	-1.0198315	0.221	0.85	1.16E-26	6	Shared with clusters 0, 1, 3, 4, 5 6, and 7
FERMT3	-1.0266058	0.079	0.687	2.05E-05	6	Shared with clusters 0, 1, 3, 5, 6, and 7
PLEKHO1	-1.0321341	0.071	0.663	0.0030577	6	Shared with clusters 1, 2, 5, 6, and 7
HMG20B	-1.048947	0.079	0.707	4.32E-14	6	Shared with clusters 0, 1, 3, 4, 5, and 7
PPP1R14B	-1.0609863	0.1	0.814	0.00044599	6	Shared with clusters 5, 6, and 7
TXN	-1.0739733	0.529	0.951	6.75E-14	6	Shared with clusters 0, 2, 3, 5, and 6
PLEK	-1.0772903	0.193	0.789	6.71E-07	6	Shared with clusters 0, 5, 6, and 7
LINC00936	-1.1489996	0.2	0.458	5.39E-09	6	
PGAM1	-1.1776939	0.271	0.932	0.0004867	6	
TET2	-1.1961565	0.043	0.464	1.40E-05	6	Shared with clusters 2, 5, 6, and 7
CFL1	-1.2114426	0.371	0.913	4.88E-18	6	Shared with clusters 0, 1, 2, 3, 5, 6, and 7
TGFB1	-1.2376623	0.1	0.8	0.00010259	6	Shared with clusters 0, 1, 2, 3, 5, and 6
CAPG	-1.2467357	0.179	0.811	1.11E-20	6	Shared with clusters 0, 1, 2, 3, 5, 6, and 7
ABHD5	-1.2579677	0.157	0.571	4.55E-05	6	Shared with clusters 2, 5, and 6
ARPC5	-1.267708	0.336	0.875	3.15E-06	6	Shared with clusters 2, 5, and 6
MYL12A	-1.2738329	0.236	0.942	2.93E-08	6	Shared with clusters 0, 5, and 6

SERF2	-1.2747806	1	1	1.97E-13	6	Shared with clusters 1, 2, and 6
PRDX1	-1.2859424	0.421	0.933	1.79E-12	6	Shared with clusters 0, 1, 2, 3, 5, 6, and 7
RPS15A	-1.3099684	1	0.998	0.00362972	6	Shared with clusters 0 and 6
GM2A	-1.3397508	0.143	0.791	9.15E-05	6	Shared with clusters 0, 2, 3, 5, 6, and 7
GSTO1	-1.3462969	0.686	0.982	4.51E-08	6	Shared with clusters 0, 3, 5, 6, and 7
RGCC	-1.357529	0.757	0.933	1.81E-22	6	Shared with clusters 0, 1, 5, 6, and 7
MIDN	-1.376132	0.05	0.596	0.00040018	6	Shared with clusters 0 and 6
NFKBIZ	-1.3852755	0.264	0.523	7.57E-10	6	
GUSB	-1.4027554	0.064	0.744	0.00046204	6	Shared with clusters 0, 1, 2, 3, 5, 6, and 7
CHI3L1	-1.4675676	0.071	0.668	6.71E-99	6	Shared with clusters 0, 1, 2, 3, 4, 5, 6, and 7
DUSP2	-1.4874342	0.279	0.624	1.39E-08	6	Shared with clusters 4, 5, and 6
COTL1	-1.4908564	0.271	0.957	0.00080135	6	Shared with clusters 0, 2, and 6
CTSL	-1.5340119	0.314	0.784	7.67E-15	6	Shared with clusters 0, 1, 2, 3, 5, 6, and 7
FAM63B	-1.5375395	0.15	0.692	0.00422226	6	Shared with clusters 2 and 6
FBP1	-1.6339242	0.271	0.965	4.65E-51	6	Shared with clusters 0, 1, 2, 3, 5, 6, and 7
KLF2	-1.6697438	0.036	0.612	6.40E-06	6	Shared with clusters 5 and 6
ZFP36	-1.6813268	0.614	0.95	3.69E-09	6	
MFSD12	-1.694735	0.107	0.784	5.85E-35	6	Shared with clusters 0, 1, 2, 3,4, 5, 6, and 7
TALDO1	-1.7084942	0.207	0.956	8.40E-09	6	Shared with clusters 2, 5, 6, and 7
TUBA1B	-1.7454194	0.207	0.945	6.30E-16	6	Shared with clusters 0, 1, 3, 5, 6, and 7
PPP1R15A	-1.7624079	0.729	0.926	1.86E-10	6	
STX7	-1.7726395	0.15	0.761	0.00047005	6	Shared with clusters 2, 5, and 6
ARPC1B	-1.8093191	0.243	0.91	4.20E-35	6	Shared with clusters 0, 1, 2, 3, 5, 6, and 7
SH3BGRL3	-1.8239031	0.943	0.998	4.38E-09	6	Shared with clusters 6 and 7
ANXA2	-1.884205	0.457	0.977	2.35E-15	6	Shared with clusters 0, 1, 2, 3, 5, 6, and 7
BCAP31	-1.8936293	0.179	0.942	0.00520459	6	Shared with clusters 2, 3, 6, and 7
GSN	-1.8984907	0.121	0.939	5.51E-23	6	Shared with clusters 0, 1, 2, 3, 5, 6, and 7
LAMP1	-1.9169358	0.25	0.869	0.00042536	6	Shared with clusters 0, 2, 3, 6, and 7
ANXA5	-2.0001755	0.2	0.969	0.00065126	6	Shared with clusters 1, 5, 6, and 7
MT1X	-2.0657248	0.707	0.889	2.81E-12	6	Shared with clusters 0, 1, 3, 4, 5, 6,and 7
ABCC3	-2.1598456	0.15	0.748	0.00110768	6	Shared with clusters 2, 6, and 8
RPS19	-2.1819761	1	0.999	0.00106841	6	Shared with clusters 0 and 6
CD52	-2.2419094	0.95	0.995	4.88E-14	6	Shared with clusters 2, 6, and 7
TNFRSF14	-2.2576869	0.214	0.889	0.00188767	6	
LIPA	-2.2637417	0.536	0.936	1.10E-48	6	Shared with clusters 0, 1, 2, 3, 4, 5, 6, and 7
MYL6	-2.2863987	0.95	0.997	4.57E-10	6	Shared with clusters 5 and 6
CD81	-2.3274131	0.114	0.955	0.00028242	6	Shared with clusters 0, 1, 2, 5, and 6
S100A4	-2.5481572	0.593	0.988	4.32E-07	6	Shared with clusters 0, 2, and 6
CYP27A1	-2.6196385	0.45	0.98	1.60E-05	6	Shared with clusters 2, 5, and 6
CHD2	-2.6798175	0.164	0.943	0.00030961	6	Shared with clusters 2, 5, and 6
EEF1B2	-2.7867146	0.143	0.983	1.95E-06	6	Shared with clusters 0, 5, and 6
FLNA	-2.8869428	0.271	0.977	0.00014737	6	Shared with clusters 1, 3, 5, and 6
MT2A	-2.9579326	0.829	0.961	2.30E-12	6	Shared with clusters 0, 2, 1, 3, 4, 5, 6, and 7
HCST	-3.0045981	0.786	0.993	1.71E-06	6	Shared with clusters 2, 6, and 7
RPSA	-3.0162689	0.686	0.99	0.0021411	6	Shared with clusters 0, 1, 5, and 6
ARRDC3	-3.1339768	0.807	0.991	1.35E-07	6	Shared with clusters 2, 5, and 6
CSTB	-3.1846613	1	1	2.58E-22	6	Shared with clusters 0, 1, 5, 6, and 7
YBX1	-3.254563	0.857	0.996	0.00079944	6	
CTSZ	-3.3981748	0.257	0.975	8.40E-07	6	Shared with clusters 0, 1, 2, 3, 6, and 7
BTG2	-3.3987364	0.093	0.876	1.13E-26	6	Shared with clusters 2, 5, and 6
S100A11	-3.5325377	0.993	0.999	7.43E-17	6	Shared with clusters 1, 5, 6, and 7
MCL1	-3.5578273	0.436	0.982	6.02E-27	6	Shared with clusters 5 and 6
ACTG1	-3.6216216	0.593	0.993	5.58E-08	6	Shared with clusters 0 and 6
APOE	-3.6436469	0.85	0.988	0.00146185	6	Shared with clusters 0, 2, 3, 5, 6, and 7
CCNL1	-3.6790102	0.486	0.986	7.17E-05	6	Shared with clusters 2, 5, and 6
CXCL3	-3.7278519	0.914	0.951	0.00028993	6	Shared with clusters 2, 4, and 6
PGK 1.00	-3.820516	0.586	0.988	0.00637758	6	
ZNF292	-4.0878905	0.164	0.969	1.81E-05	6	Shared with clusters 0, 2, 5, and 6
DDX5	-4.3001755	0.821	0.994	2.03E-11	6	
NFKBIA	-4.5168129	0.943	0.962	1.23E-21	6	
RPLP1	-4.7013163	1	1	5.40E-07	6	Shared with clusters 0, 2, and 6
RPS3	-4.900158	0.986	0.999	1.07E-08	6	Shared with clusters 0, 5, 6, and 7
FOSB	-4.9461039	0.764	0.903	2.70E-06	6	Shared with clusters 2, 5, and 6
S100A6	-5.1097578	1	1	4.31E-05	6	Shared with clusters 1, 2, 5, and 6

RPS27A	-5.1514391	1	0.998	0.00014363	6	Shared with clusters 0, 6, and 7
RPS12	-5.1905405	0.971	0.999	2.22E-20	6	Shared with clusters 0, 4, 5, and 6
RPS23	-5.3245525	1	0.999	1.98E-13	6	Shared with clusters 0, 2, and 6
RPS24	-5.3576518	0.971	0.999	5.77E-13	6	Shared with clusters 0, 1, 2, and 6
DUSP1	-5.4370481	0.993	0.994	2.82E-27	6	
IER2	-5.4788873	0.607	0.982	8.95E-12	6	Shared with clusters 2, 5, and 6
RPS18	-5.4918919	0.993	1	5.91E-06	6	Shared with clusters 0, 1, 4, and 6
EGR1	-5.6304668	0.521	0.977	9.43E-27	6	Shared with clusters 1, 2, 5, and 6
LGALS3	-5.715251	0.957	0.999	9.10E-32	6	Shared with clusters 0, 1, 2, 3, 4, 5, 6, and 7
JUNB	-5.7170937	0.871	0.998	0.00215543	6	
IL8	-6.1115655	0.993	0.993	9.39E-16	6	Shared with clusters 0, 2, 4, 5, and 6
ACTB	-6.1997718	0.843	0.999	2.19E-17	6	Shared with clusters 0, 1, 3, 5, 6, and 7
RPL7A	-6.4578449	0.95	1	2.69E-11	6	Shared with clusters 0, 1, 4, 5, 6, and 7
GNAS	-6.5194981	0.393	0.999	0.00070297	6	
RPS3A	-6.8420147	1	1	0.00329238	6	Shared with clusters 0, 1, and 6
CD63	-7.2337311	0.993	0.999	5.04E-09	6	Shared with clusters 0, 1, 2, 3, 4, 5, 6, and 7
RPL30	-7.2551071	0.986	0.999	3.54E-17	6	Shared with clusters 0, 6, and 7
RPL13	-7.829133	0.993	0.999	0.00040102	6	Shared with clusters 0, 1, 4, 5, and 6
KLF6	-7.8361004	0.929	0.999	1.39E-16	6	
EREG	-9.6790804	0.707	0.97	1.88E-14	6	Shared with clusters 1, 2, 5, 6, and 7
JUN	-11.400421	0.971	0.997	8.68E-25	6	
MMP9	-11.960232	0.7	0.998	2.56E-08	6	Shared with clusters 0, 1, 2, 3, 4, 5, 6, and 7
TPT1	-12.807599	0.993	0.999	1.16E-20	6	Shared with clusters 1, 6, and 7
BTG1	-13.260969	1	1	6.24E-09	6	Shared with clusters 0, 2, 6, and 7
VCAN	-13.330713	0.457	0.978	0.00377958	6	Shared with clusters 0, 2, 5, 6, and 7
TIMP1	-13.774447	0.814	0.998	0.00154878	6	Shared with clusters 2, 3, 5, 6, and 7
FOS	-15.500298	0.993	0.999	1.87E-18	6	Shared with clusters 1, 5, and 6
H3F3B	-15.878607	1	0.999	2.41E-19	6	Shared with clusters 2, 5, and 6
MTRNR2L12	-17.976922	0.207	0.982	2.25E-18	6	Shared with clusters 2, 5, and 6
SAT1	-19.155002	0.986	1	3.24E-10	6	
ZFP36L1	-22.072517	0.964	1	1.96E-05	6	Shared with clusters 0, 2, 5, 6, and 7
MT-ND3	-31.016427	0.7	0.996	7.54E-07	6	Shared with clusters 5 and 6
SPP1	-39.383819	0.993	1	1.10E-52	6	Shared with clusters 0, 1, 2, 3, 4, 5, 6, and 7
MT-CO3	-43.585907	0.893	1	7.86E-06	6	Shared with clusters 0, 5, and 6
MT-ATP6	-50.31232	0.85	1	6.44E-06	6	
MT-CO2	-65.218077	0.886	0.999	4.43E-07	6	Shared with clusters 0, 2, and 6
NEAT1	-188.44177	1	1	3.46E-12	6	Shared with clusters 2 and 6
RPL13A	8.60785221	1	1	9.12E-07	7	Shared with clusters 6 and 7
MIF	1.83107717	1	0.998	0.00171931	7	
RP11-1143G	0.57958271	0.977	0.813	2.49E-19	7	
CD69	0.35118979	0.535	0.516	0.00261102	7	
TIMP3	-0.0281924	0	0.01	9.24E-46	7	Shared with clusters 5 and 7
PLCXD1	-0.0281924	0	0.015	8.49E-17	7	
PLTP	-0.0281924	0	0.015	1.31E-69	7	
TMEM106A	-0.0281924	0	0.018	3.38E-08	7	
TNFSF9	-0.0281924	0	0.018	2.25E-11	7	
TTYH3	-0.0281924	0	0.015	4.09E-45	7	
NCEH1	-0.0298507	0	0.015	5.94E-98	7	Shared with clusters 3, 5, and 7
SEMA3C	-0.0298507	0	0.012	2.37E-60	7	Shared with clusters 5 and 7
HSPA6	-0.0298507	0	0.018	0.00019819	7	Shared with clusters 5 and 7
MYO15B	-0.0298507	0	0.018	2.02E-10	7	Shared with clusters 6 and 7
PPM1F	-0.0298507	0	0.017	8.29E-10	7	
LACC1	-0.0315091	0	0.017	0.00127535	7	Shared with clusters 2 and 7
P2RX7	-0.0315091	0	0.017	9.13E-56	7	Shared with clusters 2, 5, 6, and 7
NR1H3	-0.0315091	0	0.017	4.96E-62	7	Shared with clusters 5 and 7
NRIP3	-0.0315091	0	0.017	3.61E-34	7	Shared with clusters 5 and 7
ARRB1	-0.0315091	0	0.017	2.73E-08	7	
ATG7	-0.0315091	0	0.017	5.93E-33	7	
IRF5	-0.0315091	0	0.02	1.39E-19	7	
MSC	-0.0315091	0	0.017	2.74E-12	7	
OLR1	-0.0315091	0	0.023	4.35E-12	7	
POLD3	-0.0315091	0	0.02	6.12E-08	7	
ST20	-0.0331675	0	0.02	2.57E-23	7	Shared with clusters 0, 5, and 7
FAM129B	-0.0331675	0	0.018	6.78E-106	7	Shared with clusters 5 and 7

SNX30	-0.0331675	0	0.018	7.74E-22	7	
DFNA5	-0.0348259	0	0.027	9.05E-13	7	Shared with clusters 0, 2, and 7
PHLDA3	-0.0348259	0	0.017	9.66E-74	7	Shared with clusters 0, 3, 5, 6, and 7
AHRR	-0.0348259	0	0.02	6.29E-24	7	Shared with clusters 2, 5, and 7
DHRS11	-0.0348259	0	0.022	1.54E-18	7	Shared with clusters 5 and 7
GPR107	-0.0348259	0	0.018	2.04E-10	7	
LILRB1	-0.0348259	0	0.017	4.43E-11	7	
FN1	-0.0364842	0	0.02	9.68E-16	7	Shared with clusters 1, 3, and 7
FLNB	-0.0364842	0	0.022	8.29E-50	7	
ICAM1	-0.0364842	0	0.017	7.76E-15	7	
P2RY11	-0.0364842	0	0.018	6.69E-05	7	
MS4A4A	-0.0381426	0	0.025	1.44E-39	7	Shared with clusters 0, 2, 5, and 7
MYOF	-0.0381426	0	0.02	5.90E-84	7	Shared with clusters 0, 3, 5, and 7
PLEKHO2	-0.0381426	0	0.022	1.48E-09	7	Shared with clusters 1, 2, 5, 6, and 7
TLR6	-0.0381426	0	0.022	6.31E-21	7	Shared with clusters 5 and 7
CLEC11A	-0.0381426	0	0.02	8.64E-17	7	
TET3	-0.0381426	0	0.025	0.00167547	7	
TRIO	-0.0381426	0	0.022	9.51E-15	7	
VNN 2.00	-0.039801	0	0.02	2.06E-16	7	Shared with clusters 5 and 7
HSD3B7	-0.039801	0	0.02	2.43E-82	7	Shared with clusters 5 and 7
MOB1B	-0.039801	0	0.027	1.59E-14	7	
RP11-268J1	-0.039801	0	0.025	9.39E-08	7	
TTLL4	-0.039801	0	0.02	8.70E-24	7	
SLC16A6	-0.0413822	0.023	0.045	0.0003335	7	Shared with clusters 0 and 7
NLRP3	-0.0414594	0	0.025	0.00090269	7	
VAC14	-0.0414594	0	0.025	2.39E-19	7	
CHML	-0.0431177	0	0.025	2.24E-05	7	Shared with clusters 0, 2, 5, and 7
F11R	-0.0431177	0	0.025	1.61E-16	7	Shared with clusters 5 and 7
IL18BP	-0.0431177	0	0.022	5.46E-75	7	
RPS6KA2	-0.0431177	0	0.023	2.88E-24	7	
MNDA	-0.0447761	0	0.025	1.54E-60	7	Shared with clusters 0, 2, 6, and 7
LGMN	-0.0447761	0	0.025	0.00094199	7	
MICAL1	-0.0447761	0	0.028	6.20E-18	7	
PLA2G15	-0.0447761	0	0.023	1.00E-08	7	
SLC23A2	-0.0447761	0	0.025	1.78E-55	7	
ZFHX3	-0.0447761	0	0.023	1.26E-15	7	
GNA12	-0.0464345	0	0.03	2.29E-15	7	Shared with clusters 0 and 7
C1orf54	-0.0464345	0	0.023	1.43E-43	7	Shared with clusters 2 and 7
NR4A3	-0.0464345	0	0.017	2.86E-09	7	Shared with clusters 2, 5, 6, and 7
FADS1	-0.0464345	0	0.025	3.91E-61	7	
TNRC18	-0.0464345	0	0.028	1.34E-09	7	
KCNN4	-0.0480929	0	0.028	4.23E-54	7	Shared with clusters 5 and 7
PDE4A	-0.0480929	0	0.025	6.03E-17	7	
RAI14	-0.0480929	0	0.023	1.18E-46	7	
SEPN1	-0.0480929	0	0.025	3.81E-12	7	
CLCN7	-0.0497512	0	0.03	6.58E-06	7	Shared with clusters 0, 2, and 7
ZMYM6	-0.0497512	0	0.028	0.0018842	7	Shared with clusters 2 and 7
FCHO2	-0.0497512	0	0.025	0.00013807	7	
C3	-0.0514096	0	0.028	1.96E-35	7	Shared with clusters 0, 3, 4, 5, 6, and 7
FNDC3B	-0.0514096	0	0.03	3.74E-22	7	Shared with clusters 2 and 7
ICAM5	-0.0514096	0	0.03	3.18E-52	7	Shared with clusters 5 and 7
NRP2	-0.0514096	0	0.02	2.15E-27	7	Shared with clusters 5 and 7
ATP1B1	-0.0514096	0	0.03	3.04E-06	7	
DNAJC13	-0.0514096	0	0.028	0.00020505	7	
DOCK5	-0.0514096	0	0.023	9.58E-47	7	
TRAK1	-0.053068	0	0.032	7.12E-06	7	Shared with clusters 6 and 7
C1QA	-0.0547264	0	0.032	1.71E-111	7	Shared with clusters 0, 2, 3, 4, and 7
THBS1	-0.0563847	0	0.033	3.98E-68	7	Shared with clusters 0, 2, 5, and 7
VMO1	-0.0563847	0	0.02	0.00017943	7	Shared with clusters 2, 4, and 7
MMP7	-0.0563847	0	0.028	1.54E-109	7	Shared with clusters 2, 5, 6, and 7
BMP2K	-0.0563847	0	0.03	4.21E-10	7	
SLC1A5	-0.0563847	0	0.033	0.00154763	7	
NCSTN	-0.0580431	0	0.032	3.68E-30	7	
H1F0	-0.0597015	0	0.033	6.80E-24	7	Shared with clusters 2, 5, and 7

CLMN	-0.0597015	0	0.035	5.99E-31	7	Shared with clusters 5 and 7
NFXL1	-0.0613599	0	0.035	1.06E-07	7	Shared with clusters 0, 2, 5, 6, and 7
SYNGR1	-0.0613599	0	0.028	6.78E-19	7	Shared with clusters 0, 2, and 7
ATP6V0A1	-0.0613599	0	0.032	6.29E-38	7	
ADRBK2	-0.0630182	0	0.04	3.62E-37	7	
TYK2	-0.0630182	0	0.035	0.00024417	7	
ATP13A1	-0.0646766	0	0.036	0.00386203	7	
SEMA6B	-0.0679934	0	0.02	1.64E-14	7	
DOPEY2	-0.0696517	0	0.035	4.85E-11	7	Shared with clusters 5 and 7
LXN	-0.0696517	0	0.036	3.91E-07	7	Shared with clusters 5 and 7
EMILIN2	-0.0696517	0	0.041	8.58E-111	7	
KCTD12	-0.0696517	0	0.036	6.12E-25	7	
MT1E	-0.0712715	0.023	0.041	5.22E-15	7	Shared with clusters 0, 1, 2, 3, 4, 6,and 7
ASPH	-0.0713101	0	0.038	7.13E-21	7	Shared with clusters 5 and 7
PEA15	-0.0745883	0.023	0.053	0.00224444	7	Shared with clusters 2, 5, 6, and 7
ZNF516	-0.0746269	0	0.04	1.32E-06	7	Shared with clusters 2, 5, and 7
HAVCR2	-0.0746269	0	0.035	4.58E-62	7	Shared with clusters 3 and 7
GPAA1	-0.0762852	0	0.038	1.72E-37	7	Shared with clusters 2 and 7
PINK1	-0.0762852	0	0.041	8.38E-16	7	Shared with clusters 2 and 7
CTNNA1	-0.0762852	0	0.038	2.25E-47	7	Shared with clusters 5 and 7
TEX30	-0.0762852	0	0.046	0.00294518	7	
TPCN1	-0.0779436	0	0.04	3.78E-11	7	
ABHD2	-0.0812604	0	0.04	1.08E-99	7	Shared with clusters 0, 3, 5, and 7
LIMD1	-0.0812604	0	0.043	1.84E-18	7	Shared with clusters 2 and 7
SLC25A29	-0.0812604	0	0.046	2.27E-10	7	
SLC16A10	-0.0829187	0	0.045	1.06E-11	7	Shared with clusters 0, 2, and 7
PGPEP1	-0.0829187	0	0.041	2.07E-20	7	Shared with clusters 5 and 7
DAB2	-0.0829187	0	0.046	1.03E-45	7	
SOX4	-0.0845771	0	0.033	5.79E-05	7	
H6PD	-0.0912106	0	0.046	0.00046828	7	
PACSIN2	-0.092869	0	0.05	2.03E-06	7	
MARCH2	-0.0945274	0	0.053	1.74E-14	7	Shared with clusters 0, 2, 3, 5, and 7
MITF	-0.0945274	0	0.078	2.15E-133	7	
GLA	-0.0978441	0	0.046	2.88E-75	7	Shared with clusters 0, 2, 5, and 7
GAN	-0.1028192	0	0.061	1.75E-06	7	
GSAP	-0.1028192	0	0.053	4.41E-05	7	
MSMO1	-0.106136	0	0.056	3.55E-17	7	Shared with clusters 0, 5, 6, and 7
IFRD2	-0.106136	0	0.068	0.00201742	7	
IFT57	-0.106136	0	0.07	0.00892635	7	
SH3BP2	-0.1077944	0	0.053	1.31E-27	7	Shared with clusters 2 and 7
POR	-0.1077944	0	0.058	0.00476688	7	
TNFSF14	-0.1094527	0	0.05	2.57E-45	7	Shared with clusters 5 and 7
RP5-827C21	-0.1094527	0	0.061	0.00053602	7	
LPAR2	-0.1111111	0	0.063	3.11E-05	7	Shared with clusters 2, 6, and 7
MT1H	-0.1127695	0	0.051	7.56E-25	7	Shared with clusters 0, 1, 3, 4, 5, and 7
MGLL	-0.1127695	0	0.048	4.10E-146	7	Shared with clusters 2, 3, 4, 5, 6, and 7
MTHFS	-0.1127695	0	0.056	0.00190053	7	Shared with clusters 5, 6, and 7
ALCAM	-0.1143893	0.023	0.081	2.56E-10	7	Shared with clusters 0, 1, 2, 3, 4 ,5, 6, and 7
ADCY7	-0.1144279	0	0.06	0.00218475	7	
IQSEC1	-0.1144279	0	0.06	3.68E-11	7	
MRI1	-0.1160862	0	0.07	1.07E-05	7	Shared with clusters 0, 4, and 7
CA5B	-0.1160862	0	0.07	0.00645789	7	
CCR1	-0.1160862	0	0.1	5.21E-100	7	
TMEM138	-0.1160862	0	0.06	1.28E-05	7	
ZNF699	-0.1160862	0	0.068	0.00226202	7	
AK9	-0.1177446	0	0.066	0.00659641	7	
RP11-182L2	-0.119403	0	0.073	0.00029231	7	
G6PD	-0.1210614	0	0.058	2.90E-79	7	Shared with clusters 0, 1, 2, 3, 4, 5, 6, and 7
LGALS9	-0.1210614	0	0.061	4.47E-46	7	Shared with clusters 2 and 7
PEPD	-0.1210614	0	0.063	7.22E-42	7	
PTAFR	-0.1227197	0	0.119	2.67E-66	7	Shared with clusters 0 and 7
EMC9	-0.1243781	0	0.081	2.02E-05	7	
NOB1	-0.1260365	0	0.075	0.0008782	7	
ATP6V1D	-0.1276949	0	0.061	5.35E-59	7	Shared with clusters 3, 5, 6, and 7

RPN1	-0.1276949	0	0.068	1.83E-41	7	
EIF4G1	-0.1293532	0	0.07	3.04E-14	7	
KTI12	-0.1293532	0	0.076	0.00125306	7	
FAM89A	-0.1310116	0	0.081	1.40E-05	7	
NF1	-0.13267	0	0.068	0.00401041	7	Shared with clusters 2, 5, and 7
ICT1	-0.13267	0	0.083	0.00028413	7	
RIPK2	-0.13267	0	0.091	0.00290147	7	
LINC00324	-0.1343284	0	0.076	0.00652761	7	
FNIP2	-0.1376451	0	0.08	1.84E-20	7	Shared with clusters 2, 5, and 7
RP13-582O9	-0.1376451	0	0.076	0.00029807	7	
MT1G	-0.1392263	0.023	0.088	1.87E-11	7	Shared with clusters 0, 1, 3, 4, 5, 6,and 7
EPHA1-AS1	-0.1393035	0	0.085	0.00201245	7	
PLEC	-0.1393035	0	0.068	1.89E-22	7	
GCLC	-0.1409619	0	0.073	3.28E-33	7	Shared with clusters 1, 2, 3, 4, 5, 6, and 7
PDE4B	-0.1409619	0	0.076	0.00522349	7	
CTB-61M7.2	-0.1426202	0	0.133	5.95E-06	7	Shared with clusters 0, 2, 5, and 7
ELK3	-0.145937	0	0.093	9.78E-06	7	
GBP4	-0.145937	0	0.073	8.10E-08	7	
ZNF639	-0.1475954	0	0.095	5.54E-05	7	
TET2	-0.1525705	0	0.075	2.26E-16	7	Shared with clusters 2, 5, 6, and 7
IFI6	-0.1590497	0.07	0.156	5.99E-07	7	Shared with clusters 2. 3, 5, 6, and 7
FAM101B	-0.159204	0	0.08	4.34E-08	7	Shared with clusters 6 and 7
ANO6	-0.1641791	0	0.078	8.09E-05	7	
PRKAG2-AS	-0.1641791	0	0.086	2.01E-14	7	
FAM115C	-0.1658375	0	0.09	0.00266759	7	Shared with clusters 2 and 7
GLCCI1	-0.1658375	0	0.088	0.00010642	7	
PWWP2A	-0.1658375	0	0.1	0.00062408	7	
RNF125	-0.1691542	0	0.093	0.00572729	7	
FABP4	-0.1702341	0.302	0.126	2.08E-26	7	Shared with clusters 0, 1, 2, 3, 4, 5, 6, and 7
MTMR9	-0.1708126	0	0.093	0.00034613	7	Shared with clusters 5 and 7
ZDHHC2	-0.172471	0	0.096	0.00951371	7	Shared with clusters 0, 5, and 7
RELB	-0.172471	0	0.088	1.98E-06	7	
YAF2	-0.172471	0	0.096	2.45E-05	7	
CYSTM1	-0.1740522	0.023	0.148	1.48E-06	7	Shared with clusters 0, 2, 3, 5, 6, and 7
STARD10	-0.1774461	0	0.093	0.00432535	7	Shared with clusters 2, 5, and 7
DALRD3	-0.1774461	0	0.103	3.64E-05	7	
EXOC3	-0.1791045	0	0.091	0.0001297	7	Shared with clusters 0, 2, 5, and 7
ENG	-0.1791045	0	0.139	5.25E-70	7	
RHOQ	-0.1807629	0	0.086	0.00495588	7	
SIRPA	-0.1824212	0	0.163	5.29E-75	7	
CEP135	-0.1840796	0	0.096	0.0026224	7	
NUP54	-0.1840796	0	0.101	0.00119069	7	
NIFK-AS1	-0.185738	0	0.104	1.13E-05	7	
PPP1R15B	-0.1890547	0	0.119	0.00027702	7	
PRR5	-0.1906745	0.023	0.111	0.00973753	7	
NR1D2	-0.1956882	0	0.109	0.0003518	7	Shared with clusters 5 and 7
TRAF1	-0.1956882	0	0.119	9.20E-06	7	
PRKAA1	-0.1973466	0	0.113	6.95E-07	7	
NPRL2	-0.2039801	0	0.109	9.92E-09	7	
FMR1	-0.2056385	0	0.111	0.00259509	7	
PRPF3	-0.2072968	0	0.108	0.00085937	7	
CD59	-0.2089552	0	0.101	0.00013069	7	Shared with clusters 0, 1, 2, 3, 5, 6, and 7
OTUD1	-0.2089552	0	0.113	0.00768268	7	
RP11-83A24	-0.2089552	0	0.104	0.00088063	7	
GPRIN3	-0.2106136	0	0.1	7.91E-23	7	
NOC2L	-0.2106136	0	0.123	0.00086345	7	
MED19	-0.212272	0	0.111	2.50E-05	7	
MED17	-0.2139303	0	0.111	0.00415208	7	
EHMT1	-0.2189055	0	0.118	0.00186042	7	
MARCO	-0.225539	0	0.199	1.53E-49	7	Shared with clusters 0,1, 2, and 7
DGKD	-0.225539	0	0.111	0.00397835	7	Shared with clusters 2 and 7
CLEC7A	-0.225539	0	0.176	0.00173495	7	Shared with clusters 5 and 7
RGP1	-0.225539	0	0.124	2.09E-07	7	
TTC9C	-0.2271973	0	0.123	2.11E-09	7	

ATP1B3	-0.2288557	0	0.109	5.88E-23	7	Shared with clusters 3, 5, and 7
MAP4K4	-0.2305141	0	0.136	5.54E-05	7	
CXCL5	-0.2318254	0.209	0.345	3.58E-05	7	Shared with clusters 0, 2, 3, 5, and 7
MGAT4B	-0.2338308	0	0.111	1.04E-07	7	Shared with clusters 2, 5, and 7
ATP6V1A	-0.237109	0.023	0.245	9.89E-06	7	Shared with clusters 5 and 7
CEP170	-0.2454395	0	0.159	5.45E-13	7	Shared with clusters 2 and 7
ATP13A3	-0.2470978	0	0.186	0.00068302	7	Shared with clusters 2 and 7
C4orf48	-0.2504146	0	0.124	9.43E-08	7	Shared with clusters 2 and 7
SLC7A7	-0.252073	0	0.235	8.27E-55	7	Shared with clusters 0, 2, and 7
TAF3	-0.2537313	0	0.139	0.00018261	7	
CTCF	-0.2570481	0	0.144	0.00026279	7	
MSI2	-0.2570481	0	0.133	1.80E-07	7	
SENP7	-0.2587065	0	0.144	1.42E-07	7	
PPT1	-0.2602877	0.023	0.231	5.90E-07	7	Shared with clusters 0, 1, 2, 3, 6, and 7
TIMM9	-0.2603648	0	0.136	1.88E-11	7	
LUCAT1	-0.26534	0	0.214	2.60E-30	7	
BCL2A1	-0.2669212	0.023	0.171	2.15E-11	7	Shared with clusters 0, 2, 3, 5, 6, and 7
RASAL3	-0.2669212	0.023	0.177	0.00762619	7	
PLEKHM2	-0.2686567	0	0.212	1.27E-14	7	
SLC9A3R1	-0.2703151	0	0.146	2.13E-10	7	
MATK	-0.278607	0	0.123	3.51E-49	7	Shared with clusters 0, 3, 4, 5, 6, and 7
TP53I13	-0.2802653	0	0.153	0.0049625	7	
NOTCH2	-0.2935323	0	0.214	3.26E-05	7	
FAH	-0.3000887	0.023	0.282	6.05E-08	7	Shared with clusters 5, 6, and 7
C11orf83	-0.3001658	0	0.151	1.75E-11	7	
HSD17B4	-0.3018242	0	0.201	4.17E-20	7	
KAT6B	-0.3018242	0	0.159	4.52E-05	7	
ZBTB20	-0.3084577	0	0.167	3.56E-08	7	Shared with clusters 5, 6, and 7
RELA	-0.3084577	0	0.156	2.14E-07	7	
KIAA1430	-0.3150912	0	0.161	8.74E-09	7	
SCPEP1	-0.3183694	0.023	0.26	3.15E-05	7	Shared with clusters 0, 1, 2, and 7
AHR	-0.318408	0	0.221	3.92E-40	7	Shared with clusters 0 and 7
RNF166	-0.318408	0	0.176	2.13E-10	7	
SLC4A7	-0.3217247	0	0.161	0.00099551	7	
TMEM107	-0.3266998	0	0.166	3.76E-07	7	
ZNF800	-0.3266998	0	0.169	8.30E-05	7	
PMAIP1	-0.3299395	0.023	0.124	0.00535222	7	
PTGER2	-0.3315978	0.023	0.179	0.00623737	7	
C15orf48	-0.3402754	0.767	0.977	6.32E-05	7	Shared with clusters 5 and 7
SDC2	-0.364881	0.814	0.982	1.92E-05	7	Shared with clusters 0 and 7
HNRNPL	-0.3698176	0	0.181	8.63E-07	7	
STAB1	-0.3731343	0	0.332	4.78E-118	7	Shared with clusters 3 and 7
FAM210A	-0.3747927	0	0.182	9.49E-06	7	
EMB	-0.3780323	0.023	0.207	0.00027795	7	
PIK3AP1	-0.3797678	0	0.3	2.92E-57	7	Shared with clusters 5 and 7
CDK12	-0.3814262	0	0.181	0.00358213	7	Shared with clusters 2, 6, and 7
LPP	-0.3880597	0	0.284	1.51E-21	7	Shared with clusters 2 and 7
SLC43A3	-0.3897181	0	0.322	7.04E-35	7	Shared with clusters 2 and 7
NCKAP1L	-0.3913765	0	0.299	8.56E-43	7	
MARCH9	-0.3963516	0	0.196	1.04E-06	7	
A2M	-0.4029851	0	0.323	3.89E-81	7	Shared with clusters 0, 2, 3, and 7
FAM20C	-0.4063018	0	0.378	5.31E-88	7	Shared with clusters 0, 1, 2, 3, 4, 5, 6, and 7
ALDH2	-0.4162521	0	0.357	3.83E-46	7	Shared with clusters 0, 5, and 7
ATF3	-0.4194917	0.023	0.353	7.90E-05	7	Shared with clusters 5 and 7
HN1	-0.4195688	0	0.194	0.00286971	7	
ACSL4	-0.4212272	0	0.335	4.35E-66	7	
TREM2	-0.4245439	0	0.398	5.42E-37	7	Shared with clusters 0, 2, 3, 6, and 7
DOK2	-0.4245439	0	0.212	1.63E-05	7	
HM13	-0.4278221	0.023	0.279	0.00023547	7	Shared with clusters 5 and 7
PDE4D	-0.4278607	0	0.216	3.40E-16	7	
TNS1	-0.4311774	0	0.396	9.80E-39	7	Shared with clusters 0, 2, and 7
LINC-PINT	-0.4311774	0	0.222	5.55E-16	7	
CARD16	-0.4388137	0.279	0.531	2.90E-05	7	Shared with clusters 4 and 7
ATF5	-0.4394693	0	0.322	5.40E-05	7	

SLC6A6	-0.4394693	0	0.32	1.16E-15	7	
SLFN5	-0.4427861	0	0.197	1.33E-07	7	
CSNK1G2	-0.4444444	0	0.211	6.73E-05	7	
HIPK2	-0.4461028	0	0.312	1.81E-08	7	Shared with clusters 0, 2, and 7
CSF3R	-0.4461028	0	0.41	1.83E-19	7	Shared with clusters 5 and 7
RP11-489E7	-0.4461028	0	0.194	1.81E-16	7	
XRN1	-0.4461028	0	0.219	8.91E-08	7	
ADAM28	-0.4477612	0	0.373	6.58E-18	7	Shared with clusters 2, 5, and 7
HIST2H2AC	-0.4560531	0	0.204	0.00011866	7	Shared with clusters 5 and 7
ZNF160	-0.4593698	0	0.217	9.74E-05	7	Shared with clusters 2, 5, and 7
ZFYVE16	-0.4610282	0	0.347	3.32E-20	7	
SLC2A5	-0.4626866	0	0.418	2.97E-59	7	
CDKN1A	-0.4642678	0.023	0.456	2.67E-05	7	Shared with clusters 2 and 7
IL17RA	-0.4660033	0	0.365	8.30E-47	7	
CDKN2D	-0.4709399	0.023	0.262	1.88E-09	7	
CDA	-0.4776119	0	0.438	5.76E-14	7	Shared with clusters 2 and 7
CD96	-0.4790775	0.093	0.305	7.91E-09	7	
TNFAIP2	-0.4825871	0	0.438	1.68E-30	7	
SAP30	-0.4875622	0	0.221	3.07E-09	7	
ABCC3	-0.4892206	0	0.438	2.38E-38	7	Shared with clusters 2, 6, and 9
CASP4	-0.4908404	0.023	0.226	9.78E-05	7	
GADD45A	-0.4925373	0	0.201	5.66E-09	7	
NPHP3	-0.4941957	0	0.217	0.00028196	7	Shared with clusters 2, 5, and 7
S100A9	-0.5091211	0	0.473	1.19E-60	7	Shared with clusters 0, 1, 2, 3, 5, 6, and 7
DNAJC21	-0.5140962	0	0.24	2.08E-10	7	
BAZ1A	-0.5157546	0	0.224	0.00149414	7	
IER5L	-0.5240464	0	0.355	1.78E-32	7	Shared with clusters 2 and 7
RP11-290F2	-0.5273246	0.023	0.509	5.71E-06	7	
DUSP6	-0.5303714	0.093	0.63	4.84E-06	7	
FBLN5	-0.532184	0.07	0.282	1.30E-07	7	
RXRA	-0.5339967	0	0.484	4.29E-52	7	
CHI3L1	-0.5370049	0.093	0.615	5.35E-39	7	Shared with clusters 0, 1, 2, 3, 4, 5, 6, and 7
HNRNPUL1	-0.5489221	0	0.237	3.82E-13	7	
MKNK2	-0.5489221	0	0.224	5.12E-10	7	
SLC7A11	-0.5521617	0.023	0.494	5.96E-06	7	Shared with clusters 5 and 7
CD84	-0.555517	0.023	0.431	0.00018321	7	Shared with clusters 0, 1, 3, 5, 6, and 7
IVNS1ABP	-0.5588723	0	0.237	6.36E-07	7	Shared with clusters 2, 5, and 7
CD86	-0.5671642	0	0.532	5.99E-54	7	Shared with clusters 2, 3, and 7
PLAC8	-0.5864862	0.209	0.401	0.00204093	7	
PTPN12	-0.5920398	0	0.39	0.00092806	7	Shared with clusters 0, 2, and 7
HIST1H1E	-0.5970149	0	0.257	5.03E-06	7	Shared with clusters 1, 2, 5, and 7
PHC2	-0.5970149	0	0.476	6.66E-29	7	
TDP2	-0.6036484	0	0.479	0.00031284	7	Shared with clusters 2 and 7
FNDC3A	-0.6053068	0	0.471	2.49E-08	7	Shared with clusters 0, 2, 5, and 7
FGR	-0.6117475	0.047	0.635	3.63E-05	7	
FAM13A	-0.6202322	0	0.471	1.88E-25	7	Shared with clusters 0, 2, 5, and 7
TXNRD1	-0.6252073	0	0.428	4.20E-96	7	Shared with clusters 0, 1, 3, 4, 5, 6, and 7
SGK1	-0.6277141	0.465	0.947	5.58E-15	7	
IGF2R	-0.6368159	0	0.463	2.57E-29	7	
PURA	-0.6384743	0	0.279	1.31E-11	7	
EPB41	-0.6500829	0	0.3	3.53E-23	7	
CD36	-0.6514713	0.163	0.756	9.14E-14	7	Shared with clusters 3 and 7
CD109	-0.6517413	0	0.592	1.92E-21	7	Shared with clusters 2 and 7
ANPEP	-0.6549809	0.023	0.663	2.57E-07	7	
TPM4	-0.6567164	0	0.476	2.69E-56	7	Shared with clusters 3, 5, and 7
SCARB2	-0.6650083	0	0.62	1.45E-60	7	Shared with clusters 2, 3, and 7
HMG20B	-0.6729916	0.093	0.637	1.18E-06	7	Shared with clusters 0, 1, 3, 4, 5, and 7
NPL	-0.6749585	0	0.602	1.34E-37	7	
ITGAM	-0.6782753	0	0.614	1.58E-47	7	Shared with clusters 0, 2, and 7
ASH1L	-0.6782753	0	0.285	0.00029047	7	Shared with clusters 2, 5, and 7
SLAMF9	-0.6830961	0.07	0.721	1.44E-18	7	
PRPF4B	-0.6865672	0	0.284	0.00028466	7	
MGST1	-0.6877627	0.256	0.905	5.65E-12	7	
ABCA1	-0.6880713	0.047	0.73	0.00791487	7	Shared with clusters 0, 2, 3, and 10

PHLDA1	-0.6964017	0.047	0.619	1.47E-07	7	Shared with clusters 0, 2, and 7
PGD	-0.7063905	0.023	0.658	9.79E-07	7	Shared with clusters 5 and 7
APOE	-0.7145667	0.744	0.988	2.84E-24	7	Shared with clusters 0, 2, 3, 5, 6, and 7
CD4	-0.721393	0	0.534	3.56E-12	7	Shared with clusters 0 and 7
PTPN6	-0.7228971	0.047	0.541	0.00049339	7	Shared with clusters 0, 3, 5, 6, and 7
NAP1L4	-0.7230514	0	0.313	1.90E-22	7	Shared with clusters 2 and 7
CECR1	-0.7296849	0	0.65	4.76E-38	7	Shared with clusters 2 and 7
EREG	-0.7319989	0.535	0.93	1.36E-06	7	Shared with clusters 1, 2, 5, 6, and 7
CDKN1B	-0.7412164	0.023	0.352	1.56E-05	7	
ZCCHC2	-0.7412935	0	0.566	8.52E-06	7	Shared with clusters 4 and 7
TNFSF13B	-0.7429519	0	0.597	0.00121665	7	Shared with clusters 0, 2, and 7
TMEM91	-0.744456	0.047	0.693	0.00725994	7	Shared with clusters 2, 5, and 7
IL4I1	-0.7462687	0	0.629	7.37E-35	7	Shared with clusters 2, 3, and 7
FCGR2B	-0.7539435	0.279	0.955	9.93E-10	7	Shared with clusters 0, 5, and 7
H2AFJ	-0.7628524	0	0.502	8.91E-12	7	
CD14	-0.7645108	0	0.723	4.28E-38	7	
GADD45B	-0.767519	0.093	0.352	3.47E-06	7	
MARCKS	-0.7711443	0	0.622	0.00186486	7	
VAT1	-0.7844113	0	0.695	1.27E-16	7	
IL1RN	-0.7875738	0.047	0.811	3.96E-32	7	Shared with clusters 0, 1, 3, 4, 5 6, and 7
CTSL	-0.797254	0.209	0.847	7.68E-28	7	Shared with clusters 0, 1, 2, 3, 5, 6, and 7
LIPA	-0.8049288	0.512	0.972	9.50E-91	7	Shared with clusters 0, 1, 2, 3, 4, 5, 6, and 7
FERMT3	-0.8108681	0.023	0.662	1.60E-06	7	Shared with clusters 0, 1, 3, 5, 6, and 7
GM2A	-0.8108681	0.023	0.789	4.59E-11	7	Shared with clusters 0, 2, 3, 5, 6, and 7
RNASE6	-0.8124879	0.023	0.819	0.00027441	7	Shared with clusters 5 and 7
HMOX1	-0.8175788	0	0.741	1.27E-51	7	Shared with clusters 5 and 7
ITGAX	-0.8192371	0	0.7	1.48E-46	7	Shared with clusters 5 and 7
CSTA	-0.8225539	0	0.771	7.92E-11	7	
RARRES3	-0.8235952	0.163	0.486	1.46E-05	7	
HLA-F	-0.834124	0.023	0.367	0.00063733	7	
C5AR1	-0.8374793	0	0.796	1.51E-131	7	
CSF1R	-0.8391376	0	0.793	5.43E-53	7	
FAM46A	-0.8391376	0	0.688	2.32E-32	7	
RGCC	-0.8391762	0.814	0.95	8.03E-22	7	Shared with clusters 0, 1, 5, 6, and 7
GUSB	-0.8407189	0.023	0.715	7.40E-14	7	Shared with clusters 0, 1, 2, 3, 5, 6, and 7
APOC1	-0.8418759	0.558	0.99	4.49E-33	7	Shared with clusters 0, 1, 2, 3, 4, 5, 6, and 7
ATP6AP1	-0.8423773	0.023	0.774	2.28E-05	7	
GIMAP1	-0.8456169	0.07	0.406	0.0007078	7	
FOSL2	-0.8506306	0.07	0.698	0.00845087	7	Shared with clusters 2, 5, and 7
MAFB	-0.8553743	0.186	0.962	3.83E-14	7	
CTSA	-0.8656716	0	0.73	8.82E-33	7	Shared with clusters 2 and 7
BCAT1	-0.8689884	0	0.799	2.11E-12	7	Shared with clusters 2 and 7
ADAMDEC1	-0.8706468	0	0.819	9.44E-61	7	
LGALS2	-0.8888889	0	0.784	4.00E-43	7	Shared with clusters 2, 6, and 7
TMEM176B	-0.893864	0	0.811	2.61E-14	7	Shared with clusters 0, 2, and 7
MT1X	-0.9028887	0.488	0.872	1.11E-27	7	Shared with clusters 0, 1, 3, 4, 5, 6,and 7
KIAA0930	-0.9054726	0	0.804	1.94E-61	7	Shared with clusters 0, 2, 3, 5, and 7
FCN1	-0.9203594	0.023	0.881	5.47E-18	7	Shared with clusters 0, 4, and 7
GAA	-0.9253731	0	0.834	7.52E-70	7	
GOLGA8A	-0.9285356	0.047	0.405	4.68E-05	7	Shared with clusters 2, 6, and 7
FUCA1	-0.9286899	0	0.846	9.75E-145	7	Shared with clusters 3, 4, 5, and 7
TGFBI	-0.9436153	0	0.851	8.24E-75	7	Shared with clusters 2, 4, 5, and 7
CCDC88A	-0.9502488	0	0.846	3.46E-113	7	Shared with clusters 3, 5, and 7
SULF2	-0.9534112	0.047	0.902	0.00826192	7	
GLRX	-0.9535655	0	0.509	9.96E-08	7	
GNS	-0.9552239	0	0.867	1.08E-78	7	
PLAUR	-0.9779398	0.186	0.978	1.24E-10	7	
PLEK	-0.9783254	0.047	0.708	4.42E-06	7	Shared with clusters 0, 5, 6, and 7
ACP5	-0.9876972	0.395	0.973	1.04E-15	7	Shared with clusters 0, 2, 5, and 7
LAMP1	-0.9929423	0.233	0.905	1.52E-15	7	Shared with clusters 0, 2, 3, 6, and 7
PIK3IP1	-0.997956	0.163	0.483	0.00046084	7	
HEXB	-1.0066335	0	0.852	1.24E-63	7	Shared with clusters 2 and 7
EPB41L3	-1.0115315	0.023	0.965	7.94E-06	7	
CYP1B1	-1.013267	0	0.925	5.67E-100	7	Shared with clusters 0, 1, 2, 3, 5, and 7

CTSH	-1.0148868	0.023	0.909	8.09E-25	7	Shared with clusters 0, 2, and 7
BSG	-1.0197462	0.07	0.789	0.00181896	7	
IFNGR2	-1.0232172	0	0.904	7.74E-15	7	
CAPG	-1.0330904	0.047	0.876	5.80E-80	7	Shared with clusters 0, 1, 2, 3, 5, 6, and 7
FCGR2A	-1.0464345	0	0.95	1.76E-56	7	Shared with clusters 0, 5, and 7
LMNA	-1.0595087	0.047	0.879	4.31E-05	7	Shared with clusters 5 and 7
DMXL2	-1.0629411	0.023	0.965	1.60E-07	7	Shared with clusters 0, 2, 5, and 7
RAPGEF1	-1.066335	0	0.841	1.26E-46	7	
PPP1R14B	-1.0679934	0	0.756	5.05E-42	7	Shared with clusters 5, 6, and 7
ATP6V0B	-1.07212	0.256	0.978	0.00062024	7	Shared with clusters 5 and 7
SLC11A1	-1.081029	0.07	0.975	5.12E-08	7	Shared with clusters 0, 2, and 7
ZRANB2	-1.0828416	0.023	0.416	0.00631314	7	
LYAR	-1.0908249	0.116	0.405	0.00501218	7	
KIAA1551	-1.0961857	0	0.413	8.57E-30	7	Shared with clusters 0, 2, 5, and 7
PLA2G7	-1.0995025	0	0.972	1.30E-41	7	Shared with clusters 5 and 7
GSN	-1.1028192	0	0.96	1.93E-87	7	Shared with clusters 0, 1, 2, 3, 5, 6, and 7
HEXA	-1.1309345	0.023	0.899	9.02E-08	7	Shared with clusters 2 and 7
CCL4	-1.1538818	0.047	0.295	2.62E-11	7	
BASP1	-1.1657989	0.023	0.982	1.02E-09	7	
FBP1	-1.1674573	0.023	0.968	8.66E-58	7	Shared with clusters 0, 1, 2, 3, 5, 6, and 7
PTPRE	-1.1691542	0	0.93	6.52E-75	7	
SLC16A3	-1.1721624	0.116	0.955	6.22E-07	7	
HK2	-1.1740137	0.047	0.972	0.00058206	7	Shared with clusters 0, 2, 3, and 7
CD9	-1.1831926	0.535	0.988	6.52E-58	7	Shared with clusters 1, 2, 5, and 7
BLVRB	-1.1984265	0.349	0.987	4.18E-23	7	
VEGFA	-1.2072968	0	0.846	2.82E-08	7	Shared with clusters 0, 2, and 7
IL32	-1.2128119	0.302	0.574	2.35E-13	7	
H2AFY	-1.2132361	0.419	0.988	6.69E-52	7	Shared with clusters 5 and 7
MFSD12	-1.2138532	0.023	0.844	7.47E-27	7	Shared with clusters 0, 1, 2, 3,4, 5, 6, and 7
AIF1	-1.2297042	0.419	0.98	7.28E-10	7	Shared with clusters 2 and 7
ATP6AP2	-1.2351421	0.093	0.97	3.38E-07	7	Shared with clusters 0, 1, 2, 3, 5, and 7
ANXA2	-1.2380346	0.326	0.992	4.32E-16	7	Shared with clusters 0, 1, 2, 3, 5, 6, and 7
FCGR3A	-1.2453623	0.023	0.95	1.11E-08	7	Shared with clusters 0, 2, 5, and 7
FCGRT	-1.2561611	0.419	0.985	2.06E-07	7	
CALM3	-1.2569324	0.047	0.829	0.00338297	7	Shared with clusters 0, 2, 3, 6, and 7
HIST1H1D	-1.2587065	0	0.421	2.45E-28	7	Shared with clusters 5 and 7
BCAP31	-1.2668055	0.047	0.964	2.82E-10	7	Shared with clusters 2, 3, 6, and 7
TIMP2	-1.2769486	0	0.982	8.76E-62	7	Shared with clusters 0, 2, 5, and 7
CYP27A1	-1.291604	0.14	0.985	4.93E-19	7	
NCF2	-1.3365344	0.047	0.978	1.82E-12	7	Shared with clusters 2 and 7
LRP1	-1.3416252	0	0.978	1.26E-60	7	
LIMS1	-1.401211	0.047	0.968	9.70E-06	7	Shared with clusters 1, 5, and 7
MT2A	-1.4022523	0.721	0.987	5.73E-17	7	Shared with clusters 0, 2, 1, 3, 4, 5, 6, and 7
ANXA5	-1.4344942	0	0.99	1.17E-70	7	Shared with clusters 1, 5, 6, and 7
TYMP	-1.4375024	0.163	0.97	4.01E-17	7	Shared with clusters 3, 5, and 7
VMP1	-1.4790389	0.093	0.919	0.00187535	7	
CEBPD	-1.4941957	0	0.975	0.00379449	7	Shared with clusters 5 and 7
FLNA	-1.5006364	0.093	0.985	3.01E-16	7	
PRDX1	-1.5036446	0.233	0.985	2.64E-14	7	Shared with clusters 0, 1, 2, 3, 5, 6, and 7
TALDO1	-1.5105095	0.07	0.987	1.86E-07	7	Shared with clusters 2, 5, 6, and 7
CD68	-1.5486907	0.953	0.99	4.84E-12	7	
GSTO1	-1.5596822	0.442	0.988	1.81E-06	7	Shared with clusters 0, 3, 5, 6, and 7
ZEB2	-1.5703267	0.047	0.985	8.73E-05	7	Shared with clusters 0, 2, and 7
LRPAP1	-1.5920398	0	0.968	7.89E-23	7	Shared with clusters 2 and 7
TNFRSF1B	-1.6114004	0.256	0.985	4.46E-07	7	
LSP1	-1.6345019	0.349	0.992	0.00100715	7	Shared with clusters 5 and 7
VCAN	-1.6766169	0	0.983	6.16E-147	7	Shared with clusters 0, 2, 5, 6, and 7
CALR	-1.7012611	0.07	0.978	0.00043878	7	Shared with clusters 2 and 7
PTPRCAP	-1.7023024	0.349	0.72	4.43E-13	7	
PLD3	-1.7070847	0.535	0.992	5.80E-14	7	Shared with clusters 0 and 7
CFL1	-1.7559875	0.512	0.985	0.00139665	7	Shared with clusters 0, 1, 2, 3, 5, 6, and 7
GRN	-1.7582629	0.581	0.997	2.70E-08	7	Shared with clusters 2 and 7
TUBA1B	-1.8431872	0.233	0.99	0.00036325	7	Shared with clusters 0, 1, 3, 5, 6, and 7
PSAP	-1.8986849	0.791	0.987	2.10E-12	7	Shared with clusters 3 and 7

CTSZ	-1.9369818	0	0.99	4.79E-64	7	Shared with clusters 0, 1, 2, 3, 6, and 7
CEBPB	-1.9440395	0.698	0.992	0.00010249	7	
GPNMB	-1.9770142	0.744	0.983	1.76E-10	7	
APLP2	-1.999113	0.395	0.988	1.42E-08	7	
DDIT4	-1.9994215	0.186	0.841	9.33E-08	7	
ARPC1B	-2.0344016	0.116	0.965	2.13E-09	7	Shared with clusters 0, 1, 2, 3, 5, 6, and 7
CST7	-2.0477072	0.14	0.405	4.43E-09	7	Shared with clusters 6 and 7
FABP5	-2.0850785	0.512	0.99	7.36E-75	7	Shared with clusters 0, 1, 2, 3, 4, 5, 6, and 7
LTB	-2.1937599	0.558	0.609	1.42E-05	7	Shared with clusters 2, 6, and 7
MMP9	-2.2378032	0.512	0.988	3.57E-83	7	Shared with clusters 0, 1, 2, 3, 4, 5, 6, and 7
TIMP1	-2.2692738	0.581	0.988	1.86E-13	7	Shared with clusters 2, 3, 5, 6, and 7
LCP1	-2.3415481	0.023	0.99	0.00380849	7	
CLEC2B	-2.3658452	0.186	0.897	0.00243346	7	Shared with clusters 5 and 7
CTSD	-2.43353	0.953	0.988	8.81E-19	7	
SH3BGRL3	-2.6257087	0.93	0.998	0.00075832	7	Shared with clusters 6 and 7
CD52	-2.6356589	0.953	0.987	2.00E-06	7	Shared with clusters 2, 6, and 7
CTSB	-2.6722589	0.837	0.988	6.72E-19	7	
LYZ	-2.7006826	1	0.998	4.95E-05	7	Shared with clusters 3 and 7
RNASE1	-2.7295692	0.047	0.982	8.11E-19	7	Shared with clusters 0, 4, and 7
ITGB2	-2.806433	0.558	0.985	0.00036465	7	
HCST	-2.8419916	0.651	0.987	5.23E-06	7	Shared with clusters 2, 6, and 7
CD63	-2.8701454	0.977	1	1.10E-27	7	Shared with clusters 0, 1, 2, 3, 4, 5, 6, and 7
TMBIM6	-2.9378688	0.349	0.995	0.00012097	7	Shared with clusters 1 and 7
LGALS3	-2.9868101	0.837	0.997	1.37E-45	7	Shared with clusters 0, 1, 2, 3, 4, 5, 6, and 7
ZFP36L1	-3.1999306	0.93	0.998	1.93E-07	7	Shared with clusters 0, 2, 5, 6, and 7
DDX17	-3.263643	0.023	0.997	0.00088035	7	Shared with clusters 2, 5, and 7
S100A11	-3.3681592	0.977	0.998	6.54E-18	7	Shared with clusters 1, 5, 6, and 7
PLIN2	-3.4834355	0.977	0.987	3.89E-05	7	Shared with clusters 0, 2, and 7
CCL5	-4.2312469	0.209	0.446	1.69E-16	7	Shared with clusters 0, 6, and 7
IL7R	-4.2874773	0.326	0.638	1.13E-17	7	Shared with clusters 0, 2, 3, 6, and 7
CSTB	-4.3081492	1	1	1.77E-12	7	Shared with clusters 0, 1, 5, 6, and 7
SRGN	-4.5102009	0.814	0.997	3.30E-08	7	
SPP1	-4.7752324	0.977	0.997	6.80E-63	7	Shared with clusters 0, 1, 2, 3, 4, 5, 6, and 7
ACTB	-4.8595781	0.86	1	9.01E-05	7	Shared with clusters 0, 1, 3, 5, 6, and 7
ZFP36L2	-7.2770257	0.465	0.992	0.00013205	7	Shared with clusters 5 and 7
JUND	-7.6957075	0.233	1	5.56E-05	7	
LDHA	-10.436268	0.93	1	0.00306586	7	
BTG1	-14.348529	1	1	1.33E-06	7	Shared with clusters 0, 2, 6, and 7
EIF1	-18.511975	1	1	3.90E-12	7	
GAPDH	-19.319526	1	1	2.91E-05	7	
VIM	-19.392572	1	1	0.00064589	7	Shared with clusters 4 and 7
RPL7A	-29.875005	1	1	3.44E-09	7	Shared with clusters 0, 1, 4, 5, 6, and 7
RPS27A	-48.208994	1	1	0.00062431	7	Shared with clusters 0, 6, and 7
RPL41	-49.539782	1	1	2.11E-08	7	Shared with clusters 0, 2, and 7
RPS3	-50.806394	1	1	0.00202039	7	Shared with clusters 0, 5, 6, and 7
RPS27	-54.231208	1	1	1.20E-06	7	Shared with clusters 0, 2, and 7
RPL30	-60.728682	1	1	2.82E-05	7	Shared with clusters 0, 6, and 7
MT-ND4	-71.701454	1	1	0.00162843	7	Shared with clusters 0, 2, 3, 5, and 7
FTL	-75.980794	1	1	5.72E-05	7	Shared with clusters 0 and 7
RPL10	-77.853677	1	1	7.97E-05	7	Shared with clusters 0, 4, and 7
TPT1	-87.508928	1	1	8.97E-12	7	Shared with clusters 1, 6, and 7

Footnotes:

1- Value refers to average differential expression within one subset of scaled pearsons residuals. Positive values represent increased gene expression in HIV infected samples without ART. Negative values represent increased gene expression in HIV infected samples with ART.

2- Percentage of cells, within the cluster ID for which the gene is a marker, that detect the gene

3- Percentage of all the other cells, excluding the cluster ID for which the gene is a marker, that detect the gene
