# Supplementary material for: Interactions of Monocytes, HIV, and ART Identified by an Innovative scRNAseq Pipeline: Pathways to Reservoirs and HIV-Associated Comorbidities
Source: mBio. 2020 Jul 28;11(4):e01037-20. doi: 10.1128/mBio.01037-20 (PMC7387797; doi:10.1128/mBio.01037-20)
Supplement: TABLE S5 [file mBio.01037-20-st005.pdf]

Supplementary Table 5: List of differentially expressed genes between cluster 8 and all other monocyte clusters from the integrated dataset of HIV-infected mature monocytes with and without ART-treatment (Cluster 8 vs all other clusters).

Gene.ID	Avg. Diff.[1]	Pct.1[2]	Pct.2[3]	P.Value.Adj
FTL	1054.17391499605	1	1	3.32879859429307E-121
SPP1	155.8135398	1	0.993	7.54149861834965E-34
MT-CO1	47.0183666	1	0.994	0.000164255
TMSB4X	44.3558083	1	0.999	7.58881345345325E-14
CSTB	25.20515703	1	1	1.6247044821077E-22
MT-CO3	17.69510556	1	0.974	1.90163685256304E-08
S100A11	16.28156743	1	0.996	5.91862108825992E-14
VIM	14.93026188	1	1	0.00416233
MT-ATP6	14.38791994	1	0.956	1.67995375661364E-25
ACTB	13.51531278	1	0.974	0.006327628
LGALS3	12.68822181	0.998	0.98	1.42369138538532E-07
FABP5	9.745828353	0.998	0.684	3.55004705777801E-17
SH3BGRL3	9.65976577	1	0.977	1.07122484427575E-12
MT-ND1	7.946783528	1	0.939	4.58496842732316E-33
OAZ1	7.550074876	1	0.994	1.73750431905935E-09
CTSD	7.321855791	1	0.993	0.000149909
MT-ND5	5.568202231	1	0.882	5.20175668050359E-14
MYL6	5.547623655	1	0.981	8.22933869780367E-13
HLA-B	4.819649938	1	0.997	1.18553357068471E-32
MT-ND3	4.393184513	1	0.923	2.61906283888829E-87
YBX1	4.01770521	1	0.984	3.56672971556661E-07
ARPC1B	3.691988869	1	0.715	7.1750707093598E-05
MT-ND2	3.65257174	1	0.952	2.86235030814425E-46
GRN	2.828861203	0.996	0.917	7.45855068564417E-08
HLA-C	2.572052983	1	0.962	8.59568400230214E-12
PLD3	2.223104754	1	0.913	7.38272643842793E-16
MTRNR2L12	2.122970517	1	0.787	1.60955412462066E-11
PSAP	1.621749806	1	0.982	4.48860149254226E-36
HLA-A	1.529599999	1	0.997	6.06137955992518E-67
SRGN	1.52076597	1	0.984	3.42179409557055E-12
RPS29	1.51885325	0.998	0.995	1.80193137092758E-19
GPNMB	1.444073468	0.996	0.981	3.21723006629231E-21
FABP4	1.186870864	0.295	0.206	3.94602080394931E-08
LIPA	1.136328526	0.996	0.646	1.54781552270101E-07
LGALS2	1.120550733	0.97	0.345	1.14545285301388E-05
NPC2	1.017722765	1	0.999	6.75693527602974E-05
MT-ND4L	0.903087378	0.975	0.604	1.68640603054349E-29
LDHA	0.77860465	1	0.986	0.000283825
PTPRE	0.736058173	0.97	0.733	0.005878114
C5AR1	0.671297204	0.96	0.595	1.06817847089939E-05
FUCA1	0.665517402	0.966	0.542	1.19406022666453E-30
HEXA	0.631087225	0.975	0.704	2.72610458190641E-07
TMEM176B	0.599315667	0.956	0.509	4.75518392395881E-19
CSF1R	0.591343484	0.962	0.595	4.02052671695708E-10
LRP1	0.542856758	0.979	0.848	3.23999252748414E-21
GNS	0.53703368	0.989	0.709	0.003393322
GAA	0.536442921	0.973	0.686	4.15936711621081E-07

LRPAP1	0.533293977	0.977	0.78	8.02323942783449E-07
RNASE6	0.527955917	0.966	0.66	8.99178882839105E-13
STAB1	0.487117211	0.932	0.306	1.98826327262556E-12
SLC16A3	0.48039146	0.979	0.767	3.03415674838143E-11
GPRIN3	0.468842064	0.858	0.342	0.00198956
PARP14	0.466343076	0.898	0.354	1.56133775181987E-08
ZRANB2	0.458353747	0.824	0.319	7.40357275669691E-06
HIST1H4C	0.456562282	0.947	0.389	0.000518368
CD36	0.455246445	0.966	0.557	0.008951412
RNF213	0.434733599	0.932	0.387	1.57214350293356E-13
CD74	0.43263267	0.994	0.796	1.73116147728195E-38
IL17RA	0.410023794	0.943	0.459	0.000896745
NABP1	0.409537959	0.938	0.44	1.42926020468449E-05
A2M	0.408435236	0.953	0.381	1.97638776286996E-05
TTYH3	0.402933463	0.816	0.34	4.81033746495088E-08
GTF2I	0.402905292	0.907	0.401	9.37053801499188E-08
NCF4	0.395955813	0.869	0.387	0.000105445
MGAT1	0.395878242	0.983	0.906	1.97899334698364E-07
PRPF4B	0.391700471	0.867	0.392	3.10180024243503E-06
KLF13	0.389564839	0.867	0.388	1.46801572920205E-06
C1QA	0.380515246	0.911	0.242	6.30179558176944E-12
GATAD1	0.379988585	0.892	0.401	3.4189110844844E-07
HEXB	0.374686453	0.981	0.682	6.29910287794977E-07
VMP1	0.373231398	0.972	0.715	1.77378207565499E-23
PARP4	0.373221599	0.818	0.388	0.007877213
MAF	0.358638389	0.919	0.371	6.41493518441802E-17
MACF1	0.358615526	0.934	0.433	1.32424185474819E-11
CCR1	0.352907579	0.928	0.436	3.60181565056967E-06
HLA-DMB	0.352405822	0.737	0.26	6.50773228855282E-14
SCPEP1	0.35202736	0.949	0.413	1.45036201073689E-06
CTSC	0.349943578	0.92	0.375	5.40521735195953E-18
ARL4C	0.347230251	0.949	0.494	3.32962740603222E-67
LPAR6	0.339666742	0.854	0.347	1.04446717320654E-12
IER5L	0.337492733	0.938	0.457	0.000954822
ADAMDEC1	0.332653165	0.953	0.57	4.10856584874454E-31
ARGLU1	0.325189271	0.943	0.463	4.46317467600364E-13
PLEC	0.316110284	0.746	0.363	0.0030766
PTAFR	0.314945097	0.926	0.419	2.23617795259232E-21
SEMA6B	0.31261064	0.741	0.333	3.86860307520903E-09
MIR142	0.311372373	0.867	0.381	3.92981772131714E-15
TKT	0.311068624	0.998	0.935	0.001455167
IL10RA	0.308292017	0.93	0.493	0.000591304
GOLGB1	0.307330962	0.936	0.444	6.35762032620875E-12
CFDP1	0.305252079	0.841	0.427	1.0567630393735E-05
CD4	0.302377896	0.943	0.474	0.000315759
FYB	0.297275814	0.958	0.541	1.67795818606299E-11
PRPF38B	0.295786056	0.909	0.462	9.74575693442563E-10
AMICA1	0.295079759	0.824	0.369	8.82646429984342E-15
TMEM176A	0.293289927	0.936	0.399	2.21805648871912E-10
UBC	0.288414023	0.985	0.992	0.005253786
CLMN	0.287440312	0.888	0.438	1.66594670122937E-07
ZKSCAN1	0.281547421	0.919	0.436	3.07474309868279E-16

KDM2A	0.281082816	0.831	0.422	3.16688658572818E-10
APPL1	0.274499549	0.886	0.445	4.02186761259552E-09
SLC2A1	0.273604633	0.883	0.375	7.3644187328067E-10
PCM1	0.273575647	0.932	0.499	0.000279325
CSF2RA	0.272499053	0.884	0.43	4.59572505653289E-10
DOCK8	0.270499373	0.792	0.41	9.41399145584061E-05
FAM118A	0.268314749	0.547	0.229	3.55227882974779E-14
STK4	0.265725943	0.934	0.483	1.18918534594064E-19
GGA1	0.265403006	0.822	0.424	2.49105749746767E-10
TNFSF13B	0.259368447	0.956	0.448	4.48151908443732E-18
RSF1	0.257190764	0.784	0.422	0.005330079
ABCC3	0.256723709	0.949	0.496	1.90219694357572E-13
KMT2C	0.25576837	0.888	0.467	6.18979542999042E-05
LINC00969	0.251325635	0.941	0.525	7.78061626939619E-13
ASH1L	0.249811382	0.83	0.426	1.26948812390067E-09
PTTG1IP	0.244148752	0.928	0.468	1.30919935887312E-08
S100A9	0.242538965	0.964	0.434	8.40604808772897E-10
ST8SIA4	0.241417054	0.797	0.355	1.88914329348381E-28
RAPGEF1	0.238688622	0.972	0.672	8.29750198146595E-07
AKAP9	0.238551853	0.934	0.486	7.07994003488425E-14
DDX17	0.235168973	0.981	0.8	1.08736498188417E-08
PTPRC	0.233181541	0.981	0.769	1.65859937847614E-14
DDX24	0.230525371	0.951	0.51	6.60508429885301E-12
CLN8	0.223565685	0.939	0.508	5.59784654422762E-11
BOD1L1	0.223180283	0.941	0.507	1.17351238053135E-10
U2AF1	0.222754463	0.932	0.548	0.002766444
ICAM3	0.217414362	0.913	0.395	1.70099619243315E-30
ZBTB1	0.215373448	0.917	0.505	0.005568854
AHR	0.214609585	0.928	0.502	0.000425711
RAP2B	0.211398176	0.949	0.511	1.5297270343445E-08
GK	0.211246301	0.826	0.431	2.76489328856447E-06
CEP350	0.209133124	0.78	0.4	9.84697388432814E-24
TAOK1	0.206308749	0.665	0.38	0.001619282
HLA-DRB1	0.20471121	0.968	0.559	5.64811266943332E-38
PRRC2C	0.204267835	0.972	0.748	6.80944879774168E-15
CELF2	0.204152704	0.926	0.474	1.80701613042226E-12
CD86	0.199896954	0.958	0.556	0.002432873
YIPF4	0.199712418	0.86	0.441	6.12040849742009E-30
NOTCH2	0.198476193	0.92	0.528	0.000211018
TNRC6B	0.194938989	0.93	0.489	3.8895002570734E-16
TFPI	0.194605845	0.83	0.435	1.33201110044366E-11
TNFAIP2	0.194394364	0.951	0.509	1.70493437069423E-10
ATRX	0.19180515	0.938	0.506	1.56229004040696E-11
SLC43A2	0.190976781	0.926	0.442	4.58339078891382E-29
NPEPPS	0.189842214	0.915	0.509	3.48822419799889E-05
HLA-DMA	0.18823406	0.955	0.571	0.000426317
FAM133B	0.185360694	0.884	0.492	3.73570621995626E-05
STAT3	0.184965085	0.915	0.499	7.608433370303E-19
PDCD4	0.182199093	0.805	0.438	4.81441731815222E-18
RSRC2	0.179456371	0.936	0.552	6.30376083968023E-05
CSF3R	0.179015854	0.943	0.495	3.17697957531472E-20
TMEM59	0.178614121	0.985	0.834	0.000553946

DICER1	0.175255737	0.947	0.55	4.85018535838969E-10
TMF1	0.174178327	0.928	0.525	0.000668399
FMNL1	0.17180141	0.947	0.566	0.000182739
HP1BP3	0.171022033	0.947	0.571	1.82163245031812E-05
PTBP3	0.170897513	0.924	0.502	2.76617530192694E-13
HIPK2	0.16907951	0.938	0.525	0.0014452
ZFYVE16	0.167401543	0.939	0.531	9.00609746385709E-07
ARID4A	0.167340711	0.79	0.438	1.97643906056853E-12
LGALS8	0.166784655	0.693	0.418	0.000816018
MPHOSPH8	0.166235131	0.953	0.556	1.18442835288718E-38
RBM25	0.166096729	0.934	0.547	3.98062427887326E-09
HLA-DPB1	0.16497686	0.953	0.406	6.84087553468423E-17
ARID4B	0.164652289	0.922	0.528	1.54109873005628E-08
SAMSN1	0.164563288	0.693	0.38	0.000322961
PNN	0.164004782	0.93	0.507	2.76300182562627E-24
IL6R	0.162830204	0.652	0.363	2.98788200003216E-08
FOXO3	0.162116558	0.928	0.508	5.1537675717335E-08
SPEN	0.161801786	0.718	0.416	6.50355500396547E-05
STX7	0.161396379	0.939	0.555	1.66577757281051E-06
LRRFIP1	0.160396947	0.985	0.899	0.000892454
TMEM30A	0.159735151	0.903	0.506	0.000437964
PRKAG2	0.157732613	0.949	0.556	3.50026038341807E-06
CFLAR	0.156247346	0.968	0.745	2.0267670452501E-24
PHF21A	0.154463638	0.826	0.441	1.32196503109716E-21
YME1L1	0.143814465	0.913	0.526	4.38756713529195E-05
KMT2A	0.142000137	0.913	0.476	1.60471498531102E-35
MIDN	0.141765385	0.932	0.488	5.42049803657098E-08
SMCHD1	0.141694347	0.913	0.515	8.47511851615405E-10
RAD21	0.140943548	0.742	0.449	0.000324756
MIS18BP1	0.139997191	0.936	0.517	1.70313318770787E-10
NUFIP2	0.139959631	0.915	0.509	6.98042951355504E-11
GPATCH2L	0.138754842	0.938	0.525	7.21641535719392E-13
TRIM38	0.135000849	0.93	0.492	5.22491304303349E-34
TET2	0.133584171	0.733	0.436	0.000416699
TNFRSF1B	0.133557634	0.981	0.892	2.15230911810384E-18
LAPTM5	0.132752128	1	0.984	2.87838402661212E-09
ZCCHC6	0.131857212	0.928	0.521	2.71344491986554E-06
MDM4	0.126027194	0.938	0.54	8.60824567462401E-13
PABPN1	0.121358688	0.598	0.394	1.58264436950671E-05
WAC	0.118848678	0.907	0.53	2.44443002067687E-07
FPR3	0.117491606	0.491	0.311	0.00078591
CPVL	0.117458537	0.943	0.473	4.32843856868763E-24
GOLGA4	0.114423089	0.938	0.524	2.22813457335312E-14
ANKRD11	0.114315308	0.949	0.539	5.56075514313143E-20
LUCAT1	0.107018803	0.92	0.462	1.63637441612681E-38
LUC7L3	0.106959605	0.943	0.562	4.8291208154142E-17
CDA	0.106163489	0.956	0.544	4.31355118103148E-06
AKAP10	0.103548962	0.782	0.452	3.31704923434851E-14
ZNF638	0.094937846	0.922	0.53	1.05244490227531E-15
RREB1	0.092338833	0.58	0.383	4.58516117551453E-08
ZNF148	0.091319397	0.653	0.405	7.26441578043951E-11
TGFBI	0.090426114	0.972	0.586	1.00146329794524E-42

C9orf72	0.088010821	0.812	0.476	1.65986705190674E-05
RNF145	0.083496639	0.809	0.485	4.59057424792545E-15
CASP4	0.080085589	0.672	0.441	0.00346468
ANPEP	0.080023941	0.966	0.614	2.90020132413017E-08
ATF5	0.077409006	0.943	0.469	0.000120956
CD82	0.075797586	0.424	0.306	4.5335807739816E-06
ZMYM2	0.074472767	0.909	0.53	4.67037351241834E-22
FAM63B	0.074166569	0.919	0.527	8.42884203791691E-10
ATXN1	0.066874555	0.932	0.537	1.17531468532399E-09
SBF2	0.064978166	0.75	0.476	2.48589166216781E-08
TBL1XR1	0.061759408	0.616	0.404	1.76297107988896E-17
HNRNPUL1	0.059429034	0.561	0.405	0.00259343
CECR1	0.058494924	0.955	0.546	5.57758785944169E-12
CYP1B1	0.051124116	0.979	0.63	1.24673672222861E-27
PKN2	0.050579899	0.549	0.378	1.11185581694952E-07
ATM	0.04951637	0.951	0.581	8.57846401448518E-14
CHURC1	0.045906494	0.634	0.441	4.34899111353652E-05
NKTR	0.044326919	0.939	0.523	6.16737297226646E-35
JAK1	0.041898561	0.958	0.618	0.001726945
JARID2	0.039965428	0.938	0.543	5.73676161854901E-11
IDS	0.034568987	0.958	0.607	0.00060246
SFPQ	0.033815739	0.917	0.547	4.32943766473498E-06
IVNS1ABP	0.033627529	0.699	0.456	4.45076824490831E-09
LPP	0.031978956	0.938	0.533	1.24236667148703E-15
PHF3	0.028502583	0.956	0.586	1.51802368353559E-06

Footnotes:

1- Value refers to average differential expression within one subset of scaled pearsons residuals.

2- Percentage of cells, within the cluster ID for which the gene is a marker, that detect the gene

3- Percentage of all the other cells, excluding the cluster ID for which the gene is a marker, that detect the gene
